# Stereofacial Assembly of Engineered Multichiral Aziridines via B/Si Ylide Insertion

**DOI:** 10.1021/jacsau.5c01306

**Published:** 2025-11-25

**Authors:** Mireia Pujol, Luis Tarifa, Anika Tarasewicz, María Méndez, Elena Fernández

**Affiliations:** † Faculty of Chemistry, University Rovira i Virgili, 43007 Tarragona, Spain; ‡ Sanofi R&D, Integrated Drug Discovery, Industrie Park Höchst, Bldg. G838, 65926 Frankfurt am Main, Germany

**Keywords:** aziridination, B,Si-disubstituted aziridine, stereofacial control, chiral transmission, functionalization

## Abstract

Halo-borylsilylcarbanion reagents can be added, with complete stereofacial control, to chiral *N*-*tert*-butanesulfinyl imines, featuring an asymmetric C–C bond, followed by concomitant intramolecular asymmetric C–N bond formation. There is exclusive access to α,α-B,Si-disubstituted aziridine units containing up to four contiguous stereocenters in a single operation. In addition, complete stereochemical discrimination has been observed in *N*-*tert*-butanesulfinyl alkyl aldimines. Post-transformation of B,Si-disubstituted aziridine generates multichiral aziridine scaffolds.

## Graphical abstract

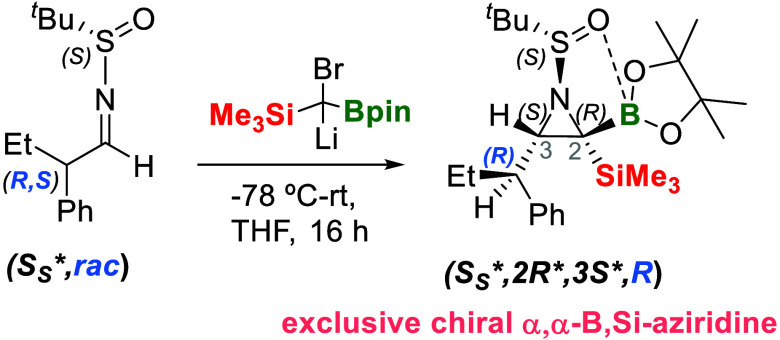

## Introduction

Aziridines are considered structural versatile components present in many biologically active products.^1^ Boron-substituted aziridines combine the ring strain properties of the three-membered ring with the transformable C–B bond, providing strategic building blocks for the construction of complex molecules. Notwithstanding the potential of the boron-substituted aziridine units, the synthesis of these molecular motifs has been rather unexplored. The available strategy for the preparation of B-substituted aziridines relies on diastereoselective aziridination of Bpin-substituted allylic alcohols reacting with *N*-aminophthalimide, as the nitrogen source, in the presence of PhI­(OAc)_2_.^2^ The access to asymmetric B-substituted aziridines has only been addressed through iridium-catalyzed enantioselective C­(sp^3^)–H borylation of *meso-*aziridines in the presence of chiral bidentate boryl ligands,^3^ showing high dependence on substrates and ligand nature (Scheme 1a). This suggests that the asymmetric synthesis of B-substituted aziridines with total control of the stereoselectivity remains a formidable challenge, particularly for drug discovery programs. In that scenario, our work hypothesis postulates the use of halo-borylcarbanion reagents to perform the aziridination reaction through insertion to the C=N bond of chiral *N*-*tert*-butanesulfinyl imines (Scheme 1b). The halo-borylsilylcarbanion might combine the nucleophile character for C–C bond formation while preserving the electrophile property to generate the C–N bond through halide displacement. Since chiral *N*-*tert*-butanesulfinamide is undoubtedly one of the most efficient auxiliaries occurring in modern organic synthesis,^4^ our purpose is to study the stereoselective transmission from this chiral auxiliary to multiple new chiral centers through aziridine formation (Scheme 1b). Inspiring works by Hall^5a,5b^ and Cho^5c^ have demonstrated the feasible addition of lithiated 1,1-diborylcarbanions onto chiral *N*-*tert*-butanesulfinyl imines producing β-sulfinimido *gem*-bis­(boronates) with high diastereoselectivity.^5^

**Scheme 1:**
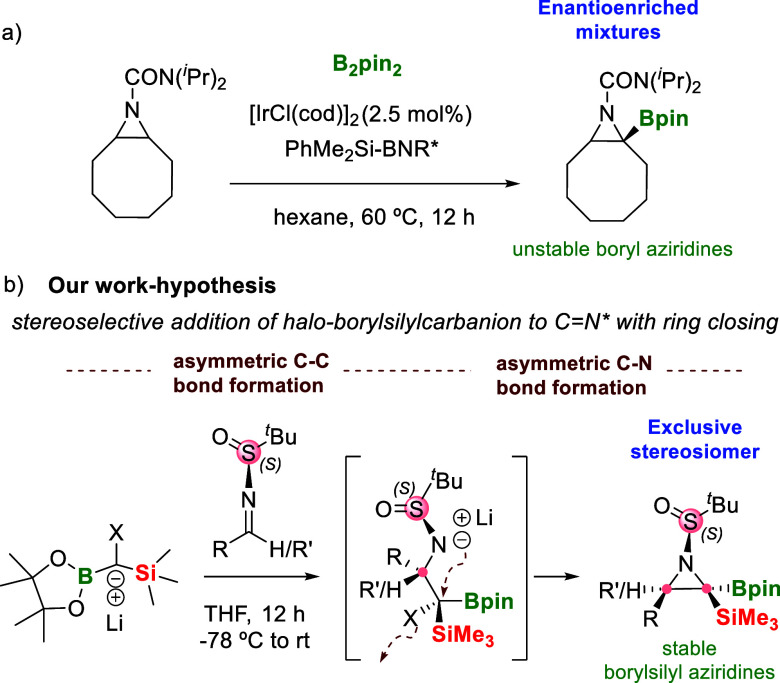
Synthesis of Chiral Boron-Substituted Aziridines: (a) Ref 3 and (b) Our Work Hypothesis

We became persuaded by the nucleophilic character of halo-borylcarbanion reagents that have demonstrated to be valuable synthons for Boron–Wittig reactions,^6^ homologative coupling,^7^ and more recently in cyclopropanation reactions.^8^ The synthesis of halo-diborylmethanes is nowadays well documented,^9^ whereas the preparation of mixed halo-borylsilylmethanes has scarcely been studied involving the reactivity of R_3_Si-Bpin and CH_2_I_2_ in the presence of lithium di-isopropylamide (LDA).^10–12^ Interestingly, photoactivation of the resulting reagent [HC­(I)­(Bpin)­(SiMe_2_Ph)] generates α-bimetalloid radicals that can intercept a series of SOMOphiles (Scheme 2a).^12^ A close related methodology involves the visible light-induced Pd-catalyzed Heck reaction between vinyl arenes/heteroarenes and [HC­(I)­(Bpin)­(SiMe_2_Ph)] to generate allylic boronic esters (Scheme 2b).^11^

**Scheme 2:**
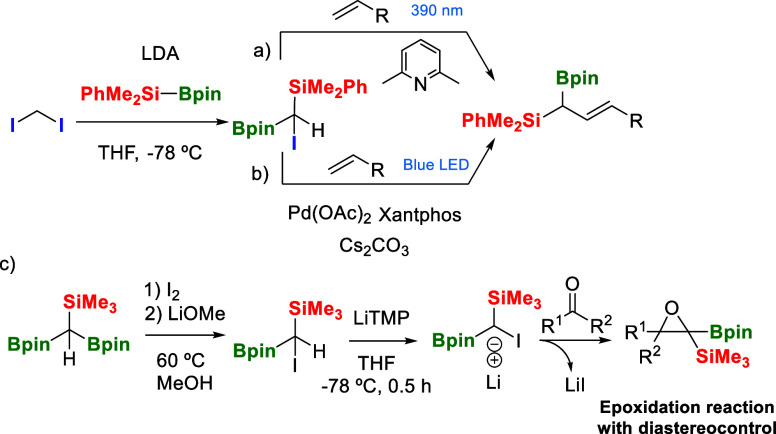
Synthesis of Reagent [HC­(I)­(Bpin)­(SiMe_3_)] and Subsequent Reactivity: (a) Ref 12, (b) ref 11, and (c) ref 13.

Our group has designed a new protocol for the synthesis of the reagent [HC­(I)­(Bpin)­(SiR_3_)] via halogenation/protodeborylation of [HC­(Bpin)_2_(SiR_3_)] (Scheme 2c).^13^ The treatment of [HC­(I)­(Bpin)­(SiR_3_)] with LiTMP or LDA favored the in situ formation of lithiated iodo-borylsilylcarbanion that reacted with ketones to generate epoxides (Scheme 2c). This precedent demonstrates that α-halo B/Si ylide can act as C1 synthon for 1,1,2,2-tetrasubstituted borosilylepoxide synthesis with intrinsic control of diastereoselectivity, opening a new reactive pathway by suppression of Boron-Witting or Peterson olefination pathways.^13^

In that context, we envision here the generation of B/Si ylides from [HC­(X)­(Bpin)­(SiR_3_)] to prove their reactivity toward aziridination pathway with *N*-*tert*-butanesulfinyl imines. This work represents two major challenges: first the outcome of the ylide insertion into C=N bond and second the control of a precise asymmetric induction involving chiral *N*-*tert*-butanesulfinyl imines.

## Results and Discussion

### Reaction Development

To tackle the aziridination study, we selected the model substrate 2-methyl-*N*-(oxetan-3-ylidene) propane-2-sulfinamide (**1**). First, α-iodo borylmethylmethane (**I**) was used as the boron ylide by deprotonation of [HC­(I)­(Bpin)­(Me)] using LDA as the base (Scheme 3a). However, the reaction of the corresponding ylide with imine **1** did not lead to the synthesis of the expected aziridine, covering experimental temperatures from −78 °C to rt. Instead, the starting material remained unaltered, suggesting the low nucleophilic character of the boron ylide.^13^ Similar behavior was observed when α-iodo diborylmethane (**II**) was involved in the azirinidation of **1** (Scheme 3b), despite the fact that this α-halo B/B ylide efficiently promoted cyclopropanation of α,β-unsaturated alkenes, suggesting a precoordination of the carbonyl oxygen with the boryl group before nucleophilic attack of the “boron ylide” to the olefinic unit.^8a^

**Scheme 3:**
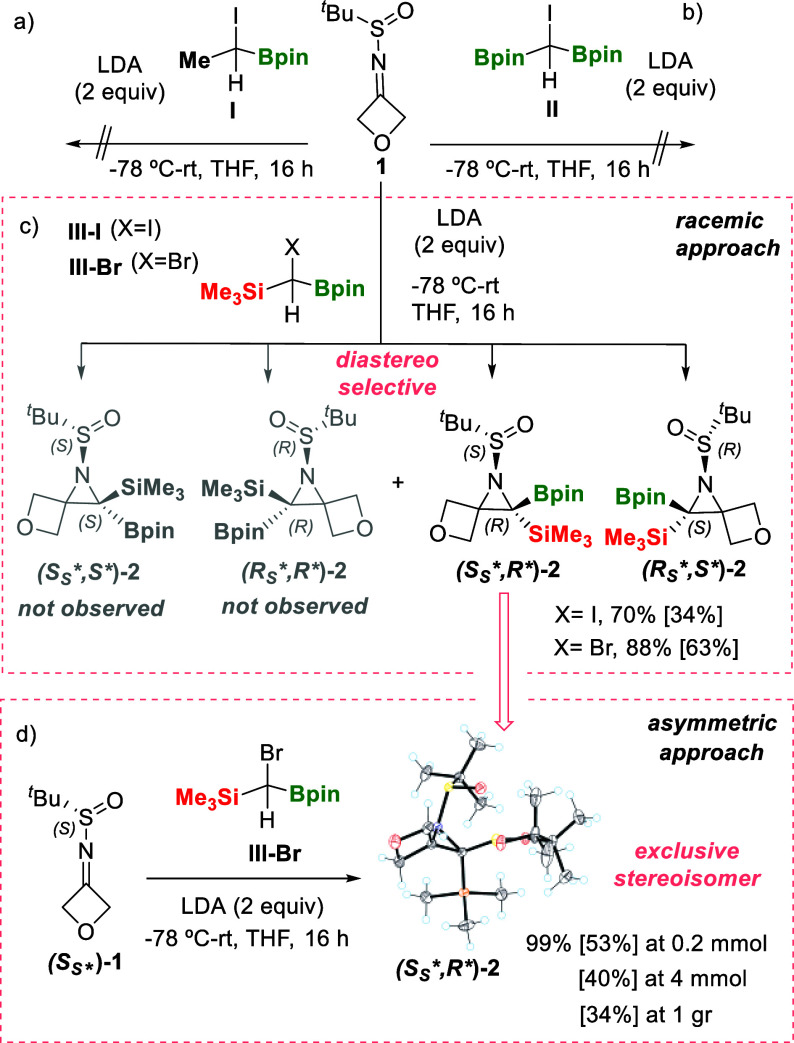
Aziridination of 2-Methyl-*N*-(oxetan-3-ylidene)­propane-2-sulfinamides [1 and (*S*_S_*)-1]: (a) with α-Iodo Borylmethylmethane (I), (b) with α-Iodo Diborylmethane (II), (c) with Halo-borylsilylmethane in Racemic Version, and (d) with Halo-borylsilylmethane in Asymmetric Version

Despite this unsuccessful preliminary study, when iodoborylsilylmethane (**III-I**) reacted with **1**, in the presence of LDA, we came across the isolation of the aziridine *tert*-butylsulfinyl-2-butyl-2-(trimethylsilyl)-5-oxa-1-azaspiro­[2.3] hexane (**2**) (34%), in a complete diastereoselective way (Scheme 3c). The use of bromo-borylsilylmethane (**III-Br**) and LDA allowed the isolation of aziridine **2** in a higher isolated yield, 63% (Scheme 3c). Even though the isolated yields were moderate in comparison with the NMR yields, boron-substituted aziridines are prone to decompose in the presence of trace acid or Lewis acidic silica gel. To minimize decomposition issues, their isolation was performed by purifying the aziridine crude through a small pad of deactivated silica gel, although isolated yields did not significantly improve. The presence of the SiMe_3_ group in the halo-borylsilylcarbanion reagents **III** seems to be responsible for the successful aziridine formation due to the higher nucleophilic character of B/Si ylide.^13^ Encouraged by these results, we conducted the addition of reagent **III-Br** to the chiral *N*-*tert*-butanesulfinyl imine (*S*_*S*_ *)-1 in the presence of LDA. The corresponding spiro-aziridine was exclusively formed as a single stereoisomer in quantitative NMR yield and 53% isolated yield at 0.2 mmol scale. To demonstrate the scalability of the approach, we performed the reaction at 4 mmol and gram-scale syntheses of (*S*_*S*_ *)-1 proving the successful 40 and 34% isolated yields, respectively (Scheme 3d). The absolute configuration of the stereoisomer obtained was assigned by single-crystal X-ray analysis as (*S*_*S*_ *,R*)-2, showing that the favored invertomer corresponds to that with the very bulky *tert*-butanesulfinyl group on the same side as the less sterically hindered Bpin moiety around the aziridine ring (Scheme 3d).^14^ Interestingly, the distance between the O atom in the S=O group and the B atom in the Bpin moiety (2.202, 2.355 Å)^14^ indicates an intramolecular interaction (see SI for characterization details), indicating a plausible directing effect B···O to control the diastereoselective aziridination reaction (Scheme 4). It has to be said that spiro-aziridine compounds containing the oxetane ring are stable to oxidative metabolism and exhibit decreased lipophilicity, conferring an enhanced pharmacokinetic profile for medicinal chemistry purposes.^15^ Additionally, the oxetane ring acts as a stable surrogate for the carbonyl group with similar hydrogen-bond basicity properties but with different electrophilic reactivity.^16^ To the best of our knowledge, the synthesis of spiro-aziridines containing the oxetane ring has been afforded by the reaction of oxetan-3-*tert*-butylsulfinimine with trimethylsulfoxonium iodide and sodium hydride.^17^ Here, we describe an efficient single-step stereoselective insertion of B/Si ylide to the C=N bond of the imine, accessing the spiro-oxetane-aziridine systems including geminal boryl/silyl functional groups for postfunctionalization steps.

**Scheme 4:**
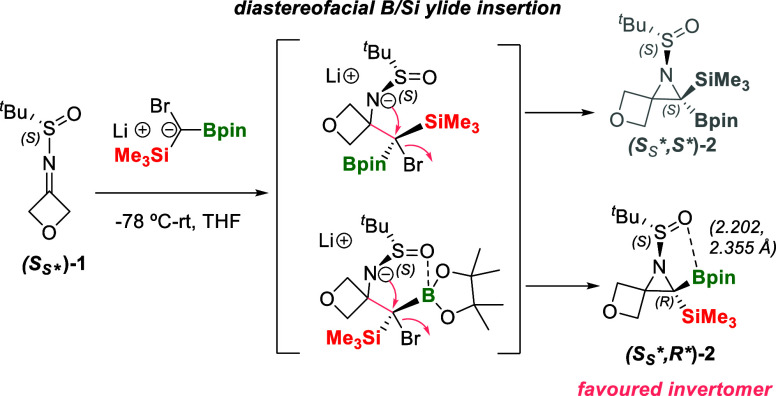
Suggested Directing Effect of B···O to Control the Diastereoselective Aziridination Reaction

With this new aziridination method in our hands, we explored the preparation of the closely related spiro-aziridine compound containing the tetrahydropyran ring since it is a key intermediate for aziridine thailanstatin synthesis.^18^ The addition of **III-Br** to (*S*)-2-methyl-*N*-(tetrahydro-4*H*-pyran-4-ylidene)­propane-2-sulfinamide [(*S*_*S*_ *)-3] in the presence of LDA, allowed the isolation of the spiro-pyrano-aziridine compound (*S*_*S*_ *,R*)-4 in 43% yield as a single stereoisomer (Scheme 5). Similarly, the chiral imine (*S*)-*N*-cyclohexylidene-2-methylpropane-2-sulfinamide [(*S*_*S*_ *)-5] could be efficiently transformed into the spiro-aziridine product (*S*_*S*_ *,R*)-6 in 52% isolated yield, confirming the efficient single-step stereoselective insertion of the B/Si ylide to the chiral cyclic ketimines (Scheme 5). Our single-step methodology to synthesize spiro-aziridine compounds, containing the cyclohexyl ring, in a stereoselective way contrasts with previous reports involving multiple reaction sequences: RMgBr addition to chiral α-chloro *tert*-butanesulfinyl imines to afford diastereoselective β-chloro *N*-sulfinamides, followed by cyclization after separate treatment with bases.^19^

**Scheme 5:**
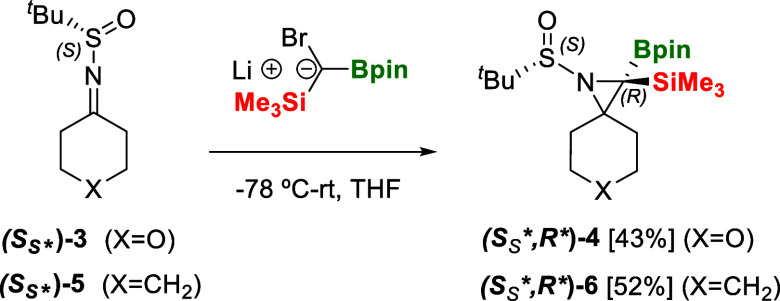
Stereoselective Insertion of B/Si Ylide to *N*-*tert*-Butanesulfinyl Imines (*S*_*S*_ *)-3 and (*S*_*S*_ *)-5, Accessing Enantiomeric Spiro-Aziridine Systems

Since our method uses the chiral *N*-*tert*-butanesulfinyl group to activate the imino function for nucleophilic addition of α-bromo-borylsilylcarbanion in a diastereofacial way, we became encouraged to explore next the B/Si ylide insertion on *N*-*tert*-butanesulfinyl aldimines. This goal involves a double challenging stereoselection since aziridination generates two newly formed stereogenic tetra- and trisubstituted centers. Accordingly, *N*-benzylidene-2-methylpropane-2-sulfinamide (**7**) reacted with **III-Br** in the presence of LDA, and we became delighted to see that only aziridines **(** *S*_*S*_ *, *2* S*, *3* S* **)-8**/(*R*_S_*,*2*R*,*3* R*)-8 were exclusively isolated, despite the fact that more diastereoisomeric aziridines could be formed (Scheme 6a). This diastereofacial insertion of the B/Si ylide was confirmed when the chiral substrate (*S*_*S*_ *)-7 was exclusively converted into (*S*_S_*,*2*S*,*3* S*)-8 in quantitative NMR yield and 54% isolated yield (Scheme 6b). The absolute configuration of (*S*_S_*,*2*S*,*3* S*)-8 was assigned by single-crystal X-ray analysis indicating that in this favored invertomer, the two newly formed stereogenic centers placed the Bpin and Ph groups along the same face of the aziridine ring, whereas the bulky *tert*-butanesulfinyl group appears *cis* to the SiMe_3_ group (Scheme 6b).^14^ This stereoselective distribution contrasts with that observed for spiro-aziridine (*S*_*S*_ *,R*)-2 where the Bpin moiety was placed *cis* to the bulky *tert*-butanesulfinyl group. It seems that in the case of aldimine involving the phenyl moiety, the steric factors prevail to control the diastereoselective aziridination reaction in front of directing B···O interactions. Our group had observed a similar preferred stereoisomer in the cyclopropanation of 1,1-diborylalkenes with (trimethylsilyl) diazomethane to generate polyfunctionalized B, B, Si-cyclopropanes.^20^

**Scheme 6:**
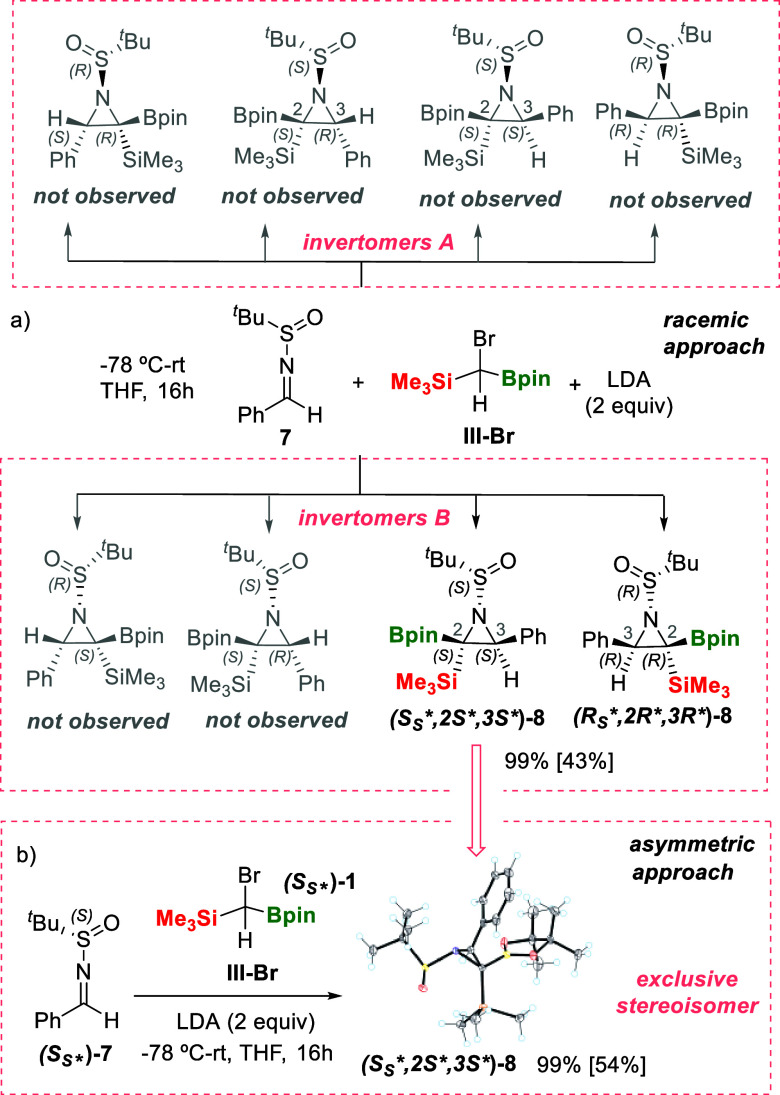
Aziridination of *N*-Benzylidene-2-methylpropane-2-sulfinamide 7 and (*S_S_* *)-7 through Insertion of B/Si Ylide: (a) with Bromo-borylsilylmethane in Racemic Version and (b) with Bromo-borylsilylmethane in Asymmetric Version

Since the preferred diastereofacial B/Si ylide insertion on *N*-*tert*-butanesulfinyl aldimine (*S*_*S*_ *)-7 takes place with stereoselective control, we planned to construct a collection of polyfunctionalized chiral aziridines modifying the aryl moiety. Substrates (*S*_*S*_ *)-9 and (*S*_*S*_ *)-11, with Cl- and Me- substituents in the *para*-position of the phenyl group, reacted with **III-Br** in the presence of LDA, producing aziridines (*S*_S_*,*2*S*,*3* S*)-10 and (*S*_S_*,*2*S*,*3* S*)-12 in slightly higher isolated yields when the electron withdrawing substituents are involved (Scheme 7). In a similar pathway, (*S*)-*N*-(2-fluorobenzylidene)-2-methylpropane-2-sulfinamide ((*S*_*S*_ *)-13) reacted with the α-bromo-borylsilylcarbanion in a diastereofacial way, generating the chiral aziridine (*S*_S_*,*2*S*,*3* S*)-14 in 51% isolated yield, demonstrating the compatibility of the method with the *ortho-*fluorinated substituents (Scheme 7). However, the imine substrate (*S*_*S*_ *)-15 with the CF_3_ group in the *ortho* position of the phenyl group was converted into the expected chiral aziridine (*S*_S_*,*2*S*,*3* S*)-16 in only 25% isolated yield (Scheme 7) as a consequence of a lower diastereomeric ratio (d.r. = 87:17). The complementary stereoisomeric aziridine (*S*_S_*,*2*S*,*3* R*)-16 could be isolated in 5%. This observation might be correlated with an intramolecular affinity of the fluorides in CF_3_ with the silyl group,^21^ confirming previous observations when α-B/Si carbanion interacts with aldehydes containing a CF_3_ group in the *ortho* position of the phenyl substituent.^22^

**Scheme 7:**
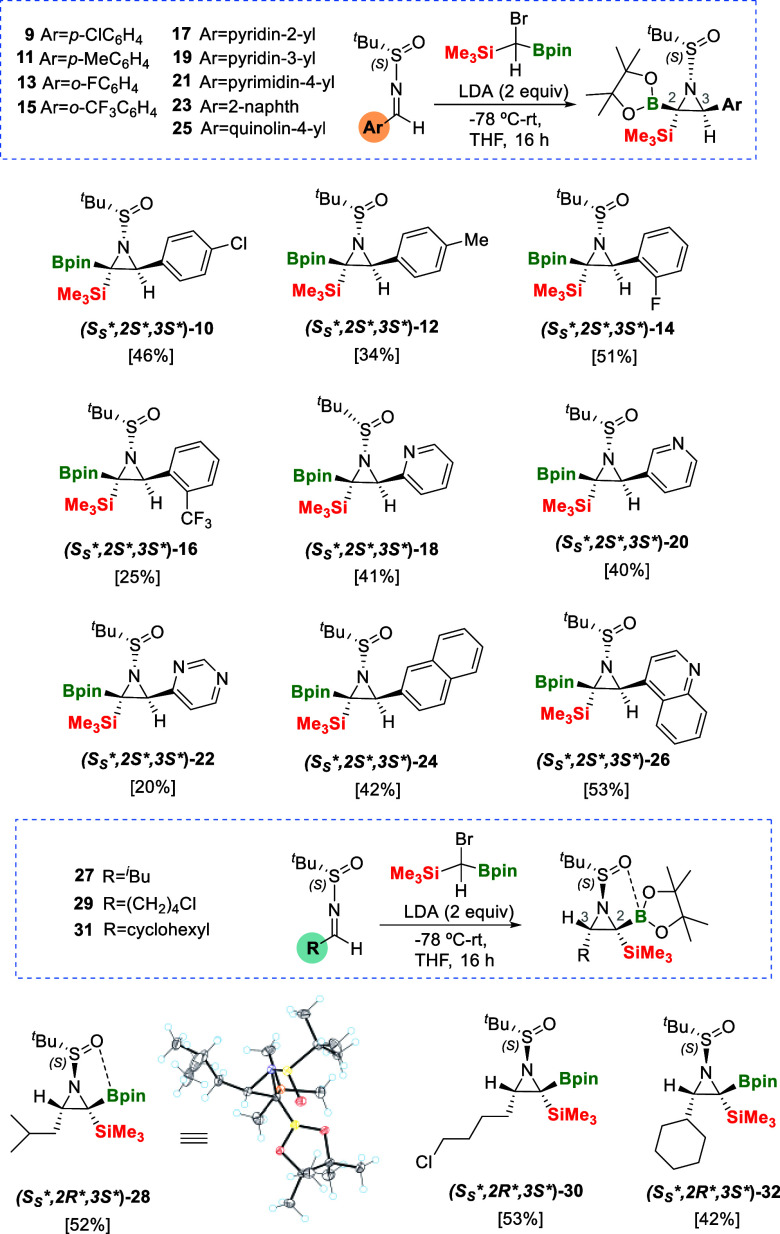
Substrate Scope on Aziridination of Chiral *N*-*tert*-Butanesulfinyl Aldimines Containing Aryl or Alkyl Groups through Complementary Stereoselective Insertion of B/Si Ylide

We were very pleased to see that the B/Si ylide insertion on the chiral *N*-*tert*-butanesulfinyl aldimines, (*S*_*S*_ *)-17 and (*S*_*S*_ *)-19 containing the pyridin-2-yl and pyridin-3-yl units, respectively, formed the chiral aziridines (*S*_S_*,*2*S*,*3* S*)-18 and (*S*_S_*,*2*S*,*3* S*)-20 in high diastereoselection and similar isolated yields (Scheme 7). In the case of the reaction of (*S*)-2-methyl-*N*-(pyrimidin-4-ylmethylene)­propane-2-sulfinamide with **III-Br**, the isolation of chiral aziridine (*S*_S_*,*2*S*,*3* S*)-22 was achieved in lower yield, despite the fact that NEt_3_-neutralized flash chromatography was used for purification. Bulkier 2-naphthyl and quinolin-4-yl substituted substrates also reacted successfully to deliver stereocontrolled aziridines (*S*_S_*,*2*S*,*3* S*)-24 and (*S*_*S*_ *,*2*S*,*3* S*)-26 (Scheme 7). The preferred diastereofacial B/Si ylide insertion on chiral (*S*)-*N*-*tert*-butanesulfinyl aldimines, containing aryl groups, established that the Bpin moiety is placed *cis* to the aryl substituent but *trans* to the *N*-*tert*-butanesulfinyl group, discarding any plausible B···O=S interaction, as illustrated in the single-crystal X-ray analysis for (*S*_S_*,*2*S*,*3* S*)-8 (Scheme 6b).^14^ However, when the chiral (*S*)-*N*-*tert*-butanesulfinyl aldimines contained alkyl groups, the aziridination protocol generated a complementary diastereofacial B/Si ylide insertion. When (*S*)-2-methyl-*N*-(3-methylbutylidene)­propane-2-sulfinamide (*S*_*S*_ *)-27 reacted with α-bromo-borylsilylcarbanion, the resulting aziridine **28** was isolated in 52% yield (Scheme 7). The absolute configuration of the resulting stereoisomer could be assigned by single-crystal X-ray analysis as (*S*_*S*_ *,2R*,3S*)-28 showing that the Bpin moiety is facing the less sterically hindered H atom, forcing the trimethylsilyl group and the alkyl group to be *cis* (Scheme 7). This favored invertomer also pointed out that the *tert*-butanesulfinyl group is placed on the same side as the less sterically hindered Bpin moiety showing an intramolecular interaction, as illustrated by the short distance between the O atom in the S=O group and the B atom in the Bpin moiety (2.27, 2.47 Å; see SI for characterization details). This suggests a plausible directing effect of O toward the empty *p* orbital of B to control the diastereoselective aziridination reaction (Scheme 7), in contrast to the observed lack of directing effect when aryl groups are involved. The new preferred diastereofacial B/Si ylide insertion is even noteworthy when bulkier alkyl groups are present in *N*-*tert*-butanesulfinyl aldimines, such as 4-chlorobutyl or cyclohexyl groups, contributing to the synthesis of diastereoselective chiral aziridines (*S*_*S*_ *,2R*,3S*)-30 and (*S*_*S*_ *,2R*,3S*)-32 respectively, (Scheme 7).

We next studied the synthesis of aziridines with chirality along the alkyl moiety to increase the number of stereocenters in the final product. The B/Si ylide insertion into chiral (*S*)-*N*-((*S*)-3,7-dimethyloct-6-en-1-ylidene)-2-methylpropane-2-sulfinamide ((*S*_S_*,*S*)-33 proved to be efficient in generating the aziridine (*S*_*S*_ *,2R*,3S*,*S*)-34 that contains four chiral centers (Scheme 8a). The aziridination of (*S*)-2-methyl-*N*-(3-phenylbutylidene)­propane-2-sulfinamide (*S*s*,*rac*)-35 with Li­[CBr­(Bpin)­(SiMe_3_)] allowed the isolation of both diastereoisomers (*S*_*S*_ *,2R*,3S*,*S*)-36 and (*S*_*S*_ *,2R*,3S*,*R*)-36 in similar isolated yields, demonstrating the diastereodivergent aziridination procedure (Scheme 8b). Intriguingly, when (*S*)-2-methyl-*N*-(2-methylbutylidene)­propane-2-sulfinamide (*S*s*,*rac*)-37 reacted with α-bromo-borylsilylcarbanion, the aziridination process went through the formation of a mixture of both diastereoisomers (*S*_*S*_ *,2R*,3S*,*S*)-38/(*S*_*S*_ *,2R*,3S*,*R*)-38 in d.r. = 6/4 postulating a plausible stereochemical discrimination procedure (Scheme 8c). The most remarkable observation came through the exclusive formation of the stereoisomer (*S*_*S*_ *,2R*,3S*,*R*)-40 when the B/Si ylide was inserted in (*S*)-2-methyl-*N*-(2-phenylpropylidene)­propane-2-sulfinamide (Ss*,*rac*)-39, suggesting not only the preferred diastereofacial B/Si ylide insertion but also a complete stereochemical discrimination on the racemic center at −CH­(Me)­(Ph) (Scheme 8d). The absolute configuration of (*S*_*S*_ *,2R*,3S*,*R*)-40 was assigned by single-crystal X-ray analysis, establishing that the Bpin moiety is placed *cis* to the *N*-*tert*-butanesulfinyl group and to H (Scheme 8d),^14^ pointing out the intramolecular B···O=S interaction with short distance between the O atom and the B atom (2.46, 2.51 Å), (see SI for characterization details). This complete stereochemical discrimination suggests that the pair interaction energy of both molecules (*S*_*S*_ *,*R*)-39 and (*S*_*S*_ *,*S*)-39 is different when they react with the B/Si ylide, referring to it as a chiral discrimination process. The exclusively formation of (*S*_*S*_ *,2R*,3S*,*R*)-40 suggests that the aziridination proceeds efficiently with the ***R***-enantiomer of the C­(Me)­(Ph) group based on the different spatial arrangements of Me and Ph substituents. This difference can manifest as varying the reaction rates through binding affinities, as was also observed in the aziridination of *N*-*tert*-butanesulfinyl aldimine (*S*s*,*rac*)-41, exclusively generating the stereoisomer (*S*_*S*_ *,2R*,3S*,*R*)-42 (Scheme 8e). Despite the fact that the isolated yield is low, no other isomers were observed.

**Scheme 8:**
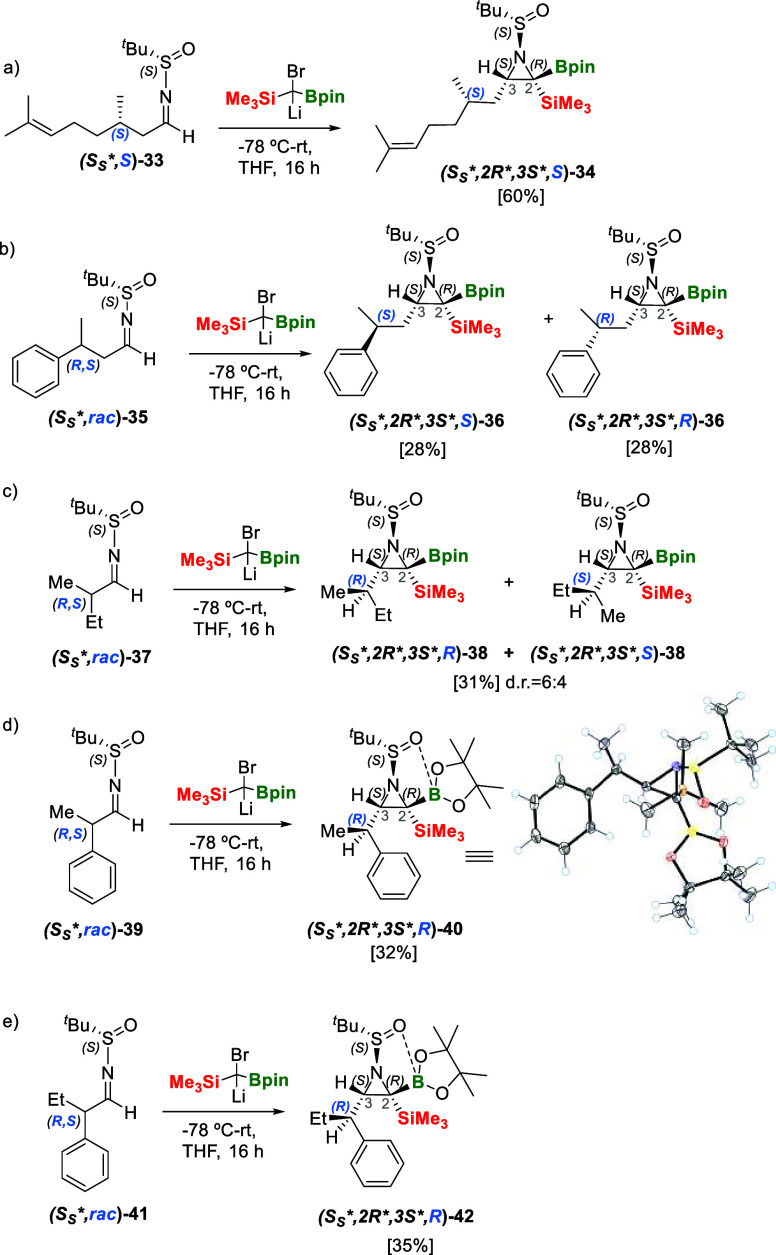
Aziridination of Chiral *N*-*tert*-Butanesulfinyl Aldimines, Aimed to Construct Vicinal Stereogenic Carbon Centers: (a) with (*S*_S_*,*S*)-33, (b) with (*S*s*,*rac*)-35, (c) with (*S*s*,*rac*)-37, (d) with (*S*s*,*rac*)-39, and (e) with (*S*s*,*rac*)-41

This new protocol allowed us to construct chiral aziridines bearing three vicinal stereogenic carbon centers with complete stereocontrol. To the best of our knowledge, chiral aziridines bearing two vicinal tetrasubstituted and acyclic quaternary stereogenic carbon centers could be prepared through a copper­(I)-catalyzed decarboxylative Mannich reaction between α,α-disubstituted cyanoacetic acids and various 2H-azirines.^23^

Taking advantage of the stereoselective formation of the aziridines prepared in this work, we explored next the transformation of the C–B bond. Treatment of **28** with vinylmagnesium bromide followed by the Zweifel olefination^24^ enabled the instalment of a vinyl group in α position to the SiMe_3_ moiety in product **43** (Scheme 9). Next, we explored the oxidation of the sulfinamide group in aziridine **28** with *m*-chloroperbenzoic acid to produce the *tert*-butylsulfonamide derivative **44** in high yields (Scheme 9).^25^ Interestingly, the *tert*-butylsulfonyl group had a marked influence on the reaction of **44** with vinylmagnesium bromide since the resulting product **45** did not replace the C–B bond by the C-vinyl group; instead, a regioselective nucleophilic attack of the vinyl group with concomitant ring opening took place (Scheme 9). Complementarily, when we studied the reactivity of compound **44** with LiCH_2_Br (formed from CH_2_Br_2_ and nBuLi), we observed a regioselective ring opening sequence via nucleophilic attack at the carbon α to the silyl group, followed by the Br-Bpin elimination pathway, generating the 1,1-disubstituted alkene **46** (Scheme 9). Eventually, the C-SiMe_3_ motif in **44** was treated with TBAF, and protodesilylation sequence occurred to form product **47** in 45% yield, although the instability of the α-boryl aziridine diminished the isolation of the product in higher yield (Scheme 9).

**Scheme 9:**
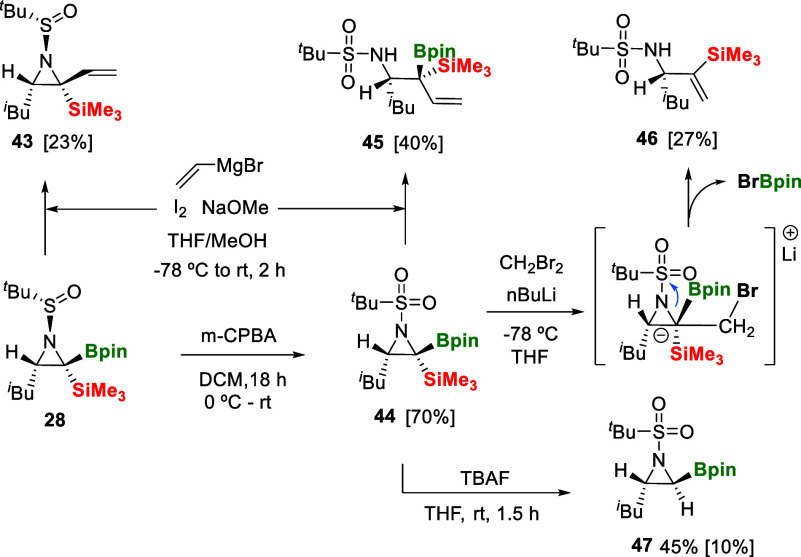
Functionalization of α-Borylsilyl Aziridines

Next, we applied the protodeborylation protocol with KOH/H_2_O (3 equiv) at 90 °C on the aziridine (*S*_*S*_ *,R*)-28 and observed the straightforward formation of the desired aziridine (*S*_*S*_ *,R*)-48 with complete retention of the configuration (Scheme 10). The nucleophilic attack of ^–^OH is chemoselective on the empty orbital of the B atom, discarding any direct attack on the aziridine ring that would have evolved toward the ring opening pathway. Similarly, the aziridines (*S*_S_*,*2*S*,*3* S*)-8 smoothly evolved toward the protodeborylated aziridine (*S*_S_*,*2*S*,*3* S*)-49 with comparable isolated yields (Scheme 9). It is worthy to mention that the stereoselective aziridination procedure followed by the protodeborylation allowed the isolation of chiral C-silylated aziridines containing two vicinal trisubstituted stereogenic centers. In addition, the complementary stereochemical distribution is also noteworthy as in (*S*_S_*,*2*S*,*3* S*)-49, the SiMe_3_ moiety is *trans* to the aryl group, whereas in (*S*_*S*_ *,2R*,3S*)-48, the SiMe_3_ moiety is *cis* to the alkyl group. The resulting complementary conformation is due to the effective aryl/alkyl influence of the substituents along the stereoselective aziridination process. When protodeborylation was conducted on the chiral aziridine (*S*_*S*_ *,2R*,3S*,*R*)-40, we were pleased to prove that the C–B transformation gave access to the chiral C-silylated aziridine (*S*_*S*_ *,2R*,3S*,*R*)-50 which has created three vicinal trisubstituted stereogenic centers from (*S*)-2-methyl-*N*-(2-phenylpropylidene)­propane-2-sulfinamide (Ss*,*rac*)-39 (Scheme 10). Access to chiral C-silylated aziridines has been elegantly developed by Oestreich and co-workers through copper-catalyzed silylation of 3-substituted 2*H*-azirines using a silyl boronic ester reagent, although only one stereogenic center is reported for the new chiral aziridines.^26^ Eventually, we also explored the protodeborylation of (*S*_*S*_ *,R*)-2 with the isolation of the spiro compound (*S*_*S*_ *,R*)-51 in 60% isolated yield, as well as the formation of 2-deuteroaziridine **(** *S*_*S*_ *,R* **)-52** using D_2_O (Scheme 10). The reaction afforded the deuterated product in 52% isolated yield with > 99% D-incorporation, contributing to fill the gap on the chiral 2-deuteroaziridine synthesis.^27^

**Scheme 10:**
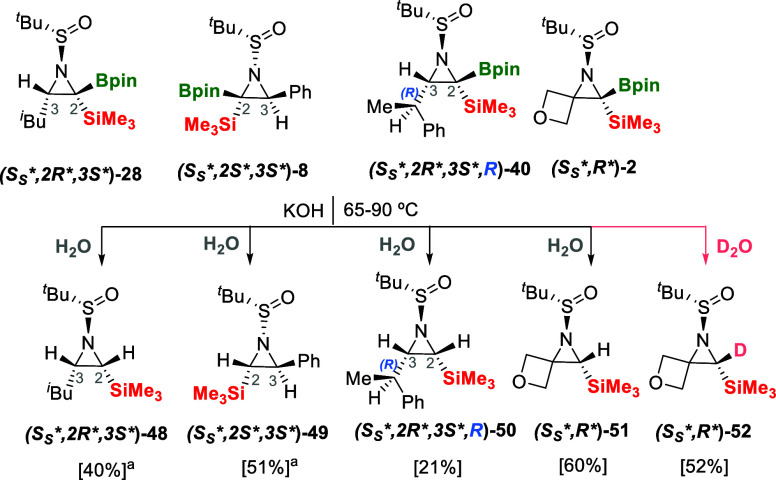
Protodeborylation and Deuterodeborylation of Chiral Aziridines with Multiple Chiral Centers[Fn sch10-fn1]

a
*T* = 90 °C.

## Conclusions

In summary, we have developed an efficient and general method for the aziridination of *N*-*tert*-butanesulfinyl imines with halo-borylsilylcarbanion reagents, resulting in a complete diastereofacial control. This represents the first use of the Li­[C­(Br)­(Bpin)­(SiR_3_)] reagents for aziridination reaction and is postulated as a single-step access to tetrasubstituted carbon centers with precise stereochemical control. The chiral *N*-*tert*-butanesulfinyl imines featured an asymmetric C–C bond followed by concomitant intramolecular asymmetric C–N bond formation delivering exclusive stereoisomeric α,α-B,Si-disubstituted aziridines, which resulted in being very stable. The procedure occurred with stereofacial control allowing the generation of multiple adjacent chiral centers. Stereochemical discrimination has also been observed in *N*-*tert*-butanesulfinyl alkyl aldimines. Late-stage diversification on the C–B, C–Si, and sulfinyl groups has been explored, together with the proto-and deuterodeborylation giving access to *C*-silylated aziridines with two and three vicinal new stereocenters around the aziridine ring. The method leads stereocontrolled single-step access to medicinally relevant spiro-aziridine scaffolds.

## Methods

### General Procedure for Aziridination of *N*-*tert*-Butanesulfinyl Ketimines and Aldimines

In an oven-dried Schlenk tube, charged with a magnetic stir bar, a solution of α-bromo-borylsilylmethane (0.2 mmol) in 2 mL of anhydrous THF was added. Next, the solution was cooled down to −78 °C in a dry ice bath with acetone, and LDA (0.4 mL, 2 equiv) was added to the reaction mixture. After stirring the solution at −78 °C for 15 min, the corresponding *N*-*tert-*butanesulfinyl ketimine or aldimine (0.2 mmol, 1 equiv) dissolved in 0.5 mL of THF was added to the reaction mixture, and it was allowed to stir for 16 h at rt. Upon the completion of the reaction time, the solvents were evaporated under vacuum, and the reaction crude was purified with flash column chromatography to afford the aziridine product.

### Synthesis of Product (*S*_*S*_ *,R*)-2

In an oven-dried Schlenk tube, charged with a magnetic stir bar, a solution of (iodo­(4,4,5,5-tetramethyl-1,3,2-dioxaborolan-2-yl)­methyl)­trimethylsilane (68.02 mg, 0.2 mmol) in 2 mL of anhydrous THF was added. Next, the solution was cooled down to −78 °C in a dry ice bath with acetone, and LDA (0.4 mL, 2 equiv) was added to the reaction mixture. After stirring the solution at −78 °C for 15 min, *N*-*tert*-butanesulfinyl ketimine **(*S***_***S***_ *)-1 (35 mg, 0.2 mmol, 1 equiv), dissolved in 0.5 mL of THF, was added to the reaction mixture, and it was allowed to stir for 16 h at rt. Upon the completion of the reaction time, the solvents were evaporated under vacuum, and the reaction crude was purified using silica gel chromatographic techniques to afford the aziridine product **(*S***_***S***_ *,R*)-2 as a white solid (26 mg, 34%).

### Synthesis of Product (*S*_S_*,2S*,3S*)-8

In an oven-dried Schlenk tube, charged with a magnetic stir bar, a solution of (iodo­(4,4,5,5-tetramethyl-1,3,2-dioxaborolan-2-yl)­methyl)­trimethylsilane (58.6 mg, 0.2 mmol, 1 equiv) in 2 mL of anhydrous THF was added. Next, the solution was cooled down to −78 °C in a dry ice bath with acetone, and LDA (0.4 mL, 2 equiv) was added to the reaction mixture. After stirring the solution at −78 °C for 15 min, *N*-tertbutanesulfinyl aldimine **(*S***_***S***_ *)-7 (41.8 mg, 0.2 mmol, 1 equiv) dissolved in 0.5 mL of THF, was added to the reaction mixture, and it was allowed to stir for 16 h at rt. Upon the completion of the reaction time, the solvents were evaporated under vacuum, and the reaction crude was purified using silica gel chromatographic techniques to afford the aziridine product (*S*_S_*,2S*,3S*)-8 as a white yellowish solid (45 mg, 54%).

### Synthesis of Product (*S*_*S*_ *,R*)-51

In an oven-dried Schlenk tube, charged with a magnetic stir bar, were introduced KOH (3 equiv, 33.66 mg) and aziridine (*S*_*S*_ *,R*)-2 (0.2 mmol, 77,48 mg). After that, 2 mL of THF and 0.2 mL of water were added, and the reaction mixture was allowed to stir for 16 h at 65 °C. Upon the completion of the reaction time, the reaction was allowed to warm to rt, the solvents were evaporated under vacuum, and the reaction crude was purified using silica gel chromatographic techniques to afford the product (*S*_*S*_ *,R*)-51 as a solid (32 mg, 60%).

### Synthesis of Product (*S*_*S*_ *,R*)-52

In an oven-dried Schlenk tube, charged with a magnetic stir bar, was introduced KOH (3 equiv, 33.66 mg) and aziridine (*S*_*S*_ *,R*)-2 (0.1 mmol, 38,74 mg). After that, 1 mL of THF and 0.1 mL of D_2_O were added, and the reaction mixture was allowed to stir for 16 h at 65 °C. Upon the completion of the reaction time, the reaction was allowed to warm to rt, the solvents were evaporated under vacuum, and the reaction crude was purified using silica gel chromatographic techniques to afford the product (*S*_*S*_ *,R*)-52 as a yellowish oil (28 mg, 52%).

The diastereo- and enantioselectivities were determined by ^1^H NMR spectroscopy and HPLC analysis, comparing the aziridines containing both *rac*- and (*S*)-sulfinyl groups.

## Supplementary Material



## Notes

General and specific procedures for the synthesis of substrates, reagents, and products, as well as full characterization through spectral data, HPLC analysis, and X-ray diffraction (PDF)
