# Supplementary material for: Stereofacial Assembly of Engineered Multichiral Aziridines via B/Si Ylide Insertion
Source: JACS Au. 2025 Nov 25;5(12):6315–23. doi: 10.1021/jacsau.5c01306 (PMC12728649; doi:10.1021/jacsau.5c01306)
Supplement: Supplementary file 1 [file au5c01306_si_001.pdf]

Supporting Information

Stereofacial assembly of engineered multi-chiral aziridines via B/Si ylide insertion

Mireia Pujol,[a+] Luis Tarifa,[a+] Anika Tarasewicz,[b] María Méndez,[b]* Elena Fernández[a]*

[a]Faculty of Chemistry, University Rovira i Virgili, 43007 Tarragona, Spain. [b]Sanofi R&D, Integrated Drug Discovery, Industriepark Höchst, Bldg. G838, 65926 Frankfurt am Main, Germany.
E-mail: Maria.MendezPerez@sanofi.com, mariaelena.fernandez@urv.cat
[+] These authors contributed equally to this work

Contents

- General information S2
- Specific procedure for the synthesis of 2-(1-iodoethyl)-4,4,5,5-tetramethyl-1,3,2-dioxaborolane (**I**) S3
- Specific procedure for the synthesis of 2,2'-(iodomethylene)bis(4,4,5,5-tetramethyl-1,3,2-dioxaborolane) (**II**) S3
- General procedure for the synthesis of α-monohalo diborylsilylmethane S3
- General procedure for the synthesis of α-monohalo borylsilylmethane (**III**) S4
- General procedure A for the synthesis of *N*-*tert*-butanesulfinyl ketimines S4
- General procedure B for the synthesis of *N*-*tert*- butanesulfinylsulfinyl aldimines S6
- General procedure for aziridination of *N*-*tert*-butanesulfinyl ketimines and aldimines S7
- Specific example for reaction of **28** with vinyl magnesium bromide S8
- Specific example for oxidation of **28** with *m*-chloroperbenzoic acid S9
- Specific example for reaction of **28** with CH_2Br_2/nBuLi S9
- Specific example for protodesilylation of **28** S9
- General procedure for protodeborylation S10
- Spectral data for diborylsilylmethane, α-monohalo diborylsilylmethane and α-monohalo borylsilylmethane (**III**) reagents S12
- Spectral data for *N*-*tert*-butanesulfinyl ketimines and aldimines S13
- Spectral data for chiral aziridines S22
- Spectral data for diversified aziridines S35
- ^{1}H, ^{13}C, ^{11}B NMR spectra for diborylsilymethane, α-monohalo diborylsilylmethanes and α-monohalo borylsilylmethane reagents S41
- ^{1}H, ^{13}C, ^{19}F NMR spectra for *N*-*tert*-butanesulfinyl ketimines and aldimines S49
- ^{1}H, ^{13}C, ^{11}B, ^{19}F NMR spectra for chiral aziridines S71
- ^{1}H, ^{13}C, ^{11}B NMR spectra for diversified aziridines S110
- HPLC analysis of chiral aziridines S123
- X-Ray Diffraction S147
- References S206

General information

Solvents and reagents: Solvents and reagents were obtained from commercial suppliers and dried and/or purified (if needed) by standard procedures. Diboron reagents were purchased from Ally Chem and used without further purification. All reactions were conducted in oven and flame-dried glassware under an inert atmosphere of argon, using Schlenk-type techniques. *Flash chromatography* was performed on standard silica gel (Merck Kieselgel 60 F254 400-630 mesh). *Thin layer chromatography* was performed on Merck Kieselgel 60 F254 which was developed using standard visualizing agents: UV fluorescence (254 and 366 nm) or potassium permanganate/Δ. *NMR spectra* were recorded at a Varian Goku 400 or a Varian Mercury 400 spectrometer. ^{1}H NMR and $^{13}C\{^{1}H\}$ NMR chemical shifts (δ) are reported in ppm with the solvent resonance as the internal standard ($CDCl_3$: 7.26 ppm (^{1}H) and $CDCl_3$: 77.16 ppm (^{13}C)). $^{11}B\{^{1}H\}$ NMR chemical shifts (δ) are reported in ppm relative to $(CH_3)_2O\cdots BF_3$. Data are reported as follows: chemical shift, multiplicity (s = singlet, d = doublet, t = triplet, q = quartet, hept = heptuplet, br = broad, m = multiplet), coupling constants (Hz) and integration. *High resolution mass spectra (HRMS)* were recorded using a 6210 Time of Flight (TOF) mass spectrometer from Agilent Technologies (Waldbronn, Germany) with an ESI interface and it was performed at the Servei de Recursos Científics i Tècnics (Universitat Rovira i Virgili, Tarragona) or using a BIOTOF II Time of Flight (TOF) mass spectrometer from Bruker with an APCI interface or EI interface and it was performed at the Unidad de Espectrometría de Masas e Proteómica (Universidade de Santiago de Compostela, Santiago de Compostela). GC-MS analyses were performed on a HP6890 gas chromatograph and an Agilent Technologies 5973 Mass selective detector (Waldbronn, Germany) equipped with an achiral capillary column HP-5 (30m, 0.25mm i. d., 0.25µm thickness) using He as the carrier gas. Chiral HPLC was performed using Diacel or Chiralcel columns (4.6 x 250 mm) fitted with the respective guards (4 x 10 mm) and monitored by DAD (Diode Array Detector). M.p.: Melting points were measured in a Büchi B-540 apparatus in open capillary tubes and are uncorrected. $[\alpha]_D^{20}$: Optical rotations were measured at 20 ºC on a Jasco P-2000 polarimeter with sodium lamp at 589 nm and a path of length of 1 dm. Solvent and concentration are specified in each case. Full sphere single crystal data collection where performed at 100 K on a Bruker Kappa Apex II DUO diffractometer equiped with a Cryostream 700 plus low temperature device.

Specific procedure for the synthesis of 2-(1-iodoethyl)-4,4,5,5-tetramethyl-1,3,2-dioxaborolane (I)

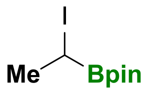

I

To a solution of 2-(1-chloroethyl)-4,4,5,5-tetramethyl-1,3,2-dioxaborolane (952.4 mg, 5 mmol, 1 equiv.) in acetone (0.3 M), NaI (2.3 g, 15 mmol, 3 equiv) was added. The resulting suspension was stirred at ambient temperature overnight. Solvent was removed under reduced pressure and the crude residue was diluted with CH_2Cl_2 (2 mL) and filtered. After concentration under reduced pressure, the residue was dissolved in EtOAc and passed through a silica plug. After concentration under reduced pressure, the crude residue was purified via flash column chromatography to afford the desired product **I** (917 mg, 65%).Spectral data are in agreement with the reported one.[1]

Specific procedure for the synthesis of 2,2'-(iodomethylene)bis(4,4,5,5-tetramethyl-1,3,2-dioxaborolane) (II)

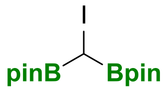

II

An oven-dried 100 mL round-bottom Schlenk tube was charged with chloro *gem*-diboronate (302 mg, 1 mmol, 1 equiv) and sodium iodide (750 mg, 5 mmol, 5 equiv) in acetone (10 mL). The reaction mixture was stirred at room temperature overnight. The resulting suspension was filtered, and the solvent was evaporated under reduced pressure. The residue was diluted with water and extracted with hexane. The organic layer was dried over Na_2SO_4, filtered and concentrated, yielding the desired product **II** (178 mg, 45%). Spectral data are in agreement with the reported one.[2]

General procedure for the synthesis of α-monohalo diborylsilylmethane

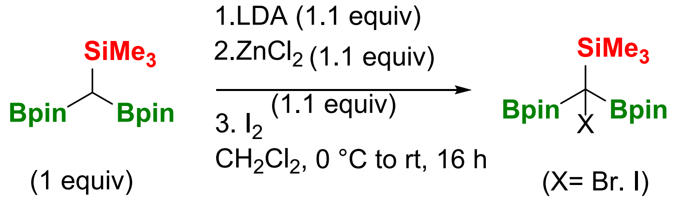

An oven-dried Schlenk tube covered with aluminium foil and equipped with a magnetic stirring bar was charged with I_2 (1.1 mmol, 1.1 equiv) or NBS (2 mmol, 2 equiv) and dry CH_2Cl_2 (2 mL). In another oven-dried Schlenk tube equipped with a magnetic stirring bar, was added the corresponding *gem*-diborylsilylmethane (1 mmol, 1 equiv). The Schlenk tube was filled with Ar and dry THF (1 mL) and LDA 1M in THF/hexane (1.1 mmol, 1.1 equiv) were added and stirred at 0 °C for 1 hour. Then, $ZnCl_2$ (1.1 mmol, 1.1 equiv) was added to the reaction mixture under Ar at 0 °C and was stirred for 10 min at 0 °C and warmed to room temperature over 2 hours. Afterwards, this solution was transferred

dropwise *via* cannula to the I_2 or NBS in CH_2Cl_2 at 0 °C and the resulting reaction mixture was stirred for 16 h at room temperature. After the reaction time, the reaction was quenched with 10 mL of sodium thiosulfate (saturated solution) and extracted with ethyl acetate (3 x 25 mL). The organic layers were collected and washed with 20 mL of water. The organic phase was then dried with anhydrous $MgSO_4$ and filtered, and the solvents were evaporated. The reaction crude was purified using silica gel chromatographic techniques to afford the desired product.

General procedure for the synthesis of α-monohalo borylsilylmethane (III)

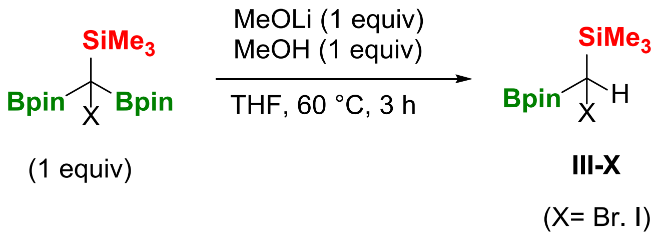

In an oven-dried Schlenk tube equipped with a magnetic stirring bar, were added the corresponding α-monohalo diborylsilylmethane (2 mmol, 1 equiv) and lithium methoxide (2 mmol, 1 equiv). The flask was purged with Ar, then 6 mL of anhydrous THF and MeOH (2 mmol, 1 equiv) were added, and the reaction mixture was stirred at 60 °C for 3 h. After the reaction time, the solvents were gently evaporated using a rotatory evaporator and the reaction crude was purified using silica gel chromatographic techniques to afford the desired product.

General procedure A for the synthesis of *N*-*tert*-butanesulfinyl ketimines[3]

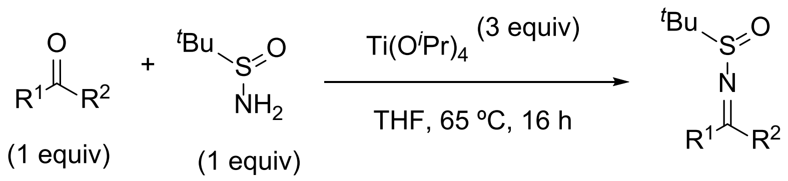

To a solution of $Ti(O^iPr)_4$ (6 mmol, 3 equiv, 1.78 mL) in anhydrous THF (7.6 mL), the corresponding ketone was added (2 mmol, 1 equiv) and the mixture was stirred at room temperature for 15 min. This was followed by the slow addition of *tert*-butylsulfinamide (2 mmol, 1 equiv, 242,4 mg), and the reaction mixture was heated to 65 °C. When the reaction reached completion, it was allowed to cool at room temperature. The reaction was then quenched with brine (10 mL) and the resulting slurry solution was filtered through a pad of Celite, and the filter cake was washed with ethyl acetate (5 mL). The filtrate was then transferred to a separating funnel where the organic layer was washed with brine (5 mL). The aqueous layer was extracted once with ethyl acetate (15 mL), and

the combined organic extracts were dried ($MgSO_4$), filtered, and concentrated under vacuum. Flash column chromatography afforded the desired *N*-*tert*-butanesulfinyl ketimines.

Example for the synthesis of *N*-*tert*-sulfinyl ketimine (*S*$_S$*)-5

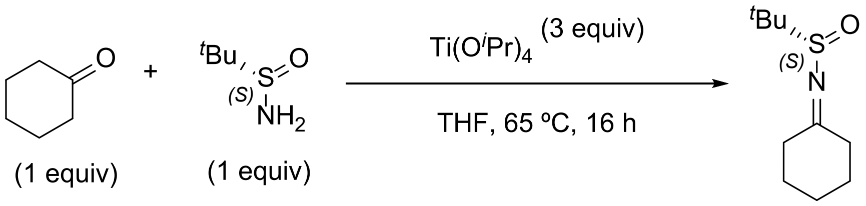

(*S*$_S$*)-5

To a solution of Ti(O^i*Pr*)$_4$ (6 mmol, 3 equiv, 1.78 mL) in anhydrous THF (7.6 mL), cyclohexanone was added (2 mmol, 1 equiv, 0.21 mL) and the mixture was stirred at room temperature for 15 min. This was followed by the slow addition of (*S*)-*tert*-butylsulfinamide (2 mmol, 1 equiv, 242.4 mg), and the reaction mixture was heated to 65 °C. When the reaction had reached completion, the reaction was allowed to cool at room temperature. The reaction was then quenched with brine (10 mL) and the resulting slurry solution was filtered through a pad of Celite, and the filter cake was washed with ethyl acetate (5 mL). The filtrate was then transferred to a separating funnel where the organic layer was washed with brine (5 mL). The aqueous layer was extracted once with ethyl acetate (15 mL), and the combined organic extracts were dried ($MgSO_4$), filtered, and concentrated under vacuum. Flash column chromatography using petroleum ether/ethyl acetate (100:8) afforded desired *N*-tert-butanesulfinyl imine **(*S*$_S$*)-5** as a yellowish oil (109 mg, 27%).

Example for the synthesis of *N*-*tert*-butanesulfinyl ketimine (*S*$_S$*)-1[4]

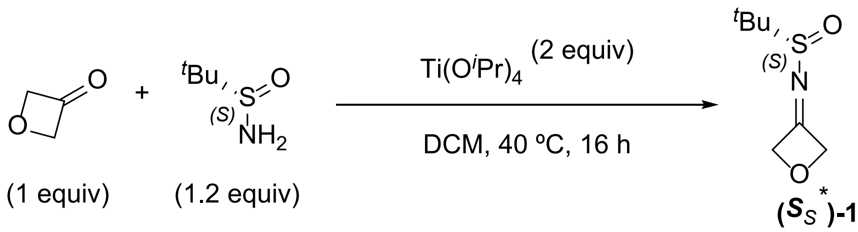

(*S*$_S$*)-1

To a mixture of oxetan-3-one (0.43 mL, 5 mmol) and (*S*)-*tert*-butylsulfinamide (605.99 mg, 5 mmol), in CH_2Cl_2 (5.6 mL), Ti(O^{i}Pr)$_4$ (1.48 mL, 5 mmol) was added at room temperature. The reaction mixture was refluxed overnight and cooled to room temperature. The reaction was then quenched with brine (10 mL) and the resulting slurry solution was filtered through a pad of Celite, and the filter cake was rinsed with CH_2Cl_2.

The organic layer of the filtrate was separated, washed with brine, dried over $MgSO_4$, filtered, and concentrated under a reduced pressure. The crude product was purified via flash chromatography on silica gel (1:1, petroleum ether/EtOAc) and concentrated under reduced pressure to afford **(*S*$_S$*)-1** as a colourless oil (524mg, 60%).

General procedure B for the synthesis of *N*-*tert*-butanesulfinyl aldimines[5]

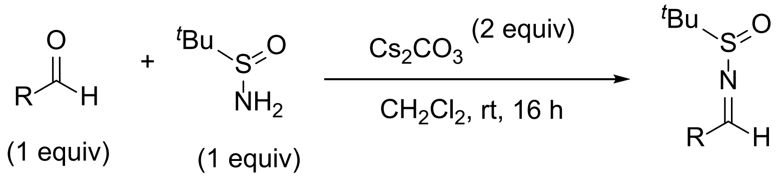

Tert-butylsulfinamide (2 mmol, 1 equiv) and the corresponding aldehyde (2 mmol, 1 equiv) were dissolved in CH_2Cl_2 (3 mL), then Cs_2CO_3 (4 mmol, 2 equiv) was added. The mixture was stirred at rt for 16 h, cooled, and filtered through a pad of celite. The solids were washed with CH_2Cl_2, and the combined filtrates were evaporated in vacuo. The corresponding *N*-*tert*-butanesulfinyl aldimines were obtained by column chromatography with the stated solvent system.

Example for the synthesis of *N*-*tert*-butanesulfinyl aldimine (*S*$_S$*)-7

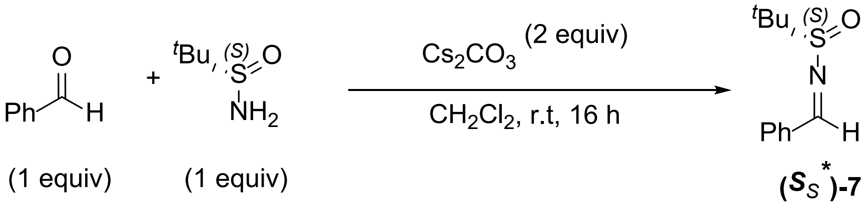

(*S*$_S$*)-7

(*S*)-*Tert*-butylsulfinamide (2 mmol, 1 equiv, 242.4 mg) and benzaldehyde (2 mmol, 1 equiv, 0.2 mL) were dissolved in CH_2Cl_2 (3 mL), then Cs_2CO_3 (4 mmol, 2 equiv, 1.3 g) was added. The mixture was stirred at rt for 16 h, cooled, and filtered through a pad of celite. The solids were washed with CH_2Cl_2, and the combined filtrates were evaporated in vacuo. The desired *N*-*tert*-butanesulfinyl aldimine **(*S*$_S$*)-7** was obtained after flash column chromatography (petroleum ether/ethyl acetate (100:8) as a white solid (377 mg, 99%).

General procedure for the aziridination of N-*tert*-butanesulfinyl ketimines and aldimines

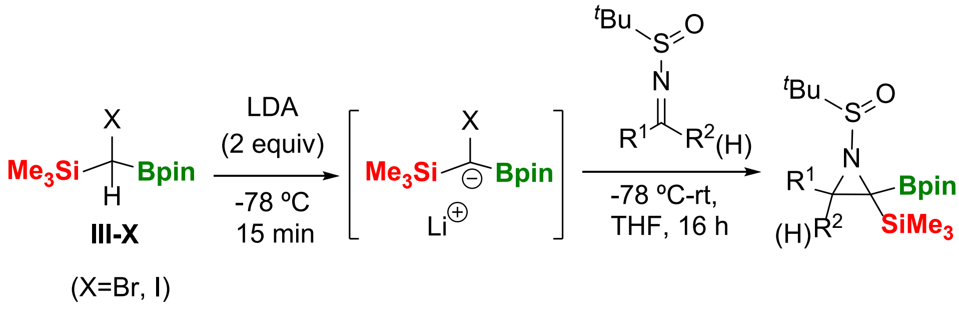

In an oven-dried Schlenk tube, charged with a magnetic stir bar, a solution of α-monohalo borylsilylmethane (**III-X**) (0.2 mmol) in 2 mL of anhydrous THF, were added. Next, the solution was cooled down to -78 °C in a dry ice bath with acetone and LDA (0.4 mL, 2 equiv) was added to the reaction mixture. After stirring the solution at -78 °C, 15 min, the corresponding *N*-*tert*-butanesulfinyl ketimine or aldimine (0.2 mmol, 1 equiv), dissolved in 0.5 mL of THF, were added to the reaction mixture and it was allowed to stir for 16 h at rt. Upon the completion of the reaction time, the solvents were evaporated under vacuum and the reaction crude was purified with flash column chromatography to afford the aziridine product.

Example for the aziridination of *N-tert*-butanesulfinyl ketimine (S_S*)-1 for the synthesis of product (S_S*,*R**)-2

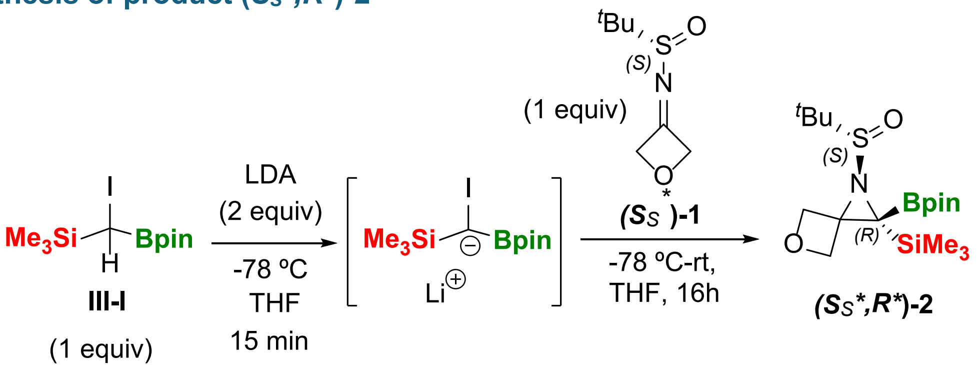

In an oven-dried Schlenk tube, charged with a magnetic stir bar, a solution of (iodo(4,4,5,5-tetramethyl-1,3,2-dioxaborolan-2-yl)methyl)trimethylsilane (68.02 mg, 0.2 mmol) in 2 mL of anhydrous THF were added. Next, the solution was cooled down to -78 °C in a dry ice bath with acetone and LDA (0.4 mL, 2 equiv) was added to the reaction mixture. After stirring the solution at -78 °C, 15 min, *N-tert*-butanesulfinyl ketimine **(S_S*)-1** (35mg, 0.2 mmol, 1 equiv), dissolved in 0.5 mL of THF, was added to the reaction mixture and it was allowed to stir for 16 h at rt. Upon the completion of the reaction time, the solvents were evaporated under vacuum and the reaction crude was purified using

silica gel chromatographic techniques to afford the aziridine product **(S_S*,R*)-2** as a white solid (26 mg, 34%).

Example for the aziridination of *N-tert*-butanesulfinyl aldimine (*S*$_S$*)-7 for the synthesis of product (*S*$_S$*,2*S**,3*S**)-8

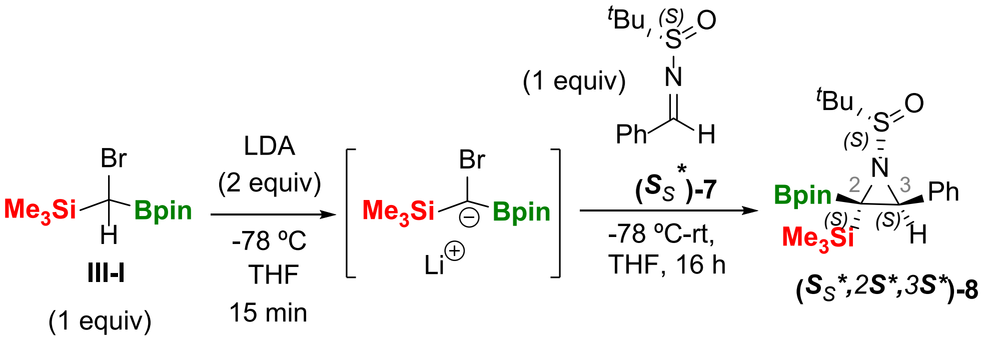

In an oven-dried Schlenk tube, charged with a magnetic stir bar, a solution of (iodo(4,4,5,5-tetramethyl-1,3,2-dioxaborolan-2-yl)methyl)trimethylsilane (58.6 mg, 0.2 mmol, 1 equiv) in 2 mL of anhydrous THF were added. Next, the solution was cooled down to -78 °C in a dry ice bath with acetone and LDA (0.4 mL, 2 equiv) was added to the reaction mixture. After stirring the solution at -78 °C, 15 min, *N*-tertbutanesulfinyl aldimine **(S_S*)-7** (41.8mg, 0.2 mmol, 1 equiv), dissolved in 0.5 mL of THF, was added to the reaction mixture and it was allowed to stir for 16 h at rt. Upon the completion of the reaction time, the solvents were evaporated under vacuum and the reaction crude was purified using silica gel chromatographic techniques to afford the aziridine product **(*S*$_S$*,2*S**,3*S**)-8** as a white yellowish solid (45 mg, 54%).

Specific example for reaction of 28 with vinyl magnesium bromide.

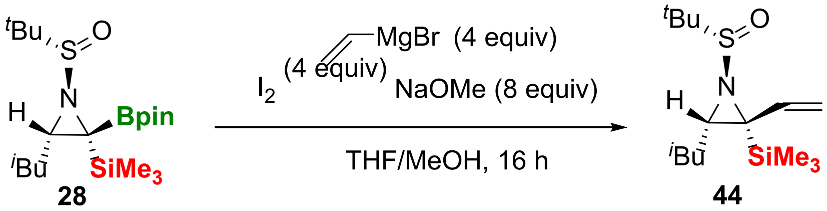

To a 25-mL flame-dried Schlenk tube charged with a THF solution (1 mL) of the aziridine **28** (80.30 mg, 1 equiv), the reagent vinyl magnesium bromide (1.14 mL, 0.7 M in THF, 4 equiv) was added dropwise and the reaction mixture was stirred at room temperature for 30 min. Subsequently, a solution of I_2 (203.05 mg, 4 equiv) in 2.70 mL of MeOH was introduced slowly to the reaction mixture at -78 °C. The reaction was then allowed to stir at this temperature for additional 30 min. A solution of NaOMe (86.44 mg, 8 equiv) in 3.30 mL of MeOH was then added slowly at -78 °C. The resulting mixture was then

warmed to rt and continued to stir at this temperature for 1 h. Saturated aqueous $Na_2S_2O_3$ (5 mL) was then added to quench the reaction. After dilution with H_2O (10 mL), the mixture was extracted with EtOAc (3 x 10 mL). The combined organic phase was dried over anhydrous $MgSO_4$. After removal of the solvent, the product was purified using silica gel chromatographic techniques to afford product **44** as a colorless oil (14 mg, 23%).

Specific example for oxidation of 28 with *m*-chloroperbenzoic acid

In an oven-dried Schlenk tube, charged with a magnetic stir bar, the reagent *m*-CPBA (69.03 mg, 2 equiv) was added. Subsequently, the aziridine **28** (80.30 mg, 1 equiv) was dissolved in 2 mL of CH_2Cl_2 and added dropwise at 0 °C. The mixture was stirred at room temperature for 18 h. Upon the completion of the reaction time, the reaction was quenched with saturated $Na_2S_2O_3$ and $NaHCO_3$. The reaction mixture was extracted with CH_2Cl_2, and the extract was washed with brine and dried over $MgSO_4$. Then the product was purified using silica gel chromatographic techniques to afford product **44** as a colorless oil (58 mg, 70%).

Specific example for reaction of 28 with CH_2Br_2/nBuLi

A flame-dried 25-mL round bottom flask equipped with a stir bar was charged with the aziridine **28** (25.0 mg, 0.06 mmol, 1.00 equiv) and evacuated and backfilled with nitrogen three times. The reagent dibromomethane (13 μL, 0.32 mmol, 3.2 equiv) dissolved in THF (0.6 mL) weas added sequentially via syringe and the mixture was cooled to -78 °C in an acetone bath. Subsequently, *n*-butyllithium (0.06 mL, 2.5 M in hexane, 0.25 mmol, 2.5 equiv) was added dropwise via syringe over 2 minutes. The reaction was stirred at -78 °C for 1 h and then placed in a -20 °C freezer for 24 h without stirring. The reaction was warmed to room temperature and quenched with H_2O (10 mL). The layers were separated, and the aqueous layer was extracted with ethyl acetate (3 x 10 mL), the combined organic layers were dried over $MgSO_4$, filtered, and concentrated in vacuo. Purification via silica gel chromatography yielded the corresponding product **46** as colorless oil (5 mg, 27%).

Specific example for protodesilylation of 28

In an oven-dried Schlenk tube, charged with a magnetic stir bar, were introduced the aziridine **28** (41.75 mg, 1 equiv) and 0.5 mL of THF. After that, a 1M solution of TBAF in THF (0.4 mL, 4 equiv) was added dropwise at 25 ºC. The reaction was stirred for 1.5 h, quenched with saturated $Na_2S_2O_3$ and passed over a silica gel plug. Then the product

was purified using silica gel chromatographic techniques to afford product **47** as a yellowish oil (3 mg, 10%).

General procedure for protodeborylation

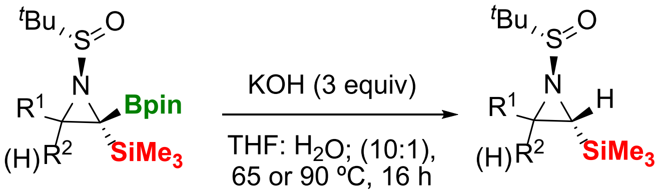

In an oven-dried Schlenk tube, charged with a magnetic stir bar, was introduced KOH (3 equiv, 33.66 mg) and the corresponding aziridine (0.2 mmol). After that, 2 mL of THF and 0.2 mL of water were added, and the reaction mixture was allowed to stir for 16 h at 65 ºC or 90 ºC depending on the substrate. Upon the completion of the reaction time, the reaction was allowed to warm to rt and the solvents were evaporated under vacuum. The reaction crude was purified using silica gel chromatographic techniques to afford the protodeborylated aziridine.

Specific example for protodeborylation of (S_S*,R*)-2 to the synthesis of (S_S*,R*)-43

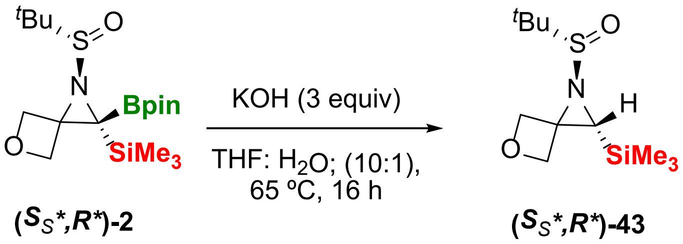

(S_S*,R*)-2 **(S_S*,R*)-43**

In an oven-dried Schlenk tube, charged with a magnetic stir bar, was introduced KOH (3 equiv, 33.66 mg) and the aziridine **(S_S*,R*)-2** (0.2 mmol, 77,48 mg). After that, 2 mL of THF and 0.2 mL of water were added, and the reaction mixture was allowed to stir for 16 h at 65 ºC. Upon the completion of the reaction time, the reaction was allowed to warm to rt and the solvents were evaporated under vacuum and the reaction crude was purified using silica gel chromatographic techniques to afford product **(S_S*,R*)-43** as a solid (32 mg, 60%).

Specific example for deuterodeborylation of (S_S*,R*)-2 to the synthesis of (S_S*,R*)-47

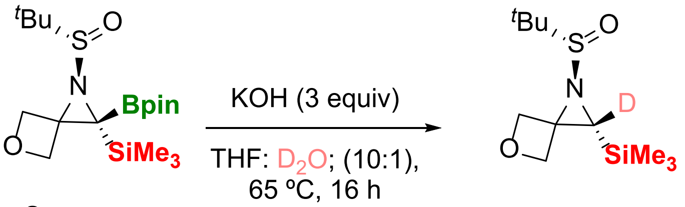

(S_S*,R*)-2 **(S_S*,R*)-47**

In an oven-dried Schlenk tube, charged with a magnetic stir bar, was introduced KOH (3 equiv, 33.66 mg) and the aziridine **(S_S*,R*)-2** (0.1 mmol, 38,74 mg). After that, 1 mL of THF and 0.1 mL of D_2O were added, and the reaction mixture was allowed to stir for 16 h at 65 ºC. Upon the completion of the reaction time, the reaction was allowed to warm to rt and the solvents were evaporated under vacuum and the reaction crude was purified using silica gel chromatographic techniques to afford product **(S_S*,R*)-47** as a yellowish oil (28 mg, 52%).

Spectral data for diborylsilymethane, α-monohalo diborylsilylmethane and α-monohalo borylsilylmethane (III) reagents.

Bis(4,4,5,5-tetramethyl-1,3,2-dioxaborolan-2-yl)methyl)trimethylsilane

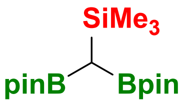

The product was synthesized following the general procedure for the synthesis of 1,1,1-diborylsilylmethane compounds[6] using chlorotrimethylsilane and was isolated using a silica gel flash column chromatography with a mixture of petroleum ether/diethyl ether (100:2 to 100:5) as eluent. The compound was obtained as a white solid (92%, 1.56 g). Mp: 62 °C

^{1}H NMR ($CDCl_3$, 400 MHz) δ 1.21 (s, 12H), 1.20 (s, 12H), 0.28 (s, 1H), 0.09 (s, 9H).

^{13}C NMR ($CDCl_3$, 100 MHz) δ 82.8, 25.1, 24.6, 0.7.

^{11}B NMR ($CDCl_3$, 128.3 MHz) δ 32.9.

HRMS (ESI) for **$C_{16}H_{34}B_2O_4SiNa$ [M+Na$^+$]$^+$: calculated:** 363.2310; **found:** 363.2309.

(Iodobis(4,4,5,5-tetramethyl-1,3,2-dioxaborolan-2-yl)methyl)trimethylsilane

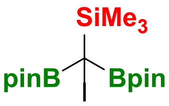

The product was synthesized following the general procedure for the synthesis of α-monohalo diborylsilylmethane and was isolated using a silica gel flash column chromatography with a mixture of petroleum ether/ethyl acetate (200:10) as eluent. The compound was obtained as a white solid (93%, 415 mg). Mp: 65 °C

^{1}H NMR ($CDCl_3$, 400 MHz) δ 1.23 (s, 24H), 0.23 (s, 9H).

^{13}C NMR ($CDCl_3$, 100 MHz) δ 84.1, 24.8, 24.6, -0.2.

^{11}B NMR ($CDCl_3$, 128.3 MHz) δ 33.0.

HRMS (ESI) for $C_{16}H_{34}B_2IO_4Si$ [M+H]$^+$: calculated: 467.1457, **found:** 467.1459.

(Bromobis(4,4,5,5-tetramethyl-1,3,2-dioxaborolan-2-yl)methyl)trimethylsilane

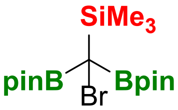

The product was synthesized following the general procedure for the synthesis of α-monohalo diborylsilylmethane and was isolated using a silica gel flash column chromatography with a mixture of petroleum ether/ethyl acetate (200:10) as eluent. The compound was obtained as a white solid (72%, 302 mg). Mp: 78 °C

^{1}H NMR ($CDCl_3$, 400 MHz) δ 1.25 (s, 12H), 1,24 (s, 12H), 0.22 (s, 9H).

^{13}C NMR ($CDCl_3$, 100 MHz) δ 84.2, 24.9, 24.6, -1.2.

^{11}B NMR ($CDCl_3$, 128.3 MHz) δ 32.6.

HRMS (ESI) for $C_{16}H_{34}B_2BrO_4Si$ [M+H]$^+$: calculated: 419.1590, **found:** 419.1597.

(Iodo(4,4,5,5-tetramethyl-1,3,2-dioxaborolan-2-yl)methyl)trimethylsilane (III-I)

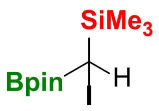

III-I

The product was synthesized following the general procedure for the protodeborylation of α-monohalo borylsilylmethane and was isolated using a silica gel flash column chromatography with a mixture of petroleum ether/diethyl ether (100:1 to 100:2) as eluent. The compound **III-I** was obtained as a pale yellowish oil (65%, 442 mg).

^{1}**H NMR** ($CDCl_3$, 400 MHz) δ 1.85 (s, 1H), 1.25 (s, 24H), 0.19 (s, 9H).

^{13}**C NMR** ($CDCl_3$, 100 MHz) δ 84.1, 24.7, 24.6, -1.0.

^{11}**B NMR** ($CDCl_3$, 128.3 MHz) δ 32.7.

HRMS (ESI) for $C_{10}H_{23}BIO_2Si$ [M+H]$^+$: calculated: 341.0600**, found:** 341.0604.

(Bromo(4,4,5,5-tetramethyl-1,3,2-dioxaborolan-2-yl)methyl)trimethylsilane (III-Br)

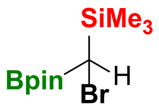

III-Br

The product was synthesized following the general procedure for the protodeborylation of -monohalo borylsilylmethane and was isolated using a silica gel flash column chromatography with a mixture of petroleum ether/diethyl ether (100:1 to 100:2) as eluent. The compound **III-Br** was obtained as a pale yellowish oil (40%, 234 mg).

^{1}**H NMR** ($CDCl_3$, 400 MHz) δ 2.35 (s, 1H), 1.27 (s, 12H), 0.17 (s, 9H).

^{13}**C NMR** ($CDCl_3$, 100 MHz) δ 84.3, 24.9, 24.8, -1.8.

^{11}**B NMR** ($CDCl_3$, 128.3 MHz) δ 32.3.

HRMS (ESI) for $C_{10}H_{23}BBrO_2Si$ [M+H]$^+$: calculated: 293.0738**, found:** 293.0741.

Spectral data for *N*-*tert*-butanesulfinyl ketimines and aldimines

(*S*)-2-methyl-*N*-(oxetan-3-ylidene)propane-2-sulfinamide (*S*$_S$*)-1

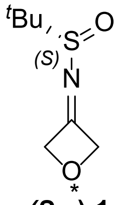

(*S*$_S$)-1

The product was synthesized following the procedure for the synthesis of *N*-*tert*-butanesulfinyl ketimine **(*S*$_S$*)-1** and purified by flash chromatography using as eluent a mixture of petroleum ether/ethyl acetate (50:50). The product **(*S*$_S$*)-1** was isolated as a colorless oil (524 mg, 2.99 mmol, 60%). *The product **1** was purchased from BLDPharm.*

$[\alpha]_D^{20}$: +433.4 (c = 1.0, CH_2Cl_2).

^{1}**H NMR (400 MHz, $CDCl_3$)** δ= 5.79 (ddd, *J* = 15.5, 4.3, 2.3 Hz, 1H), 5.65 (ddd, *J* = 15.4, 4.2, 1.9 Hz, 1H), 5.52 – 5.39 (m, 2H), 1.26 (s, 9H).

^{13}**C NMR (100 MHz, $CDCl_3$)** δ= 176.4, 86.3, 86.1, 58.2, 22.4.

HRMS-(ESI+) for $C_7H_{14}NO_2S$ [M+H]$^+$: calculated 176.0740; found: 176.0737

(*S*)- 2-methyl-*N*-(tetrahydro-4H-pyran-4-ylidene)propane-2-sulfinamide (*S*$_S$*)-3

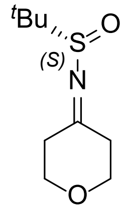

(*S*$_S$*)-3

The product was synthesized following the general procedure A for the synthesis of *N-tert*-butanesulfinyl ketimines and purified by flash chromatography using as eluent a mixture of petroleum ether/ethyl acetate (100:8). The product **(*S*$_S$*)-3** was isolated as a white solid (80 mg, 0.39 mmol, 20%). *The product **3** was isolated as a white solid (89 mg, 0.44 mmol, 22%).* M.p. = 75.2-77.6 ºC.

$[\alpha]_D^{20}$: +68.3 (c = 1.0, CH_2Cl_2).

^{1}H NMR (400 MHz, $CDCl_3$) δ= 3.92 (td, *J* = 6.3, 5.0 Hz, 2H), 3.84 (ddd, *J* = 6.5, 5.0, 1.3 Hz, 2H), 3.15 (dt, *J* = 14.3, 5.7 Hz, 1H), 2.88 (dd, *J* = 14.4, 6.3 Hz, 1H), 2.55 (t, *J* = 5.7 Hz, 2H), 1.24 (s, 9H).

^{13}C NMR (100 MHz, $CDCl_3$) δ= 182.5, 68.8, 67.8, 56.7, 41.0, 35.4, 22.4.

HRMS-(ESI+) for $C_9H_{18}NO_2S$ [M+H]$^+$: calculated 204.1058; found: 204.1053.

(*S*)-*N*-cyclohexylidene-2-methylpropane-2-sulfinamide (*S*$_S$*)-5

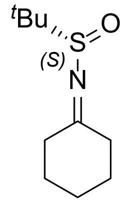

***(S*$_S$*)-5**

The product was synthesized following the general procedure A for the synthesis of *N-tert*-butanesulfinyl ketimines and purified by flash chromatography using as eluent a mixture of petroleum ether/ethyl acetate (100:8). The product **(*S*$_S$*)-5** was isolated as a yellowish oil (109 mg, 0.54 mmol, 27%). *The product **5** was isolated as a yellowish oil (101 mg, 0.50 mmol, 25%).*

$[\alpha]_D^{20}$: +128.4 (c = 1.0, CH_2Cl_2).

^{1}H NMR (400 MHz, $CDCl_3$) δ= 2.88 (ddd, *J* = 13.4, 7.9, 4.9 Hz, 1H), 2.72 (ddd, *J* = 13.6, 7.9, 4.7 Hz, 1H), 2.42 (t, *J* = 6.5 Hz, 2H), 1.92 – 1.70 (m, 4H), 1.69 – 1.62 (m, 2H), 1.22 (s, 9H).

^{13}C NMR (100 MHz, $CDCl_3$) δ= 188.8, 56.1, 40.8, 34.5, 28.0, 27.5, 25.5, 22.2.

HRMS-(ESI+) for $C_{10}H_{20}NOS$ [M+H]$^+$: calculated 202.1266; found: 202.1261.

(*S*)-*N*-benzylidene-2-methylpropane-2-sulfinamide (*S*$_S$*)-7

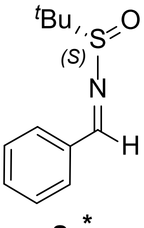

(*S*$_S$*)-7

The product was synthesized following the general procedure B for the synthesis of *N-tert*-butanesulfinyl aldimines and purified by flash chromatography using as eluent a mixture of petroleum ether/ethyl acetate (100:8). The product **(*S*$_S$*)-7** was isolated as a white solid (377 mg, 2.0 mmol, 99%). *The product **7** was isolated as a white solid (915 mg, 4.9 mmol, 96%).* M.p. = 50.4-51.4 ºC.

$[\alpha]_D^{20}$: +67.9 (c = 1.0, CH_2Cl_2).

^{1}H NMR (400 MHz, $CDCl_3$) δ= 8.60 (s, 1H), 7.90 – 7.82 (m, 2H), 7.56 – 7.44 (m, 3H), 1.27 (s, 9H).

^{13}C NMR (100 MHz, $CDCl_3$) δ=162.9, 134.3, 132.6, 129.5, 129.1, 57.9, 22.8.

HRMS-(ESI+) for $C_{11}H_{16}NOS$ [M+H]$^+$: calculated 210.0947; found: 210.0944.

(*S*)-*N*-(4-chlorobenzylidene)-2-methylpropane-2-sulfinamide (*S*$_S$*)-9

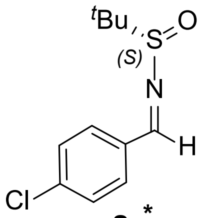

(*S*$_S$*)-9

The product was synthesized following the general procedure B for the synthesis of *N*-*tert*-butanesulfinyl aldimines using as eluent a mixture of petroleum ether/ethyl acetate (100:8). The product **(*S*$_S$*)-9** was isolated as a white solid (390 mg, 1.6 mmol, 88%). *The product **9** was isolated as a white solid (439 mg, 2.0 mmol, 99%).* M.p. = 70.4 - 70.8 ºC.

$[\alpha]_D^{20}$: +64.2 (c = 1.0, CH_2Cl_2).

^{1}H NMR (400 MHz, $CDCl_3$) δ= 8.55 (s, 1H), 7.83 – 7.75 (m, 2H), 7.49 – 7.41 (m, 2H), 1.26 (s, 9H).

^{13}C NMR (100 MHz, $CDCl_3$) δ= 161.6, 138.8, 132.7, 130.7, 129.5, 58.1, 22.8.

HRMS-(ESI+) for $C_{11}H_{15}ClNOS$ [M+H]$^+$: calculated 244.0563; found: 244.0558.

(*S*)-2-methyl-*N*-(4-methylbenzylidene)propane-2-sulfinamide (*S*$_S$*)-11

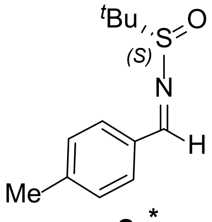

(*S*$_S$*)-11

The product was synthesized following the general procedure B for the synthesis of *N*-*tert*-butanesulfinyl aldimines using as eluent a mixture of petroleum ether/ethyl acetate (100:8). The product **(*S*$_S$*)-11** was isolated as a white solid (440 mg, 2.0 mmol, 99%). *The product **11** was isolated as a white solid (421 mg, 1.9 mmol, 94%).* M.p. = 70.5 - 71.3 ºC.

$[\alpha]_D^{20}$: +67.8 (c = 1.0, CH_2Cl_2).

^{1}H NMR (400 MHz, $CDCl_3$) δ= 8.55 (s, 1H), 7.78 – 7.71 (m, 2H), 7.31 – 7.24 (m, 2H), 2.42 (s, 3H), 1.26 (s, 9H).

^{13}C NMR (100 MHz, $CDCl_3$) δ= 162.7, 143.3, 131.8, 129.8, 129.6, 57.8, 22.7, 21.9.

HRMS-(ESI+) for $C_{12}H_{18}NOS$ [M+H]$^+$: calculated 224.1109; found: 224.1105.

(*S*)-*N*-(2-fluorobenzylidene)-2-methylpropane-2-sulfinamide (S_S*)-13

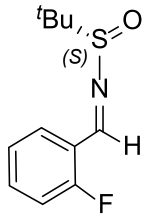

(S_S*)-13

The product was synthesized following the general procedure B for the synthesis of *N*-*tert*-butanesulfinyl aldimines and purified by flash chromatography using as eluent a mixture of petroleum ether/ethyl acetate (100:8). The product **(S_S*)-13** was isolated as a dense colorless oil (451 mg, 2 mmol, 99%).*The product **13** was isolated as a dense colorless oil (287 mg, 1.3 mmol, 63%)*

$[\alpha]_D^{20}$: +101.0 (c = 1.0, CH_2Cl_2).

^{1}H NMR (400 MHz, $CDCl_3$) δ= 8.91 (s, 1H), 8.09 – 7.91 (m, 1H), 7.59 – 7.45 (m, 1H), 7.26 – 7.21 (m, 1H), 7.15 (ddd, *J* = 10.4, 8.4, 1.1 Hz, 1H), 1.27 (s, 9H).

^{13}C NMR (100 MHz, $CDCl_3$) δ= 162.7 (d, $^1J_{C-F}$ = 257 Hz), 156.7 (d, $^3J_{C-F}$ = 5 Hz), 134.3 (d, $^3J_{C-F}$ = 8 Hz), 128.8 (d, $^4J_{C-F}$ = 2 Hz), 124.7 (d, $^3J_{C-F}$ = 4 Hz), 122.2 (d, $^2J_{C-F}$ = 9 Hz), 116.5 (d, $^2J_{C-F}$ = 21 Hz), 58.1, 22.8

^{19}F NMR (377 MHz, $CDCl_3$) δ= -117.9.

HRMS-(ESI+) for $C_{11}H_{15}FNOS$ [M+H]$^+$: calculated 228.0858; found: 228.0853.

(*S*)-2-methyl-*N*-(2-(trifluoromethyl)benzylidene)propane-2-sulfinamide (S_S*)-15

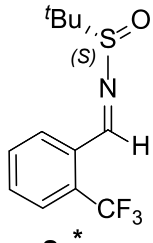

(S_S*)-15

The product was synthesized following the general procedure B for the synthesis of *N*-*tert*-butanesulfinyl aldimines and purified by flash chromatography using as eluent a mixture of petroleum ether/ethyl acetate (100:8). The product **(S_S*)-15** was isolated as a dense colorless oil (532 mg, 1.9 mmol, 96%). *The product **15** was isolated as a dense colorless liquid (439 mg, 1.6 mmol, 79%).*

$[\alpha]_D^{20}$: +106.0 (c = 1.0, CH_2Cl_2).

^{1}H NMR (400 MHz, $CDCl_3$) δ= 8.90 (d, *J* = 2.1 Hz, 1H), 8.19 – 8.09 (m, 1H), 7.74 – 7.66 (m, 1H), 7.62 – 7.46 (m, 2H), 1.21 (s, 9H).

^{13}C NMR (100 MHz, $CDCl_3$) δ= 159.5 (q, $^3J_{C-F}$ = = 2 Hz), 132.1, 131.6, 129.9 (q, $^2J_{C-F}$ = 32 Hz), 129.2, 126.1 (q, $^3J_{C-F}$ = 6 Hz), 125.1, 123.7 (q, $^1J_{C-F}$ = 274.3 Hz), 58.2, 22.7.

^{19}F NMR (377 MHz, $CDCl_3$) δ= -57.2.

HRMS-(ESI+) for $C_{12}H_{15}F_3NOS$ [M+H]$^+$: calculated 278.0826; found: 278.0822.

(*S*)-2-methyl-*N*-(pyridin-2-ylmethylene)propane-2-sulfinamide (S_S*)-17

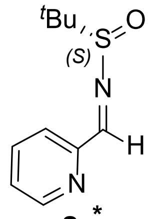

(S_S*)-17

The product was synthesized following the general procedure B for the synthesis of *N-tert*-butanesulfinyl aldimines and purified by flash chromatography using as eluent a mixture of petroleum ether/ethyl acetate (50:50). The product **(S_S*)-17** was isolated as a white solid (543 mg, 2.6 mmol, 57%). *The product* ***17*** *was isolated as a white solid (579 mg, 2.8 mmol, 61%).*

M.p. = 42.0-42.6 ºC. $[\alpha]_D^{20}$: +127.3 (c = 1.0, CH_2Cl_2).

^{1}H NMR (400 MHz, $CDCl_3$) δ= 8.75 (ddd, *J* = 4.8, 1.7, 0.9 Hz, 1H), 8.70 (d, *J* = 0.6 Hz, 1H), 8.02 (dt, *J* = 7.9, 1.1 Hz, 1H), 7.81 (tdd, *J* = 7.8, 1.7, 0.6 Hz, 1H), 7.40 (ddd, *J* = 7.5, 4.8, 1.2 Hz, 1H), 1.29 (s, 9H).

^{13}C NMR (100 MHz, $CDCl_3$) δ=163.9, 152.7, 150.4, 136.9, 126.0, 123.3, 58.2, 22.9.

HRMS-(ESI+) for $C_{10}H_{15}N_2OS$ [M+H]$^+$: calculated 211.0900; found: 211.0898.

(*S*)-2-methyl-*N*-(pyridin-3-ylmethylene)propane-2-sulfinamide (S_S*)-19

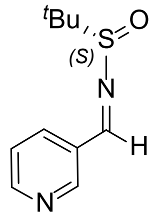

(S_S*)-19

The product was synthesized following the general procedure B for the synthesis of *N-tert*-butanesulfinyl aldimines and purified by flash chromatography using as eluent a mixture of DCM/ethyl acetate (80:20). The product **(S_S*)-19** was isolated as a dense colorless oil (295 mg, 1.4 mmol, 70%). *The product* ***19*** *was isolated as dense colorless oil (355 mg, 1.7 mmol, 84%).*

$[\alpha]_D^{20}$: +133.2 (c = 1.0, CH_2Cl_2).

^{1}H NMR (400 MHz, $CDCl_3$) δ= 9.03 (s, 1H), 8.74 (d, *J* = 4.7, 2.0 Hz, 1H), 8.65 (s, 1H), 8.16 (dd, *J* = 7.9, 2.0 Hz, 1H), 7.42 (d, *J* = 7.7, 1.5 Hz, 1H), 1.27 (s, 9H).

^{13}C NMR (100 MHz, $CDCl_3$) δ= 160.6, 153.1, 151.2, 135.8, 129.8, 124.1, 58.3, 22.8.

HRMS-(ESI+) for $C_{10}H_{15}N_2OS$ [M+H]$^+$: calculated 211.0905; found: 211.0901.

(*S*)-2-methyl-*N*-(pyrimidin-4-ylmethylene)propane-2-sulfinamide (S_S*)-21

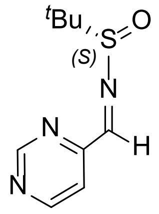

(S_S*)-21

The product was synthesized following the general procedure B for the synthesis of *N-tert*-butanesulfinyl aldimines and purified by flash chromatography using as eluent a mixture of petroleum ether/ethyl acetate (100:100). The product **(S_S*)-21** was isolated as a reddish solid (327 mg, 1.6 mmol, 77%). T*he product* ***21*** *was isolated as a reddish solid (330 mg, 1.6 mmol, 78%).* M.p. = 43.2 - 43.7 ºC.

$[\alpha]_D^{20}$: +28.3 (c = 1.0, CH_2Cl_2).

^{1}H NMR (400 MHz, $CDCl_3$) δ= 9.38 (d, *J* = 1.4 Hz, 1H), 8.91 (dd, *J* = 5.1, 0.6 Hz, 1H), 8.65 (d, *J* = 0.6 Hz, 1H), 7.92 (dd, *J* = 5.1, 1.4 Hz, 1H), 1.29 (s, 9H).

^{13}C NMR (100 MHz, $CDCl_3$) δ= 162.7, 159.8, 158.4, 118.7, 59.0, 22.9.

HRMS-(ESI+) for $C_9H_{14}N_3OS$ [M+H]$^+$: calculated 212.0858; found: 212.0748.

(*S*)-2-methyl-*N*-(naphthalen-2-ylmethylene)propane-2-sulfinamide (*S*$_S$*)-23

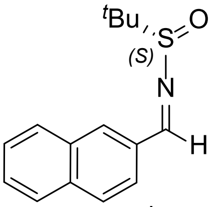

(*S*$_S$*)-23

The product was synthesized following the general procedure B for the synthesis of *N*-*tert*-butanesulfinyl aldimines and purified by flash chromatography using as eluent a mixture of petroleum ether/ethyl acetate (100:8). The product **(*S*$_S$*)-23** was isolated as a white solid (682 mg, 2.6 mmol, 58%). *The product **23** was isolated as a white solid (284 mg, 1.1 mmol, 24%)*. M.p. = 111.3-112.2 ºC.

$[\alpha]_D^{20}$: +159.8(c = 1.0, CH_2Cl_2).

^{1}H NMR (400 MHz, $CDCl_3$) δ= 8.75 (s, 1H), 8.24 – 8.18 (m, 1H), 8.04 (dd, *J* = 8.6, 1.7 Hz, 1H), 7.98 – 7.93 (m, 1H), 7.92 – 7.86 (m, 2H), 7.62 – 7.53 (m, 2H), 1.30 (s, 9H).

^{13}C NMR (100 MHz, $CDCl_3$) δ=162.9, 135.5, 133.1, 132.6, 131.9, 129.3, 129.0, 128.4, 128.1, 127.1, 124.0, 58.0, 22.8.

HRMS-(ESI+) for $C_{15}H_{18}NOS$ [M+H]$^+$: calculated 260.1104; found: 260.1105.

(*S*)-2-methyl-*N*-(quinolin-4-ylmethylene)propane-2-sulfinamide (*S*$_S$*)-25

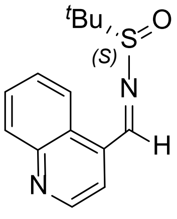

(*S*$_S$*)-25

The product was synthesized following the general procedure B for the synthesis of *N*-*tert*-butanesulfinyl aldimines and purified by flash chromatography using as eluent a mixture of petroleum ether/ethyl acetate (50:50). The product **(*S*$_S$*)-25** was isolated as a white solid (117 mg, 0.5 mmol, 25%). *The product **25** was isolated as a white solid (279 mg, 1.1 mmol, 59%).* M.p. = 89.1 – 89.7 ºC.

$[\alpha]_D^{20}$: +63.8 (c = 1.0, CH_2Cl_2).

^{1}H NMR (400 MHz, $CDCl_3$) δ= 9.21 (s, 1H), 9.08 (d, *J* = 4.4 Hz, 1H), 8.84 – 8.76 (m, 1H), 8.26 – 8.18 (m, 1H), 7.85 (d, *J* = 4.4 Hz, 1H), 7.81 (ddd, *J* = 8.4, 6.9, 1.4 Hz, 1H), 7.73 – 7.67 (m, 1H), 1.34 (s, 9H).

^{13}C NMR (100 MHz, $CDCl_3$) δ=161.1, 150.5, 149.4, 136.3, 130.6, 130.0, 128.5, 125.4, 124.0, 122.4, 58.5, 22.9.

HRMS-(ESI+) for $C_{14}H_{17}N_2OS$ [M+H]$^+$: calculated 261.1062; found: 261.1056.

(*S*)-methyl-*N*-(3-methylbutylidene)propane-2-sulfinamide (*S*$_S$*)-27

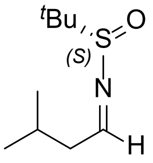

(*S*$_S$*)-27

The product was synthesized following the general procedure B for the synthesis of *N*-*tert*-butanesulfinyl aldimines and purified by flash chromatography using as eluent a mixture of petroleum ether/ethyl acetate (100:4). The product **(*S*$_S$*)-27** was isolated as a colorless oil (300 mg, 1.6 mmol, 79%). *The product **27** was isolated as a colorless oil (244 mg, 1.3 mmol, 64%).*

$[\alpha]_D^{20}$: +277.4 (c = 1.0, CH_2Cl_2).

^{1}H NMR (400 MHz, $CDCl_3$) δ= 8.06 (t, *J* = 5.2 Hz, 1H), 2.41 (ddd, *J* = 6.5, 5.2, 1.3 Hz, 2H), 2.06 (dp, *J* = 13.5, 6.7 Hz, 1H), 1.20 (s, 9H), 0.99 (dd, *J* = 6.7, 1.1 Hz, 6H).

^{13}C NMR (100 MHz, $CDCl_3$) δ= 169.6, 56.7, 45.1, 26.3, 22.8, 22.7, 22.5.

HRMS-(ESI+) for $C_9H_{20}NOS$ [M+H]$^+$: calculated 190.1265; found: 190.1258.

(*S*)-*N*-(5-chloropentylidene)-2-methylpropane-2-sulfinamide (*S*$_S$*)-29

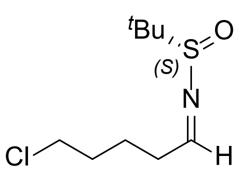

(*S*$_S$*)-29

The product was synthesized following the general procedure B for the synthesis of *N*-*tert*-butanesulfinyl aldimines and purified by flash chromatography using as eluent a mixture of petroleum ether/ethyl acetate (100:4). The product **(*S*$_S$*)-29** was isolated as a colorless oil (289 mg, 1.3 mmol, 65%). *The product **29** was isolated as a colorless oil (212 mg, 0.9 mmol, 47%).*

$[\alpha]_D^{20}$: +299.6 (c = 1.0, CH_2Cl_2).

^{1}H NMR (400 MHz, $CDCl_3$) δ= 8.08 (t, *J* = 4.4 Hz, 1H), 3.56 (t, *J* = 6.2 Hz, 2H), 2.61 – 2.52 (m, 2H), 1.92 – 1.74 (m, 4H), 1.19 (s, 9H).

^{13}C NMR (100 MHz, $CDCl_3$) δ= 168.9, 56.8, 44.6, 35.4, 32.1, 22.8, 22.5.

HRMS-(ESI+) for $C_9H_{19}ClNOS$ [M+H]$^+$: calculated 224.0876; found: 224.0871.

(*S*)-*N*-(cyclohexylmethylene)-2-methylpropane-2-sulfinamide (*S*$_S$*)-31

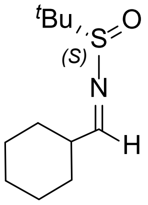

(*S*$_S$*)-31

The product was synthesized following the general procedure B for the synthesis of *N*-*tert*-butanesulfinyl aldimines and purified through a short plug of silica gel with ethyl acetate. The product **(*S*$_S$*)-31** was isolated as colorless oil (369 mg, 1.9 mmol, 94%). *The product **31** was isolated as a colorless oil (377 mg, 1.9 mmol, 96%).*

[α]D^{20}: +230.4 (c = 1.0, CH_2Cl_2).

^{1}H NMR (400 MHz, $CDCl_3$) δ=7.93 (d, *J* = 4.5 Hz, 1H), 2.50 – 2.36 (m, 1H), 1.92 – 1.80 (m, 2H), 1.80 – 1.68 (m, 2H), 1.70 – 1.62 (m, 1H), 1.35 – 1.27 (m, 4H), 1.15 (s, 9H).

^{13}C NMR (100 MHz, $CDCl_3$) δ= 172.8, 56.5, 44.1, 29.4, 26.0, 25.5, 25.4, 22.4.

HRMS-(ESI+) for $C_{11}H_{22}NOS$ [M+H]$^+$: calculated 216.1422; found: 216.1417.

(*S*)-*N*-((*S*)-3,7-dimethyloct-6-en-1-ylidene)-2-methylpropane-2-sulfinamide (*S*$_S$*,S)-33

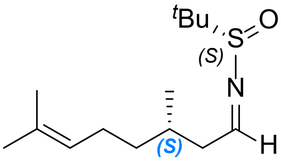

(*S*$_S$*,*S*)-33

The product was synthesized following the general procedure B for the synthesis of *N-tert*-butanesulfinyl aldimines and purified by flash chromatography using as eluent a mixture of petroleum ether/ethyl acetate (100:4). The product **(*S*$_S$*,S)-33** was isolated as a bright yellow oil (363 mg, 1.4 mmol, 71%). *The product* ***(S)-33*** *was isolated as a bright yellow oil (409 mg, 1.6 mmol, 79%).*

$[\alpha]_D^{20}$: +239.5 (c = 1.0, CH_2Cl_2).

^{1}H NMR (400 MHz, $CDCl_3$) δ= 8.06 (t, *J* = 5.2 Hz, 1H), 5.07 (tdq, *J* = 7.1, 2.9, 1.4 Hz, 1H), 2.51 (dt, *J* = 15.0, 5.3 Hz, 1H), 2.36 (ddd, *J* = 15.0, 7.8, 5.4 Hz, 1H), 2.10 – 1.85 (m, 3H), 1.68 (s, 3H), 1.60 (s, 3H), 1.47 – 1.34 (m, 1H), 1.32 – 1.22 (m, 1H), 1.20 (s, 9H), 0.97 (d, *J* = 6.7 Hz, 3H).

^{13}C NMR (100 MHz, $CDCl_3$) δ= 169.6, 131.8, 124.3, 56.7, 43.4, 37.0, 30.6, 25.8, 25.5, 22.5, 19.9, 17.8.

HRMS-(ESI+) for $C_{14}H_{28}NOS$ [M+H]$^+$: calculated 258.1892; found: 258.1885.

(*S*)-2-methyl-*N*-((*E*)-3-phenylbutylidene)propane-2-sulfinamide (*S*$_S$*,rac)-35

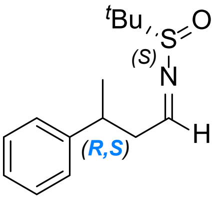

(*S*$_S$*,*rac*)-35

The product was synthesized following the general procedure B for the synthesis of *N-tert*-butanesulfinyl aldimines and purified by flash chromatography using as eluent a mixture of petroleum ether/ethyl acetate (100:4). The product **(*S*$_S$*,rac)-35** was isolated as a colorless oil and as a mixture of diasteroisomers (477 mg, 1.9 mmol, 95%). *The product* ***(rac)-35*** *was isolated as a colorless oil and as a mixture of diasteroisomers (338 mg, 1.3 mmol, 67%).*

Diasteroisomer 1+2 $[\alpha]_D^{20}$: +191.3 (c = 1.0, CH_2Cl_2).

^{1}H NMR (400 MHz, $CDCl_3$) diasteroisomer 1 δ= 8.02 (dd, *J* = 4.9, 4.2 Hz, 1H), 7.33 – 7.14 (m, 5H), 3.32 – 3.16 (m, 1H), 2.96 – 2.72 (m, 2H), 1.35 (d, *J* = 7.0 Hz, 3H), 1.08 (s, 9H). **diasteroisomer 2** δ= 7.98 (dd, *J* = 5.6, 4.6 Hz, 1H), 7.33 – 7.14 (m, 5H), 3.32 – 3.16 (m, 1H), 2.96 – 2.72 (m, 2H), 1.33 (d, *J* = 7.0 Hz, 3H), 1.05 (s, 9H).

^{13}C NMR (100 MHz, $CDCl_3$) diasteroisomer 1 δ= 168.6, 145.8, 128.8, 127.0, 126.6, 56.7, 44.5, 37.4, 22.7, 22.4. **diasteroisomer 2** δ= 168.4, 145.5, 128.7, 126.9, 126.5, 56.7, 44.1, 36.9, 22.6, 22.3.

HRMS-(ESI+) for $C_{14}H_{22}NOS$ [M+H]$^+$: calculated 252.1422; found: 252.1414.

(*S*)-2-methyl-*N*-((*E*)-2-methylbutylidene)propane-2-sulfinamide (S_S*,rac)-37

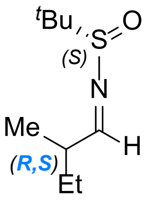

(S_S*,*rac*)-37

The product was synthesized following the general procedure B for the synthesis of *N-tert*-butanesulfinyl aldimines and purified by flash chromatography using as eluent a mixture of petroleum ether/ethyl acetate (100:4). The product **(S_S*,rac)-37** was isolated as a colorless oil and as a mixture of diasteroisomers 1:1 (323 mg, 1.7 mmol, 85%). *The product* ***(rac)-37*** *was isolated as a colorless oil and as a mixture of diasteroisomers 1:1 (209 mg, 1.1 mmol, 55%).*

Diasteroisomer 1+2 $[\alpha]_D^{20}$: +155.4 (c = 1.0, CH_2Cl_2).

^{1}H NMR (400 MHz, $CDCl_3$) diasteroisomer 1 δ= 7.97 (d, *J* = 2.6 Hz, 1H), 2.59 – 2.48 (m, 1H), 1.73 – 1.62 (m, 1H), 1.56 – 1.44 (m, 1H), 1.20 (s, 9H), 1.14 (d, *J* = 3.8 Hz, 3H), 0.94 (td, *J* = 7.5, 1.7 Hz, 3H).**diasteroisomer 2** δ= 7.95 (d, *J* = 2.9 Hz, 1H), 2.59 – 2.48 (m, 1H), 1.73 – 1.62 (m, 1H), 1.56 – 1.44 (m, 1H), 1.19 (s, 9H), 1.13 (d, *J* = 3.8 Hz, 3H), 0.94 (td, *J* = 7.5, 1.7 Hz, 3H).

^{13}C NMR (100 MHz, $CDCl_3$) diasteroisomer 1 δ= 173.6, 56.6, 41.8, 26.8, 22.5, 16.6, 11.8. **diasteroisomer 2** δ= 173.5, 56.6, 41.7, 26.8, 22.5, 16.6, 11.7

HRMS-(ESI+) for $C_9H_{20}NOS$ [M+H]$^+$: calculated 190.1266; found: 190.1268.

(*S*)-2-methyl-*N*-((*E*)-2-phenylpropylidene)propane-2-sulfinamide (S_S*,rac)-39

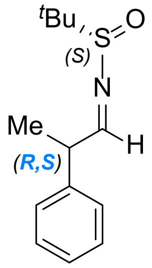

(S_S*,*rac*)-39

The product was synthesized following the general procedure B for the synthesis of *N-tert*-butanesulfinyl aldimines and purified by flash chromatography using as eluent a mixture of petroleum ether/ethyl acetate (100:4). The product **(S_S*,rac)-39** was isolated as a colorless oil and as a mixture of diasteroisomers 1:1 (468 mg, 2.0 mmol, 99%). *The product* ***(rac)-39*** *was isolated as a colorless oil and as a mixture of diasteroisomers 1:1 (341 mg, 1.4 mmol, 72%).*

Diasteroisomer 1+2 $[\alpha]_D^{20}$: +284.4 (c = 1.0, CH_2Cl_2).

^{1}H NMR (400 MHz, $CDCl_3$) diasteroisomer 1 δ= 8.12 (d, *J* = 1.2 Hz, 1H), 7.38 – 7.31 (m, 2H), 7.29 – 7.22 (m, 3H), 3.95 – 3.83 (m, 1H), 1.55 (d, *J* = 5.3 Hz, 3H), 1.22 (s, 9H). **diasteroisomer 2** δ= 8.11 (d, *J* = 1.2 Hz, 1H), 7.38 – 7.31 (m, 2H), 7.29 – 7.22 (m, 3H), 3.95 – 3.83 (m, 1H), 1.53 (d, *J* = 5.3 Hz, 3H), 1.18 (s, 9H).

^{13}C NMR (100 MHz, $CDCl_3$) diasteroisomer 1 δ= 171.2, 140.8, 129.0, 127.9, 127.4, 57.1, 46.2, 22.6, 18.0. **diasteroisomer 2** δ= 170.9, 140.8, 129.0, 127.9, 127.4, 56.9, 46.2, 22.5, 18.0.

HRMS-(ESI+) for $C_{13}H_{20}NOS$ [M+H]$^+$: calculated 238.1266; found: 238.1258.

(*S*)-2-methyl-*N*-((*E*)-2-phenylbutylidene)propane-2-sulfinamide (S_S*,rac)-41

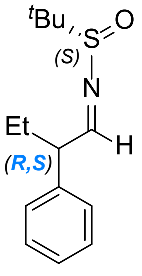

(S_S*,*rac*)-41

The product was synthesized following the general procedure B for the synthesis of *N-tert*-butanesulfinyl aldimines and purified by flash chromatography using as eluent a mixture of petroleum ether/ethyl acetate (100:4). The product **(S_S*,rac)-39** was isolated as a colorless oil and as a mixture of diasteroisomers 1:1 (136 mg, 0.5 mmol, 77%). *The product* ***(rac)-39*** *was isolated as a colorless oil and as a mixture of diasteroisomers 1:1 (127 mg, 0.5 mmol, 72%).*

Diasteroisomer 1+2 $[\alpha]_D^{20}$: +231.0 (c = 1.0, CH_2Cl_2).

^{1}H NMR (400 MHz, $CDCl_3$) diasteroisomer 1 δ= 8.10 (d, *J* = 0.7 Hz, 1H), 7.41 – 7.29 (m, 3H), 7.23 – 7.20 (m, 2H), 3.70 – 3.57 (m, 1H), 2.11 – 2.02 (m, 1H), 1.94 – 1.84 (m, 1H), 1.21 (s, 9H), 0.94 – 0.90 (m, 3H). **diasteroisomer 2** δ= 8.08 (s, 1H), 7.41 – 7.29 (m, 3H), 7.23 – 7.20 (m, 2H), 3.70 – 3.57 (m, 1H), 2.11 – 2.02 (m, 1H), 1.94 – 1.84 (m, 1H), 1.14 (s, 9H), 0.94 – 0.90 (m, 3H).

^{13}C NMR (100 MHz, $CDCl_3$) diasteroisomer 1 δ=171.0, 139.5, 129.0, 128.4, 127.4, 127.4, 57.2, 54.1, 26.1, 22.6, 12.1.**diasteroisomer 2** δ= 170.6, 139.4, 128.9, 128.4, 127.4, 56.9, 54.0, 25.9, 22.5, 12.1.

HRMS-(ESI+) for $C_{14}H_{22}NOS$ [M+H]$^+$: calculated 252.1422; found: 252.1424.

Spectral data for chiral aziridines

(*R*)-1-((*S*)-tert-butylsulfinyl)-2-(4,4,5,5-tetramethyl-1,3,2-dioxaborolan-2-yl)-2-(trimethylsilyl)-5-oxa-1-azaspiro[2.3]hexane ((S_S*,*R)-2)**

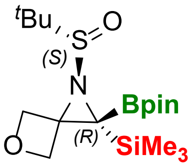

(S_S*,*R**)-2

The product was synthesized following the general procedure for the aziridination of *N-tert*-butanesulfinyl ketimines and purified by flash chromatography using as eluent a mixture of petroleum ether/ethyl acetate (100:5). The product **(S_S*,*R**)-2** was isolated as a white solid (41 mg, 0.11 mmol, 53%). *The product* ***2*** *was isolated as a white-yellowish solid (97 mg, 0.25 mmol, 63%).* M.p = 92.1 – 92.6 ºC.

$[\alpha]_D^{20}$: +110.8 (c = 1.0, CH_2Cl_2).

The enantiopurity was determined by HPLC analysis using a Daicel Chiralpak IC column, *n*-hexane/*i*-PrOH (92:08), flow rate 1.0 mL/min, wavelength = 210 nm, t_R = 7.27.

^{1}H NMR (400 MHz, $CDCl_3$) δ= 5.40 (dd, *J* = 8.2, 1.1 Hz, 1H), 4.96 (d, *J* = 1.0 Hz, 1H), 4.87 (dd, *J* = 7.3, 1.0 Hz, 1H), 4.76 (d, *J* = 1.1 Hz, 1H), 1.26 (s, 9H), 1.23 (s, 6H), 1.22 (s, 6H), 0.08 (s, 9H).

^{13}C NMR (100 MHz, $CDCl_3$) δ= 82.8, 79.2, 56.1, 49.3, 25.3, 25.0, 23.1, -1.3.

^{11}B NMR (129 MHz, $CDCl_3$) δ= 30.9.

HRMS-(ESI+) for $C_{17}H_{35}BNO_4SSi$ [M+H]$^+$: calculated 388.2144; found: 388.2141.

(*R*)-1-((*S*)-tert-butylsulfinyl)-2-(4,4,5,5-tetramethyl-1,3,2-dioxaborolan-2-yl)-2-(trimethylsilyl)-6-oxa-1-azaspiro[2.5]octane ((*S*$_S$*,*R)-4)**

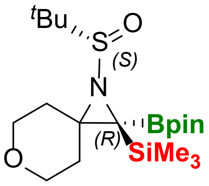

(*S*$_S$*,*R)-4**

The product was synthesized following the general procedure for the aziridination of *N*-*tert*-butanesulfinyl ketimines and purified by flash chromatography using as eluent a mixture of petroleum ether/ethyl acetate (100:4). The product **(*S*$_S$*,*R**)-4** was isolated as a white solid (36 mg, 0.09 mmol, 43%). *The product **4** was isolated as a white solid (31 mg, 0.07 mmol, 37%).* M.p = 115.4 – 116.3 ºC.

$[\alpha]_D^{20}$: +84.9 (c = 1.0, CH_2Cl_2).

The enantiopurity was determined by HPLC analysis using a Daicel Chiralpak ID column, *n*-hexane/*i*-PrOH (95:05), flow rate 1.0 mL/min, wavelength = 210 nm, t_R = 4.74.

^{1}H NMR (400 MHz, $CDCl_3$) δ= 3.94 – 3.83 (m, 2H), 3.82 – 3.74 (m, 2H), 2.34 – 2.18 (m, 1H), 2.12 – 1.99 (m, 1H), 1.91 – 1.80 (m, 1H), 1.69 – 1.62 (m, 1H), 1.25 (s, 9H), 1.23 (s, 12H), 0.18 (s, 9H).

^{13}C NMR (100 MHz, $CDCl_3$) δ= 83.0, 67.6, 66.8, 56.7, 25.4, 24.9, 23.1, 1.4.

^{11}B NMR (129 MHz, $CDCl_3$) δ= 31.2.

HRMS-(ESI+) for $C_{19}H_{39}BNO_4SSi$ [M+H]$^+$: calculated 416.2462; found: 416.2447.

(*R*)-1-((*S*)-tert-butylsulfinyl)-2-(4,4,5,5-tetramethyl-1,3,2-dioxaborolan-2-yl)-2-(trimethylsilyl)-1-azaspiro[2.5]octane ((*S*$_S$*,*R)-6)**

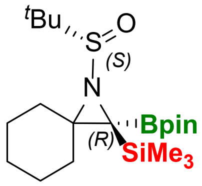

(*S*$_S$*,*R)-6**

The product was synthesized following the general procedure for the aziridination of *N*-*tert*-butanesulfinyl ketimines and purified by flash chromatography using as eluent a mixture of petroleum ether/ethyl acetate (100:4). The product **(*S*$_S$*,*R**)-6** was isolated as a white solid (43 mg, 0.10 mmol, 52%). *The product **6** was isolated as a white solid (39 mg, 0.09 mmol, 47%).*

M.p = 47.0 – 48.5 ºC.

$[\alpha]_D^{20}$: +74.5 (c = 1.0, CH_2Cl_2).

The enantiopurity was determined by HPLC analysis using a Daicel Chiralpak IF column, *n*-hexane/*i*-PrOH (90:10), flow rate 1.0 mL/min, wavelength = 210 nm, t_R = 3.59.

^{1}H NMR (400 MHz, $CDCl_3$) δ= 2.03 – 1.81 (m, 2H), 1.75 – 1.54 (m, 6H), 1.52 – 1.43 (m, 2H), 1.27 – 1.20 (m, 21H), 0.17 (s, 9H).

^{13}C NMR (100 MHz, $CDCl_3$) δ= 83.1, 56.4, 52.4, 35.2, 33.5, 25.9, 25.9, 25.5, 25.3, 25.0, 22.9, 1.3.

^{11}B NMR (129 MHz, $CDCl_3$) δ= 30.9.

HRMS-(ESI+) for $C_{20}H_{41}BNO_3SSi$ [M+H]$^+$: calculated 414.2669; found: 414.2654.

(2*S*,3*S*)-1-((*S*)-tert-butylsulfinyl)-3-phenyl-2-(4,4,5,5-tetramethyl-1,3,2-dioxaborolan-2-yl)-2-(trimethylsilyl)aziridine ((*S*$_S$*,*2S,*3S**)-8)**

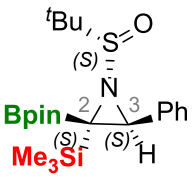

(*S*$_S$*,*2S,*3S**)-8**

The product was synthesized following the general procedure for the aziridination of *N*-*tert*-butanesulfinyl aldimines and purified by flash chromatography using as eluent a mixture of petroleum ether/ethyl acetate (100:5). The product **(*S*$_S$*,*2S**,*3S**)-8** was isolated as a white-yellowish solid (45 mg, 0.11 mmol, 54%). *The products **(S$_S$*,2S*,3S*)-8/(R$_S$*,2R*,3R*)-8** were isolated as a white-yellowish solid (36 mg, 0.09 mmol, 43%).* M.p = 122.3 ºC – 122.7 ºC.

[α]$_D$^{20}: -1.7 (c = 1.0, CH_2Cl_2).

The enantiopurity was determined by HPLC analysis using a Daicel Chiralpak IC column, *n*-hexane/*i*-PrOH (98:02), flow rate 1.0 mL/min, wavelength = 210 nm, t_R = 17.68.

^{1}H NMR (400 MHz, $CDCl_3$) δ= 7.35 – 7.29 (m, 2H), 7.30 – 7.22 (m, 2H), 7.23 – 7.14 (m, 1H), 3.81 (s, 1H), 1.25 (s, 9H), 1.11 (s, 6H), 0.96 (s, 6H), 0.29 (s, 9H).

^{13}C NMR (100 MHz, $CDCl_3$) δ= 138.9, 128.2, 127.3, 127.3, 83.6, 57.5, 41.0, 25.3, 25.1, 22.8, 0.3.

^{11}B NMR (129 MHz, $CDCl_3$) δ= 31.1.

HRMS-(ESI+) for $C_{21}H_{37}BNO_3SSi$ [M+H]$^+$: calculated 422.2351; found: 422.2340.

(2*S*,3*S*)-1-((*S*)-tert-butylsulfinyl)-3-(4-chlorophenyl)-2-(4,4,5,5-tetramethyl-1,3,2-dioxaborolan-2-yl)-2-(trimethylsilyl)aziridine ((*S*$_S$*,*2S,*3S**)-10)**

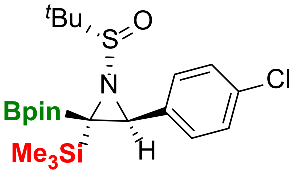

(*S*$_S$*,*2S,*3S**)-10**

The product was synthesized following the general procedure for the aziridination of *N*-*tert*-butanesulfinyl aldimines and purified by flash chromatography using as eluent a mixture of petroleum ether/ethyl acetate (100:5). The product **(*S*$_S$*,*2S**,*3S**)-10** was isolated as a white-yellowish solid (42

mg, 0.09 mmol, 46%). *The products* ***(S$_S$*,2S*,3S*)-10/(R$_S$*,2R*,3R*)-10*** *were isolated as a white-yellowish solid (36 mg, 0.08 mmol, 39%).* M.p = 104.9 - 141.1 ºC.

$[\alpha]_D^{20}$: -9.7 (c = 1.0, CH_2Cl_2).

The enantiopurity was determined by HPLC analysis using a Daicel Chiralpak IC column, *n*-hexane/*i*-PrOH (99:01), flow rate 1.0 mL/min, wavelength = 210 nm, t_R = 16.13.

^{1}H NMR (400 MHz, CDCl$_3$) δ= 7.29 – 7.22 (m, 4H), 3.76 (s, 1H), 1.24 (s, 9H), 1.12 (s, 6H), 0.99 (s, 6H), 0.29 (s, 9H).

^{13}C NMR (100 MHz, CDCl$_3$) δ= 137.6, 133.0, 128.8, 128.4, 83.7, 57.5, 40.6, 25.3, 25.1, 22.7, 0.2.

^{11}B NMR (129 MHz, CDCl$_3$) δ= 30.4.

HRMS-(ESI+) for $C_{21}H_{36}BClNO_3SSi$ [M+H]$^+$: calculated 456.1967; found: 456.1956.

(2*S*,3*S*)-1-((*S*)-tert-butylsulfinyl)-2-(4,4,5,5-tetramethyl-1,3,2-dioxaborolan-2-yl)-3-(p-tolyl)-2-(trimethylsilyl)aziridine ((*S$_S$*,2S*,3S)-12)**

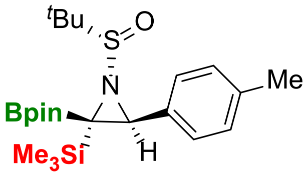

(*S$_S$*,2S*,3S**)-12

The product was synthesized following the general procedure for the aziridination of *N-tert*-butanesulfinyl aldimines and purified by flash chromatography using as eluent a mixture of petroleum ether/ethyl acetate (100:5). The product **(*S$_S$*,2S*,3S**)-12** was isolated as a white-yellowish solid (30 mg, 0.07 mmol, 34%). *The products* ***(S$_S$*,2S*,3S*)-12/(R$_S$*,2 R*,3R*)-12*** *were isolated as a white-yellowish solid (28 mg, 0.06 mmol, 32%).* M.p = 99.7 - 101.9 ºC.

$[\alpha]_D^{20}$: -11.2 (c = 1.0, CH_2Cl_2).

The enantiopurity was determined by HPLC analysis using a Daicel Chiralpak IC column, *n*-hexane/*i*-PrOH (95:05), flow rate 1.0 mL/min, wavelength = 210 nm, t_R = 9.07.

^{1}H NMR (400 MHz, CDCl$_3$) δ= 7.23 – 7.16 (m, 2H), 7.10 – 7.03 (m, 2H), 3.77 (s, 1H), 2.30 (s, 3H), 1.24 (s, 9H), 1.13 (s, 6H), 0.99 (s, 6H), 0.29 (s, 9H).

^{13}C NMR (100 MHz, CDCl$_3$) δ= 136.5, 135.5, 128.6, 126.9, 83.3, 57.1, 40.5, 25.0, 24.8, 22.5, 21.0, 0.0.

^{11}B NMR (129 MHz, CDCl$_3$) δ= 31.3.

HRMS-(ESI+) for $C_{22}H_{39}BNO_3SSi$ [M+H]$^+$: calculated 436.2513; found: 436.2499.

(2*S*,3*S*)-1-((*S*)-tert-butylsulfinyl)-3-(2-fluorophenyl)-2-(4,4,5,5-tetramethyl-1,3,2-dioxaborolan-2-yl)-2-(trimethylsilyl)aziridine ((*S*$_S$*,*2S*,3S)-14)**

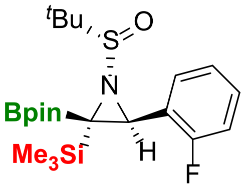

(*S*$_S$*,*2S*,3S)-14**

The product was synthesized following the general procedure for the aziridination of *N-tert*-butanesulfinyl aldimines and purified by flash chromatography using as eluent a mixture of petroleum ether/ethyl acetate (100:5). The product **(*S*$_S$*,*2S*,3S**)-14** was isolated as a white-yellowish solid (45 mg, 0.1 mmol, 51%). *The products **(S$_S$*,2S*,3S*)-14/(R$_S$*,2R*,3R*)-14** were isolated as a white-yellowish solid (43 mg, 0.1 mmol, 49%).* M.p = 112.8 - 114.2 ºC.

$[\alpha]_D^{20}$: -11.6 (c = 1.0, CH_2Cl_2).

The enantiopurity was determined by HPLC analysis using a Daicel Chiralpak IF column, *n*-hexane/*i*-PrOH (80:20), flow rate 1.0 mL/min, wavelength = 210 nm, t_R = 4.19.

^{1}H NMR (400 MHz, $CDCl_3$) δ= 7.30 (td, *J* = 7.5, 1.8 Hz, 1H), 7.21 – 7.13 (m, 1H), 7.05 – 6.94 (m, 2H), 4.03 (s, 1H), 1.29 (s, 9H), 1.06 (s, 6H), 0.91 (s, 6H), 0.30 (s, 9H).

^{13}C NMR (100 MHz, $CDCl_3$) δ= 162.4 (d, $^1J_{C-F}$ = 247.0 Hz), 128.5 (d, $^3J_{C-F}$ = 8.0 Hz), 128.0 (d, $^3J_{C-F}$ = 3.8 Hz), 126.6 (d, $^2J_{C-F}$ = 13.4 Hz), 124.6 (d, $^4J_{C-F}$ = 3.6 Hz), 115.0 (d, $^2J_{C-F}$ = 20.7 Hz), 83.5, 57.5, 36.3, 29.9, 25.1, 24.9, 22.7, 0.1

^{11}B NMR (129 MHz, $CDCl_3$) δ= 31.1.

^{19}F NMR (377 MHz, $CDCl_3$) δ= -119.1.

HRMS-(ESI+) for $C_{21}H_{36}BFNO_3SSi$ [M+H]$^+$: calculated 440.2262; found: 440.2253.

(2*S*,3*S*)-1-((*S*)-tert-butylsulfinyl)-2-(4,4,5,5-tetramethyl-1,3,2-dioxaborolan-2-yl)-3-(2-(trifluoromethyl)phenyl)-2-(trimethylsilyl)aziridine ((*S*$_S$*,*2S*,3S)-16)**

Major stereoisomer

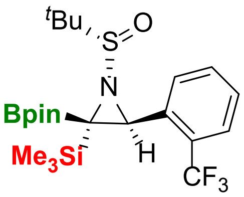

(*S*$_S$*,*2S*,3S)-16**

The product was synthesized following the general procedure for the aziridination of *N-tert*-butanesulfinyl aldimines and purified by flash chromatography using as eluent a mixture of petroleum ether/ethyl acetate (100:5). The product **(*S*$_S$*,*2S*,3S**)-16** was isolated as a white-yellowish solid (24 mg, 0.05 mmol, 25%). *The products **(S$_S$*,2S*,3S*)-16/(R$_S$*,2R*,3R*)-16** were isolated as a white-yellowish solid (32 mg, 0.07 mmol, 33%).* M.p = 126.1 - 127.0 ºC.

$[\alpha]_D^{20}$: -24.5 (c = 1.0, CH_2Cl_2).

The enantiopurity was determined by HPLC analysis using a Daicel Chiralpak IC column, *n*-hexane/*i*-PrOH (90:10), flow rate 1.0 mL/min, wavelength = 210 nm, t_R = 4.97.

^{1}H NMR (400 MHz, CDCl$_3$) δ= 7.63 – 7.55 (m, 2H), 7.43 (ddd, *J* = 8.4, 7.4, 1.5 Hz, 1H), 7.30 (tt, *J* = 7.7, 1.2 Hz, 1H), 4.16 (q, *J* = 1.9 Hz, 1H), 1.30 (s, 9H), 1.05 (s, 6H), 0.88 (s, 6H), 0.28 (s, 9H).

^{13}C NMR (100 MHz, CDCl$_3$) δ= 131.5,129.2, 127.8, 127.9, 125.7 (q, $^{3}J_{C-F}$= 5.8 Hz), 122.8 83.4, 57.6, 38.7, 25.1, 24.9, 22.8, -0.1

^{11}B NMR (129 MHz, CDCl$_3$) δ= 30.6.

^{19}F NMR (377 MHz, CDCl$_3$) δ= -59.5.

HRMS-(ESI+) for $C_{22}H_{36}BF_3NO_3SSi$ [M+H]$^+$: calculated 490.2230; found: 490.2222.

(2*S*,3*R*)-1-((*S*)-tert-butylsulfinyl)-2-(4,4,5,5-tetramethyl-1,3,2-dioxaborolan-2-yl)-3-(2-(trifluoromethyl)phenyl)-2-(trimethylsilyl)aziridine ((*S*$_S$*,2*S,3*R**)-16)**

Minor stereoisomer

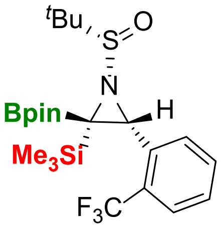

(*S*$_S$*,2*S,3*R**)-16**

The product was synthesized following the general procedure for the aziridination of *N-tert*-butanesulfinyl aldimines and purified by flash chromatography using as eluent a mixture of petroleum ether/ethyl acetate (100:5). The product **(*S*$_S$*,2*S**,3*R**)-16** was isolated as a white-yellowish solid (5 mg, 0.01 mmol, 5%). M.p = 126.1 - 127.0 ºC.

^{1}H NMR (400 MHz, CDCl$_3$) δ= 7.66 – 7.59 (m, 2H), 7.48 – 7.41 (m, 1H), 7.39 – 7.32 (m, 1H), 4.00 (d, *J* = 1.9 Hz, 1H), 1.35 (s, 9H), 1.29 (s, 12H), -0.18 (s, 9H).

^{13}C NMR (100 MHz, CDCl$_3$) δ= 129.5,129.2, 127.8, 127.9, 125.7 (q, $^{3}J_{C-F}$= 5.8 Hz), 122.8 83.4, 57.6, 38.7, 25.1, 24.9, 22.8, -0.1

^{11}B NMR (129 MHz, CDCl$_3$) δ= 29.8.

^{19}F NMR (377 MHz, CDCl$_3$) δ= -59.5.

HRMS-(ESI+) for $C_{22}H_{36}BF_3NO_3SSi$ [M+H]$^+$: calculated 490.2230; found: 490.2222.

2-((2*S*,3*S*)-1-((*S*)-tert-butylsulfinyl)-3-(4,4,5,5-tetramethyl-1,3,2-dioxaborolan-2-yl)-3-(trimethylsilyl)aziridin-2-yl)pyridine ((*S*$_S$*,2*S,3*S**)-18)**

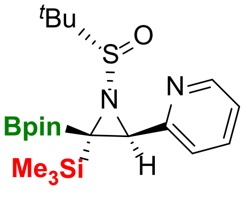

(*S*$_S$*,2*S,3*S**)-18**

The product was synthesized following the general procedure for the aziridination of *N-tert*-butanesulfinyl aldimines and purified by flash chromatography using as eluent a mixture of petroleum ether/ethyl acetate (70:30). The product **(*S*$_S$*,2*S**,3*S**)-18** was isolated as a white-yellowish solid (35 mg, 0.08 mmol, 41%). *The products **(S$_S$*,2S*,3S*)-18/(R$_S$*,2R*,3R*)-18** were isolated as a white-yellowish solid (29 mg, 0.07 mmol, 34%)*. M.p = 138 ºC decomposes.

$[\alpha]_D^{20}$: -6.6 (c = 1.0, CH_2Cl_2).

The enantiopurity was determined by HPLC analysis using a Daicel Chiralpak IC column, *n*-hexane/*i*-PrOH (80:20), flow rate 1.0 mL/min, wavelength = 210 nm, t_R = 12.77.

^{1}H NMR (400 MHz, CDCl$_3$) δ= 8.51 (ddd, *J* = 5.0, 1.8, 0.9 Hz, 1H), 7.60 (td, *J* = 7.7, 1.8 Hz, 1H), 7.34 (dt, *J* = 7.9, 1.1 Hz, 1H), 7.14 (ddd, *J* = 7.5, 4.9, 1.2 Hz, 1H), 3.90 (s, 1H), 1.20 (s, 9H), 1.19 (s, 6H), 1.10 (s, 6H), 0.30 (s, 9H).

^{13}C NMR (100 MHz, CDCl$_3$) δ= 158.4, 148.7, 136.5, 122.4, 122.2, 83.3, 57.6, 42.6, 25.5, 25.3, 22.6, 0.3.

^{11}B NMR (129 MHz, CDCl$_3$) δ= 29.4.

HRMS-(ESI+) for $C_{20}H_{36}BN_2O_3SSi$ [M+H]$^+$: calculated 423.2303; found: 423.2304.

3-((2*S*,3*S*)-1-((*S*)-tert-butylsulfinyl)-3-(4,4,5,5-tetramethyl-1,3,2-dioxaborolan-2-yl)-3-(trimethylsilyl)aziridin-2-yl)pyridine ((*S*$_S$*,2*S,3*S**)-20)**

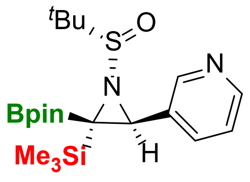

(*S*$_S$*,2*S,3*S**)-20**

The product was synthesized following the general procedure for the aziridination of *N-tert*-butanesulfinyl aldimines and purified by flash chromatography using as eluent a mixture of petroleum ether/ethyl acetate (70:30). The product **(*S*$_S$*,2*S**,3*S**)-20** was isolated as a white-yellowish solid (34 mg, 0.08 mmol, 40%). *The products **(S$_S$*,2S*,3S*)-20/(R$_S$*,2R*,3R*)-20** were isolated as a white-yellowish solid (38 mg, 0.09 mmol, 45%).* M.p = 139.2 - 140.4 ºC.

$[\alpha]_D^{20}$: -4.1 (c = 1.0, CH_2Cl_2).

The enantiopurity was determined by HPLC analysis using a Daicel Chiralpak IA column, *n*-hexane/*i*-PrOH (80:20), flow rate 1.0 mL/min, wavelength = 210 nm, t_R = 5.97.

^{1}H NMR (400 MHz, CDCl$_3$) δ= 8.60 (s, 1H), 8.47 (s, 1H), 7.63 (dd, *J* = 7.9, 1.7 Hz, 1H), 7.20 (s, 1H), 3.80 (s, 1H), 1.25 (s, 9H), 1.10 (s, 6H), 0.97 (s, 6H), 0.29 (s, 9H).

^{13}C NMR (100 MHz, CDCl$_3$) δ= 148.9, 148.4, 135.0, 122.9, 83.7, 57.4, 38.9, 25.0, 24.9, 22.5, 0.0.

^{11}B NMR (129 MHz, CDCl$_3$) δ= 30.8.

HRMS-(ESI+) for $C_{20}H_{36}BN_2O_3SSi$ [M+H]$^+$: calculated 423.2309; found: 423.2295.

4-((2*S*,3*S*)-1-((*S*)-tert-butylsulfinyl)-3-(4,4,5,5-tetramethyl-1,3,2-dioxaborolan-2-yl)-3-(trimethylsilyl)aziridin-2-yl)pyrimidine ((*S*$_S$*,2*S,3*S**)-22)**

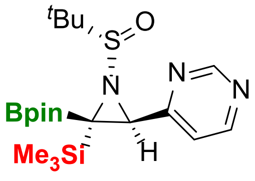

(*S*$_S$*,2*S,3*S**)-22**

The product was synthesized following the general procedure for the aziridination of *N-tert*-butanesulfinyl aldimines and purified by flash chromatography using as eluent a mixture of petroleum ether/ethyl acetate (100:100). The product **(*S*$_S$*,2*S**,3*S**)-22** was isolated as an orangish solid (17 mg, 0.04 mmol, 20%). *The*

products ***(S*** $_S$ ****,2S*,3S*)-22/(R*** $_S$ ****,2R*,3R*)-22*** *were isolated as an orangish solid (10 mg, 0.02 mmol, 12%)*. M.p = 84.7 - 85.4 °C.

$[\alpha]_D^{20}$: -3.5 (c = 1.0, CH_2Cl_2).

The enantiopurity was determined by HPLC analysis using a Daicel Chiralpak IF column, *n*-hexane/*i*-PrOH (80:20), flow rate 1.0 mL/min, wavelength = 210 nm, t_R = 5.79.

^{1}H NMR (400 MHz, $CDCl_3$) δ= 9.10 (d, *J* = 1.4 Hz, 1H), 8.61 (d, *J* = 5.1 Hz, 1H), 7.34 (dd, *J* = 5.2, 1.4 Hz, 1H), 3.83 (s, 1H), 1.22 (s, 9H), 1.17 (s, 6H), 1.08 (s, 6H), 0.30 (s, 9H).

^{13}C NMR (100MHz, $CDCl_3$) δ=167.4, 158.4, 156.7, 119.5, 83.9, 57.8, 41.7, 25.2, 22.5, 0.0.

^{11}B NMR (129 MHz, $CDCl_3$) δ= 30.7.

HRMS-(ESI+) for $C_{19}H_{35}BN_3O_3SSi$ [M+H]$^+$: calculated 424.2261; found: 424.2253.

(2*S*,3*S*)-1-((*S*)-tert-butylsulfinyl)-3-(naphthalen-1-yl)-2-(4,4,5,5-tetramethyl-1,3,2-dioxaborolan-2-yl)-2-(trimethylsilyl)aziridine ((*S*$_S$*,2*S,3*S**)-24)**

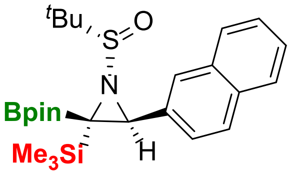

(*S*$_S$*,2*S,3*S**)-24**

The product synthesized following the general procedure for the aziridination of *N*-*tert*-butanesulfinyl aldimines and purified by flash chromatography using as eluent a mixture of petroleum ether/ethyl acetate (100:5). The product **(*S*$_S$*,2*S**,3*S**)-24** was isolated as a white-yellowish solid (39 mg, 0.08 mmol, 42%). *The products* ***(S*** $_S$ ****,2S*,3S*)-24/(R*** $_S$ ****,2R*,3R*)-24*** *were isolated as a white-yellowish solid (29 mg, 0.06 mmol, 31%).* M.p = 122.5 - 123.1 °C.

$[\alpha]_D^{20}$: -30.9 (c = 1.0, CH_2Cl_2).

The enantiopurity was determined by HPLC analysis using a Daicel Chiralpak IC column, *n*-hexane/*i*-PrOH (90:10), flow rate 1.0 mL/min, wavelength = 210 nm, t_R = 6.27.

^{1}H NMR (400 MHz, $CDCl_3$) δ= 7.81 – 7.73 (m, 4H), 7.47 – 7.39 (m, 3H), 3.97 (s, 1H), 1.27 (s, 9H), 1.10 (s, 6H), 0.88 (s, 6H), 0.33 (s, 9H).

^{13}C NMR (100 MHz, $CDCl_3$) δ= 136.6, 133.3, 133.0, 127.9, 127.9, 127.8, 126.2, 126.1, 125.7, 125.4, 83.6, 57.5, 41.1, 25.3, 25.1, 22.9, 0.4.

^{11}B NMR (129 MHz, $CDCl_3$) δ= 29.8

HRMS-(ESI+) for $C_{22}H_{41}BO_6SSi$ [M+H]$^+$: calculated 472.2481; found: 472.2502.

4-((2*S*,3*S*)-1-((*S*)-tert-butylsulfinyl)-3-(4,4,5,5-tetramethyl-1,3,2-dioxaborolan-2-yl)-3-(trimethylsilyl)aziridin-2-yl)quinoline ((*S*$_S$*,2*S,3*S**)-26)**

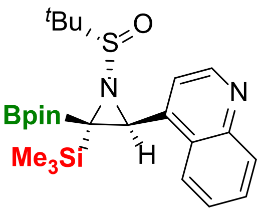

(*S*$_S$*,2*S**,3*S**)-26

The product synthesized following the general procedure for the aziridination of *N-tert*-butanesulfinyl aldimines and purified by flash chromatography using as eluent a mixture of petroleum ether/ethyl acetate (70:30). The product **(*S*$_S$*,2*S**,3*S**)-26** was isolated as a white-yellowish solid (50 mg, 0.11 mmol, 53%). *The products **(S$_S$*,2S*,3S*)-26/(R$_S$*,2R*,3R*)-26** were isolated as a white-yellowish solid (10 mg, 0.02 mmol, 11%)*. M.p = 134.4 - 136.9 ºC.

$[\alpha]_D^{20}$: -24.6 (c = 1.0, CH_2Cl_2).

The enantiopurity was determined by HPLC analysis using a Daicel Chiralpak IC column, *n*-hexane/*i*-PrOH (70:30), flow rate 1.0 mL/min, wavelength = 210 nm, t_R = 7.20.

^{1}H NMR (400 MHz, $CDCl_3$) δ= 8.83 (s, 1H), 8.22 – 8.04 (m, 2H), 7.73 (ddd, *J* = 8.4, 6.9, 1.4 Hz, 1H), 7.61 (ddd, *J* = 8.1, 6.9, 1.1 Hz, 1H), 7.44 (d, *J* = 4.3 Hz, 1H), 4.41 (s, 1H) 1.34 (s, 9H), 0.96 (s, 6H), 0.77 (s, 6H), 0.38 (s, 9H).

^{13}C NMR (100 MHz, $CDCl_3$) δ= 149.7, 147.6, 144.8, 129.8, 129.1, 126.5, 123.0, 118.0, 83.3, 57.4, 38.0, 24.6, 24.4, 22.4, 0.0.

^{11}B NMR (129 MHz, $CDCl_3$) δ= 30.9.

HRMS-(ESI+) for $C_{24}H_{38}BN_2O_3SSi$ $[M+H]^+$: calculated 473.2465; found: 473.2448.

(2*R*,3*S*)-1-((*S*)-tert-butylsulfinyl)-3-isobutyl-2-(4,4,5,5-tetramethyl-1,3,2-dioxaborolan-2-yl)-2-(trimethylsilyl)aziridine ((*S*$_S$*,2*R,3*S**)-28)**

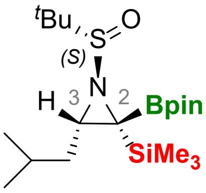

(*S*$_S$*,2*R**,3*S**)-28

The product synthesized following the general procedure for the aziridination of *N-tert*-butanesulfinyl aldimines and purified by flash chromatography using as eluent a mixture of petroleum ether/ethyl acetate (100:2). The product **(*S*$_S$*,2*R**,3*S**)-28** was isolated as a white-yellowish solid (42 mg, 0.11 mmol, 52%). *The products **(S$_S$*,2R*,3S*)-28/(R$_S$*,2S*,3R*)-28** were isolated as a white-yellowish solid (49 mg, 0.12 mmol, 61%)*. M.p = 66.3 - 67.1 ºC.

[α]D^{20}: +72.9 (c = 1.0, CH_2Cl_2).

^{1}H NMR (400 MHz, $CDCl_3$) δ= 2.34 (dd, *J* = 9.2, 3.4 Hz, 1H), 1.86 – 1.72 (m, 1H), 1.68 (ddd, *J* = 13.8, 6.0, 3.5 Hz, 1H), 1.58 – 1.47 (m, 1H), 1.26 – 1.22 (m, 21H), 0.95 (t, *J* = 6.7 Hz, 6H), 0.16 (s, 9H).

^{13}C NMR (100 MHz, $CDCl_3$) δ= 82.7, 56.1, 42.1, 39.4, 28.2, 25.5, 24.8, 23.2, 23.1, 22.8, 0.7.

^{11}B NMR (129 MHz, $CDCl_3$) δ= 29.6.

HRMS-(ESI+) for $C_{19}H_{41}BNO_3SSi$ [M+H]$^+$: calculated 402.2669; found: 402.2657.

(2*R*,3*S*)-1-((*S*)-tert-butylsulfinyl)-3-(4-chlorobutyl)-2-(4,4,5,5-tetramethyl-1,3,2-dioxaborolan-2-yl)-2-(trimethylsilyl)aziridine ((*S*$_S$*,2*R,3*S**)-30)**

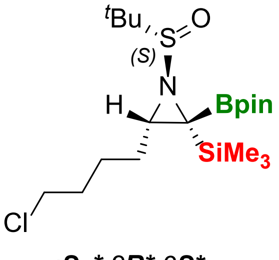

(*S*$_S$*,2*R**,3*S**)-30

The product was synthesized following the general procedure for the aziridination of *N-tert*-butanesulfinyl aldimines and purified by flash chromatography using as eluent a mixture of petroleum ether/ethyl acetate (100:3). The product **(*S*$_S$*,2*R**,3*S**)-30** was isolated as a colorless oil (46 mg, 0.11 mmol, 53%). *The products **(S$_S$*,2R*,3S*)-30/(R$_S$*,2S*,3R*)-30** were isolated as a colorless oil (49 mg, 0.11 mmol, 56%).*

$[\alpha]_D^{20}$: +63.3 (c = 1.0, CH_2Cl_2).

The enantiopurity was determined by HPLC analysis using a Daicel Chiralpak ODH column, *n*-hexane/*i*-PrOH (99:01), flow rate 0.8 mL/min, wavelength = 210 nm, t_R = 4.88.

^{1}H NMR (400 MHz, $CDCl_3$) δ= 3.54 (t, *J* = 6.7 Hz, 2H), 2.28 (dd, *J* = 9.3, 3.4 Hz, 1H), 1.91 – 1.75 (m, 3H), 1.68 – 1.59 (m, 2H), 1.43 – 1.33 (m, 1H), 1.26 – 1.22 (m, 21H), 0.16 (s, 9H).

^{13}C NMR (100 MHz, $CDCl_3$) δ= 82.8, 56.3, 44.9, 42.8, 32.3, 29.7, 25.5, 25.4, 24.8, 23.2, 0.6.

^{11}B NMR (129 MHz, $CDCl_3$) δ= 29.9.

HRMS-(ESI+) for $C_{19}H_{40}BClNO_3SSi$ [M+H]$^+$: calculated 436.2280; found: 436.2270.

(2*R*,3*S*)-1-((*S*)-tert-butylsulfinyl)-3-cyclohexyl-2-(4,4,5,5-tetramethyl-1,3,2-dioxaborolan-2-yl)-2-(trimethylsilyl)azirdine ((*S*$_S$*,2*R,3*S**)-32)**

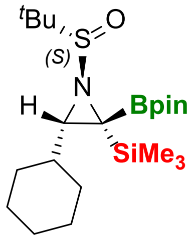

(*S*$_S$*,2*R**,3*S**)-32

The product was synthesized following the general procedure for the aziridination of *N-tert*-butanesulfinyl aldimines and purified by flash chromatography using as eluent a mixture of petroleum ether/ethyl acetate (100:4). The product **(*S*$_S$*,2*R**,3*S**)-32** was isolated as a white-yellowish solid (36 mg, 0.08 mmol, 42%). *The products **(S$_S$*,2R*,3S*)-32/(R$_S$*,2S*,3R*)-32** were isolated as a white-yellowish solid (31 mg, 0.08 mmol, 36%)*. M.p = 79.9 - 81.4 ºC.

[α]D^{20}: +92.4 (c = 1.0, CH_2Cl_2).

The enantiopurity was determined by HPLC analysis using a Daicel Chiralpak ODH column, *n*-hexane/*i*-PrOH (99:01), flow rate 0.8 mL/min, wavelength = 210 nm, t_R = 4.5.

^{1}H NMR (400 MHz, $CDCl_3$) δ= 2.10 (d, *J* = 8.7 Hz, 1H), 1.89 – 1.81 (m, 1H), 1.79 – 1.69 (m, 3H), 1.69 – 1.60 (m, 2H), 1.24 – 1.20 (m, 21H), 1.20 – 1.15 (m, 4H), 0.15 (s, 9H).

^{13}C NMR (100 MHz, $CDCl_3$) δ= 82.8, 56.1, 48.6, 38.9, 31.1, 31.1, 26.6, 26.0, 25.9, 25.5, 24.8, 23.2, 0.6.

^{11}B NMR (129 MHz, $CDCl_3$) δ= 31.4

HRMS-(ESI+) for $C_{21}H_{43}BNO_3SSi$ [M+H]$^+$: calculated 428.2826; found: 428.2809.

(2*R*,3*S*)-1-((*S*)-tert-butylsulfinyl)-3-((*S*)-2,6-dimethylhept-5-en-1-yl)-2-(4,4,5,5-tetramethyl-1,3,2-dioxaborolan-2-yl)-2-(trimethylsilyl)aziridine ((*S*$_S$*,2*R,3*S**,*S*)-34)**

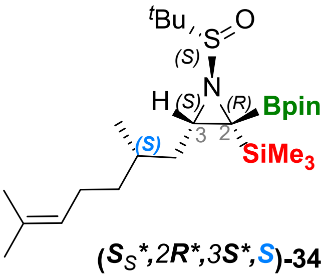

(*S*$_S$*,2*R,3*S**,*S*)-34**

The product was synthesized following the general procedure for the aziridination of *N-tert*-butanesulfinyl aldimines and purified by flash chromatography using as eluent a mixture of petroleum ether/ethyl acetate (100:3). The product **(*S*$_S$*,2*R**,3*S**,*S*)-34** was isolated as a bright yellow oil (56 mg, 0.12 mmol, 60%). *The products **(S$_S$*,2R*,3S*,S)-34/(R$_S$*,2S*,3R*,S)-34** were isolated as a bright yellow oil (63 mg, 0.14 mmol, 68%).*

[α]D^{20}: +74.1 (c = 1.0, CH_2Cl_2).

^{1}H NMR (400 MHz, $CDCl_3$) δ= 5.09 (tdt, *J* = 7.0, 2.8, 1.4 Hz, 1H), 2.33 (dd, *J* = 9.2, 3.3 Hz, 1H), 2.05 – 1.90 (m, 2H), 1.78 (ddd, *J* = 13.8, 5.0, 3.3 Hz, 1H), 1.68 (s, 3H), 1.60 (s, 3H), 1.42 – 1.35 (m, 1H), 1.29 – 1.13 (m, 24H), 0.97 (d, *J* = 6.7 Hz, 3H), 0.16 (s, 9H).

^{13}C NMR (100 MHz, $CDCl_3$) δ= 131.4, 124.9, 82.7, 56.1, 42.2, 37.7, 37.6, 32.7, 25.9, 25.7, 25.5, 24.8, 23.2, 19.8, 17.8, 0.7.

^{11}B NMR (129 MHz, $CDCl_3$) δ= 30.9.

HRMS-(ESI+) for $C_{24}H_{49}BNO_3SSi$ [M+H]$^+$: calculated 470.3295; found: 470.3287.

(2*R*,3*S*)-1-((*S*)-tert-butylsulfinyl)-2-methyl-2-(2-phenylpropyl)-3-(4,4,5,5-tetramethyl-1,3,2-dioxaborolan-2-yl)-3-(trimethylsilyl)aziridine ((*S*$_S$*,2*R,3*S**,*S*)-36)**

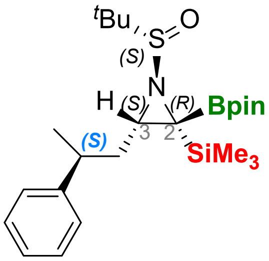

(*S*$_S$*,2*R,3*S**,*S*)-36**

The product was synthesized following the general procedure for the aziridination of *N-tert*-butanesulfinyl aldimines and purified by flash chromatography using as eluent a mixture of petroleum ether/ethyl acetate (100:3). The product **(*S*$_S$*,2*R**,3*S**,*S*)-36** was isolated as a white-yellowish solid (25 mg, 0.05 mmol, 28%). *The products **(S$_S$*,2R*,3S*,S)-36/(R$_S$*,2S*,3R*,S)-36** were isolated as a white-yellowish solid (23 mg, 0.05 mmol, 27%).* M.p = 61.4 - 62.2 ºC.

[α]$_D$^{20}: +53.3 (c = 1.0, CH_2Cl_2).

The enantiopurity was determined by HPLC analysis using a Daicel Chiralpak ODH column, *n*-hexane/*i*-PrOH (99:01), flow rate 0.8 mL/min, wavelength = 210 nm, t_R = 4.75.

^{1}H NMR (400 MHz, $CDCl_3$) δ= 7.31 – 7.26 (m, 2H), 7.23 – 7.13 (m, 3H), 3.00 – 2.87 (m, 1H), 2.30 (dd, *J* = 9.3, 3.2 Hz, 1H), 2.14 – 2.02 (m, 1H), 1.65 – 1.54 (m, 1H), 1.32 (d, *J* = 7.0 Hz, 3H), 1.25 (s, 9H), 1.23 (s, 6H), 1.22 (s, 6H), 0.16 (s, 9H).

^{13}C NMR (100 MHz, $CDCl_3$) δ= 147.4, 128.6, 127.1, 126.2, 82.7, 56.2, 42.1, 39.6, 39.3, 29.8, 25.5, 24.8, 23.2, 22.0, 0.7.

^{11}B NMR (129 MHz, $CDCl_3$) δ= 30.8.

HRMS-(ESI+) for $C_{24}H_{43}BNO_3SSi$ $[M+H]^+$: calculated 464.2826; found: 464.2814.

(2*R*,3*S*)-1-((*S*)-tert-butylsulfinyl)-2-methyl-2-(2-phenylpropyl)-3-(4,4,5,5-tetramethyl-1,3,2-dioxaborolan-2-yl)-3-(trimethylsilyl)aziridine ((*S*$_S$*,2*R,3*S**,*R*)-36)**

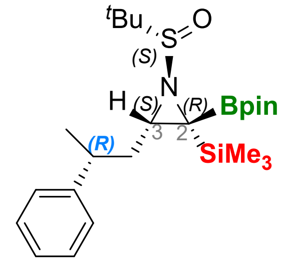

(*S*$_S$*,2*R**,3*S**,*R*)-36

The product was synthesized following the general procedure for the aziridination of *N*-*tert*-butanesulfinyl aldimines and purified by flash chromatography using as eluent a mixture of petroleum ether/ethyl acetate (100:3). The product **(*S*$_S$*,2*R**,3*S**,*R*)-36** was isolated as a white-yellowish solid (26 mg, 0.06 mmol, 28%). *The products **(S$_S$*,2R*,3S*,R)-36/(R$_S$*,2S*,3R*,R)-36** were isolated as a white-yellowish solid (27 mg, 0.06 mmol, 29%).* M.p = 61.4 - 62.2 ºC.

$[\alpha]_D^{20}$: +56.5 (c = 1.0, CH_2Cl_2).

The enantiopurity was determined by HPLC analysis using a Daicel Chiralpak ODH column, *n*-hexane/*i*-PrOH (99:01), flow rate 0.8 mL/min, wavelength = 210 nm, t_R = 5.83.

^{1}H NMR (400 MHz, $CDCl_3$) δ= 7.28 – 7.21 (m, 2H), 7.20 – 7.12 (m, 3H), 2.96 – 2.83 (m, 1H), 2.28 (dd, *J* = 9.1, 3.4 Hz, 1H), 2.09 – 1.94 (m, 1H), 1.78 – 1.66 (m, 1H), 1.29 (d, *J* = 7.0 Hz, 3H), 1.22 (s, 6H), 1.22 (s, 6H), 1.07 (s, 9H), 0.16 (s, 9H).

^{13}C NMR (100 MHz, $CDCl_3$) δ= 146.8, 128.5, 127.5, 126.1, 82.6, 55.8, 42.3, 39.9, 39.0, 29.9, 25.5, 24.8, 23.1, 22.7, 0.7.

^{11}B NMR (129 MHz, $CDCl_3$) δ= 29.6.

HRMS-(ESI+) for $C_{24}H_{43}BNO_3SSi$ $[M+H]^+$: calculated 464.2826; found: 464.2814.

((*S*)-sec-butyl)-1-((*S*)-tert-butylsulfinyl)-2-(4,4,5,5-tetramethyl-1,3,2-dioxaborolan-2-yl)-2-(trimethylsilyl)aziridine ((*S*$_S$*,2*R,3*S**,*R*)-38)/((*S*$_S$*,2*R**,3*S**,*R*)-38)**

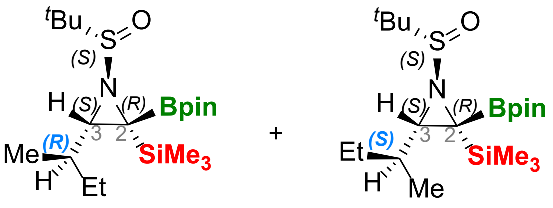

(*S*$_S$*,2*R**,3*S**,*R*)-38 + (*S*$_S$*,2*R**,3*S**,*S*)-38

The product was synthesized following the general procedure for the aziridination of *N*-*tert*-butanesulfinyl aldimines and purified by flash chromatography using as eluent a mixture of petroleum ether/ethyl acetate (100:3). The products **(*S*$_S$*,2*R**,3*S**,*R*)-38)/((*S*$_S$*,2*R**,3*S**,*S*)-38** were isolated as a colorless oil and as a mixture of diasteroisomers (6:4) (25 mg, 0.06 mmol, 31%).

^{1}H NMR (400 MHz, CDCl$_3$) diasteroisomer 1 δ= 2.11 (d, *J* = 9.2 Hz, 1H), 1.60 – 1.50 (m, 1H), 1.26 – 1.20 (m, 23H), 1.01 – 0.97 (m, 3H), 0.94 – 0.87 (m, 3H), 0.15 (s, 9H). **diasteroisomer 2** δ= 2.08 (d, *J* = 9.5 Hz, 1H), 1.60 – 1.50 (m, 1H), 1.26 – 1.20 (m, 23H), 1.01 – 0.97 (m, 3H), 0.94 – 0.87 (m, 3H), 0.16 (s, 9H).

^{13}C NMR (100 MHz, CDCl$_3$) diasteroisomer 1δ= 82.8, 56.1, 49.4, 35.6, 28.0, 25.5, 24.8, 23.2, 17.3, 11.4, 0.6. **diasteroisomer 2** δ= 82.7, 56.1, 48.8, 35.5, 28.3, 25.5, 24.8, 23.3, 17.5, 11.6, 0.7.

^{11}B NMR (129 MHz, CDCl$_3$) δ= 30.9.

HRMS-(ESI+) for $C_{19}H_{41}BNO_3SSi$ [M+H]$^+$: calculated 402.2669; found: 402.2670.

(2*R*,3*S*)-1-((*S*)-tert-butylsulfinyl)-3-((*S*)-1-phenylethyl)-2-(4,4,5,5-tetramethyl-1,3,2-dioxaborolan-2-yl)-2-(trimethylsilyl)aziridine ((*S*$_S$*,2*R,3*S**,*R*)-40)**

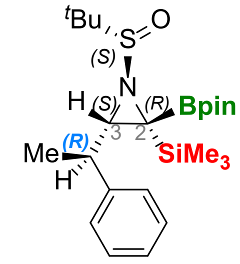

(*S*$_S$*,2*R**,3*S**,*R*)-40

The product was synthesized following the general procedure for the aziridination of *N*-*tert*-butanesulfinyl aldimines and purified by flash chromatography using as eluent a mixture of petroleum ether/ethyl acetate (100:3). The product **(*S*$_S$*,2*R**,3*S**,*R*)-40** was isolated as a white-yellowish solid (29 mg, 0.07 mmol, 32%). *The products **(S$_S$*,2R*,3S*,R)-40/(R$_S$*,2S*,3R*,R)-40** were isolated as a white-yellowish solid (27 mg, 0.06 mmol, 30%).* M.p= 98.3- 99.5 ºC. $[\alpha]_D^{20}$: 93.9 (c = 1.0, CH_2Cl_2).

The enantiopurity was determined by HPLC analysis using a Daicel Chiralpak IF column, *n*-hexane/*i*-PrOH (99:01), flow rate 0.8 mL/min, wavelength = 210 nm, t_R = 5.6.

^{1}H NMR (400 MHz, CDCl$_3$) δ= 7.43 – 7.35 (m, 2H), 7.33 – 7.24 (m, 2H), 7.22 – 7.14 (m, 1H), 2.80 (d, *J* = 9.8 Hz, 1H), 2.67 (dq, *J* = 9.8, 6.8 Hz, 1H), 1.36 (d, *J* = 6.8 Hz, 3H), 1.29 – 1.24 (m, 21H), -0.10 (s, 9H).

^{13}**C NMR (100 MHz, CDCl$_3$)** δ= 145.3, 128.3, 127.6, 126.1, 83.0, 56.2, 48.9, 40.4, 29.8, 25.5, 24.8, 23.3, 23.2, 0.2.

^{11}**B NMR (129 MHz, CDCl$_3$)** δ= 31.4.

HRMS-(ESI+) for $C_{23}H_{41}BNO_3SSi$ [M+H]$^+$: calculated 450.2669; found: 450.2660.

(2*R*,3*S*)-1-((*S*)-tert-butylsulfinyl)-3-((*R*)-1-phenylpropyl)-2-(4,4,5,5-tetramethyl-1,3,2-dioxaborolan-2-yl)-2-(trimethylsilyl)aziridine ((*S*$_S$*,2*R,3*S**,*R*)-42)**

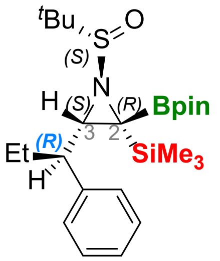

(*S*$_S$*,2*R,3*S**,*R*)-42**

The product was synthesized following the general procedure for the aziridination of *N-tert*-butanesulfinyl aldimines and purified by flash chromatography using as eluent a mixture of petroleum ether/ethyl acetate (100:3). The product **(*S*$_S$*,2*R**,3*S**,*R*)-42** was isolated as a white solid (32 mg, 0.07 mmol, 35%). *The products* ***(S$_S$*,2R*,3S*,R)-42/(R$_S$*,2S*,3R*,R)-42/*** *were isolated as a white solid (30 mg, 0.06 mmol, 32%).* M.p = 95.6 - 97.8 ºC.

$[\alpha]_D^{20}$: +61.8 (c = 1.0, CH_2Cl_2).

The enantiopurity was determined by HPLC analysis using a Daicel Chiralpak IF column, *n*-hexane/*i*-PrOH (90:10), flow rate 1.0 mL/min, wavelength = 210 nm, t_R = 4.34.

^{1}**H NMR (400 MHz, CDCl$_3$)** δ= 7.64 – 7.59 (m, 2H), 7.57 – 7.52 (m, 2H), 7.49 – 7.42 (m, 1H), 3.15 (d, *J* = 9.8 Hz, 1H), 2.79 – 2.70 (m, 1H), 2.17 – 2.09 (m, 1H), 1.96 – 1.91 (m, 1H), 1.55 (s, 9H), 1.53 (s, 6H), 1.51 (s, 6H), 1.19 (t, *J* = 7.5 Hz, 3H), 0.14 (s, 9H).

^{13}**C NMR (100 MHz, CDCl$_3$)** δ= 143.8, 129.2, 128.5, 128.1, 127.4, 126.1, 82.9, 56.1, 47.8, 47.3, 31.2, 25.5, 24.8, 23.2, 11.8, 0.2.

^{11}**B NMR (129 MHz, CDCl$_3$)** δ= 32.1.

HRMS-(ESI+) for $C_{24}H_{43}BNO_3SSi$ [M+H]$^+$: calculated 464.2826; found: 464.2815.

Spectral data for diversified aziridines

(2*R*,3*S*)-1-(tert-butylsulfinyl)-3-isobutyl-2-(trimethylsilyl)-2-vinylaziridine (43)

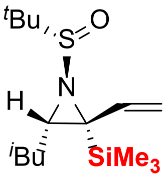

43

The product was synthesized following the desbribed method and purified by flash chromatography using as eluent a mixture of petroleum ether/ethyl acetate (100:3). The product **43** was isolated as a colourless oil (14 mg, 0.05 mmol, 23%).

^{1}**H NMR (400 MHz, CDCl$_3$)** δ= 6.43 (dd, *J* = 17.1, 10.4 Hz, 1H), 5.29 (dd, *J* = 10.4, 1.5 Hz, 1H), 5.21 (dd, *J* = 17.2, 1.6 Hz, 1H), 2.67 (dd, *J* = 8.9, 4.6 Hz, 1H), 1.73

(dddd, J = 13.3, 12.2, 8.4, 6.6 Hz, 1H), 1.54 – 1.47 (m, 1H), 1.41 – 1.30 (m, 1H), 1.24 (s, 9H), 1.06 (d, J = 8.9 Hz, 3H), 0.94 (d, J = 8.9, 3H), 0.12 (s, 9H).

^{13}C NMR (100 MHz, CDCl$_3$) δ= 137.5, 119.0, 56.1, 48.9, 48.4, 39.0, 27.6, 23.3, 22.5, 22.5, -1.9.

1-(tert-butylsulfonyl)-3-isobutyl-2-(4,4,5,5-tetramethyl-1,3,2-dioxaborolan-2-yl)-2-(trimethylsilyl)aziridine (44)

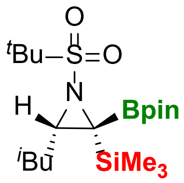

44

The product was synthesized following the desbribed method and purified by flash chromatography using as eluent a mixture of CH_2Cl_2/Et_2O (200:1). The product **44** was isolated as a colourless oil (58 mg, 0.14 mmol, 70%).

^{1}H NMR (400 MHz, CDCl$_3$) δ= 3.15 (t, J = 6.6 Hz, 1H), 1.76 (dt, J = 13.4, 6.7 Hz, 1H), 1.49 – 1.41 (m, 2H), 1.45 (s, 9H), 1.25 (s, 12H), 1.01 (d, J = 8.9 Hz, 3H), 0.94 (d, J = 8.9, 3H), 0.15 (s, 9H).

^{13}C NMR (100 MHz, CDCl$_3$) δ= 83.9, 59.2, 46.4, 39.2, 27.7, 25.1, 25.0, 24.6, 22.8, -0.2.

^{11}B NMR (129 MHz, CDCl$_3$) δ= 30.6.

HRMS-(ESI+) for $C_{19}H_{41}BNO_4SSi$ [M+H]$^+$: calculated 418.2619; found: 418.2609.

2-methyl-*N*-((3*R*,4*R*)-6-methyl-3-(4,4,5,5-tetramethyl-1,3,2-dioxaborolan-2-yl)-3-(trimethylsilyl)hept-1-en-4-yl)propane-2-sulfonamide (45)

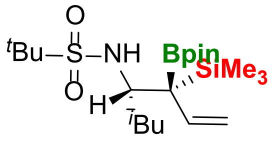

45

The product was synthesized following the described method and purified by flash chromatography using as eluent a mixture of petroleum ether/ethyl acetate (100:3). The product **45** was isolated as a yellowish solid (18 mg, 0.04 mmol, 40%).

^{1}H NMR (400 MHz, CDCl$_3$) δ= 5.80 (dd, J = 17.9, 10.9 Hz, 1H), 5.05 (dd, J = 17.8, 1.3 Hz, 1H), 4.98 (dd, J = 10.9, 1.3 Hz, 1H), 4.43 (d, J = 9.2 Hz, 1H), 3.90 (ddd, J = 11.5, 9.2, 2.2 Hz, 1H), 2.21 – 2.08 (m, 1H), 1.74 (ddd, J = 14.2, 11.6, 2.6 Hz, 1H), 1.38 (s, 9H), 1.34 – 1.28 (m, 1H), 1.28 – 1.21 (m, 12H), 1.00 (d, J = 6.5 Hz, 3H), 0.93 (d, J = 6.6 Hz, 3H), 0.09 (s, 9H).

^{13}C NMR (100 MHz, CDCl$_3$) δ= 141.1, 113.7, 83.5, 60.5, 57.1, 46.5, 25.3, 25.1, 25.0, 24.7, 24.6, 21.2, -0.6.

^{11}B NMR (129 MHz, CDCl$_3$) δ= 33.2.

HRMS-(ESI+) for $C_{21}H_{45}BNO_4SSi$ [M+H]$^+$: calculated 446.2932; found: 446.2923

2-methyl-N-(5-methyl-2-(trimethylsilyl)hex-1-en-3-yl)propane-2-sulfonamide (46)

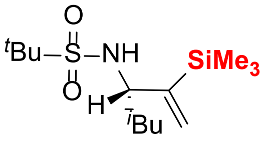

46

The product was synthesized following the described method and purified by flash chromatography using as eluent a mixture of petroleum ether/ethyl acetate (100:3). The product **46** was isolated as a colourless oil (5 mg, 27%).

^{1}H NMR (400 MHz, $CDCl_3$) δ= 5.76 (dd, *J* = 1.8, 1.1 Hz, 1H), 5.49 (dd, *J* = 1.8, 0.5 Hz, 1H), 4.15 (t, *J* = 9.3, 4.4 Hz, 1H), 3.82 (d, *J* = 9.4 Hz, 1H), 1.82 (dt, *J* = 9.0, 6.7, 4.6 Hz, 1H), 1.44 – 1.37 (m, 1H), 1.35 (s, 9H), 1.27 – 1.24 (m, 1H), 0.98 (d, *J* = 6.5 Hz, 3H), 0.92 (d, *J* = 6.7 Hz, 3H), 0.15 (s, 9H).

^{13}C NMR (100 MHz, $CDCl_3$) δ= 153.9, 124.7, 59.8, 57.80, 48.2, 24.9, 24.4, 23.4, 21.9, -0.4.

HRMS-(ESI+) for $C_{14}H_{32}NO_2SSi$ [M+H]$^+$: calculated 306.1923; found: 306.1918

1-(tert-butylsulfonyl)-2-isobutyl-3-(4,4,5,5-tetramethyl-1,3,2-dioxaborolan-2-yl) aziridine (47)

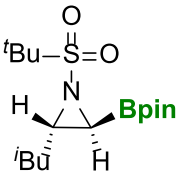

47

The product was synthesized following the described method and purified by flash chromatography using as eluent a mixture of CH_2Cl_2/Et_2O (200:1). The product **47** was isolated as a colourless oil (3 mg, 10%).

^{1}H NMR (400 MHz, $CDCl_3$) δ= 2.70 (ddd, *J* = 8.1, 7.1, 6.1 Hz, 1H), 1.99 (d, *J* = 8.1 Hz, 1H), 1.67 – 1.52 (m, 1H), 1.44 – 1.40 (m, 1H), 1.31 (s, 9H), 1.25 – 1.20 (m, 1H), 1.10 (s, 12H), 0.81 (t, *J* = 6.7 Hz, 6H).

^{13}C NMR (100 MHz, $CDCl_3$) δ= 84.5, 59.4, 39.2, 38.9, 27.1, 24.9, 24.8, 24.4, 22.7, 22.6.

^{11}B NMR (129 MHz, $CDCl_3$) δ= 30.5.

HRMS-(ESI+) for $C_{16}H_{33}BNO_4S$ [M+H]$^+$: calculated 346.2223; found: 346.2215.

(2*R*,3*S*)-1-((*S*)-tert-butylsulfinyl)-2-isobutyl-3-(trimethylsilyl)aziridine ((*S*$_S$,2R**,*3S**)-48)**

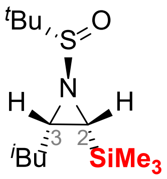

(*S*$_S$*,2*R,3*S**)-48**

The product was synthesized following the general procedure for protodeborylation, heating up to 90 ºC, and it was purified by flash chromatography using as eluent a mixture of petroleum ether/ethyl acetate (100:4). The product **(*S*$_S$*,2*R**,**3S***)-48** was isolated as yellowish oil (22 mg, 0.08 mmol, 40%). *The products **(S$_S$*,2R*,3S*)-48/(R$_S$*,2R*,3S*)-48** were isolated as a yellowish oil (12 mg, 0.04 mmol, 22%).*

$[\alpha]_D^{20}$: 98.7 (c = 1.0, CH_2Cl_2).

The e.r. was determined by HPLC analysis using a Daicel Chiralpak IF column, *n*-hexane/*i*-PrOH (90:10), flow rate 1.0 mL/min, wavelength = 210 nm, t_R = 4.75.

^{1}H NMR (400 MHz, CDCl$_3$) δ= 2.11 (ddd, *J* = 9.3, 8.0, 3.2 Hz, 1H), 2.02 (d, *J* = 8.0 Hz, 1H), 1.86 – 1.71 (m, 1H), 1.63 – 1.53 (m, 1H), 1.20 (s, 9H), 1.14 – 1.02 (m, 1H), 0.96 (dd, *J* = 6.7, 5.9 Hz, 6H), 0.13 (s, 9H).

^{13}C NMR (100 MHz, CDCl$_3$) δ= 56.1, 40.0, 36.5, 29.8, 27.7, 24.5, 23.0, 22.9, -0.6.

HRMS-(ESI+) for $C_{13}H_{30}NOSSi$ [M+H]$^+$: calculated 276.1817; found: 276.1819.

(2*S*,3*S*)-1-((*S*)-tert-butylsulfinyl)-2-phenyl-3-(trimethylsilyl)aziridine ((*S*$_S$*,2*S,3S*)-49)**

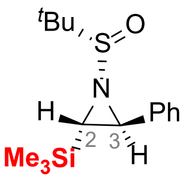

(*S*$_S$*,2*S**,3*S**)-49

The product was synthesized following the general procedure for protodeborylation, heating up to 90 °C, and it was purified by flash chromatography using as eluent a mixture of petroleum ether/ethyl acetate (100:5). The product **(*S*$_S$*,2*S**,3S*)-49** was isolated as a white solid (35 mg, 0.1 mmol, 51%). *The products **(S$_S$*,2S*,3S*)-49/(R$_S$*,2S*,3S*)-49** were isolated as a white solid (28 mg, 0.09 mmol, 44%)*. M.p = 72.5 – 73.0 °C.

$[\alpha]_D^{20}$: -18.9 (c = 1.0, CH_2Cl_2).

The enantiopurity was determined by HPLC analysis using a Daicel Chiralpak IF column, *n*-hexane/*i*-PrOH (98:02), flow rate 1.0 mL/min, wavelength = 210 nm, t_R = 5.31.

^{1}H NMR (400 MHz, CDCl$_3$) δ= 7.37 – 7.26 (m, 5H), 3.57 (d, *J* = 5.6 Hz, 1H), 1.69 (d, *J* = 5.6 Hz, 1H), 1.15 (s, 9H), 0.25 (s, 9H).

^{13}C NMR (100 MHz, CDCl$_3$) δ= 139.3, 128.7, 127.6, 126.6, 57.1, 39.8, 35.4, 23.1, -0.6.

HRMS-(ESI+) for $C_{15}H_{26}NOSSi$ [M+H]$^+$: calculated 296.1504; found: 296.1500.

(2*R*,3*S*)-1-((*S*)-tert-butylsulfinyl)-2-((*R*)-1-phenylethyl)-3-(trimethylsilyl)aziridine ((*S*$_S$*,2*R,3S*,*R*)-50)**

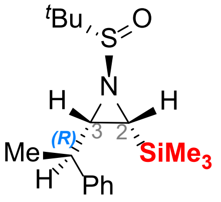

(*S*$_S$*,2*R**,3*S**,*R*)-50

The product was synthesized following the general procedure for protodeborylation, heating up to 65 °C, and it was purified by flash chromatography using as eluent a mixture of petroleum ether/ethyl acetate (100:4). The product **(*S*$_S$*,2*R**,3S*,*R*)-50)** was isolated as a yellowish oil (14 mg, 0.04 mmol, 21%). *The products **(S$_S$*,2R*,3S*,R)-50/(R$_S$*,2R*,3S*,R)-50** were isolated as a yellowish oil (10 mg, 0.03 mmol, 16%).*

$[\alpha]_D^{20}$: +74.9 (c = 1.0, CH_2Cl_2).

The enantiopurity was determined by HPLC analysis using a Daicel Chiralpak IC column, *n*-hexane/*i*-PrOH (90:10), flow rate 1.0 mL/min, wavelength = 210 nm, t_R = 5.76.

^{1}H NMR (400 MHz, $CDCl_3$) δ= 7.36 – 7.16 (m, 5H), 2.53 – 2.38 (m, 2H), 2.17 (d, *J* = 7.3 Hz, 1H), 1.37 (d, *J* = 6.4 Hz, 3H), 1.26 (s, 9H), -0.05 (s, 9H).

^{13}C NMR (100 MHz, $CDCl_3$) δ= 144.7, 128.7, 127.3, 126.6, 56.1, 43.0, 41.3, 29.9, 27.3, 23.0, -0.7.

HRMS-(ESI+) for $C_{17}H_{29}NOSSi$ [M+H]$^+$: calculated 323.1739; found: 324.1819.

(*R*)-1-((*S*)-tert-butylsulfinyl)-2-(trimethylsilyl)-5-oxa-1-azaspiro[2.3]hexane ((*S*$_S$*,*R)-51)**

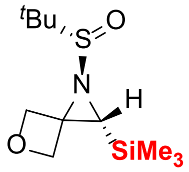

(*S*$_S$*,*R)-51**

The product was synthesized following the general procedure for protodeborylation, heating up to 65 °C, and it was purified by flash chromatography using as eluent a mixture of petroleum ether/ethyl acetate (100:5). The product **(*S*$_S$*,*R**)-51** was isolated as a white solid (32 mg, 0.12 mmol, 60%). *The products **(S$_S$*,R*)-51/(R$_S$*,R*)-51** were isolated as a white solid (30 mg, 0.12 mmol, 57%).* M.p = 67.3 – 64.1 °C.

$[\alpha]_D^{20}$: +201.9 (c = 1.0, CH_2Cl_2).

The enantiopurity was determined by HPLC analysis using a Daicel Chiralpak IF column, *n*-hexane/*i*-PrOH (90:10), flow rate 1.0 mL/min, wavelength = 210 nm, t_R = 7.49.

^{1}H NMR (400 MHz, $CDCl_3$) δ= 5.07 (dt, *J* = 7.9, 0.9 Hz, 1H), 4.87 (dd, *J* = 8.0, 1.0 Hz, 1H), 4.83 – 4.76 (m, 2H), 2.10 (s, 1H), 1.22 (s, 9H), 0.10 (s, 9H).

^{13}C NMR (100 MHz, $CDCl_3$) δ= 78.5, 56.1, 44.7, 25.5, 22.8, -1.9.

HRMS-(ESI+) for $C_{11}H_{24}NO_2SSi$ [M+H]$^+$: calculated 262.1297; found: 262.1297.

(*R*)-1-((*S*)-tert-butylsulfinyl)-2-(trimethylsilyl)-5-oxa-1-azaspiro[2.3]hexane-2-d ((*S*$_S$*,*R)-52)**

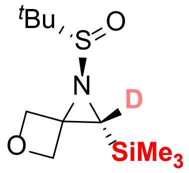

(*S*$_S$*,*R)-52**

The product was synthesized following the general procedure for deborylation reaction followed by deuteration and it was purified by flash chromatography using as eluent a mixture of petroleum ether/ethyl acetate (100:5). The product **(*S*$_S$*,*R**)-52** was isolated as a yellowish oil (28 mg, 0.11 mmol, 52%). *The product **(S$_S$*,R*)-52/ (R$_S$*,R*)-52** was isolated as yellowish oil (34 mg, 0.13 mmol, 55%).*

$[\alpha]_D^{20}$: +104.6 (c = 1.0, CH_2Cl_2).

The enantiopurity was determined by HPLC analysis using a Daicel Chiralpak IC column, *n*-hexane/*i*-PrOH (99:01), flow rate 0.8 mL/min, wavelength = 210 nm, t_R = 7.49.

^{1}H NMR (400 MHz, $CDCl_3$) δ= 5.07 (d, *J* = 7.6 Hz, 1H), 4.88 (d, *J* = 7.9 Hz, 1H), 4.84 – 4.77 (m, 2H), 1.22 (s, 9H), 0.09 (s, 9H).

^{13}C NMR (100 MHz, $CDCl_3$) δ= 78.5, 56.1, 44.6, 22.8, -1.9.

^{2}H NMR (61.285 MHz, $CDCl_3$) δ= 2.06.

HRMS-(ESI+) for $C_{11}H_{23}DNO_2SSi$ $[M+H]^+$: calculated 262.1297; found: 263.1353

^{1}H, ^{13}C, ^{11}B, NMR spectra for diborylsilymethane, α–monohalo diborylsilylmethanes and α–monohalo borylsilylmethane reagents

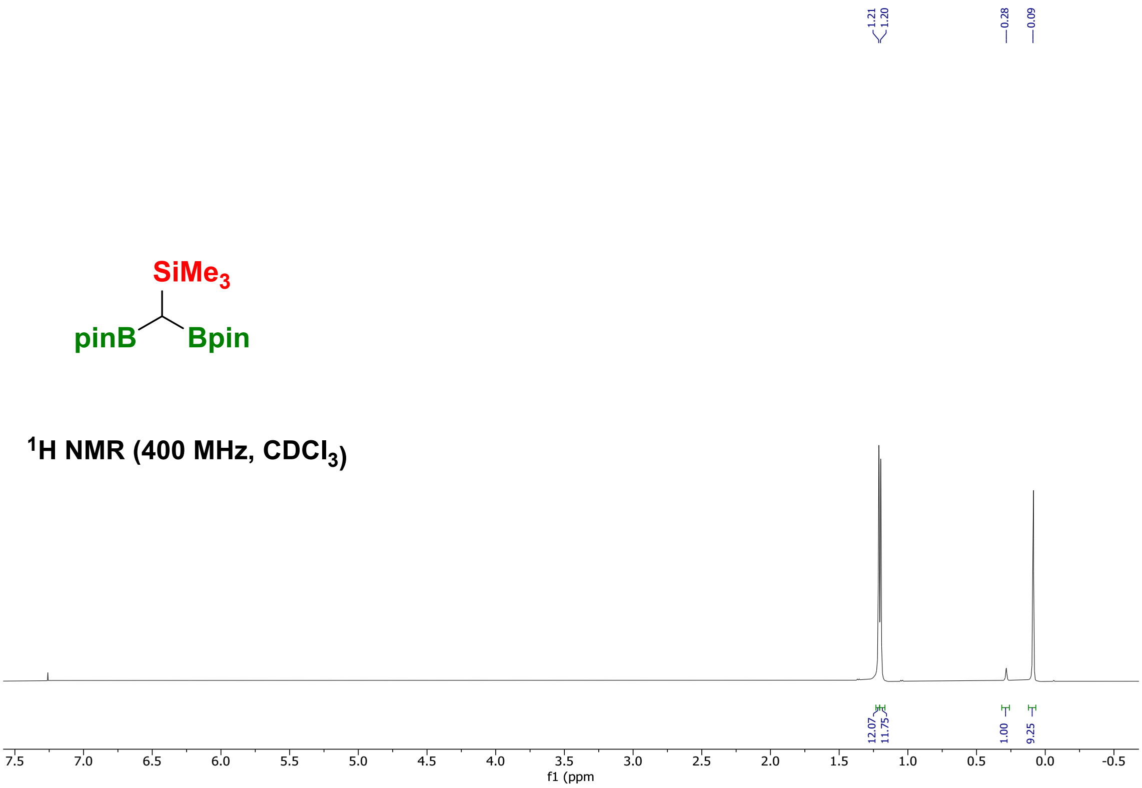

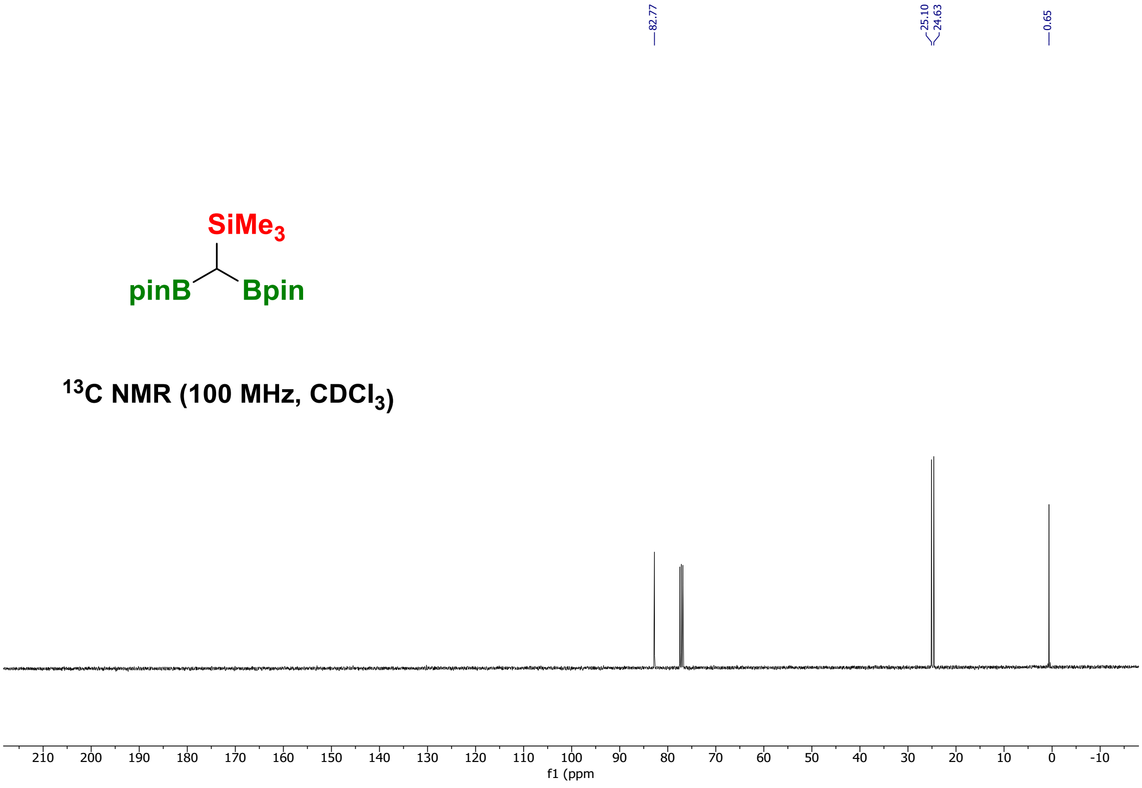

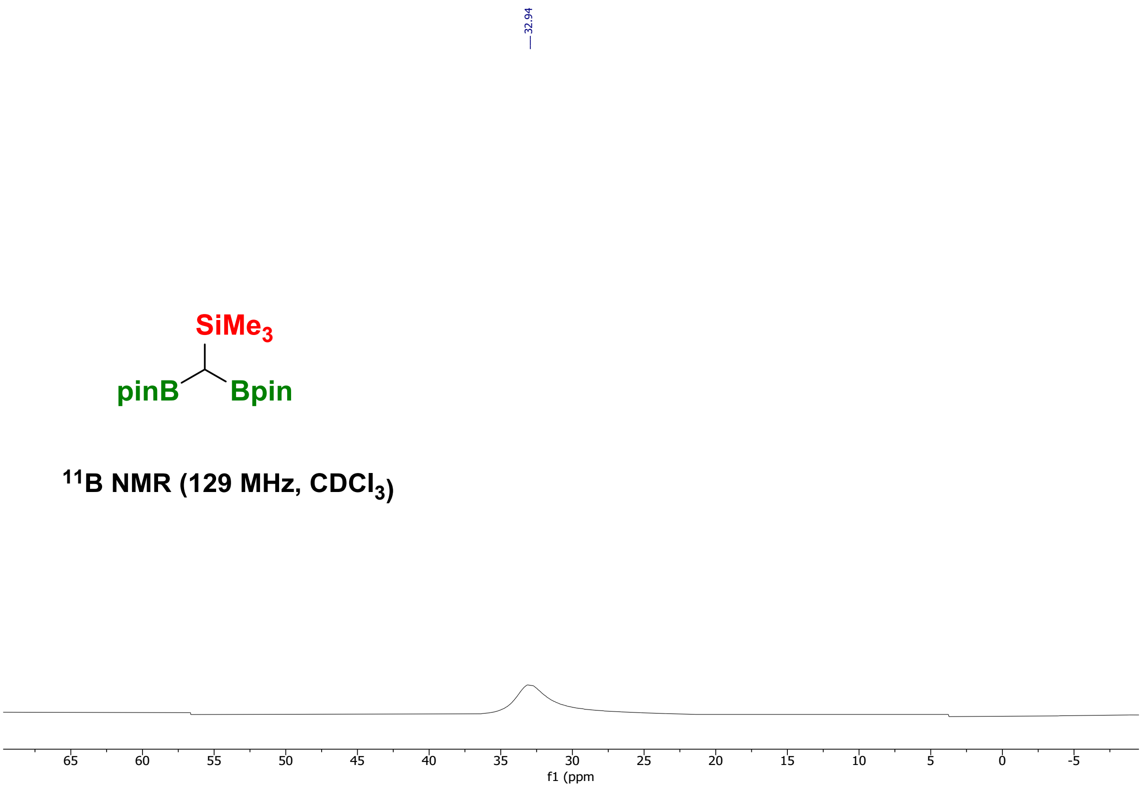
32.94
SiMe3
pinB
Bpin
11B NMR (129 MHz, CDCl3)
65 60 55 50 45 40 35 30 25 20 15 10 5 0 -5
f1 (ppm

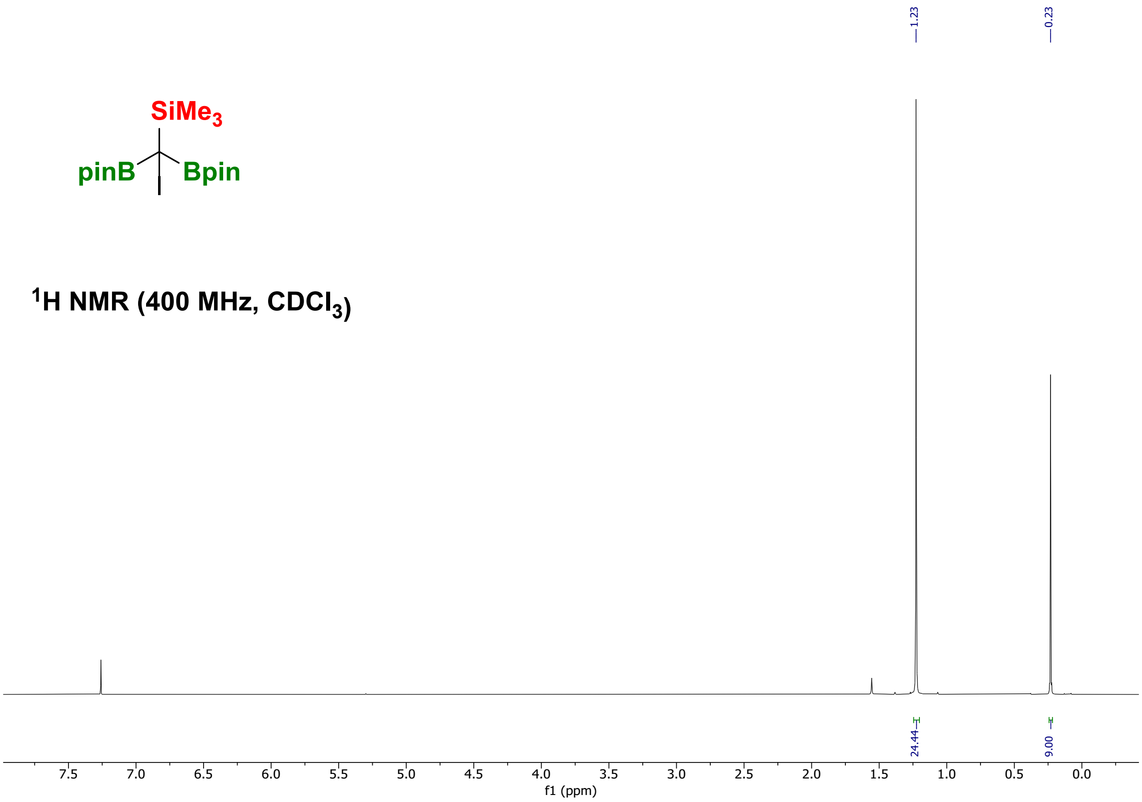
1.23
0.23
SiMe3
pinB
Bpin
1H NMR (400 MHz, CDCl3)
24.44
9.00
7.5 7.0 6.5 6.0 5.5 5.0 4.5 4.0 3.5 3.0 2.5 2.0 1.5 1.0 0.5 0.0
f1 (ppm)

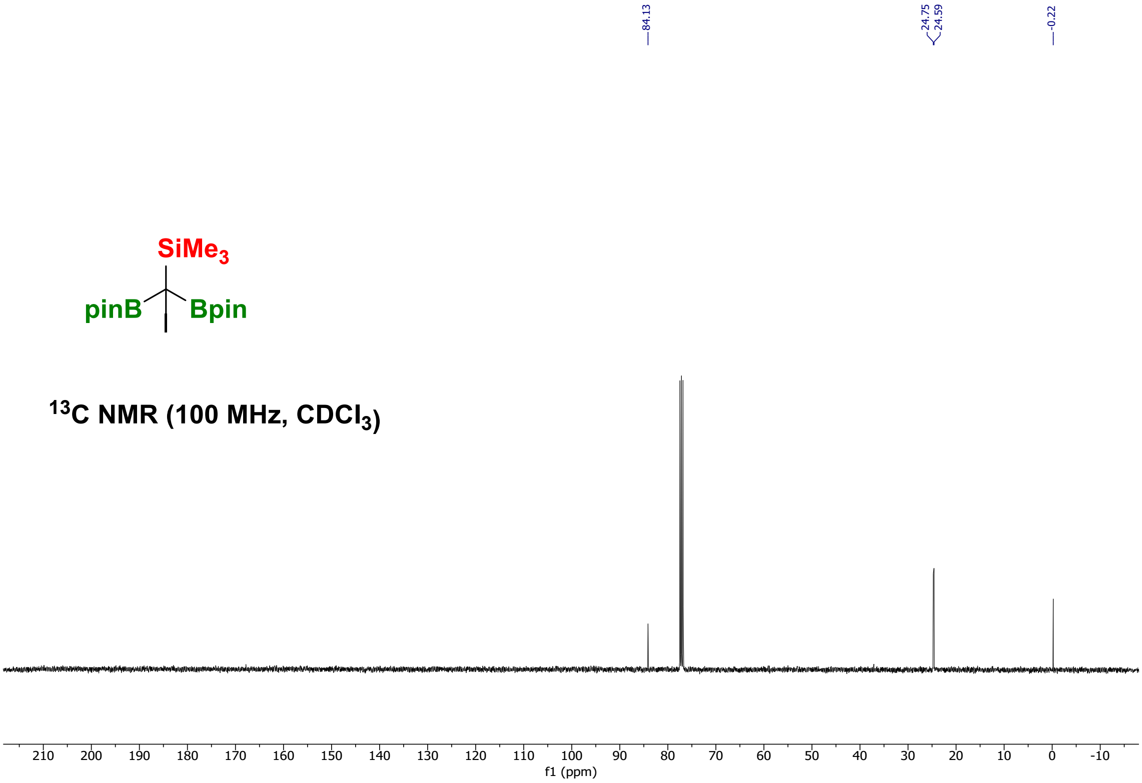

^{13}C NMR (100 MHz, $CDCl_3$)

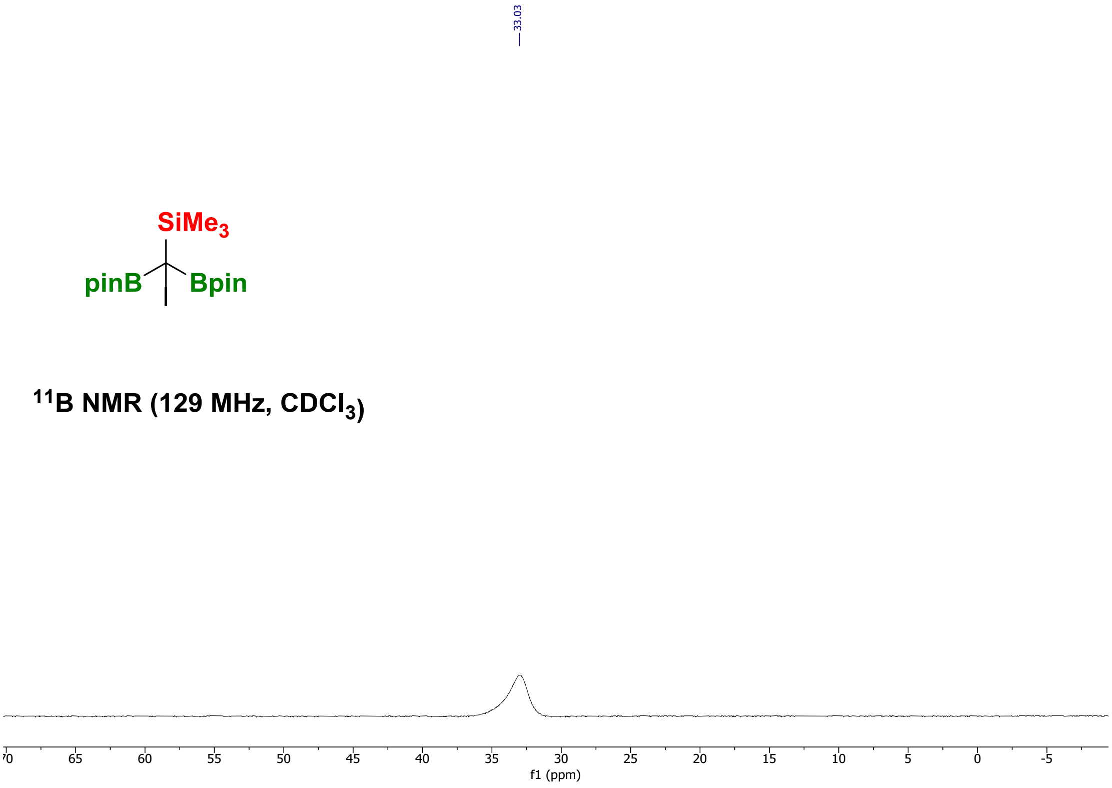

^{11}B NMR (129 MHz, $CDCl_3$)

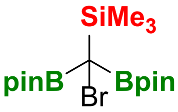

^{1}H NMR (400 MHz, $CDCl_3$)

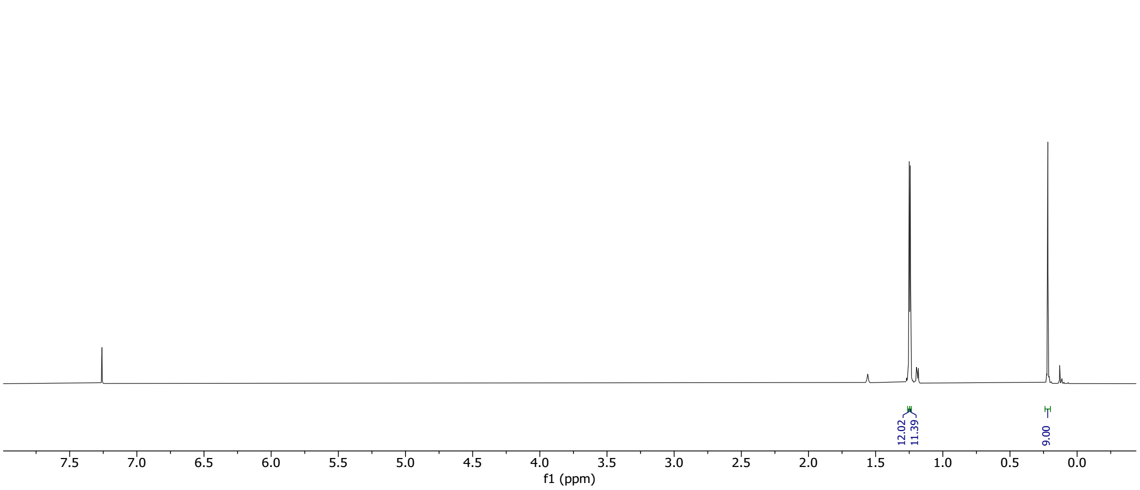

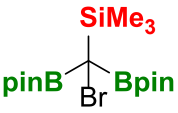

^{13}C NMR (100 MHz, $CDCl_3$)

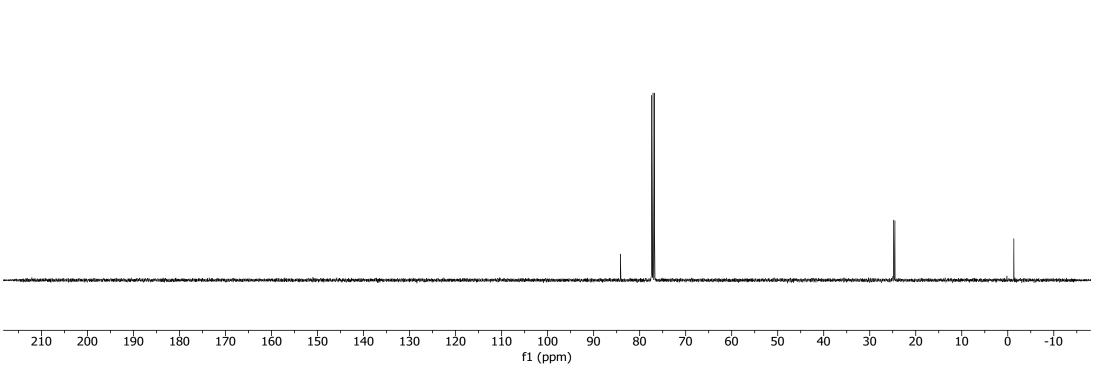

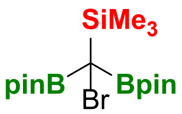

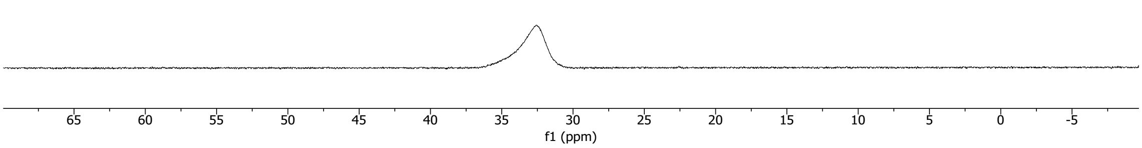

^{11}B NMR (129 MHz, $CDCl_3$)

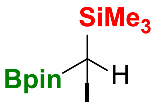

III-I

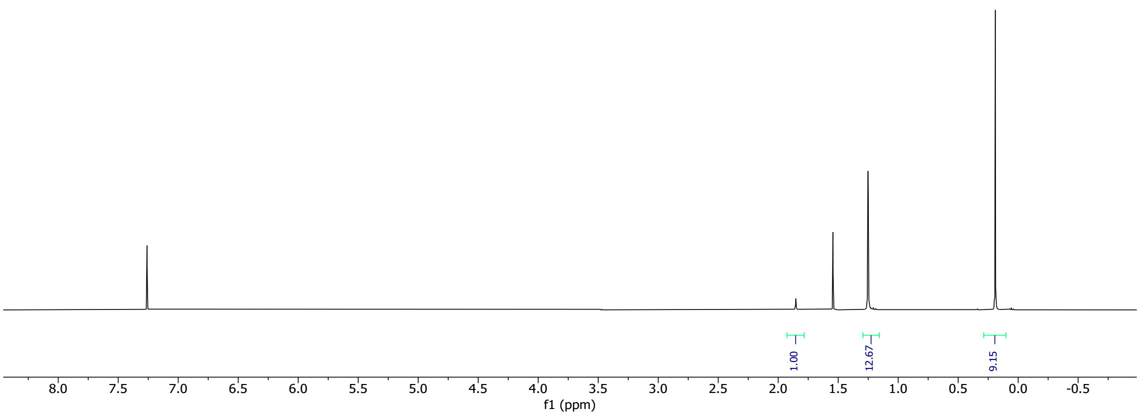

^{1}H NMR (400 MHz, $CDCl_3$)

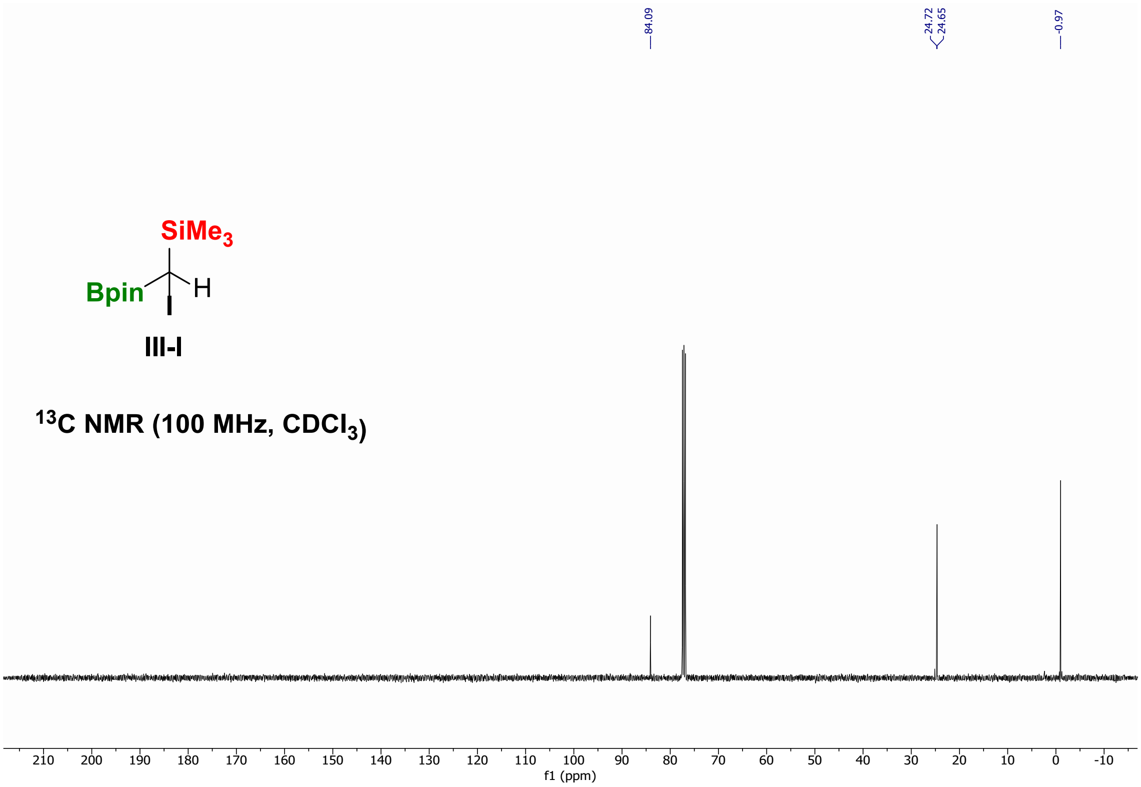

84.09
24.72
24.65
-0.97
SiMe3
Bpin
H
I
III-I
13C NMR (100 MHz, CDCl3)
210 200 190 180 170 160 150 140 130 120 110 100 90 80 70 60 50 40 30 20 10 0 -10
f1 (ppm)

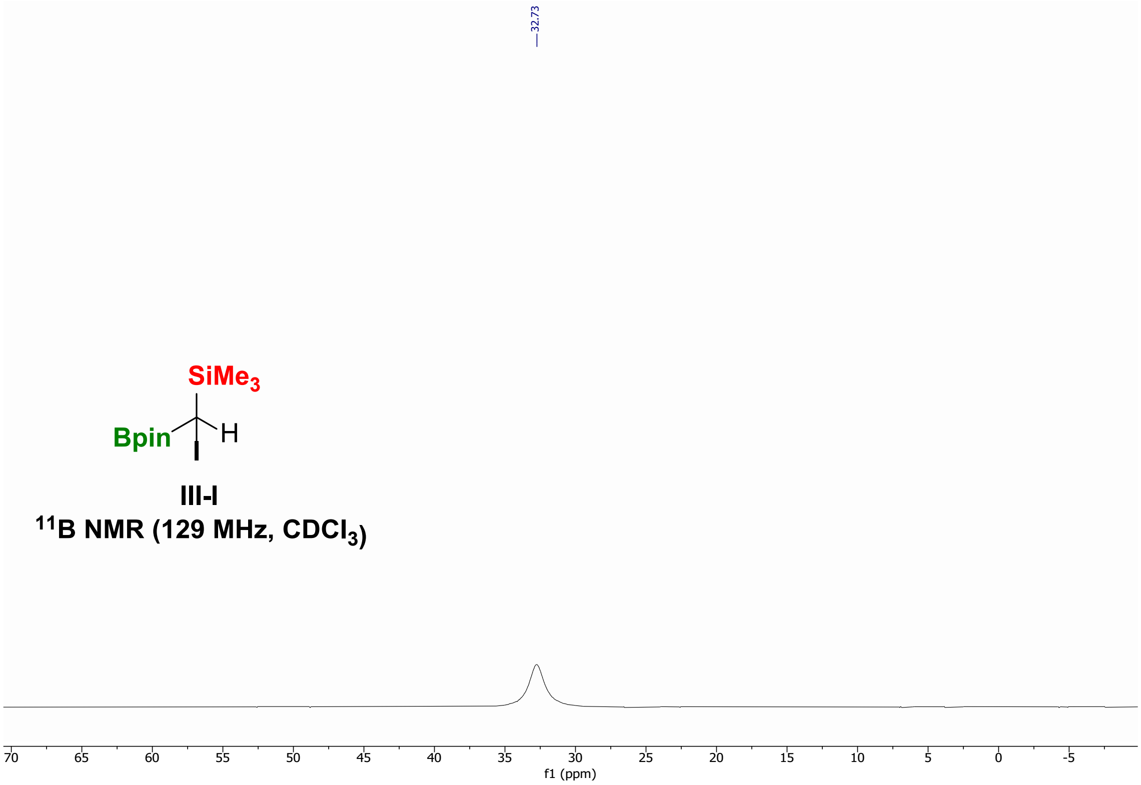

32.73
SiMe3
Bpin
H
I
III-I
11B NMR (129 MHz, CDCl3)
70 65 60 55 50 45 40 35 30 25 20 15 10 5 0 -5
f1 (ppm)

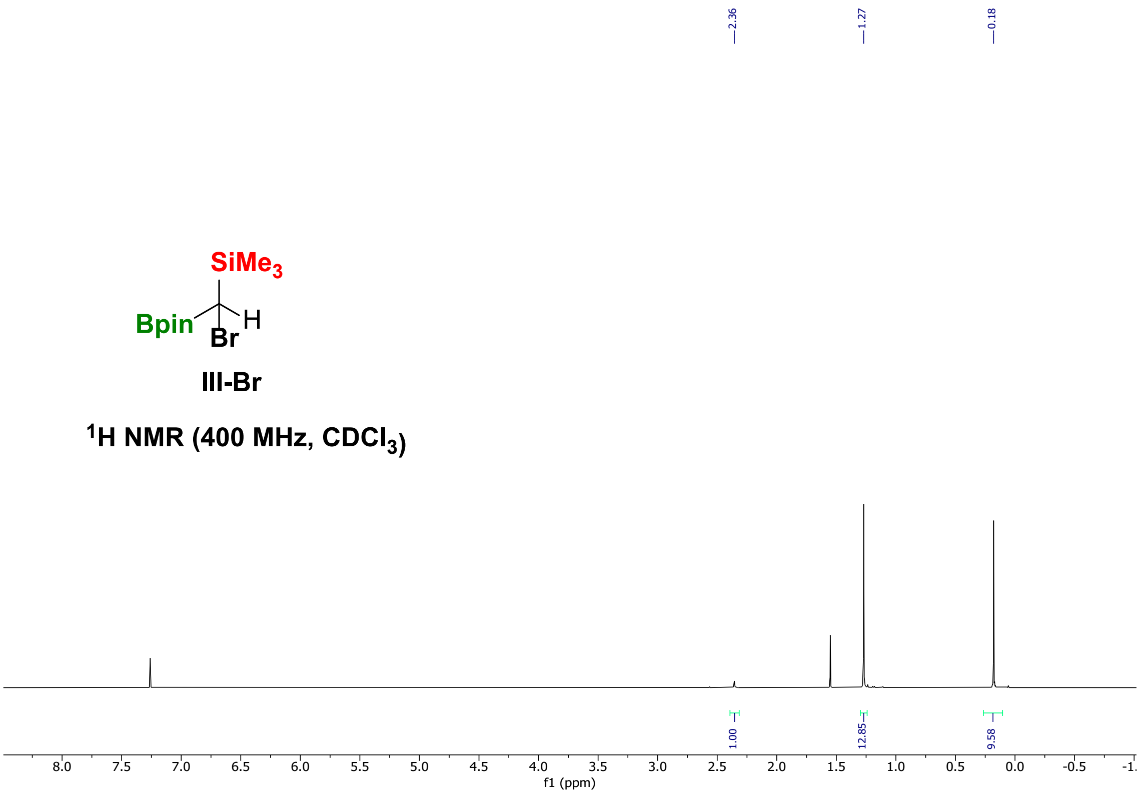
2.36
1.27
0.18
SiMe3
Bpin
H
Br
III-Br
1H NMR (400 MHz, CDCl3)
1.00
12.85
9.58
8.0
7.5
7.0
6.5
6.0
5.5
5.0
4.5
4.0
3.5
3.0
2.5
2.0
1.5
1.0
0.5
0.0
-0.5
f1 (ppm)

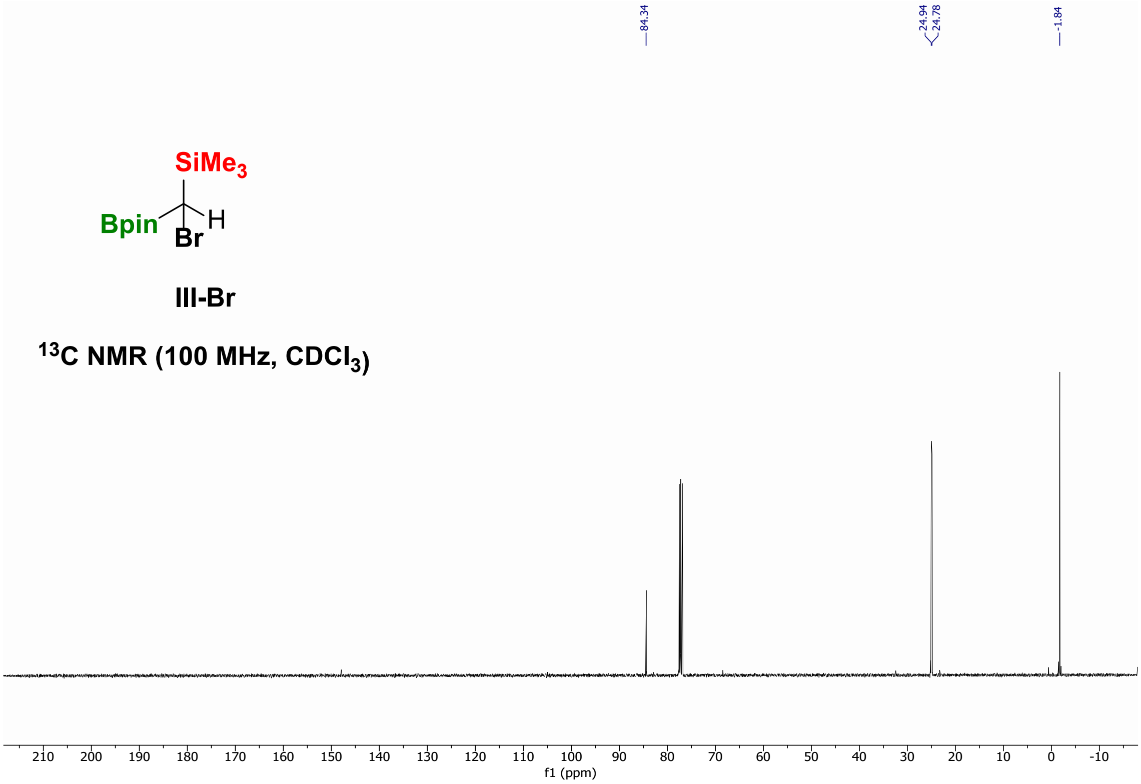
84.34
24.94
24.78
-1.84
SiMe3
Bpin
H
Br
III-Br
13C NMR (100 MHz, CDCl3)
210
200
190
180
170
160
150
140
130
120
110
100
90
80
70
60
50
40
30
20
10
0
-10
f1 (ppm)

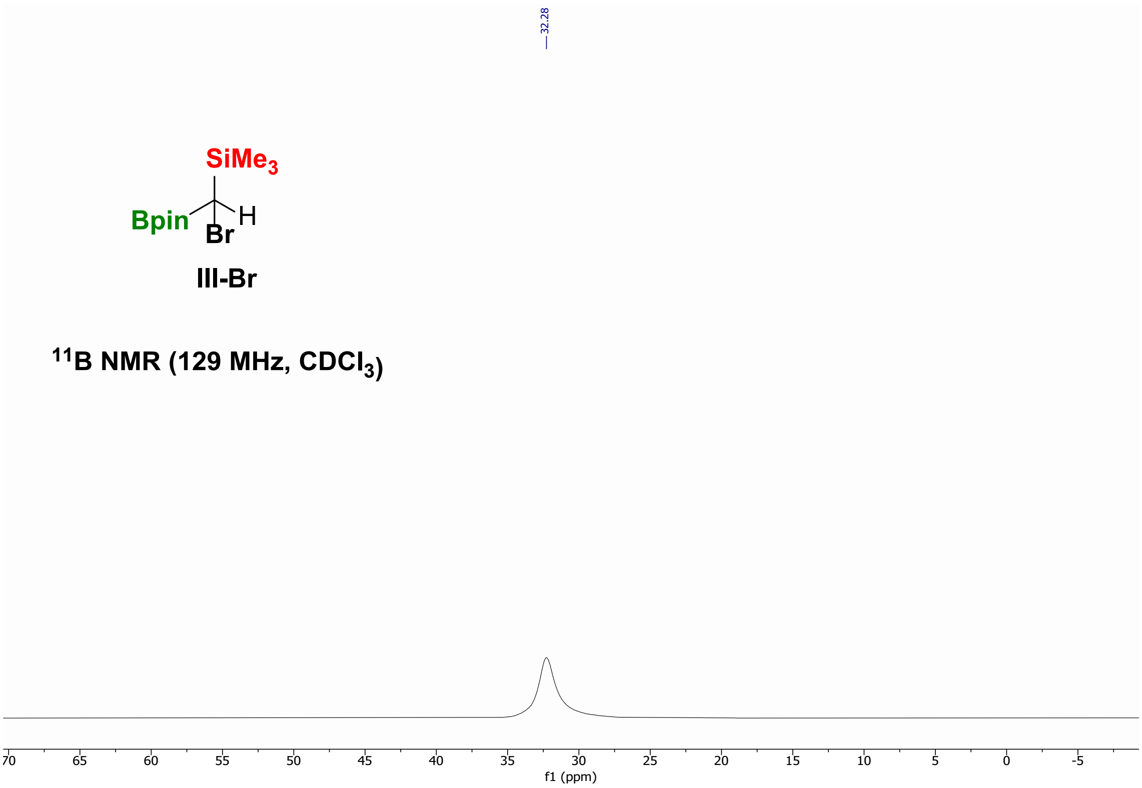

32.28
SiMe3
Bpin
H
Br
III-Br
11B NMR (129 MHz, CDCl3)
70
65
60
55
50
45
40
35
30
25
20
15
10
5
0
-5
f1 (ppm)

^{1}H, ^{13}C, ^{19}F NMR spectra for *N-tert*-butanesulfinyl ketimines and aldimines

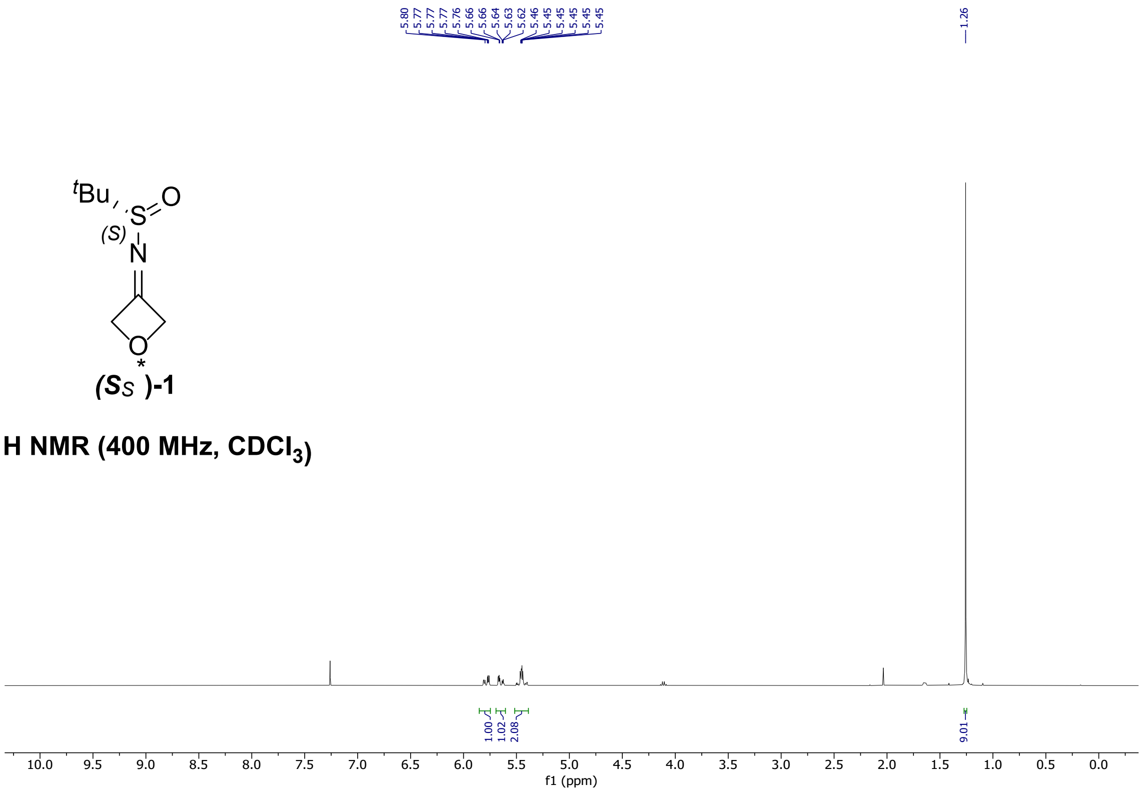

(S_S*)-1

^{1}H NMR (400 MHz, $CDCl_3$)

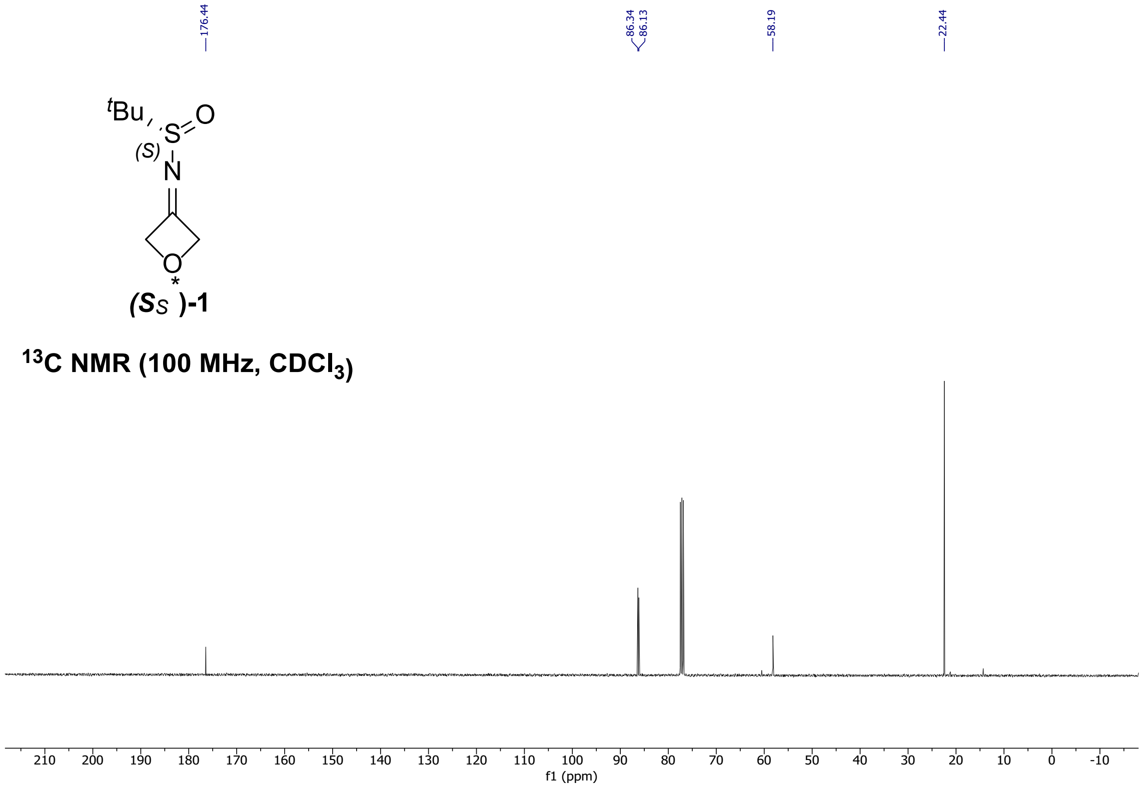

(S_S*)-1

^{13}C NMR (100 MHz, $CDCl_3$)

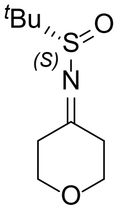

(S_S*)-3

^{1}H NMR (400 MHz, $CDCl_3$)

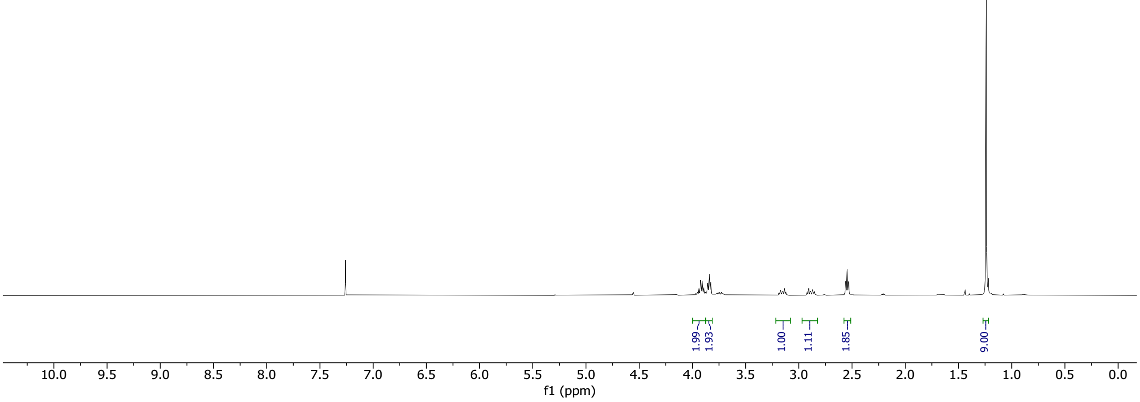

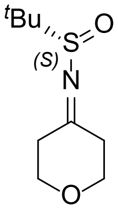

(S_S*)-3

^{13}C NMR (100 MHz, $CDCl_3$)

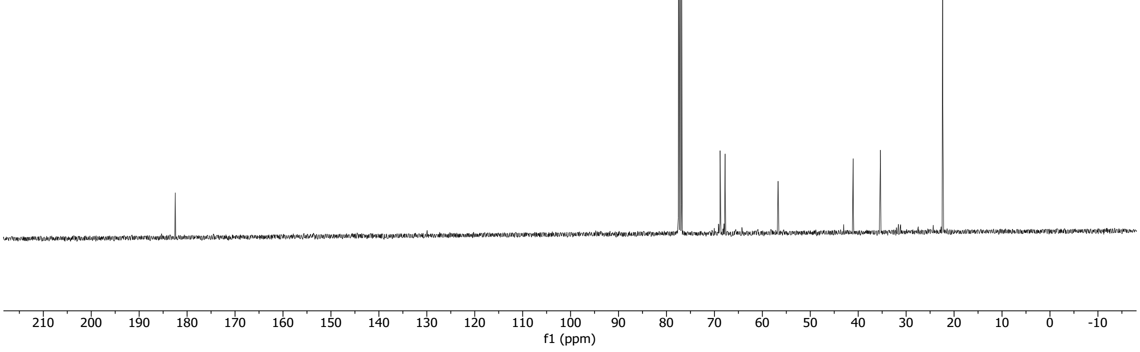

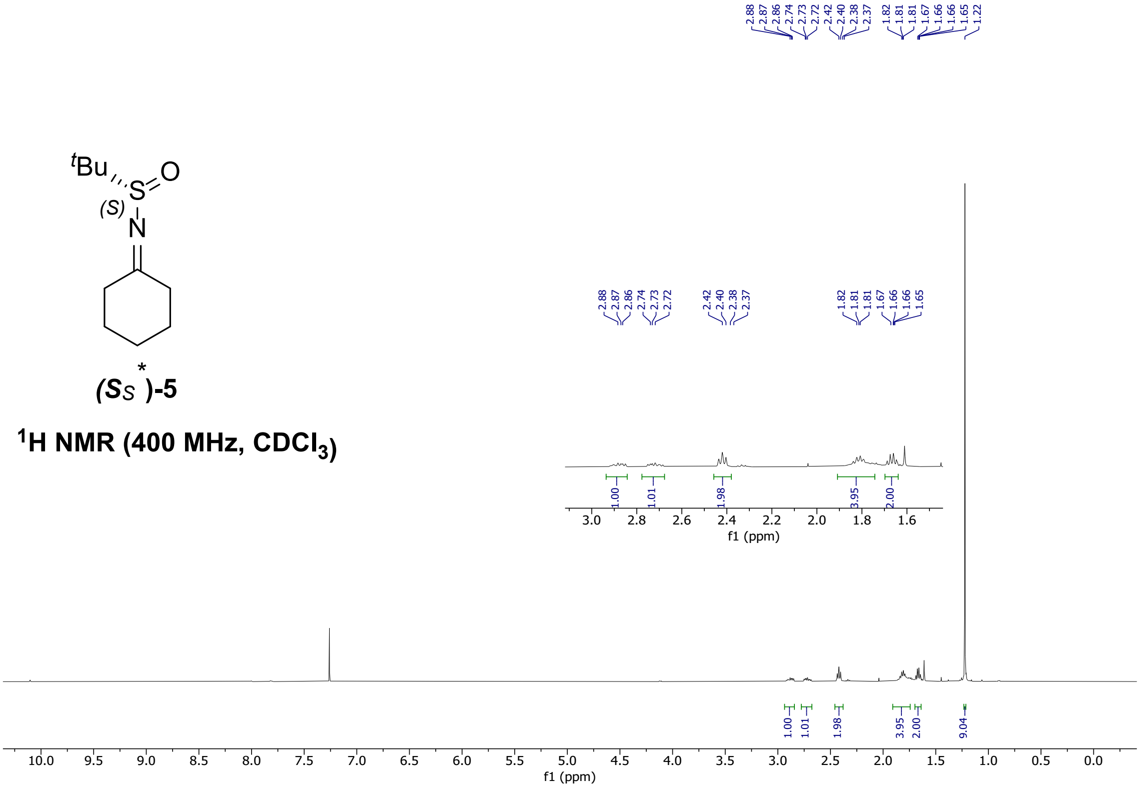

tBu
S
O
(S)
N
(SS*)-5
1H NMR (400 MHz, CDCl3)
2.88 2.87 2.86 2.74 2.73 2.72 2.42 2.40 2.38 2.37 1.82 1.81 1.81 1.67 1.66 1.66 1.65 1.22
1.00 1.01 1.98 3.95 2.00 9.04
f1 (ppm)

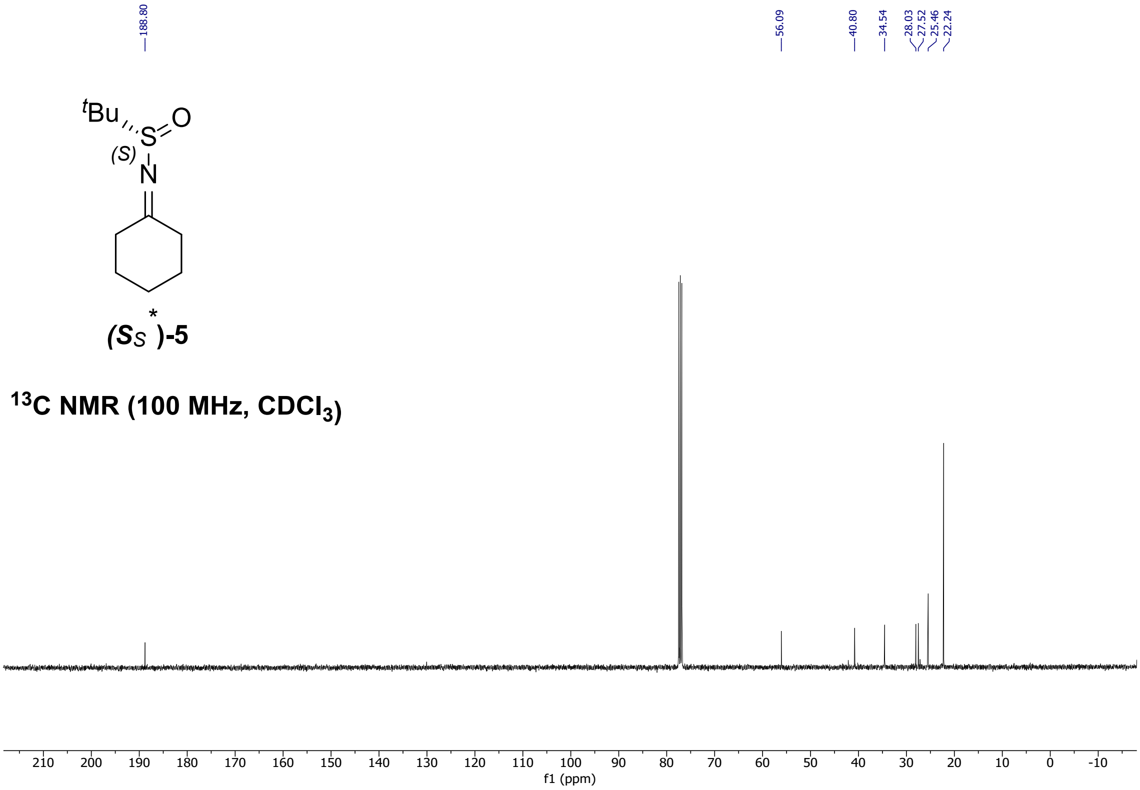

188.80
56.09
40.80
34.54
28.03 27.52 25.46 22.24
tBu
S
O
(S)
N
(SS*)-5
13C NMR (100 MHz, CDCl3)
f1 (ppm)

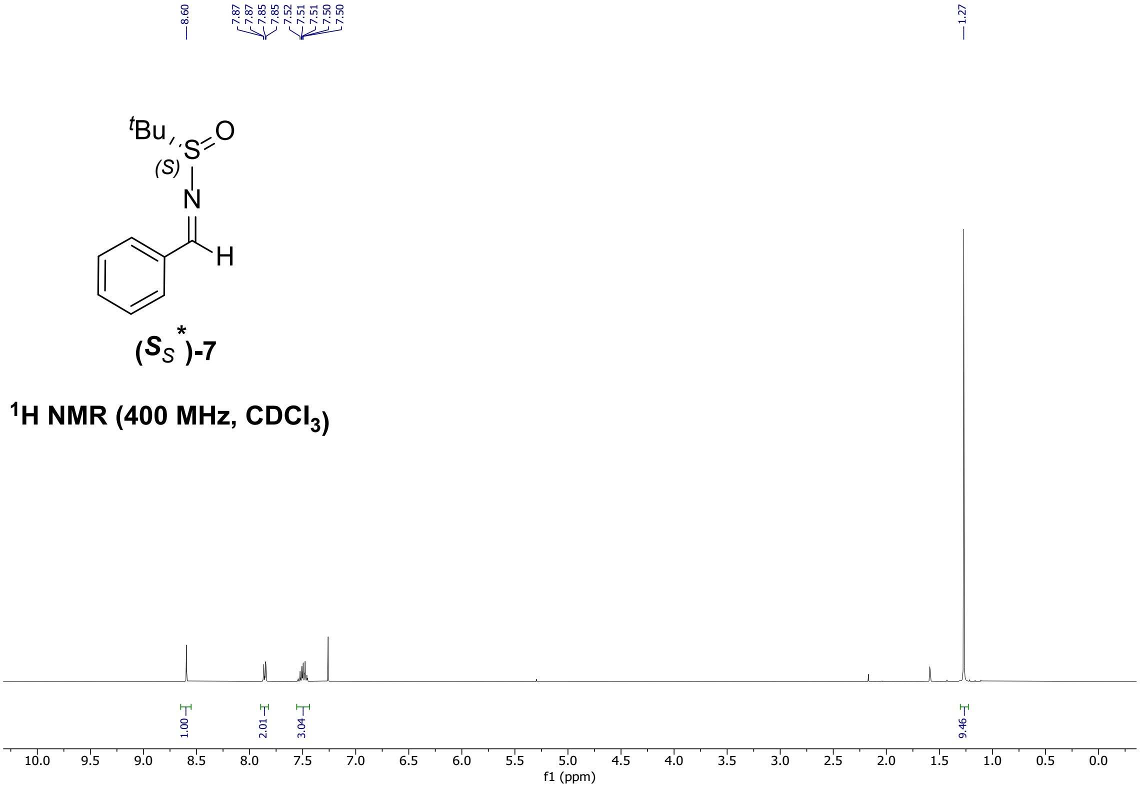
8.60
7.87
7.87
7.85
7.85
7.52
7.51
7.51
7.50
7.50
1.27
tBu
S
O
(S)
N
H
(SS*)-7
1H NMR (400 MHz, CDCl3)
1.00
2.01
3.04
9.46
10.0 9.5 9.0 8.5 8.0 7.5 7.0 6.5 6.0 5.5 5.0 4.5 4.0 3.5 3.0 2.5 2.0 1.5 1.0 0.5 0.0
f1 (ppm)

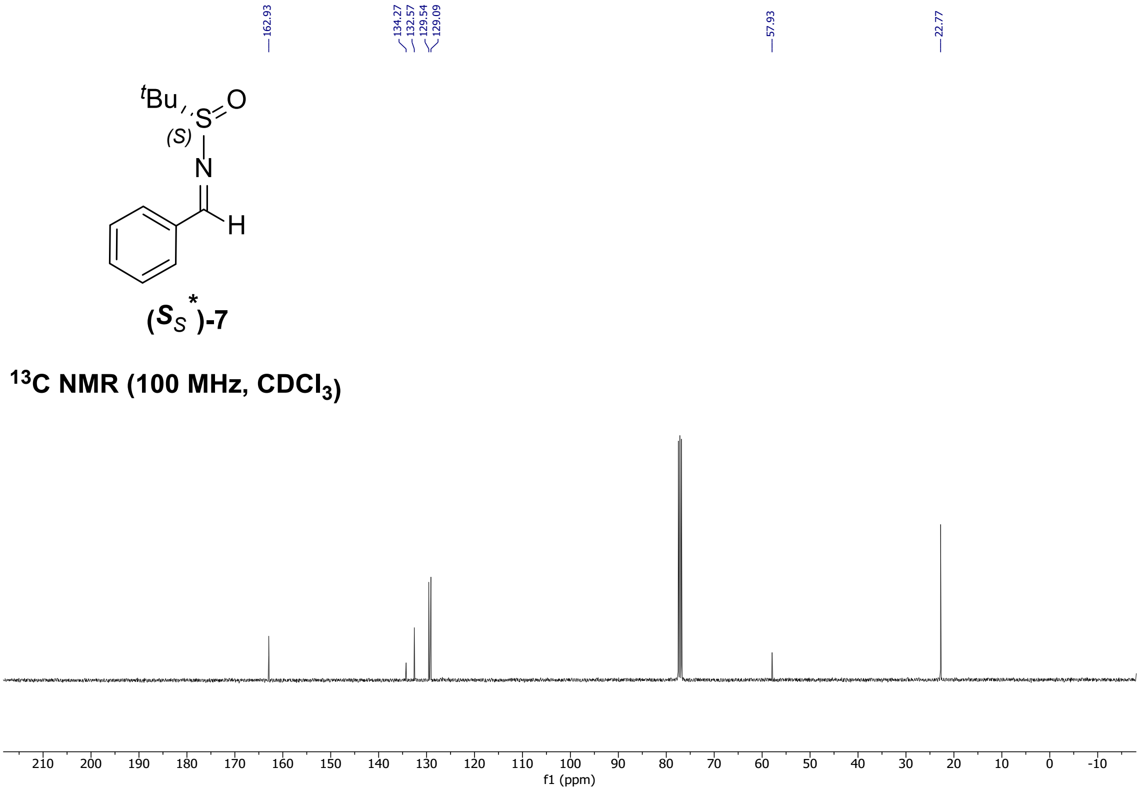
162.93
134.27
132.57
129.54
129.09
57.93
22.77
tBu
S
O
(S)
N
H
(SS*)-7
13C NMR (100 MHz, CDCl3)
210 200 190 180 170 160 150 140 130 120 110 100 90 80 70 60 50 40 30 20 10 0 -10
f1 (ppm)

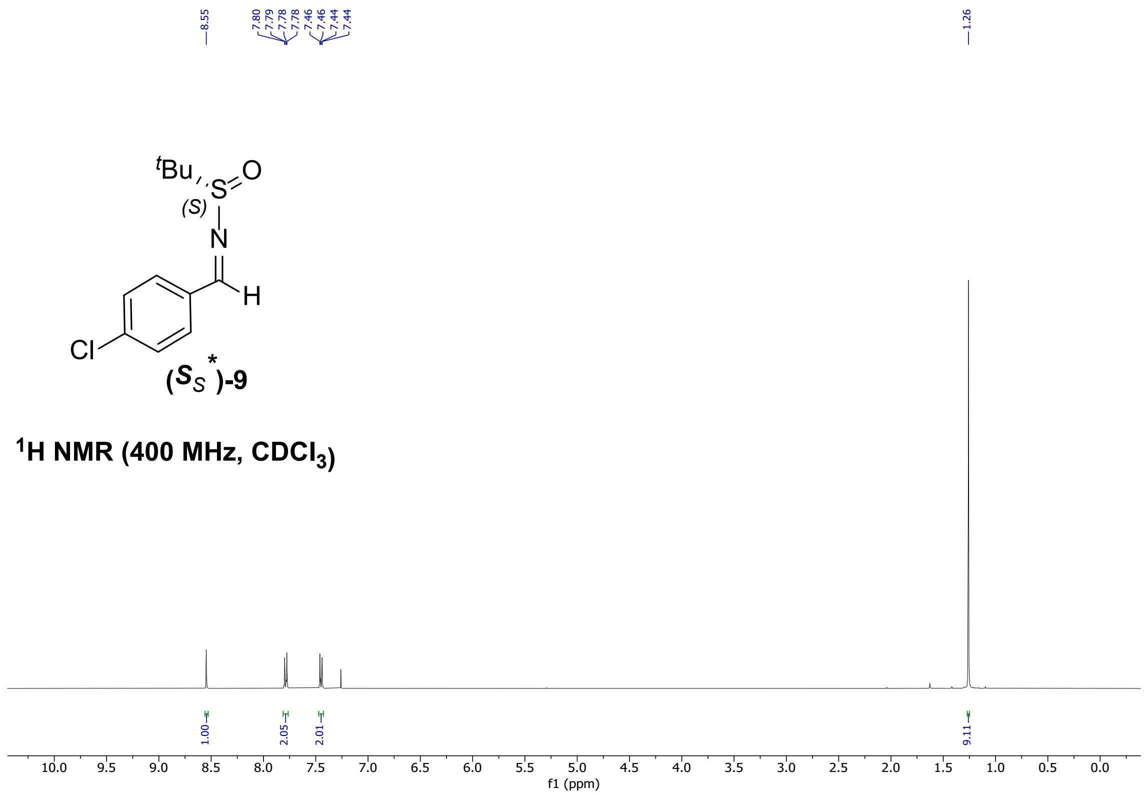
8.55
7.80
7.79
7.78
7.78
7.46
7.46
7.44
7.44
1.26
^tBu
(S)
(S$_S$*)-9
^{1}H NMR (400 MHz, CDCl$_3$)
1.00
2.05
2.01
9.11
f1 (ppm)

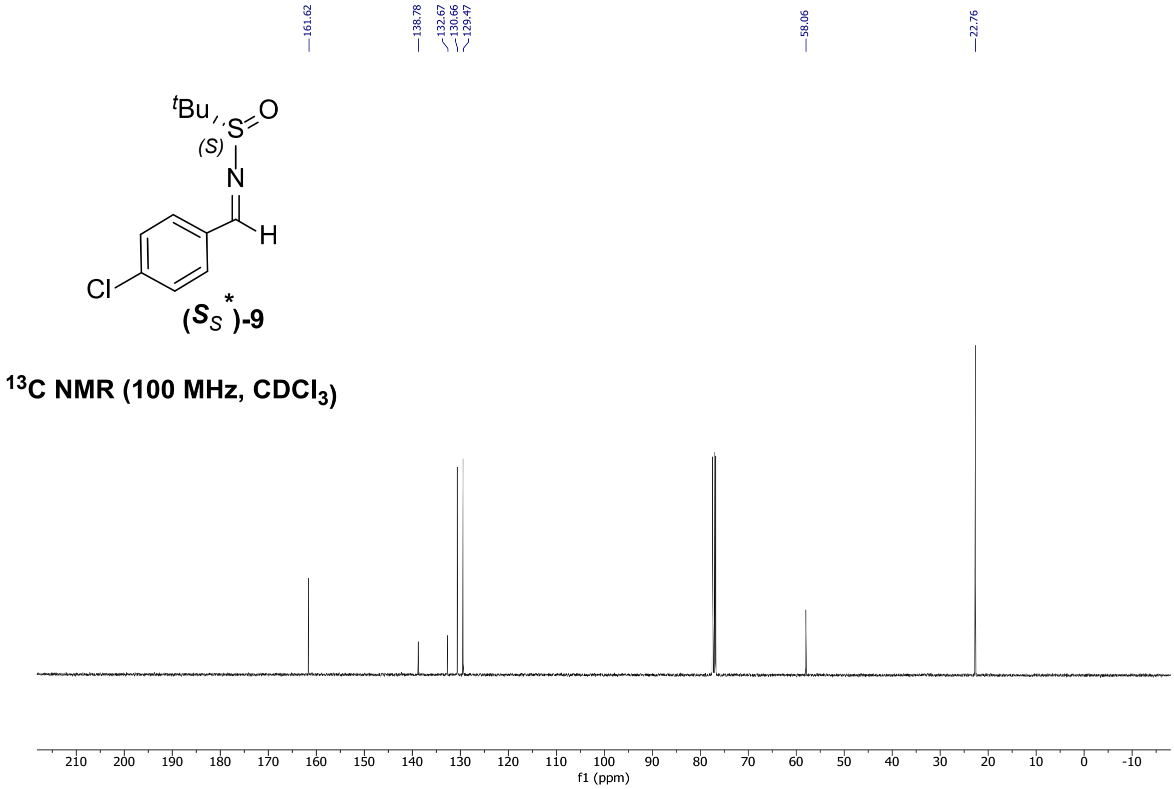
161.62
138.78
132.67
130.66
129.47
58.06
22.76
^tBu
(S)
(S$_S$*)-9
^{13}C NMR (100 MHz, CDCl$_3$)
f1 (ppm)

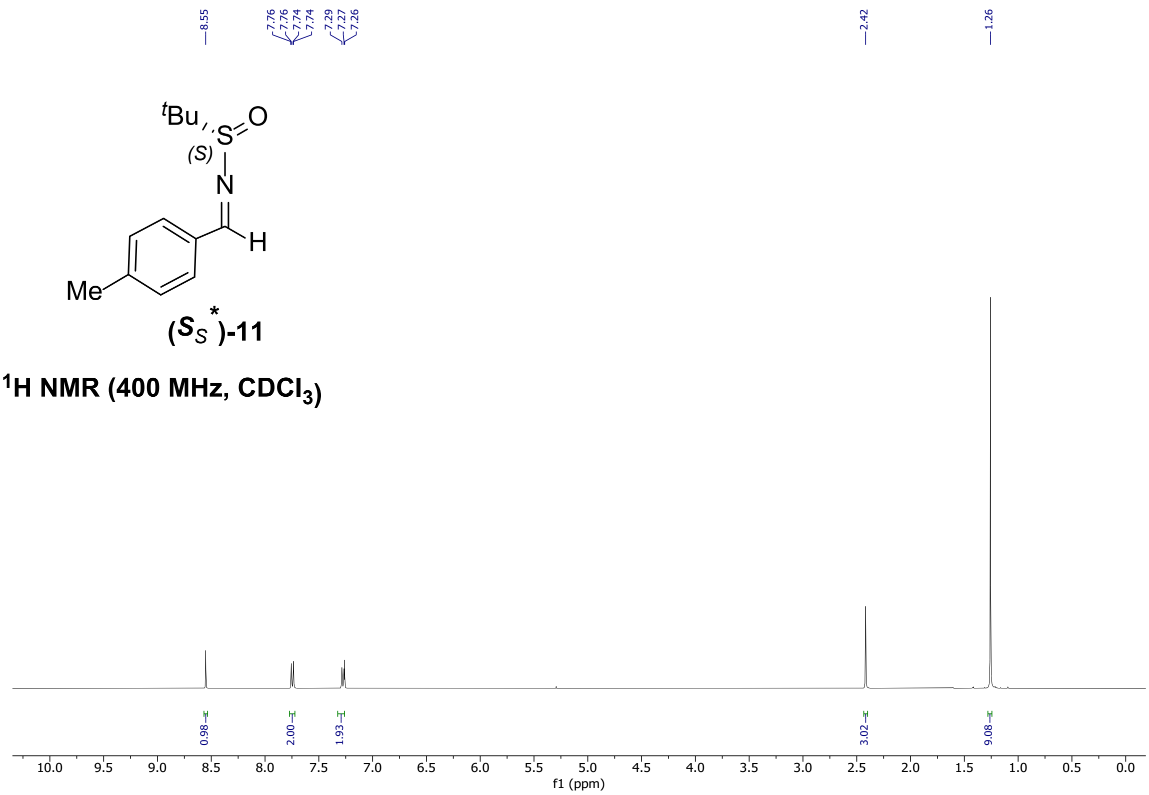
8.55
7.76
7.76
7.74
7.74
7.29
7.27
7.26
2.42
1.26
tBu
S
O
(S)
N
H
Me
(SS*)-11
1H NMR (400 MHz, CDCl3)
0.98
2.00
1.93
3.02
9.08
10.0
9.5
9.0
8.5
8.0
7.5
7.0
6.5
6.0
5.5
5.0
4.5
4.0
3.5
3.0
2.5
2.0
1.5
1.0
0.5
0.0
f1 (ppm)

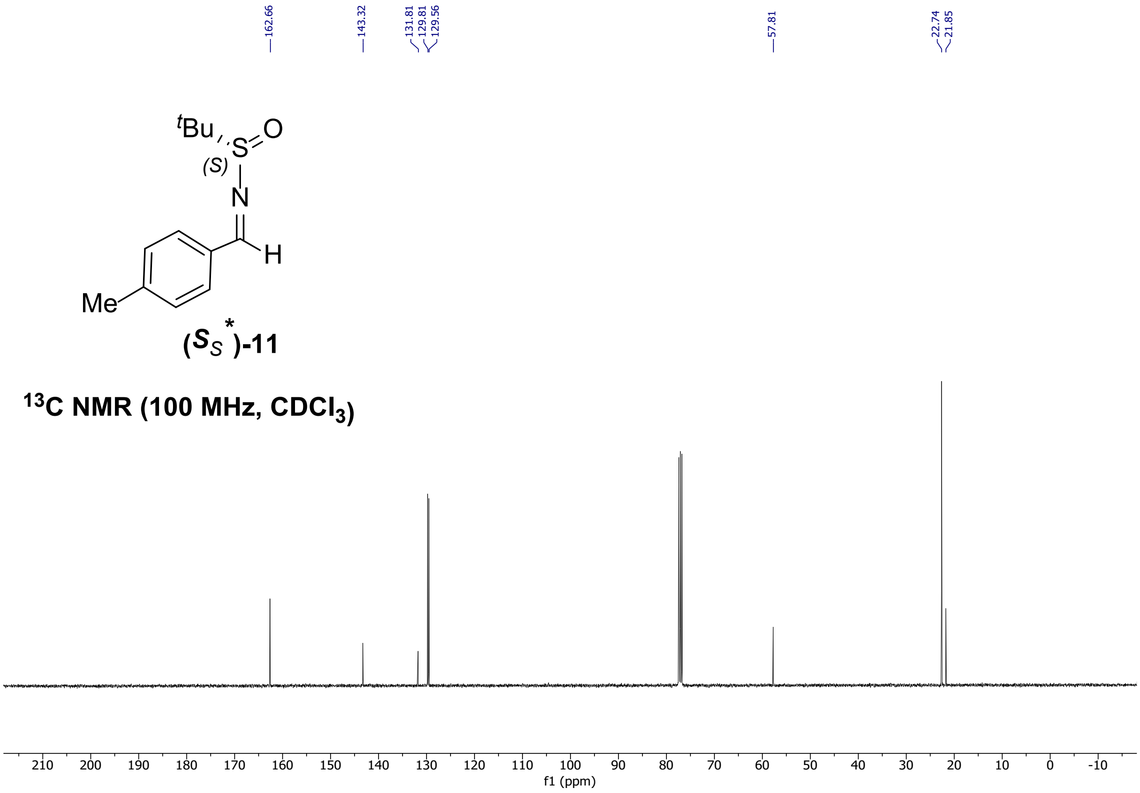
162.66
143.32
131.81
129.81
129.56
57.81
22.74
21.85
tBu
S
O
(S)
N
H
Me
(SS*)-11
13C NMR (100 MHz, CDCl3)
210
200
190
180
170
160
150
140
130
120
110
100
90
80
70
60
50
40
30
20
10
0
-10
f1 (ppm)

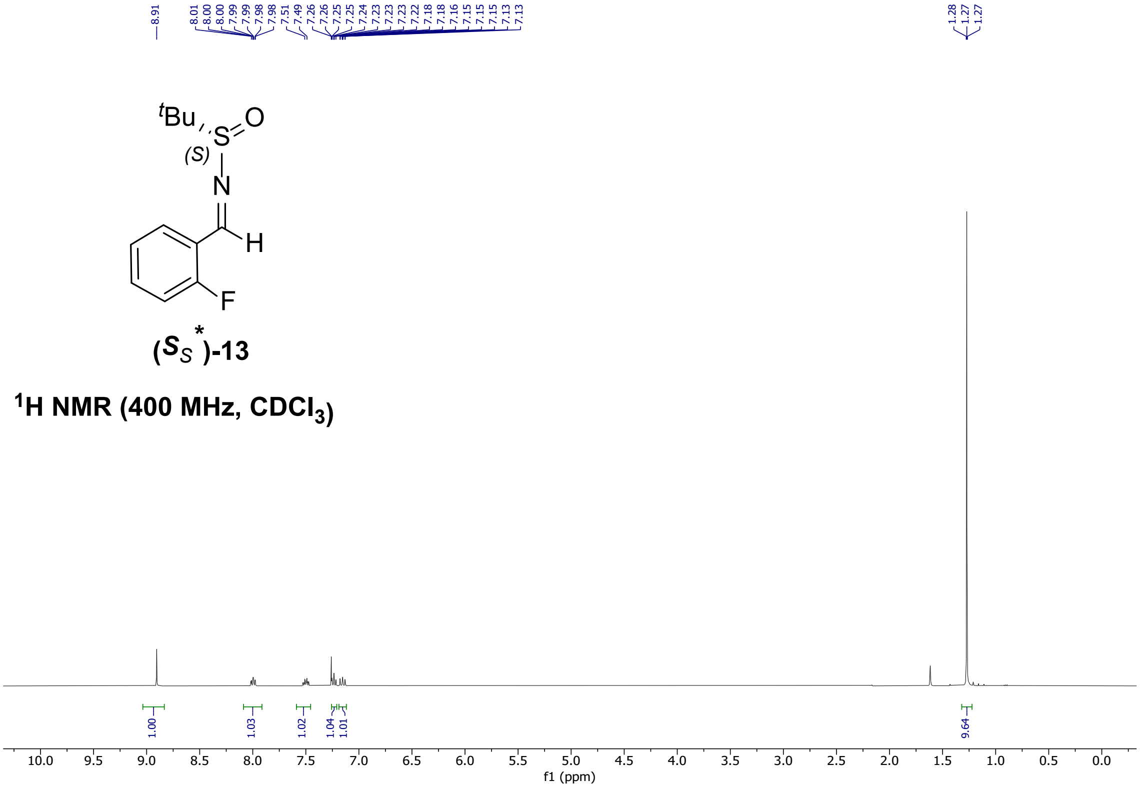
tBu
(S)
S
O
N
H
F
(SS*)-13
1H NMR (400 MHz, CDCl3)
8.91
1.28
1.27
1.27
1.00
1.03
1.02
1.04
1.01
9.64
f1 (ppm)

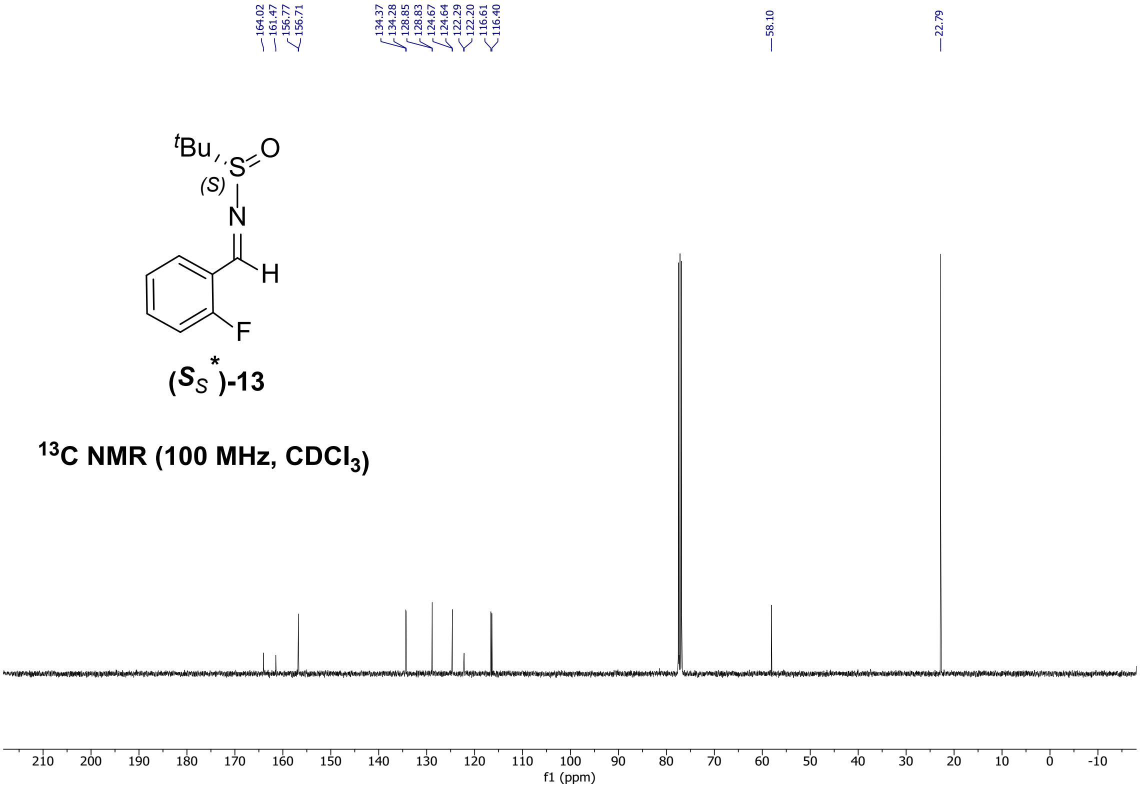
164.02
161.47
156.77
156.71
134.37
134.28
128.85
128.83
124.67
124.64
122.29
122.20
116.61
116.40
58.10
22.79
tBu
(S)
S
O
N
H
F
(SS*)-13
13C NMR (100 MHz, CDCl3)
f1 (ppm)

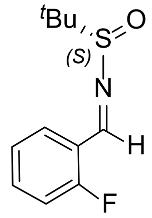

(S_S*)-13

^{19}F NMR (377 MHz, $CDCl_3$)

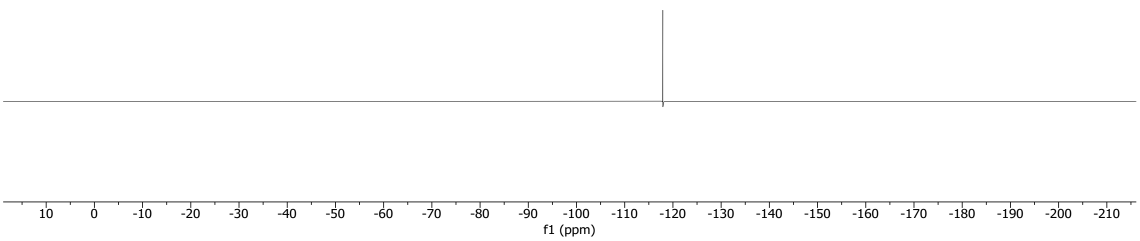

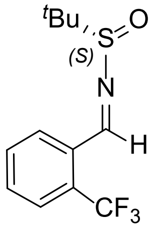

(S_S*)-15

^{1}H NMR (400 MHz, $CDCl_3$)

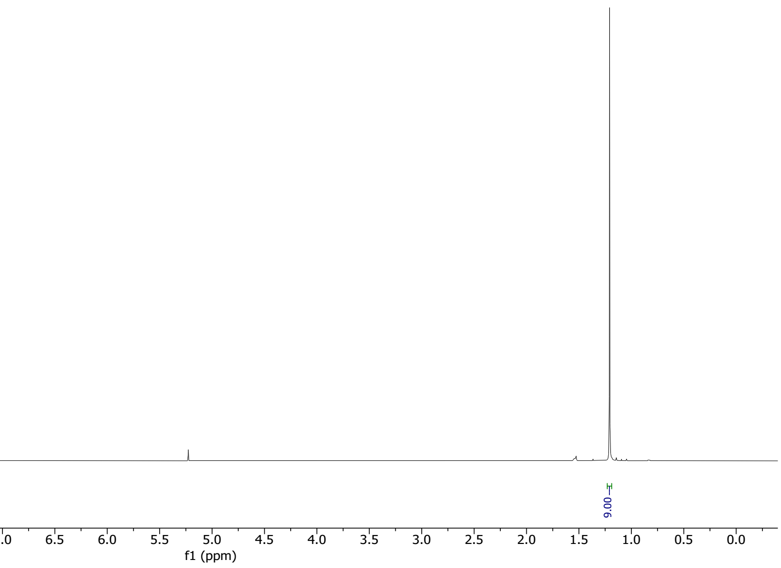

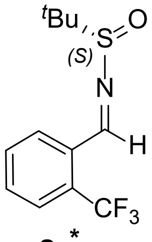

(S_S*)-15

^{13}C NMR (100 MHz, $CDCl_3$)

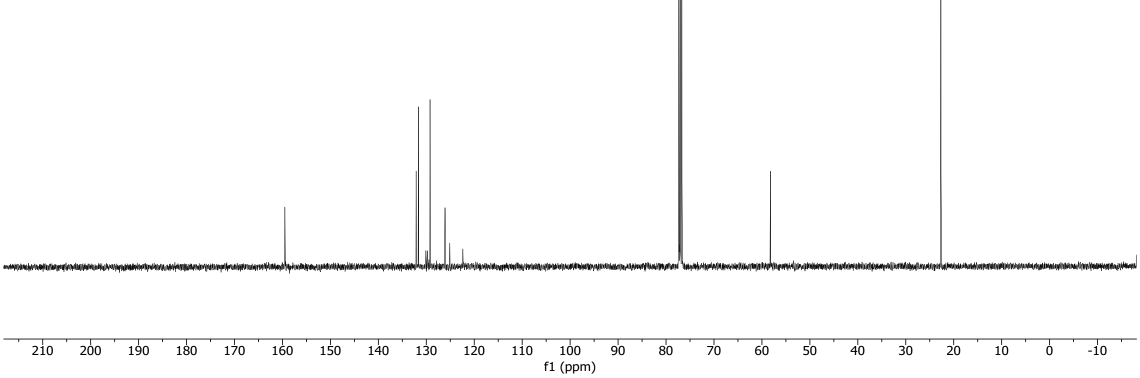

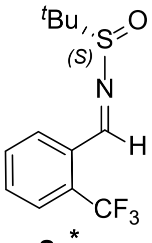

(S_S*)-15

^{19}F NMR (377 MHz, $CDCl_3$)

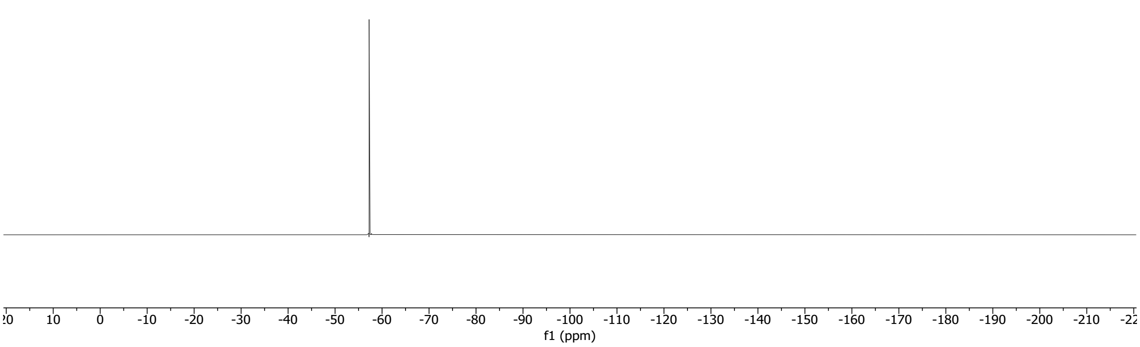

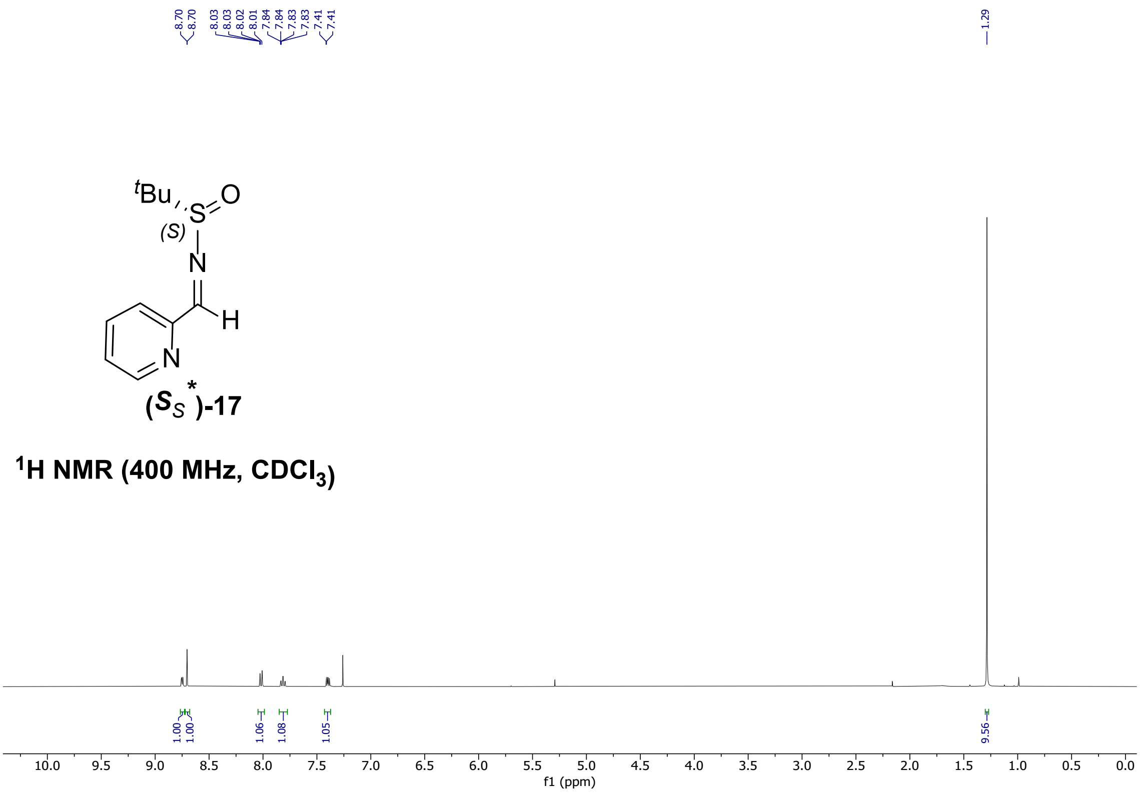
8.70 8.70 8.03 8.03 8.02 8.01 7.84 7.84 7.83 7.83 7.41 7.41
1.29
tBu
S
O
(S)
N
H
N
(SS*)-17
1H NMR (400 MHz, CDCl3)
1.00 1.00 1.06 1.08 1.05 9.56
f1 (ppm)

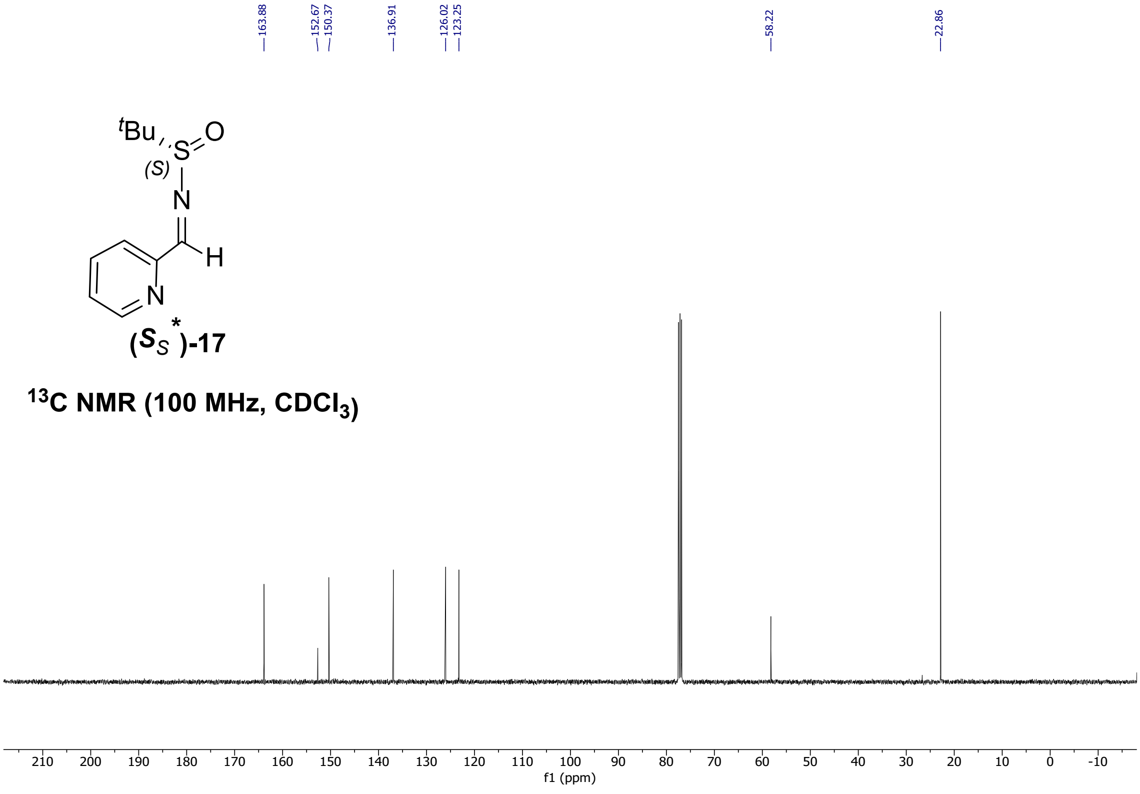
163.88 152.67 150.37 136.91 126.02 123.25 58.22 22.86
tBu
S
O
(S)
N
H
N
(SS*)-17
13C NMR (100 MHz, CDCl3)
f1 (ppm)

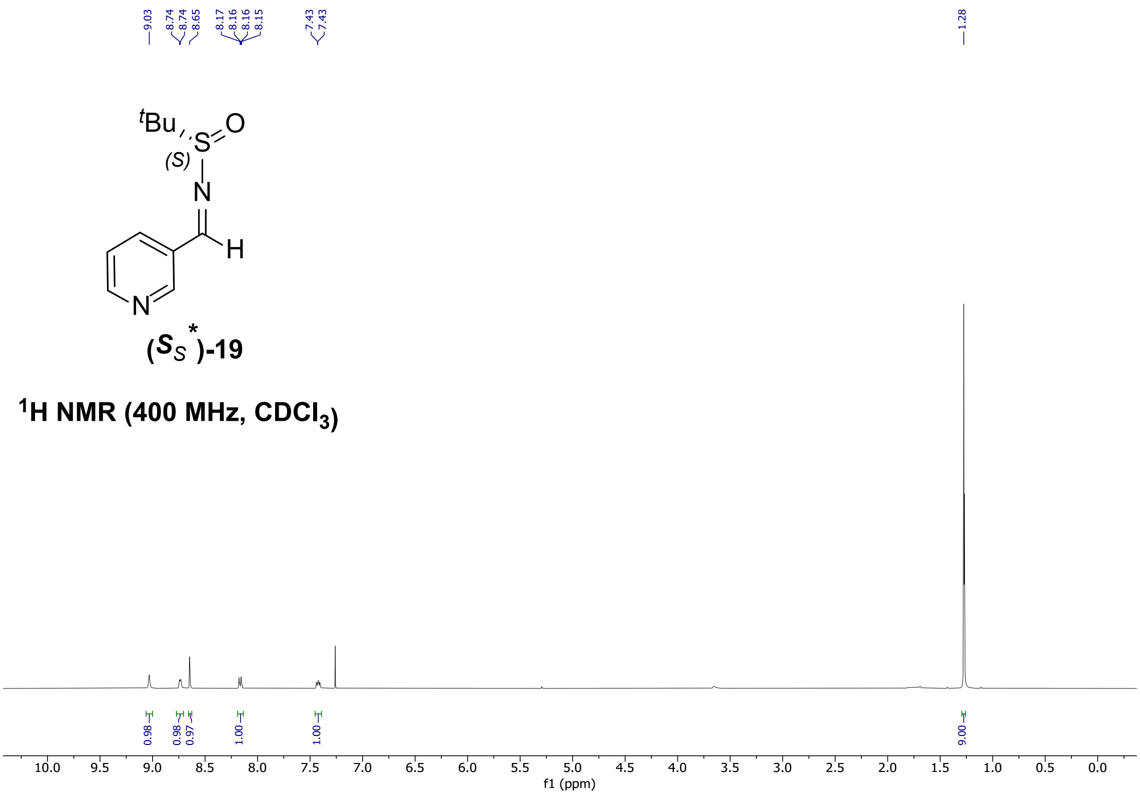

(S_S*)-19

^{1}H NMR (400 MHz, $CDCl_3$)

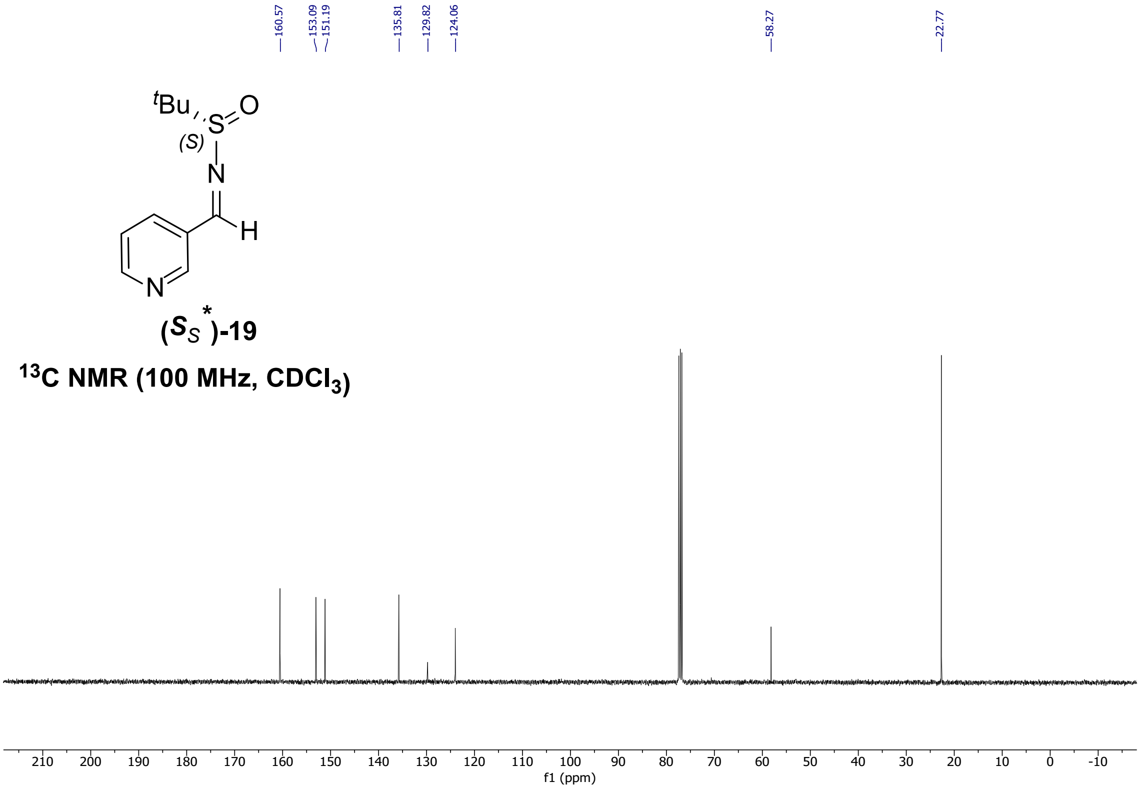

(S_S*)-19

^{13}C NMR (100 MHz, $CDCl_3$)

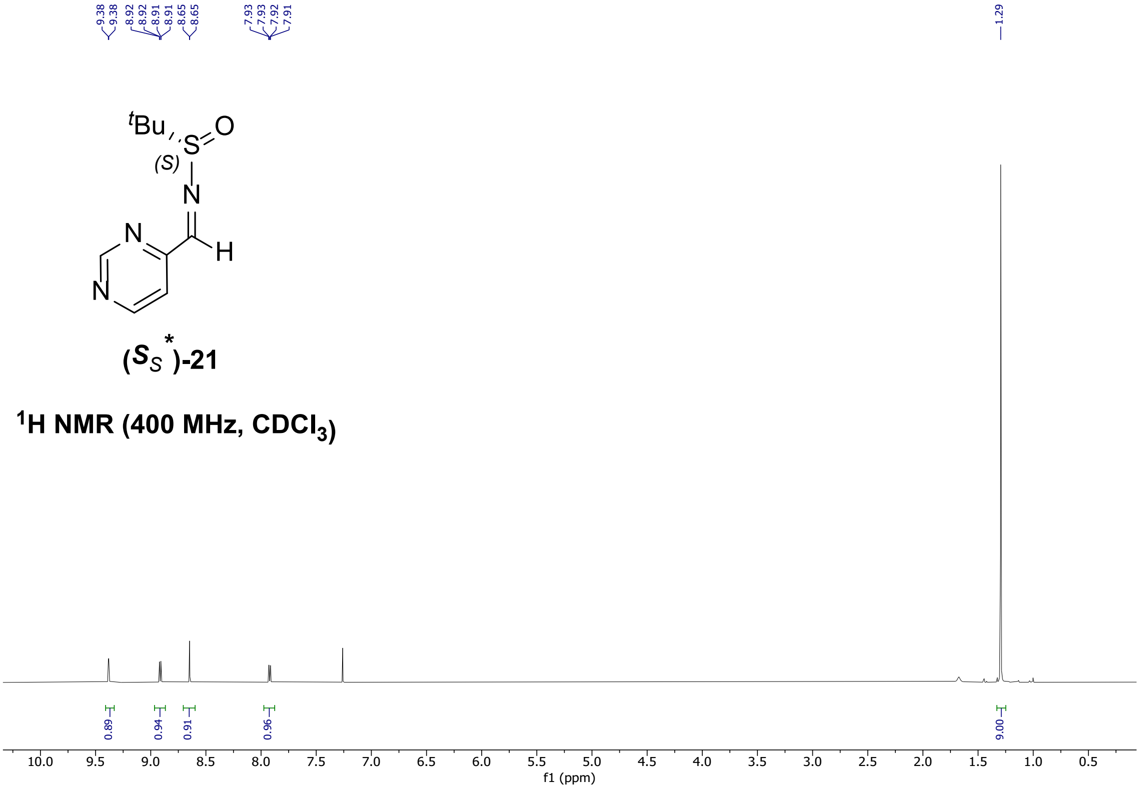

(S_S*)-21

^{1}H NMR (400 MHz, $CDCl_3$)

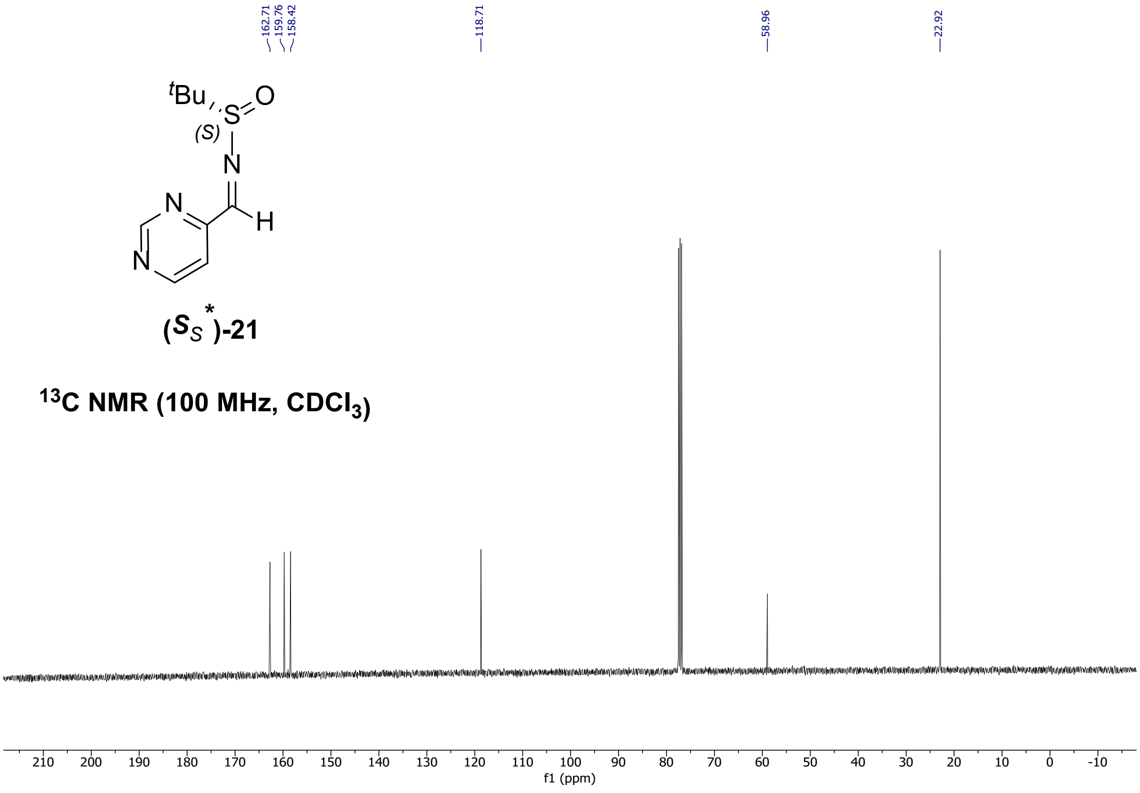

(S_S*)-21

^{13}C NMR (100 MHz, $CDCl_3$)

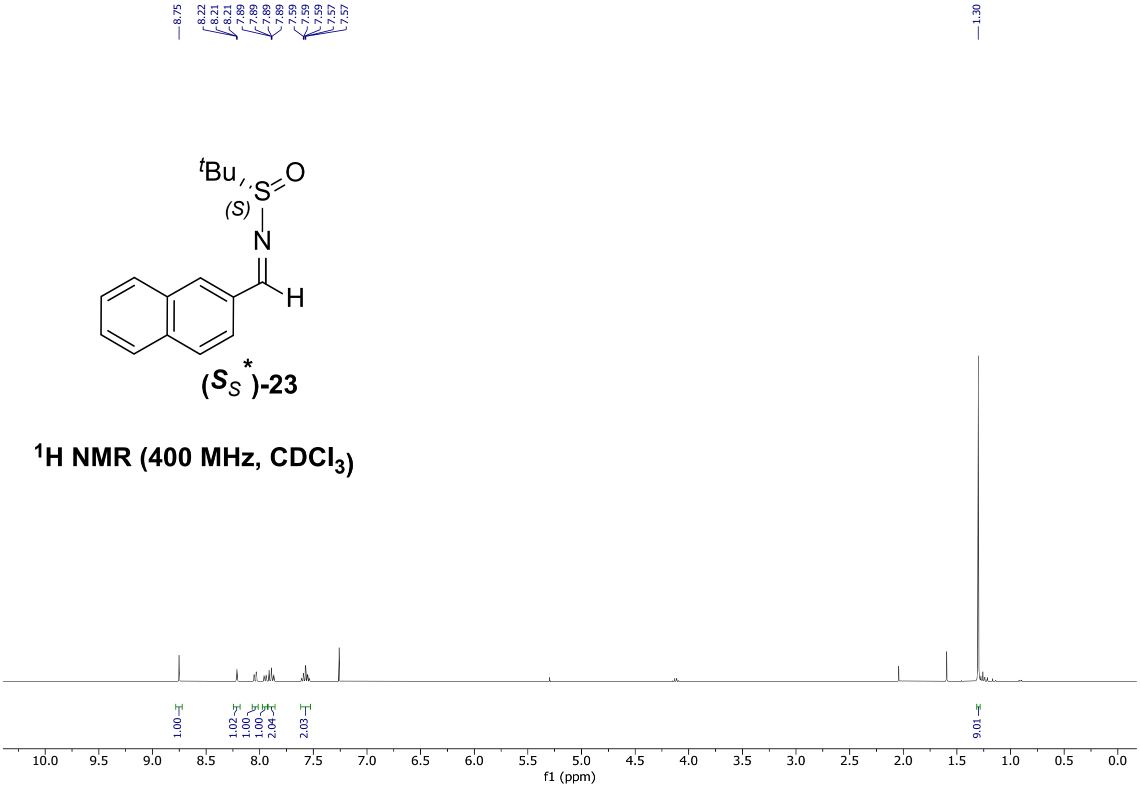

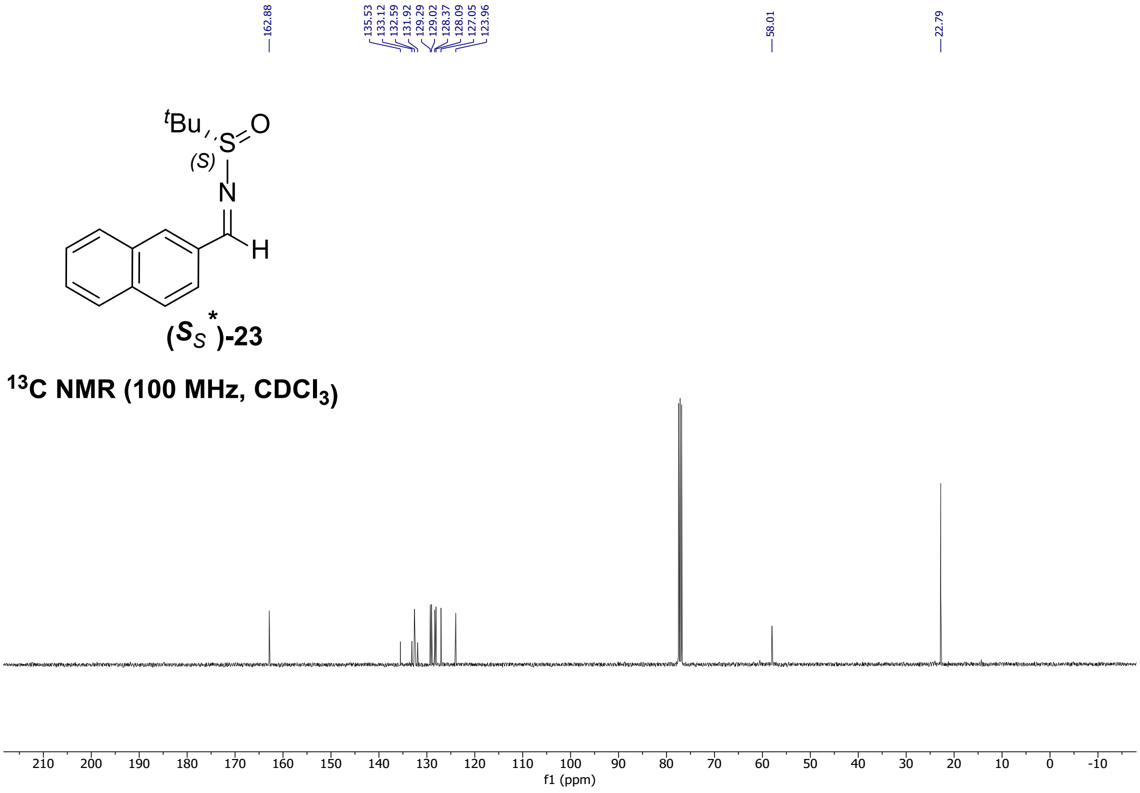

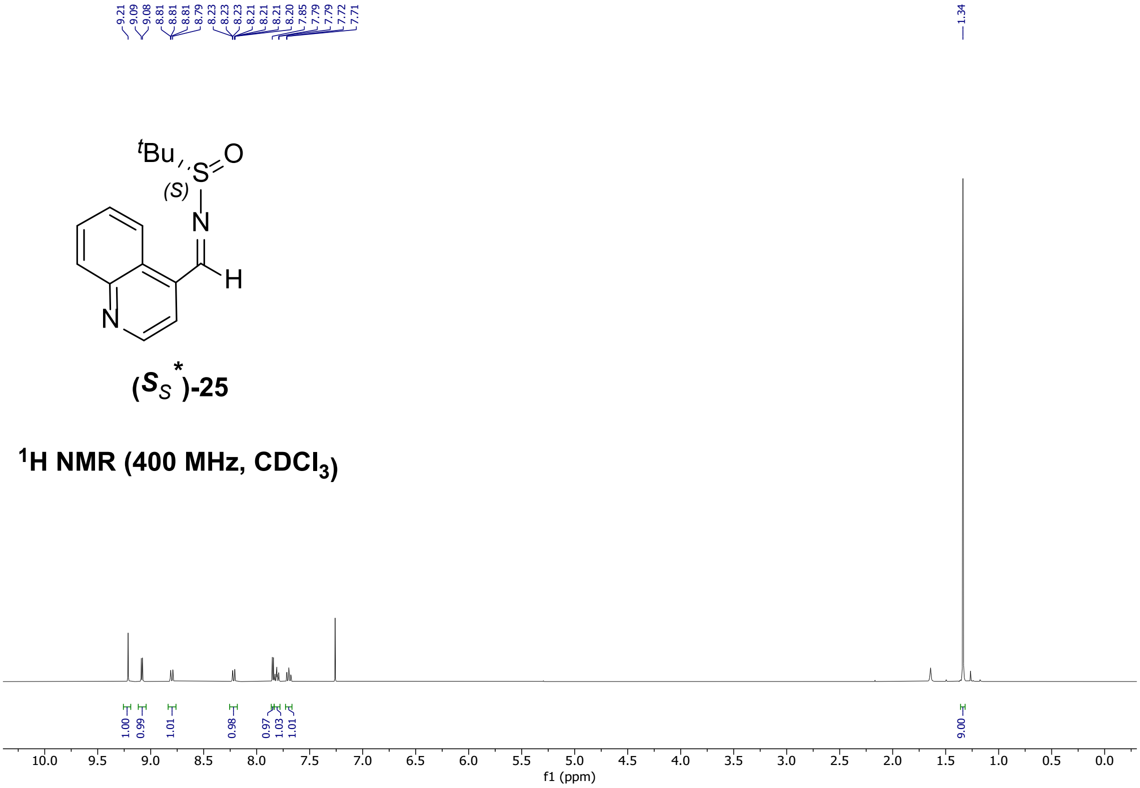

(S_S*)-25

^{1}H NMR (400 MHz, CDCl$_3$)

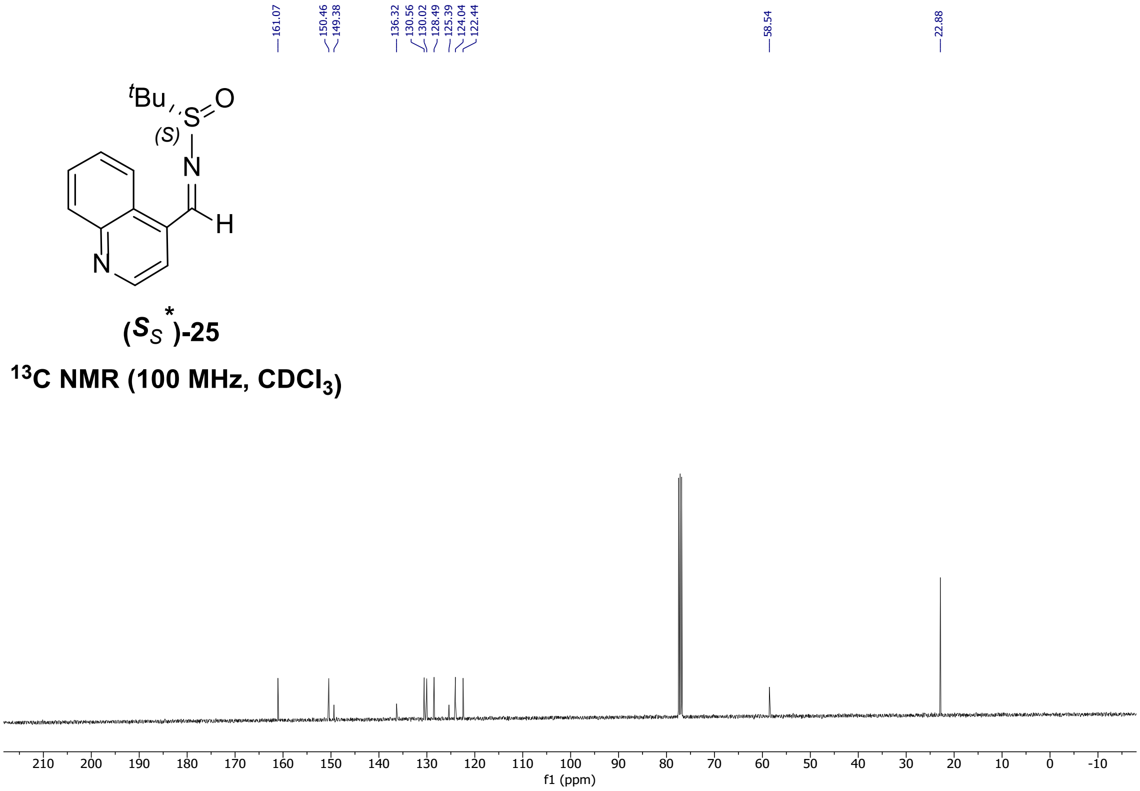

(S_S*)-25

^{13}C NMR (100 MHz, CDCl$_3$)

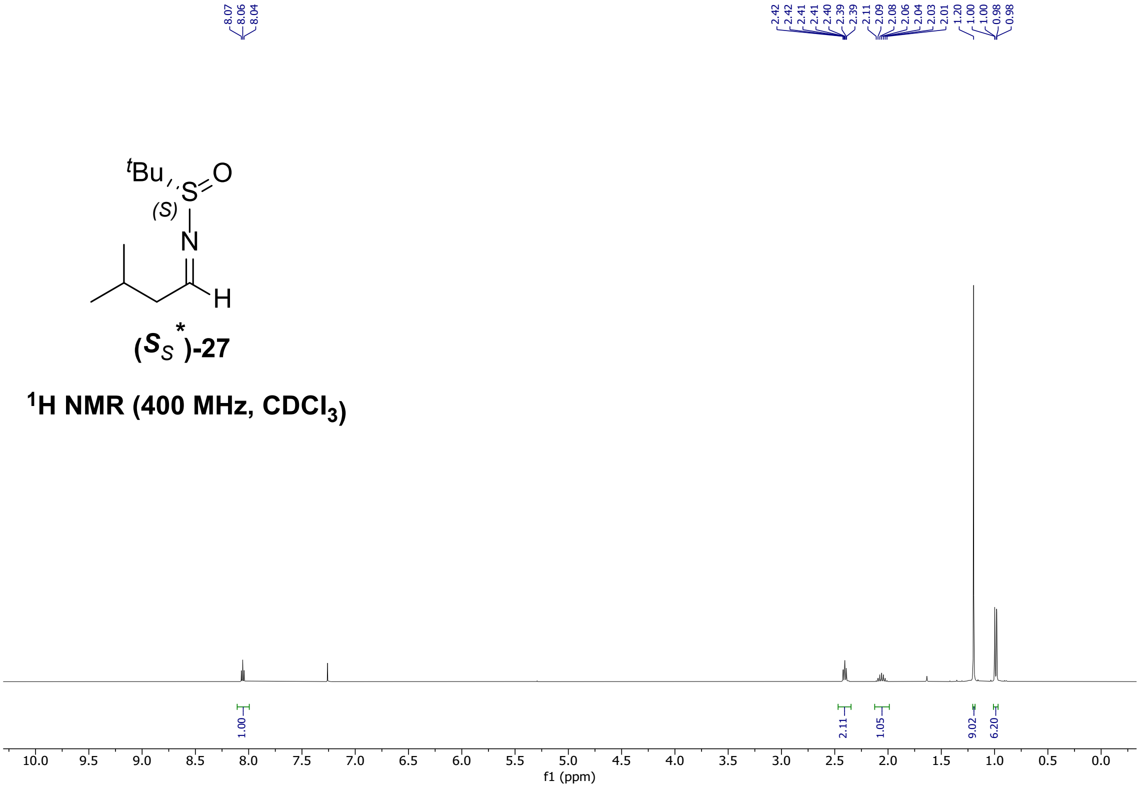

tBu
(S)
S
O
N
H
(S_S*)-27
¹H NMR (400 MHz, CDCl₃)
8.07
8.06
8.04
2.42
2.42
2.41
2.41
2.40
2.39
2.39
2.11
2.09
2.08
2.06
2.04
2.03
2.01
1.20
1.00
1.00
0.98
0.98
1.00
2.11
1.05
9.02
6.20
f1 (ppm)

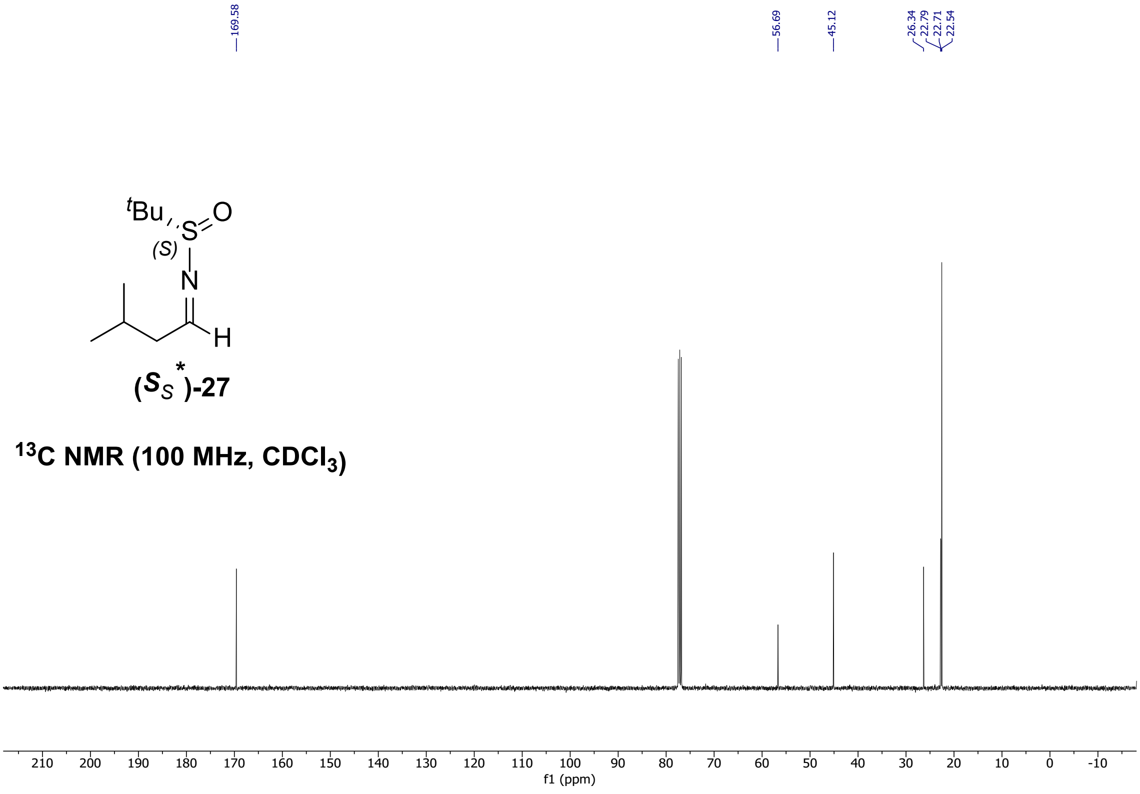

tBu
(S)
S
O
N
H
(S_S*)-27
¹³C NMR (100 MHz, CDCl₃)
169.58
56.69
45.12
26.34
22.79
22.71
22.54
f1 (ppm)

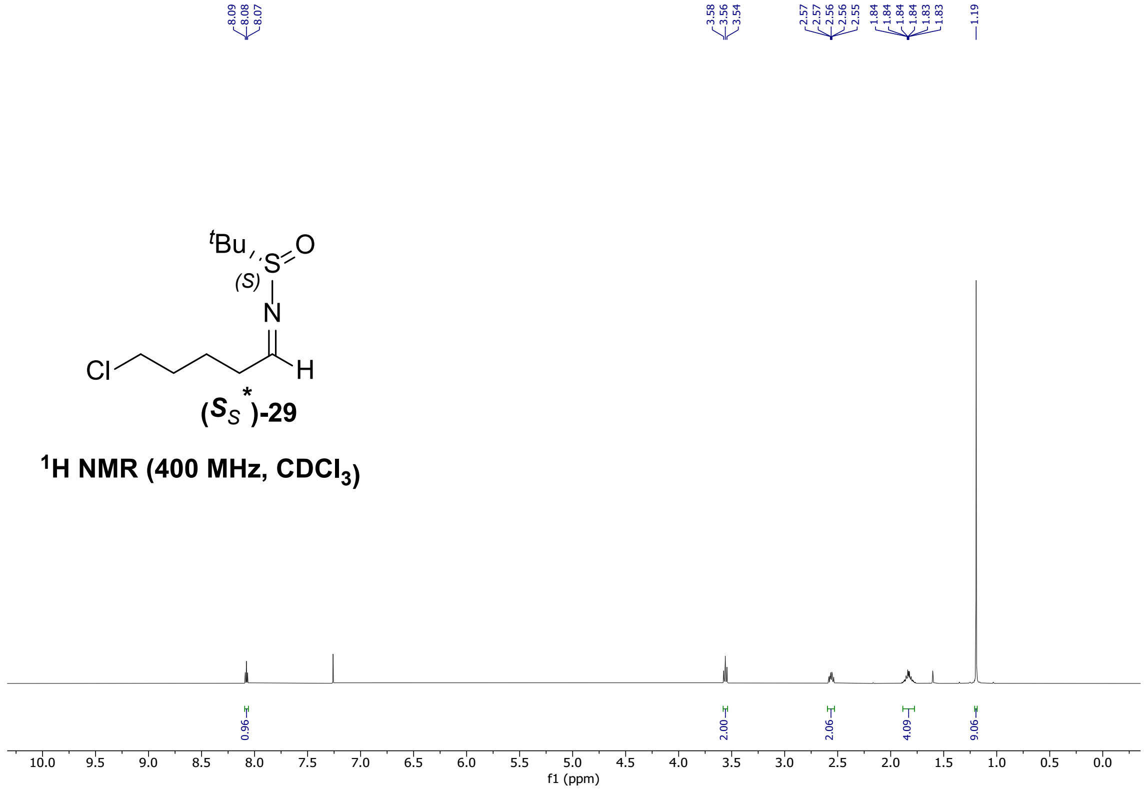
8.09
8.08
8.07
3.58
3.56
3.54
2.57
2.57
2.56
2.56
2.55
1.84
1.84
1.84
1.84
1.83
1.83
1.19
tBu
S
O
(S)
N
Cl
H
(S_S*)-29
^{1}H NMR (400 MHz, $CDCl_3$)
0.96
2.00
2.06
4.09
9.06
10.0
9.5
9.0
8.5
8.0
7.5
7.0
6.5
6.0
5.5
5.0
4.5
4.0
3.5
3.0
2.5
2.0
1.5
1.0
0.5
0.0
f1 (ppm)

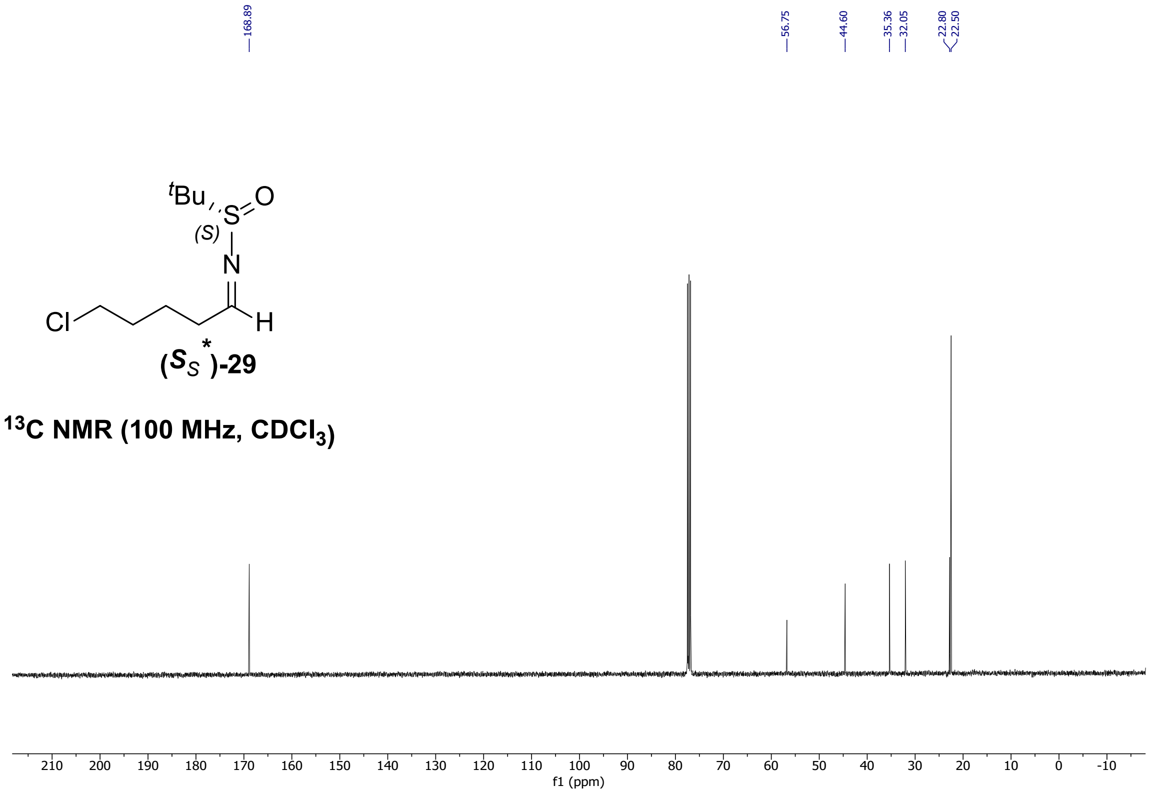
168.89
56.75
44.60
35.36
32.05
22.80
22.50
tBu
S
O
(S)
N
Cl
H
(S_S*)-29
^{13}C NMR (100 MHz, $CDCl_3$)
210
200
190
180
170
160
150
140
130
120
110
100
90
80
70
60
50
40
30
20
10
0
-10
f1 (ppm)

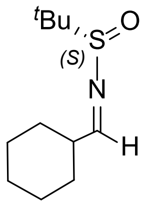

(S_S*)-31

^{1}H NMR (400 MHz, $CDCl_3$)

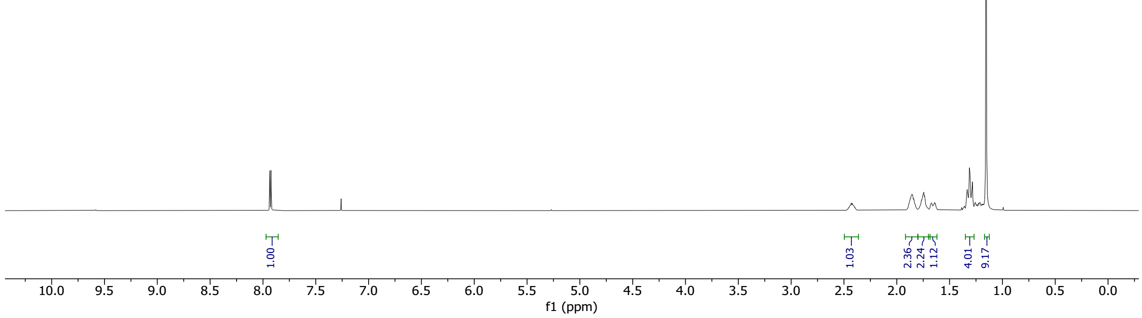

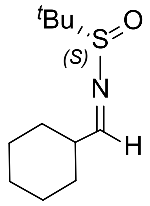

(S_S*)-31

^{13}C NMR (100 MHz, $CDCl_3$)

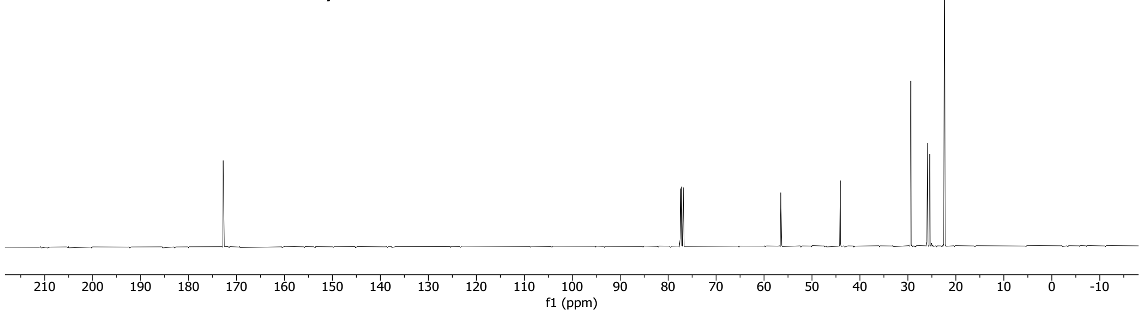

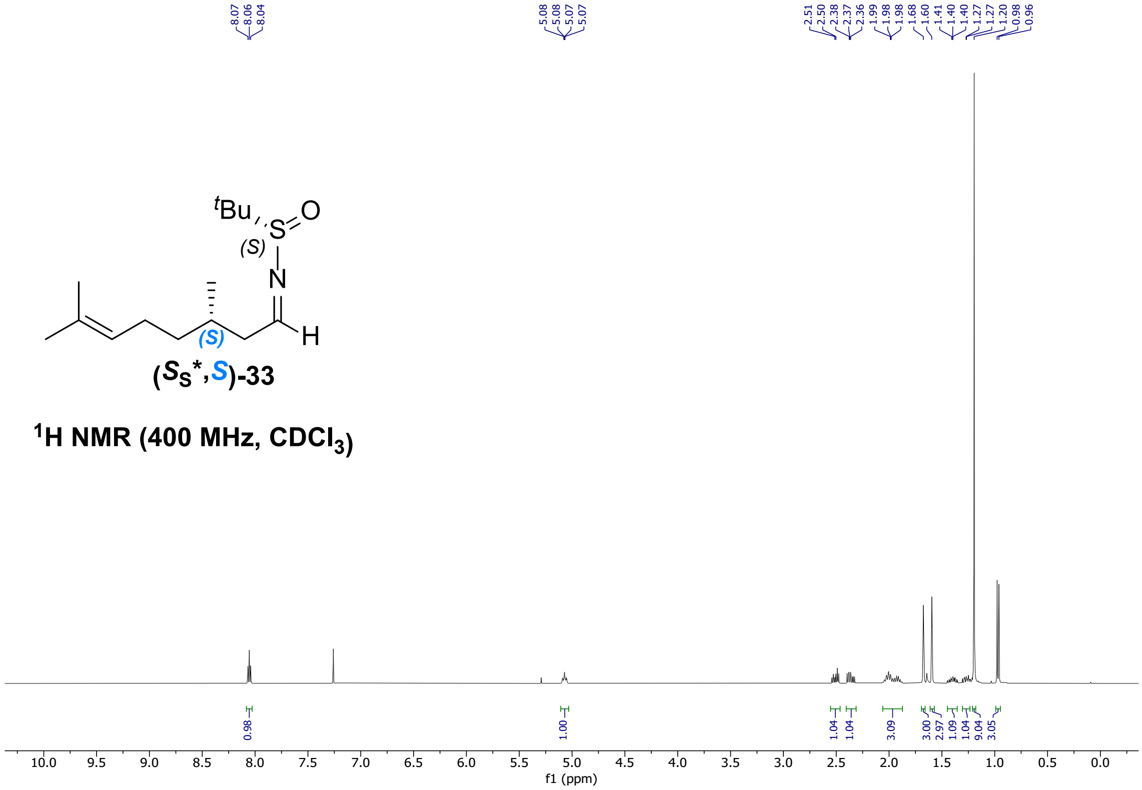

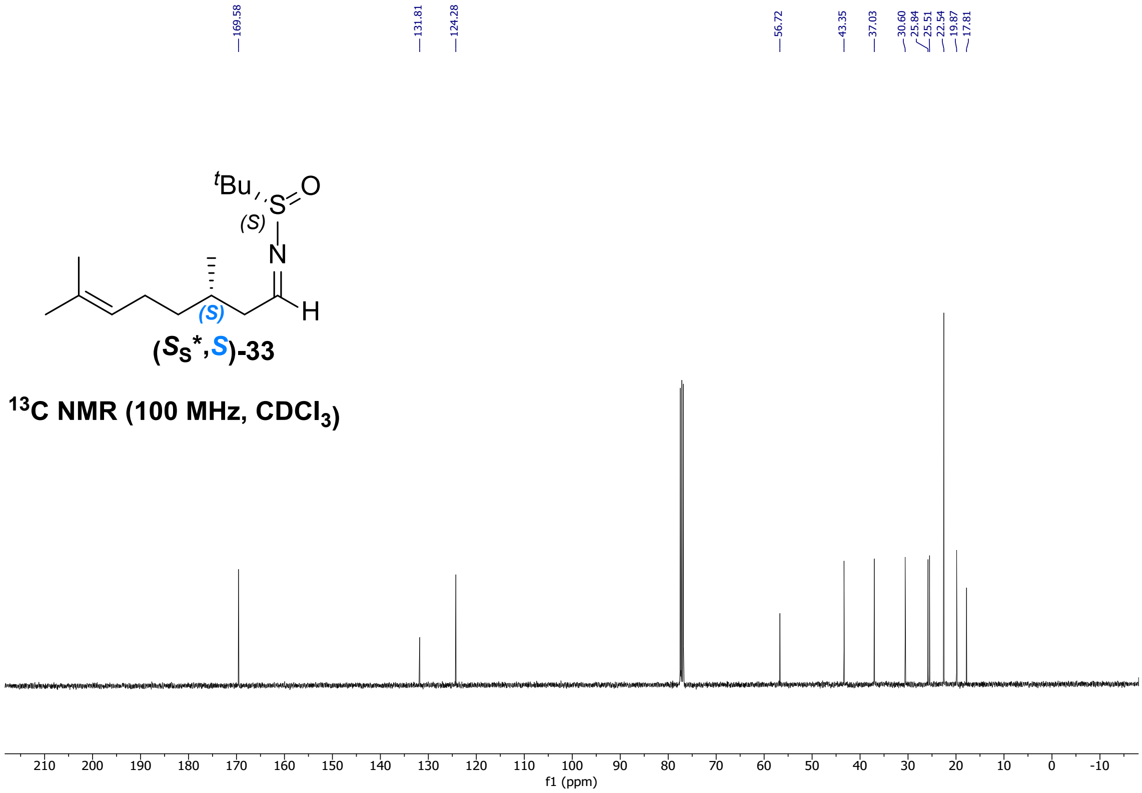

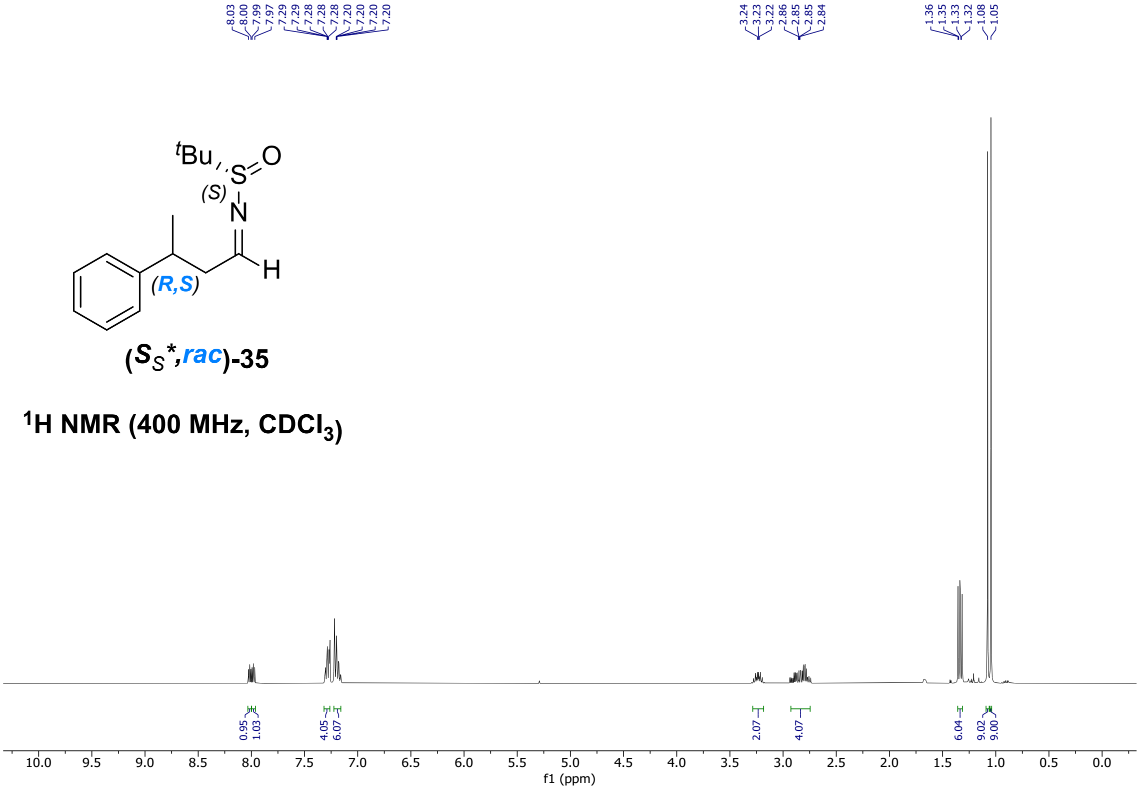
8.03
8.00
7.99
7.97
7.29
7.29
7.28
7.28
7.28
7.20
7.20
7.20
7.20
3.24
3.23
3.22
2.86
2.85
2.85
2.84
1.36
1.35
1.33
1.32
1.08
1.05
tBu
S
O
(S)
N
H
(R,S)
(S_S*,rac)-35
1H NMR (400 MHz, CDCl3)
0.95
1.03
4.05
6.07
2.07
4.07
6.04
9.02
9.00
f1 (ppm)

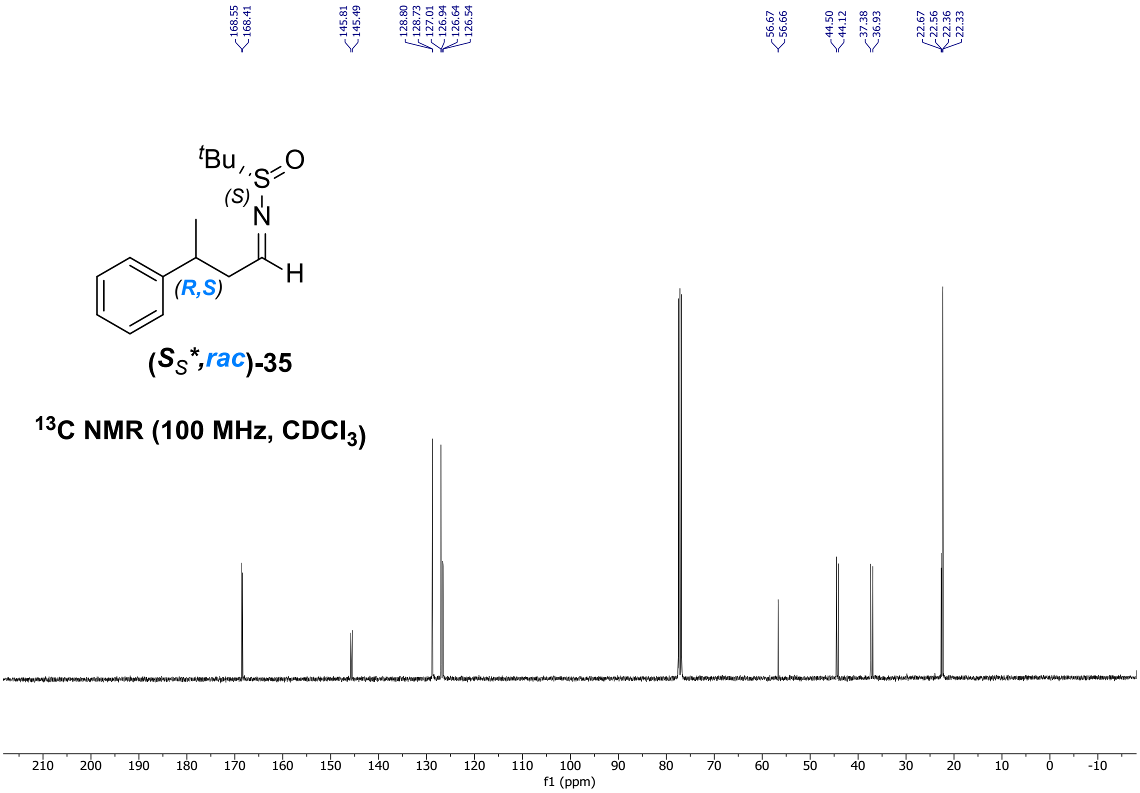
168.55
168.41
145.81
145.49
128.80
128.73
127.01
126.94
126.64
126.54
56.67
56.66
44.50
44.12
37.38
36.93
22.67
22.56
22.36
22.33
tBu
S
O
(S)
N
H
(R,S)
(S_S*,rac)-35
13C NMR (100 MHz, CDCl3)
f1 (ppm)

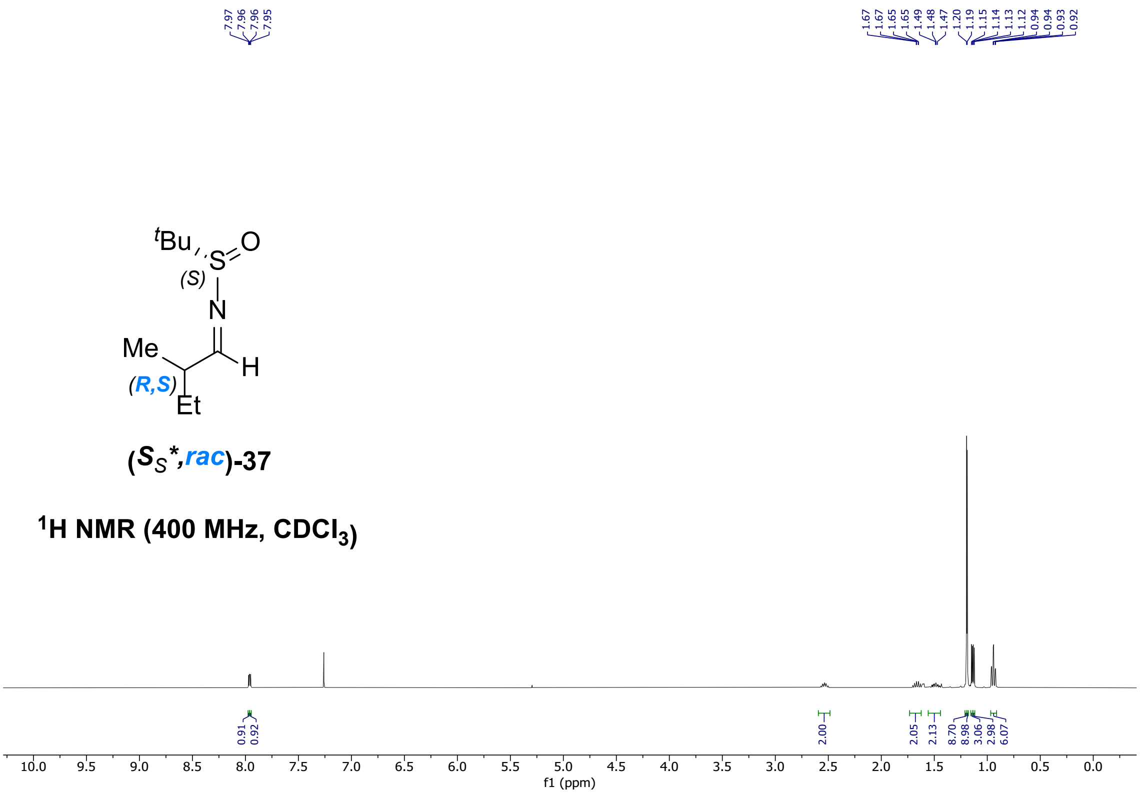
7.97
7.96
7.96
7.95
1.67
1.67
1.65
1.65
1.49
1.48
1.47
1.20
1.19
1.15
1.14
1.13
1.12
0.94
0.94
0.93
0.92
^{t}Bu
S
O
(S)
N
Me
H
(R,S)
Et
(S_S*,*rac*)-37
^{1}H NMR (400 MHz, $CDCl_3$)
0.91
0.92
2.00
2.05
2.13
8.70
8.98
3.06
2.98
6.07
10.0 9.5 9.0 8.5 8.0 7.5 7.0 6.5 6.0 5.5 5.0 4.5 4.0 3.5 3.0 2.5 2.0 1.5 1.0 0.5 0.0
f1 (ppm)

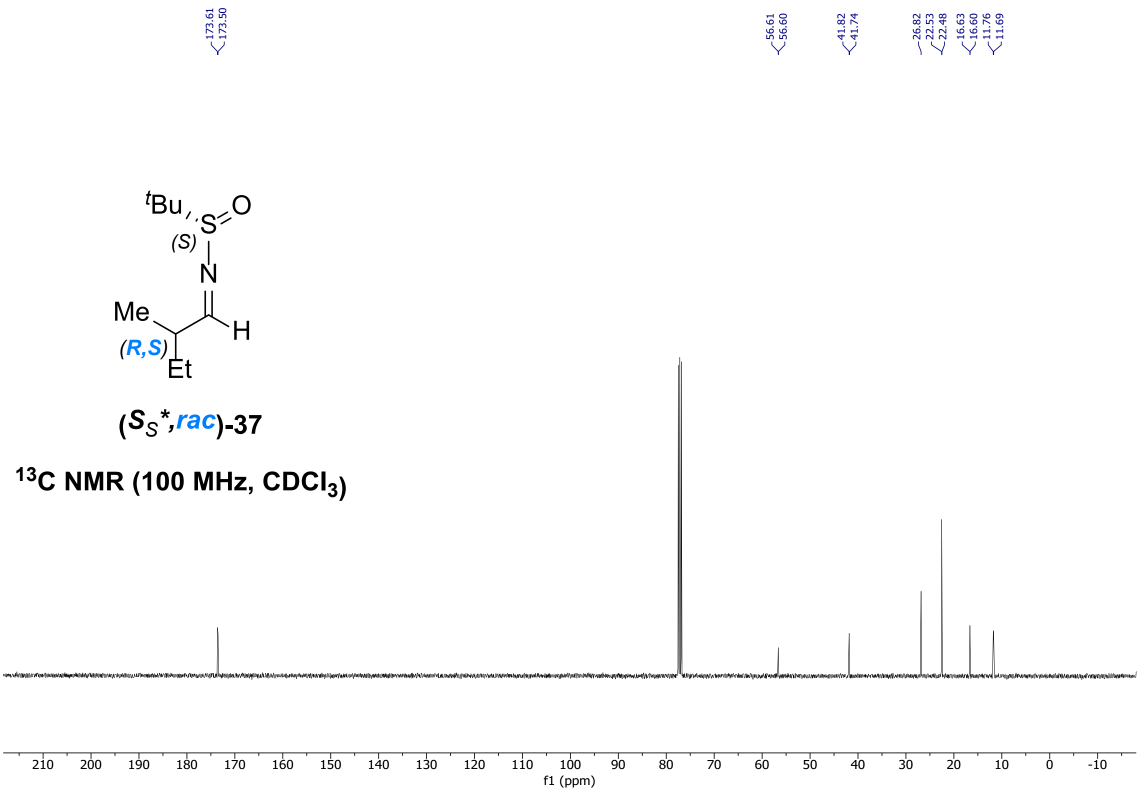
173.61
173.50
56.61
56.60
41.82
41.74
26.82
22.53
22.48
16.63
16.60
11.76
11.69
^{t}Bu
S
O
(S)
N
Me
H
(R,S)
Et
(S_S*,*rac*)-37
^{13}C NMR (100 MHz, $CDCl_3$)
210 200 190 180 170 160 150 140 130 120 110 100 90 80 70 60 50 40 30 20 10 0 -10
f1 (ppm)

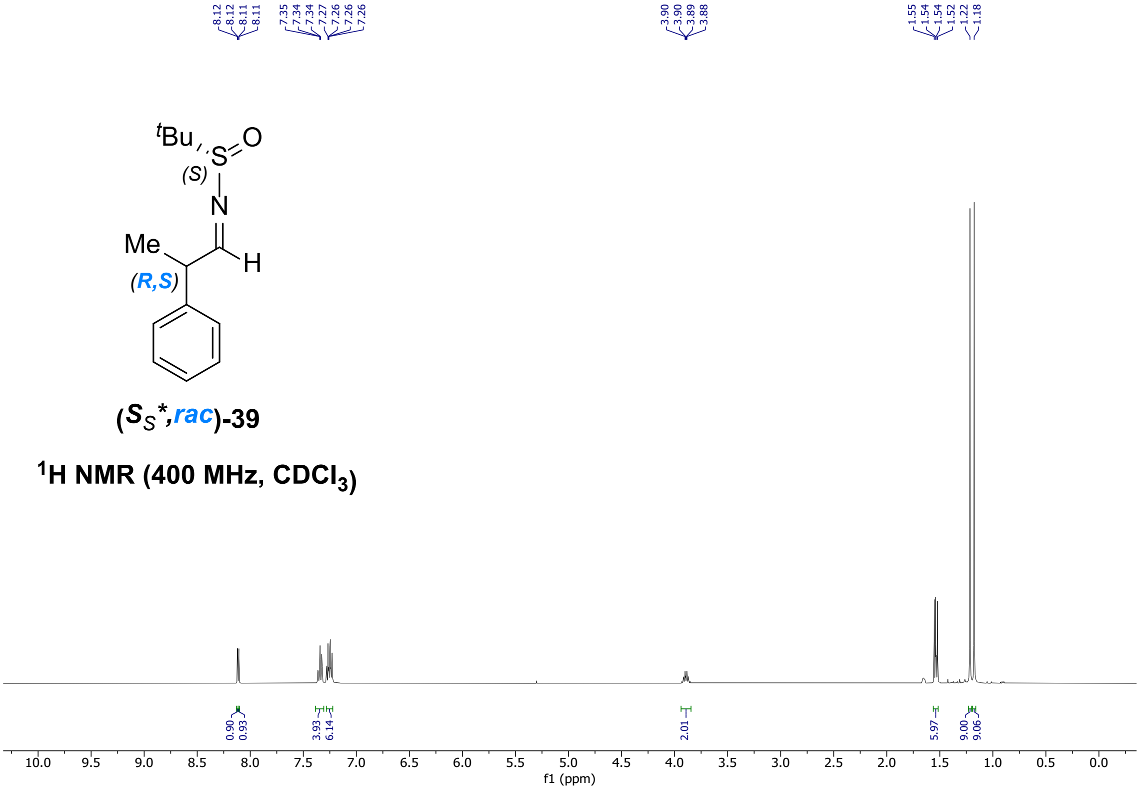

(S_S*,rac)-39
1H NMR (400 MHz, CDCl3)
f1 (ppm)

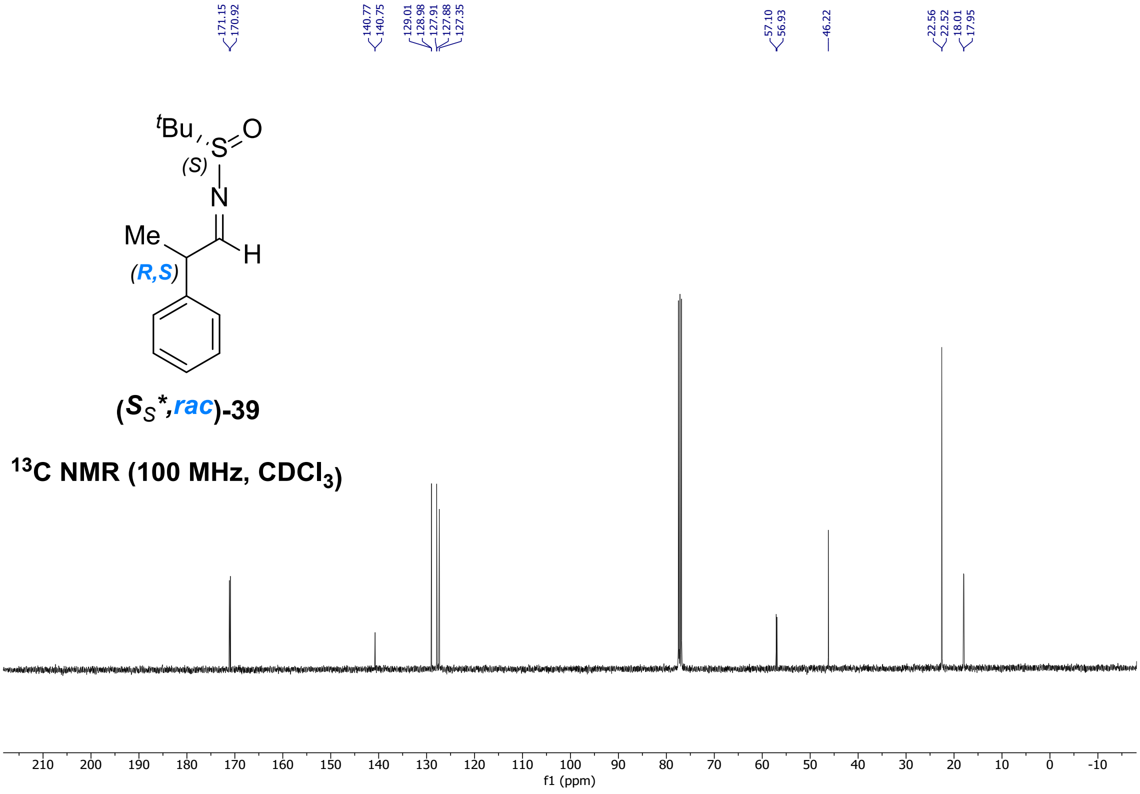

(S_S*,rac)-39
13C NMR (100 MHz, CDCl3)
f1 (ppm)

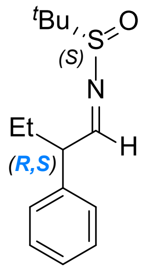

(S_S*,*rac*)-41

^{1}H NMR (400 MHz, $CDCl_3$)

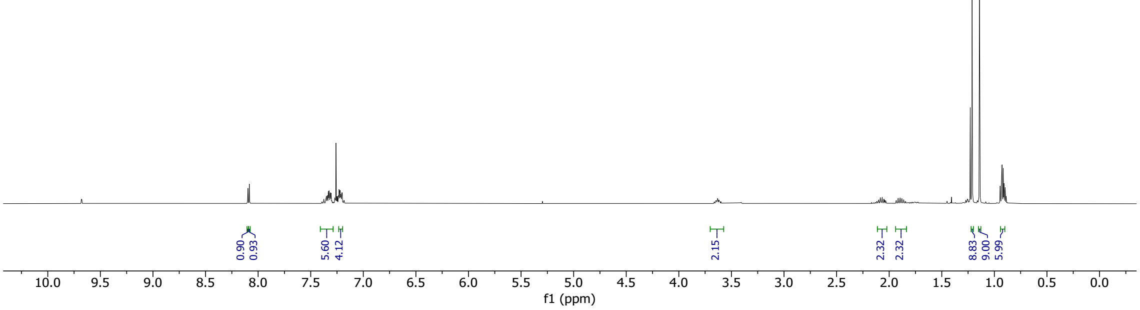

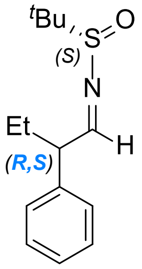

(S_S*,*rac*)-41

^{13}C NMR (100 MHz, $CDCl_3$)

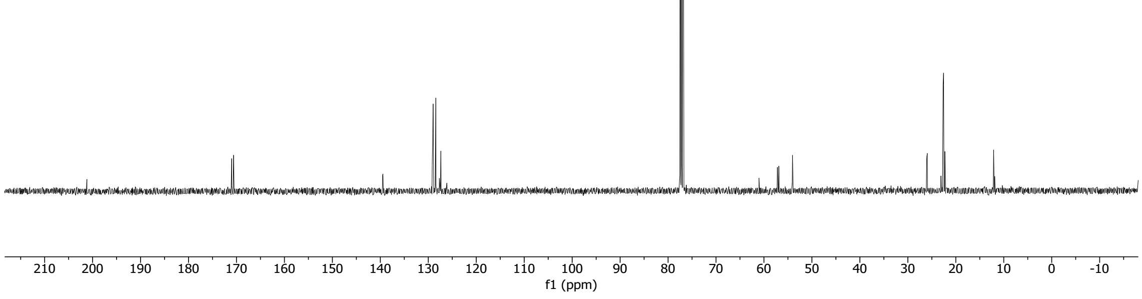

^1H, ^{13}C, ^{11}B, ^{19}F NMR spectra for chiral aziridines

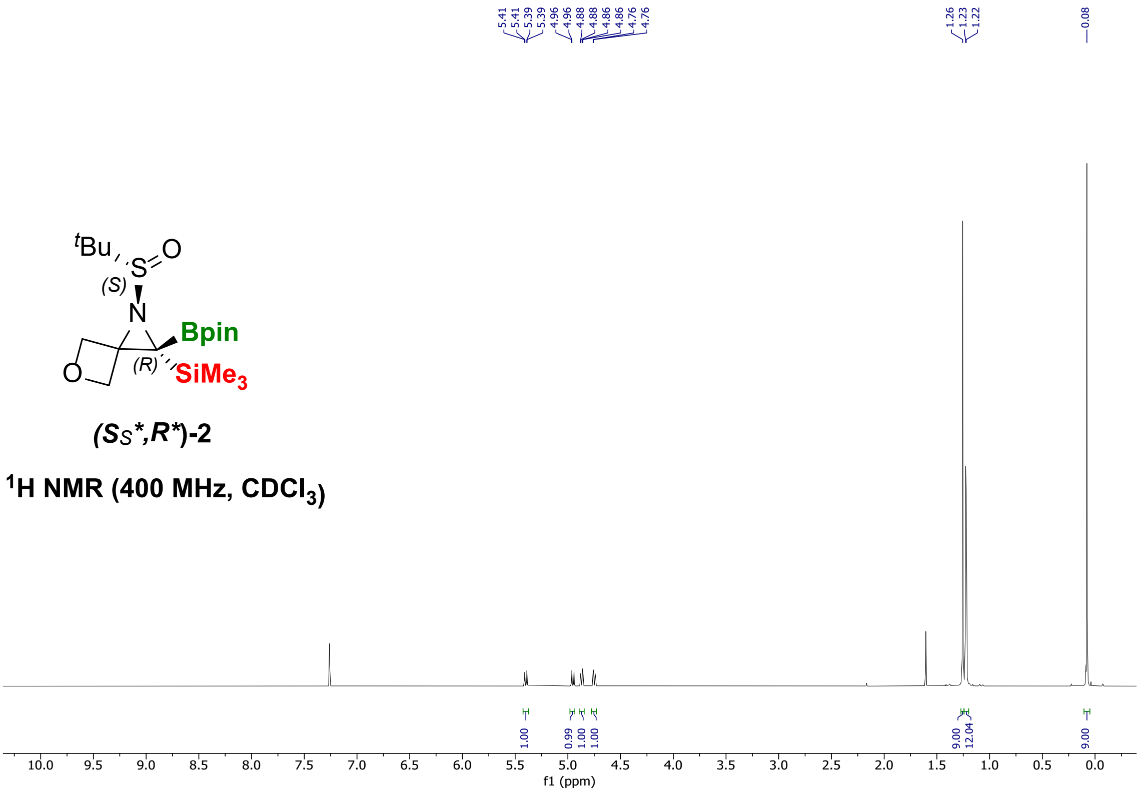

(S_S*,R*)-2

^1H NMR (400 MHz, $CDCl_3$)

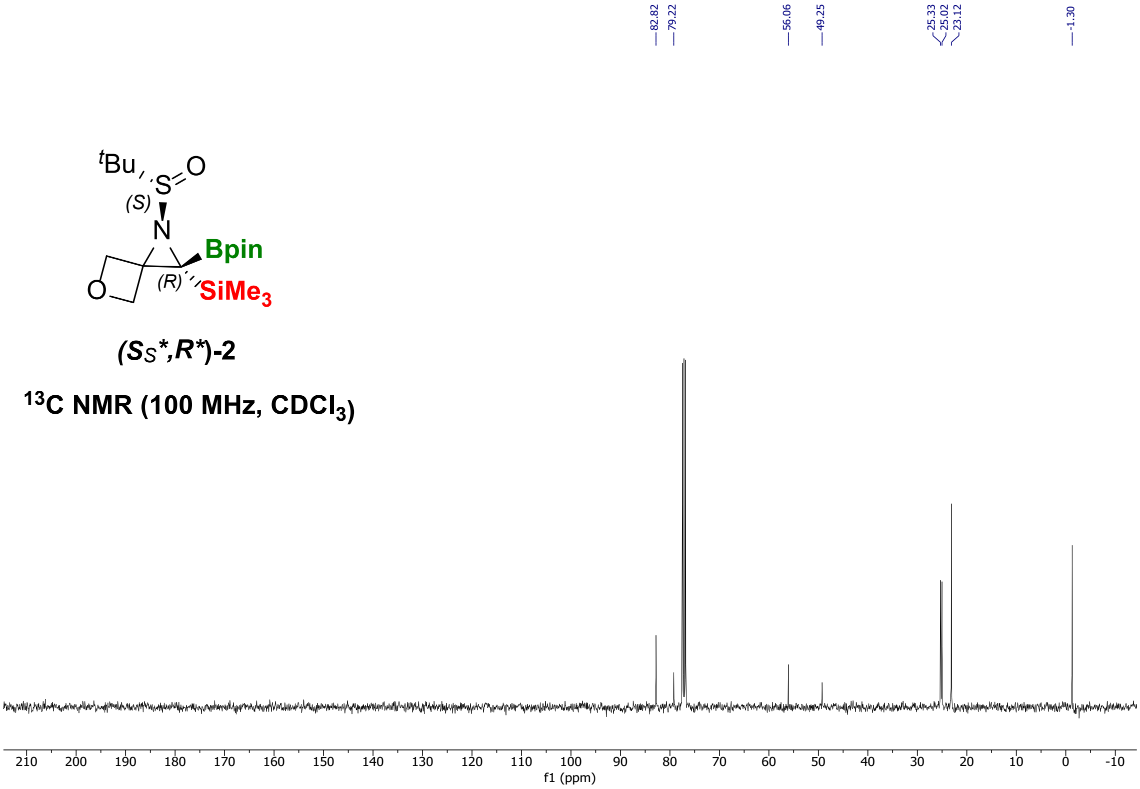

(S_S*,R*)-2

^{13}C NMR (100 MHz, $CDCl_3$)

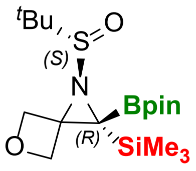

(S_S*,R*)-2

^{11}B NMR (129 MHz, CDCl$_3$)

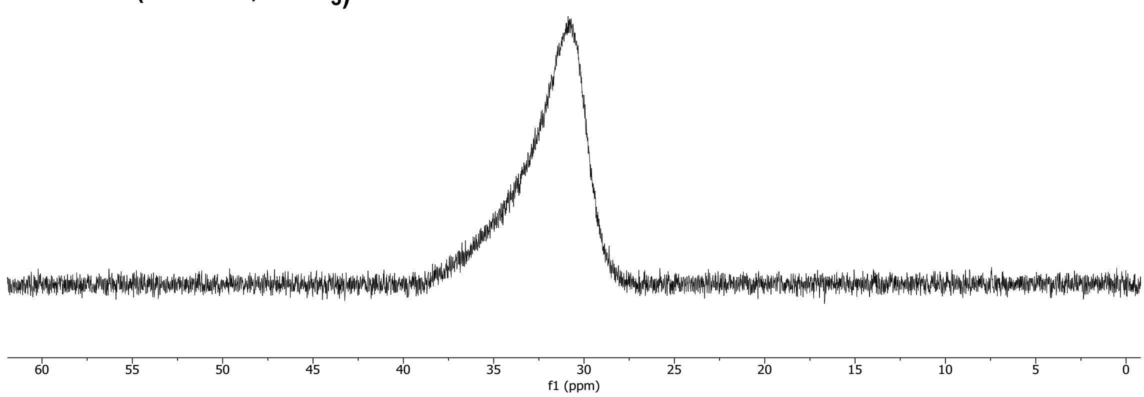

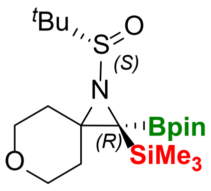

(S_S*,R*)-4

^{1}H NMR (400 MHz, CDCl$_3$)

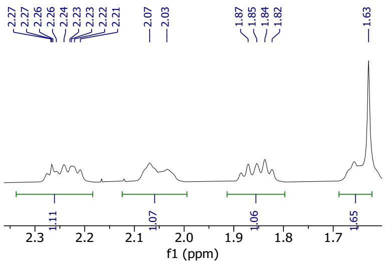

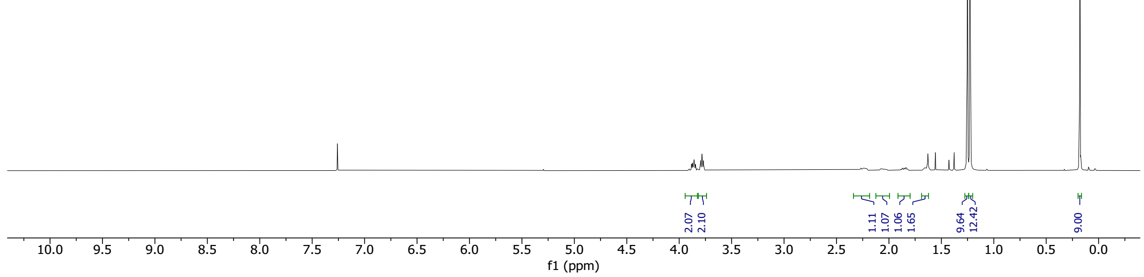

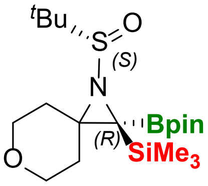

(S_S*,R*)-4

^{13}C NMR (100 MHz, $CDCl_3$)

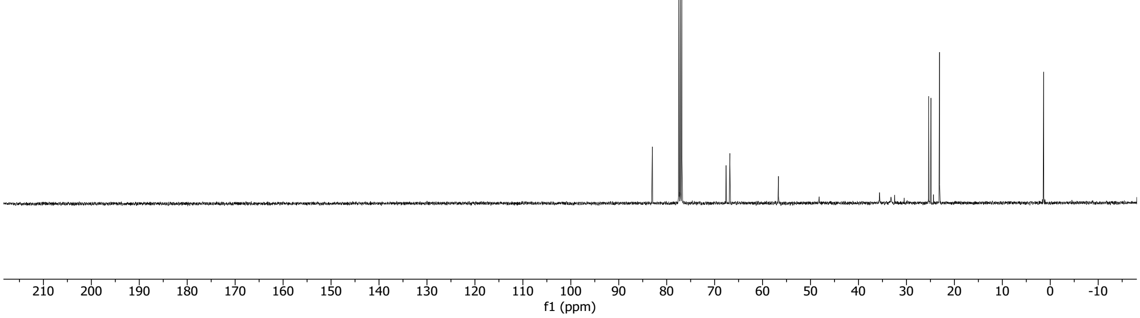

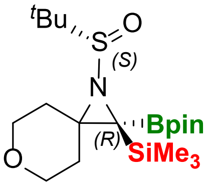

(S_S*,R*)-4

^{11}B NMR (129 MHz, $CDCl_3$)

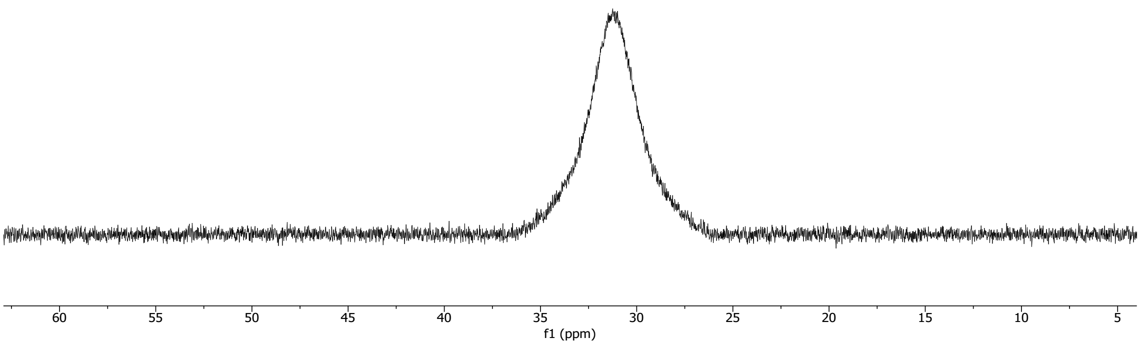

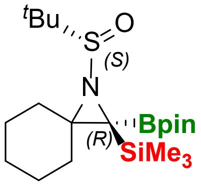

(S_S*,R*)-6

^{1}H NMR (400 MHz, $CDCl_3$)

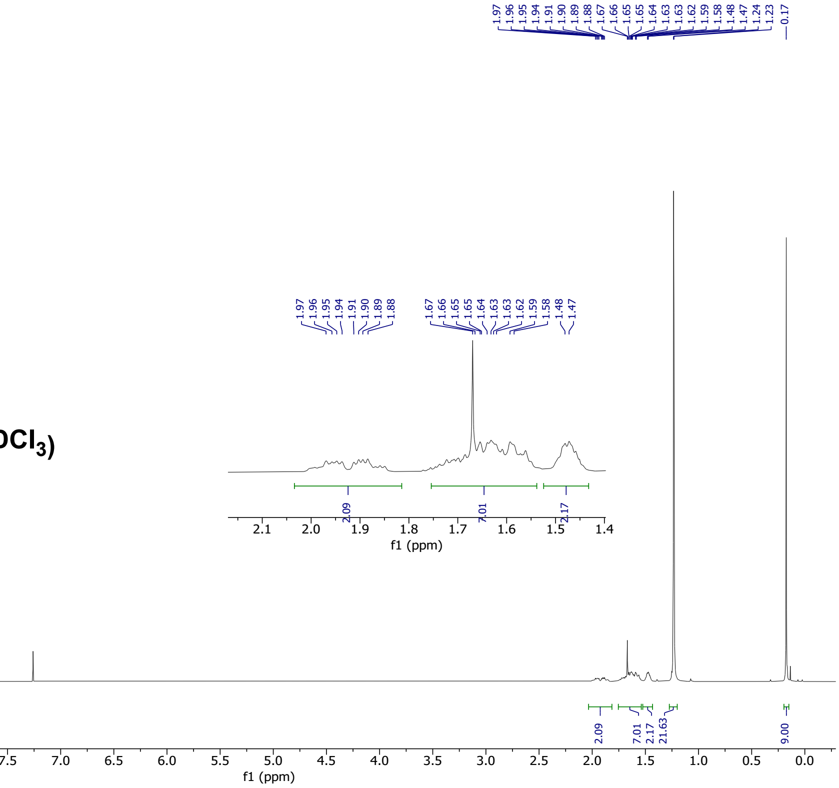

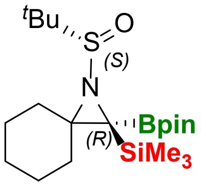

(S_S*,R*)-6

^{13}C NMR (100 MHz, $CDCl_3$)

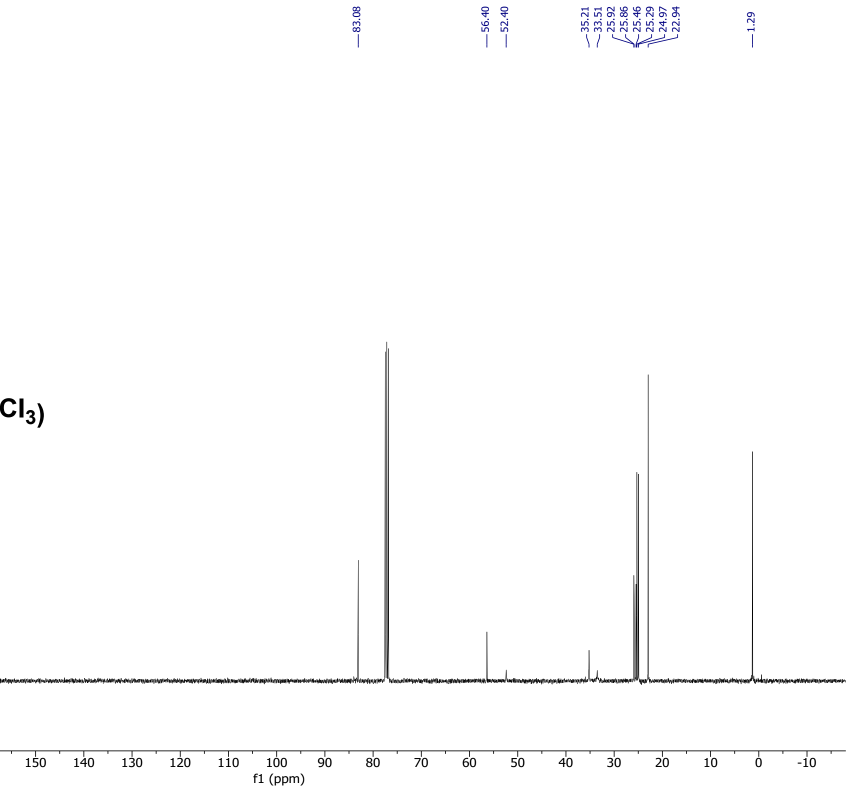

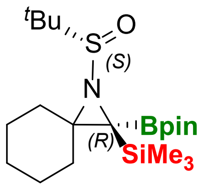

(S_S*,R*)-6

^{11}B NMR (129 MHz, $CDCl_3$)

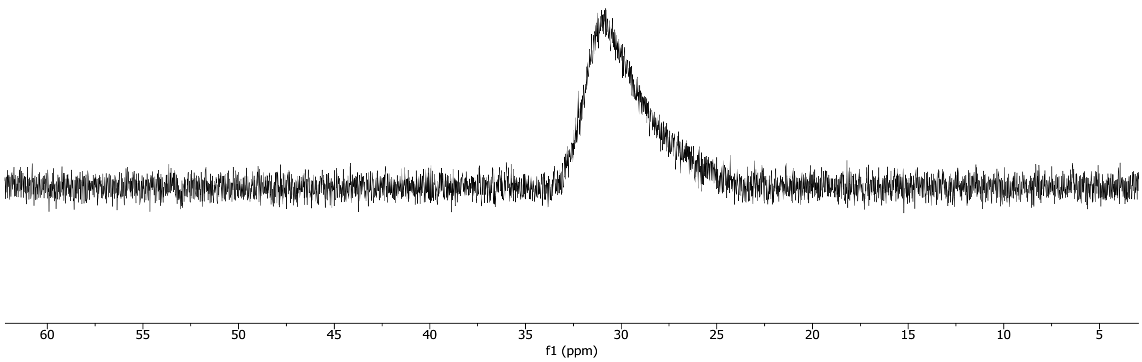

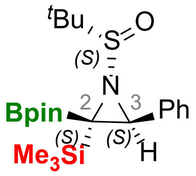

(S_S*,2S*,3S*)-8

^{1}H NMR (400 MHz, $CDCl_3$)

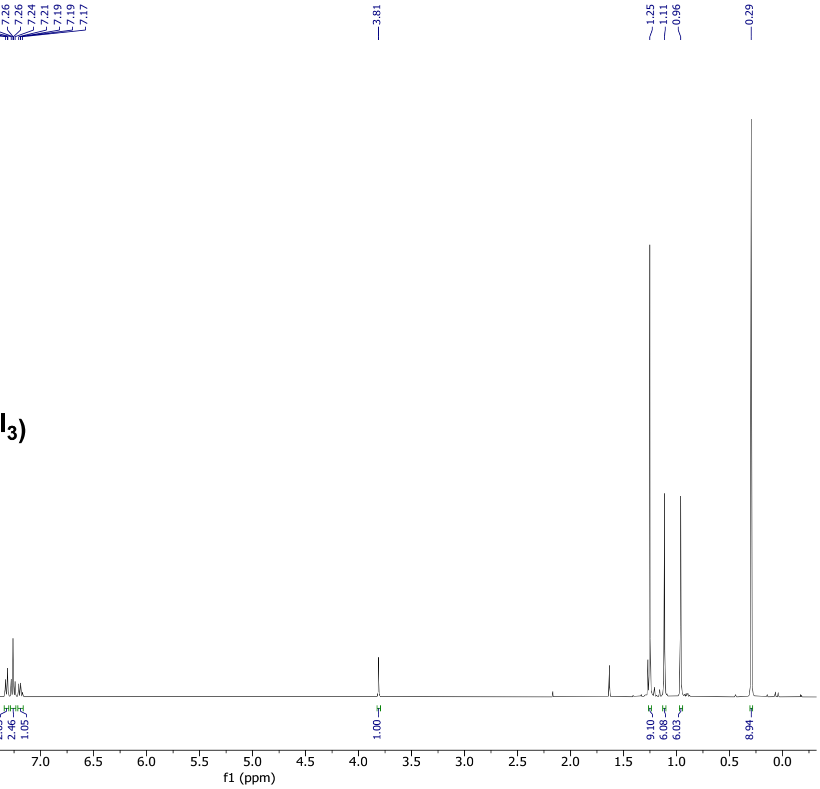

2D-NMR NOESY experiment for 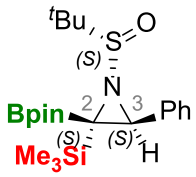

(S_S*,2*S,3*S**)-8**

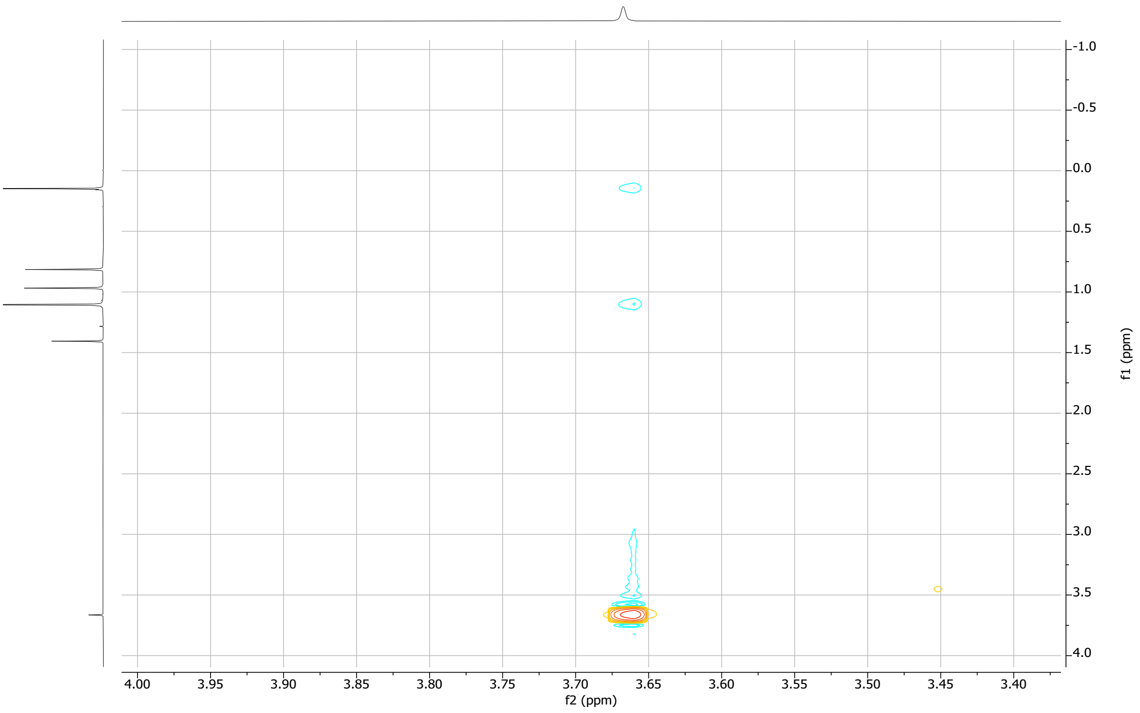

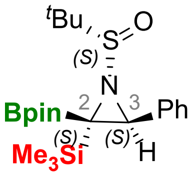

(S_S*,2*S,3*S**)-8**

^{13}C NMR (100 MHz, $CDCl_3$)

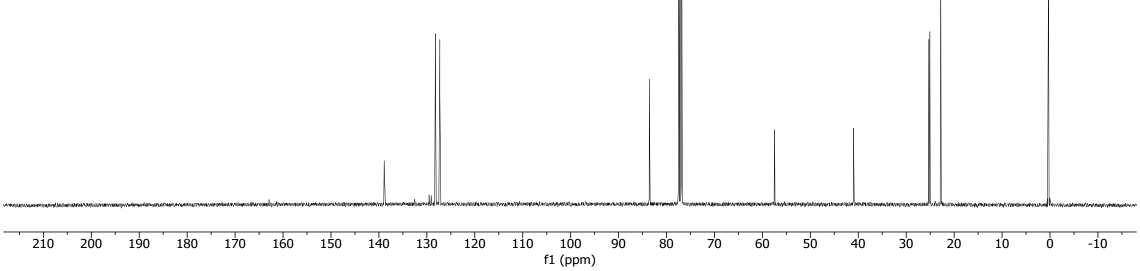

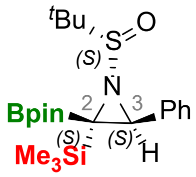

(S_S*,2**S***,3**S***)-**8**

^{11}B NMR (129 MHz, CDCl$_3$)

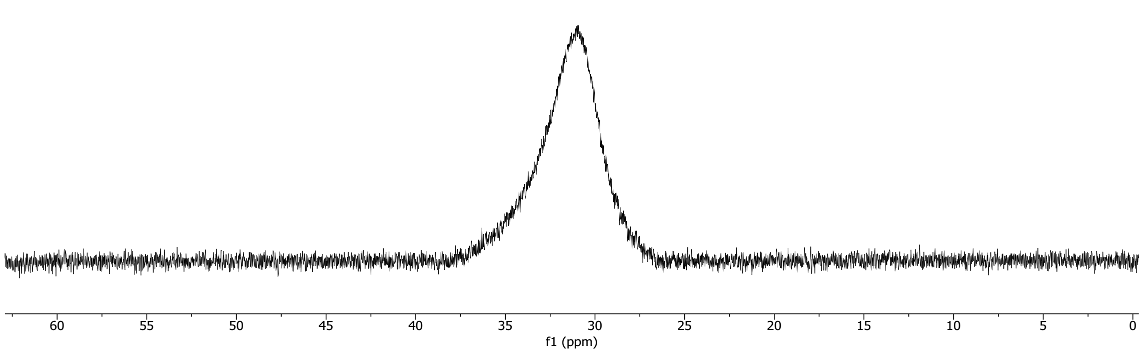

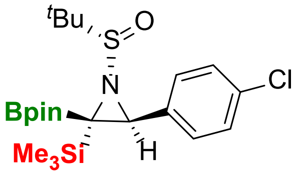

(S_S*,2**S***,3**S***)-**10**

^{1}H NMR (400 MHz, CDCl$_3$)

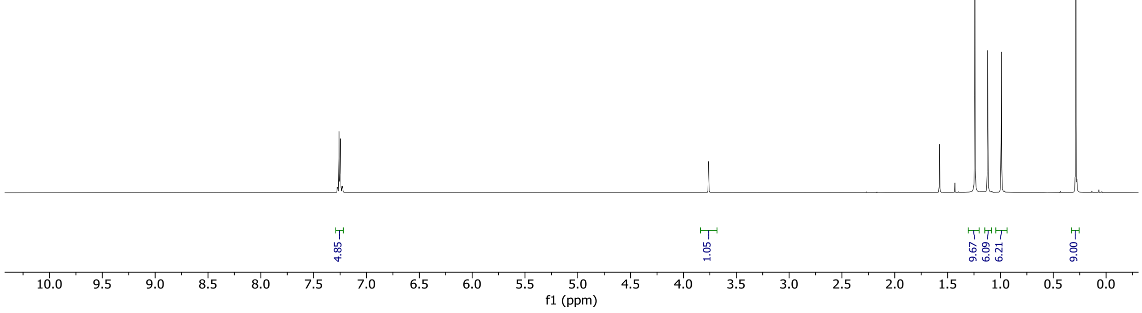

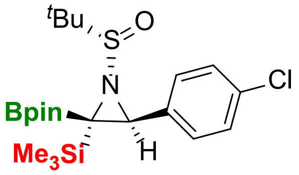

(S_S*,2*S,3*S**)-10**

^{13}C NMR (100 MHz, CDCl$_3$)

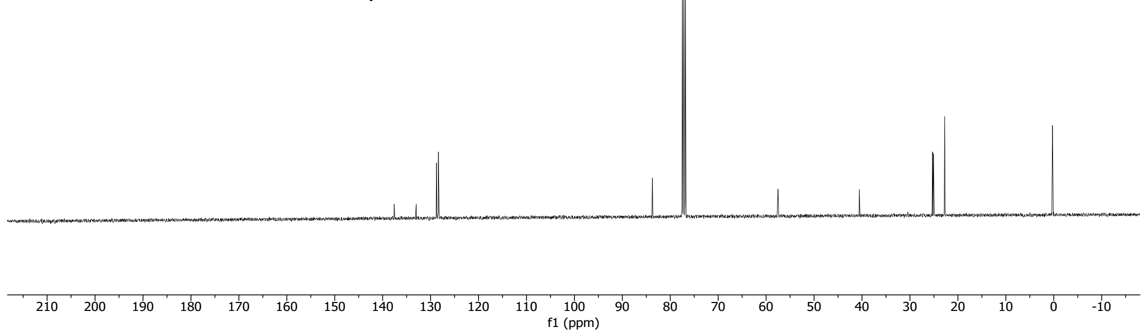

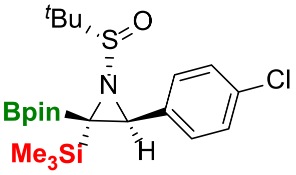

(S_S*,2*S,3*S**)-10**

^{11}B NMR (129 MHz, CDCl$_3$)

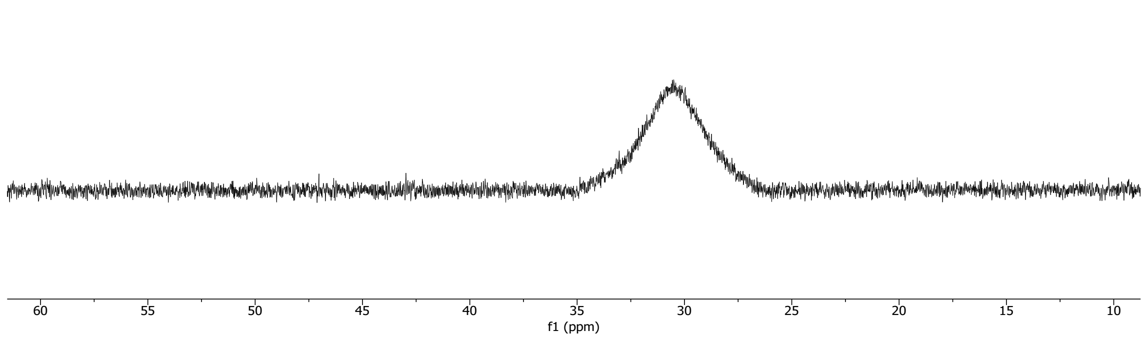

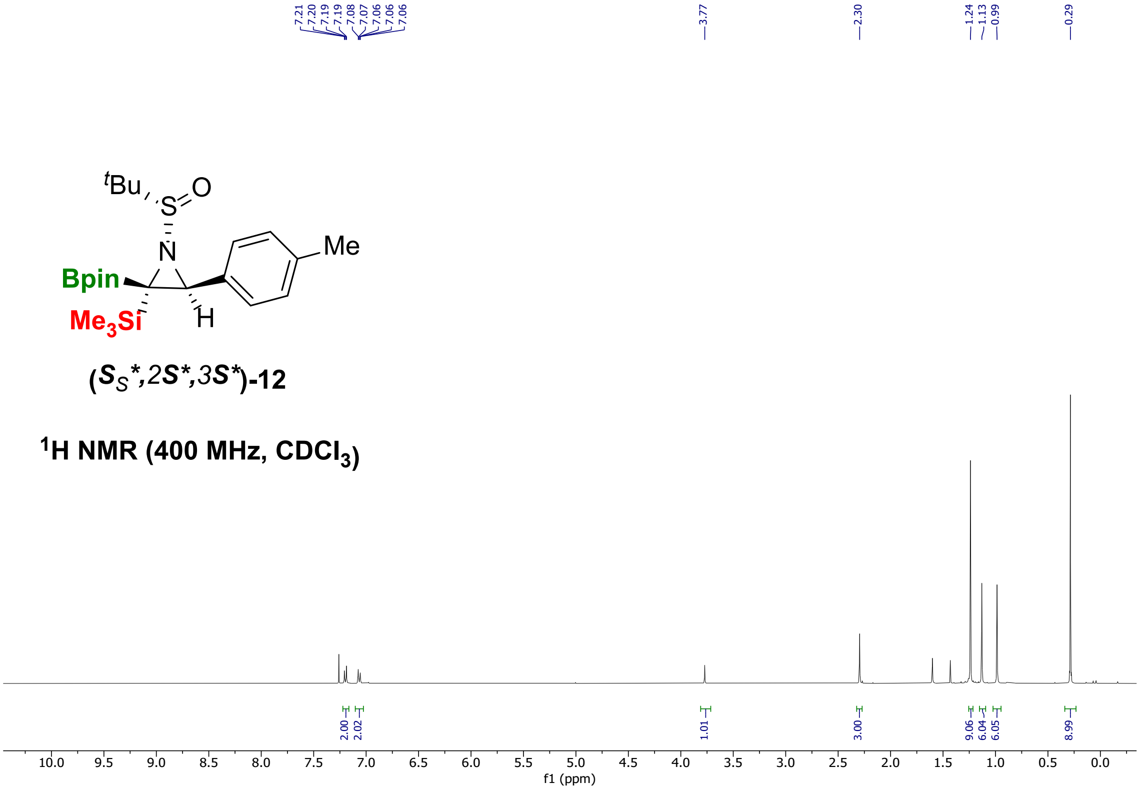

tBu
S
O
N
Me
Bpin
Me3Si
H
(SS*,2S*,3S*)-12
1H NMR (400 MHz, CDCl3)
7.21
7.20
7.19
7.19
7.08
7.07
7.06
7.06
7.06
3.77
2.30
1.24
1.13
0.99
0.29
2.00
2.02
1.01
3.00
9.06
6.04
6.05
8.99
10.0 9.5 9.0 8.5 8.0 7.5 7.0 6.5 6.0 5.5 5.0 4.5 4.0 3.5 3.0 2.5 2.0 1.5 1.0 0.5 0.0
f1 (ppm)

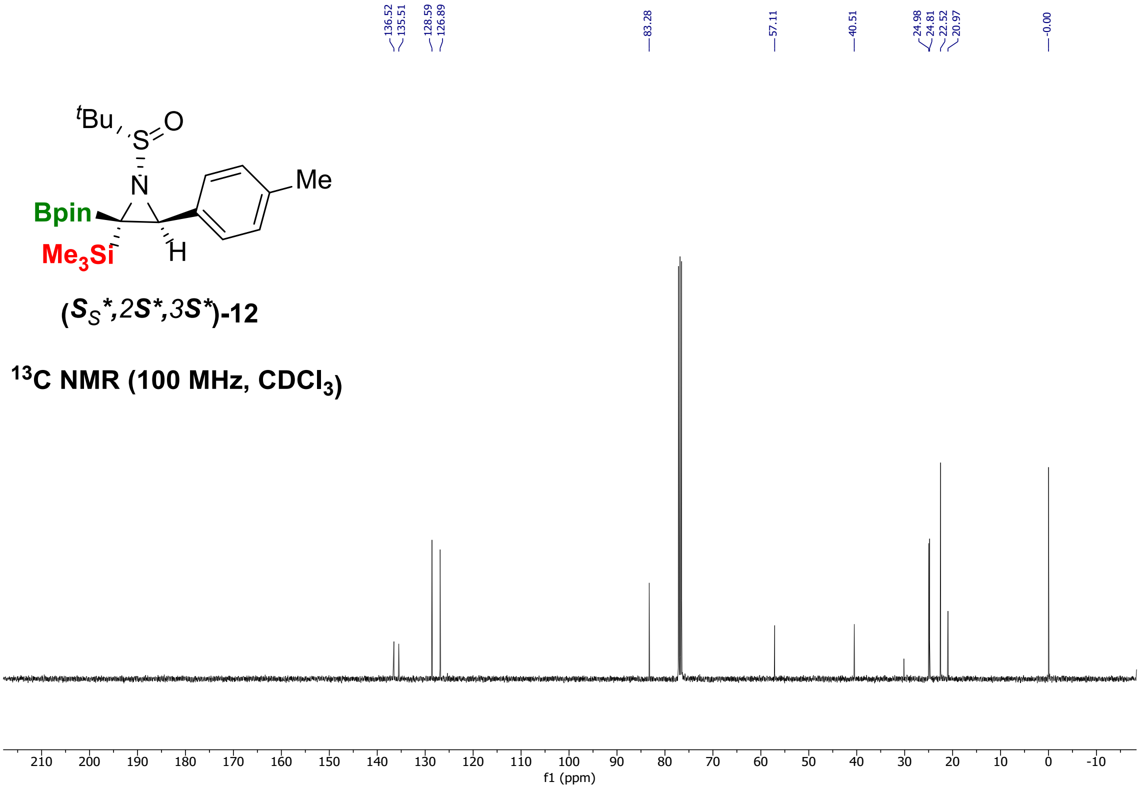

tBu
S
O
N
Me
Bpin
Me3Si
H
(SS*,2S*,3S*)-12
13C NMR (100 MHz, CDCl3)
136.52
135.51
128.59
126.89
83.28
57.11
40.51
24.98
24.81
22.52
20.97
-0.00
210 200 190 180 170 160 150 140 130 120 110 100 90 80 70 60 50 40 30 20 10 0 -10
f1 (ppm)

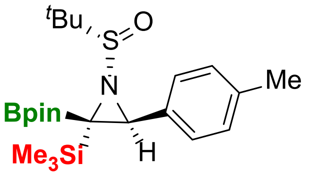

(S_S*,2S*,3S*)-12

^{11}B NMR (129 MHz, $CDCl_3$)

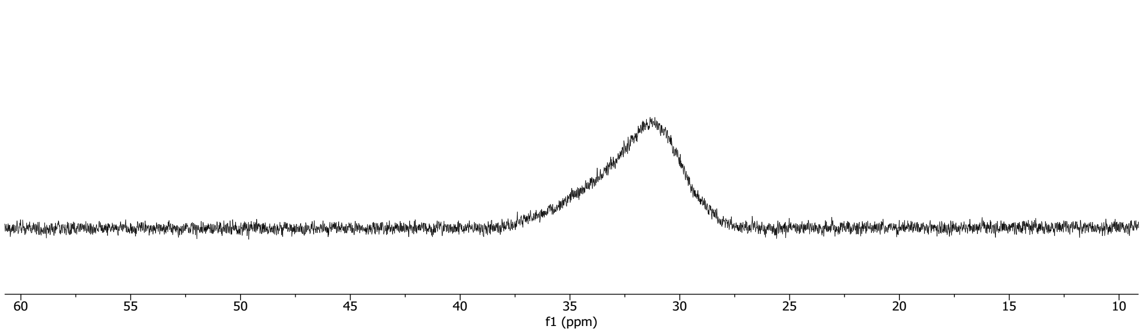

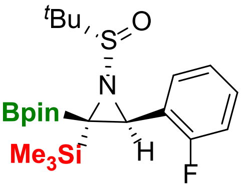

(S_S*,2S*,3S*)-14

^{1}H NMR (400 MHz, $CDCl_3$)

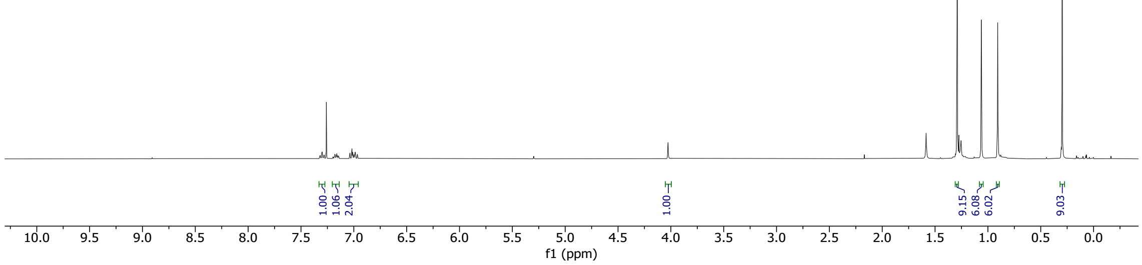

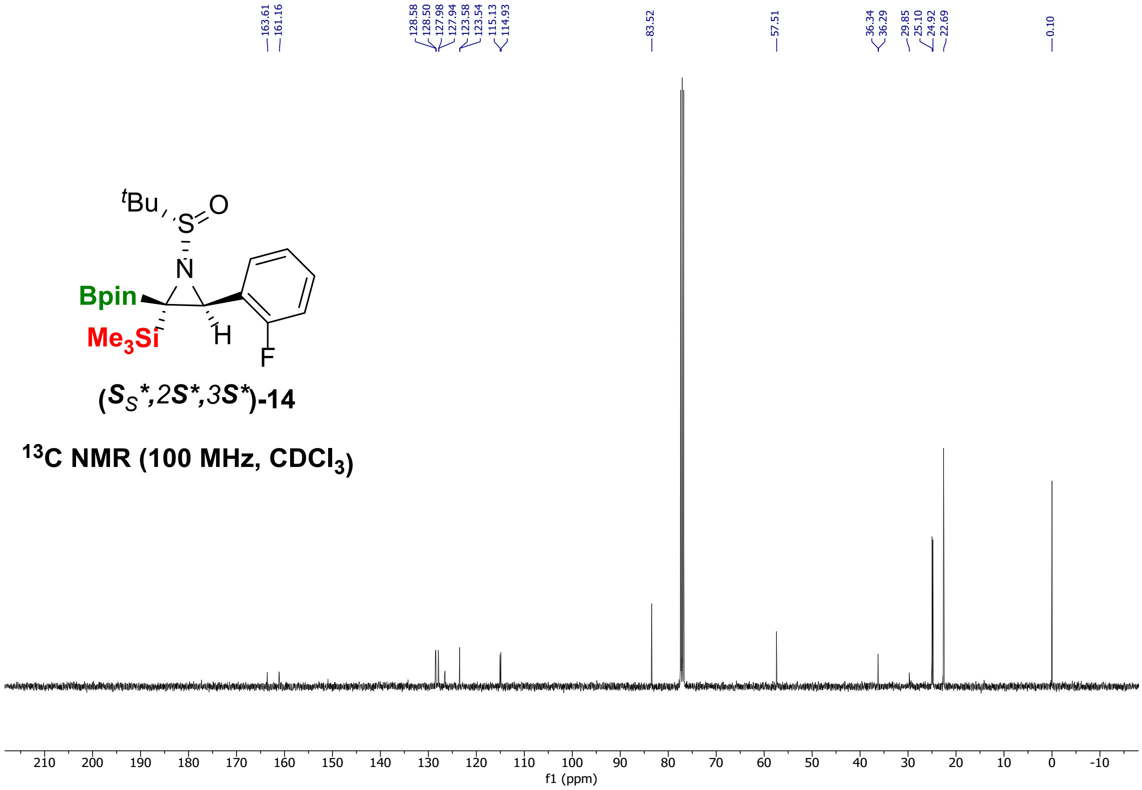

163.61
161.16
128.58
128.50
127.98
127.94
123.58
123.54
115.13
114.93
83.52
57.51
36.34
36.29
29.85
25.10
24.92
22.69
0.10
tBu
S
O
N
Bpin
Me3Si
H
F
(SS*,2S*,3S*)-14
13C NMR (100 MHz, CDCl3)
210
200
190
180
170
160
150
140
130
120
110
100
90
80
70
60
50
40
30
20
10
0
-10
f1 (ppm)

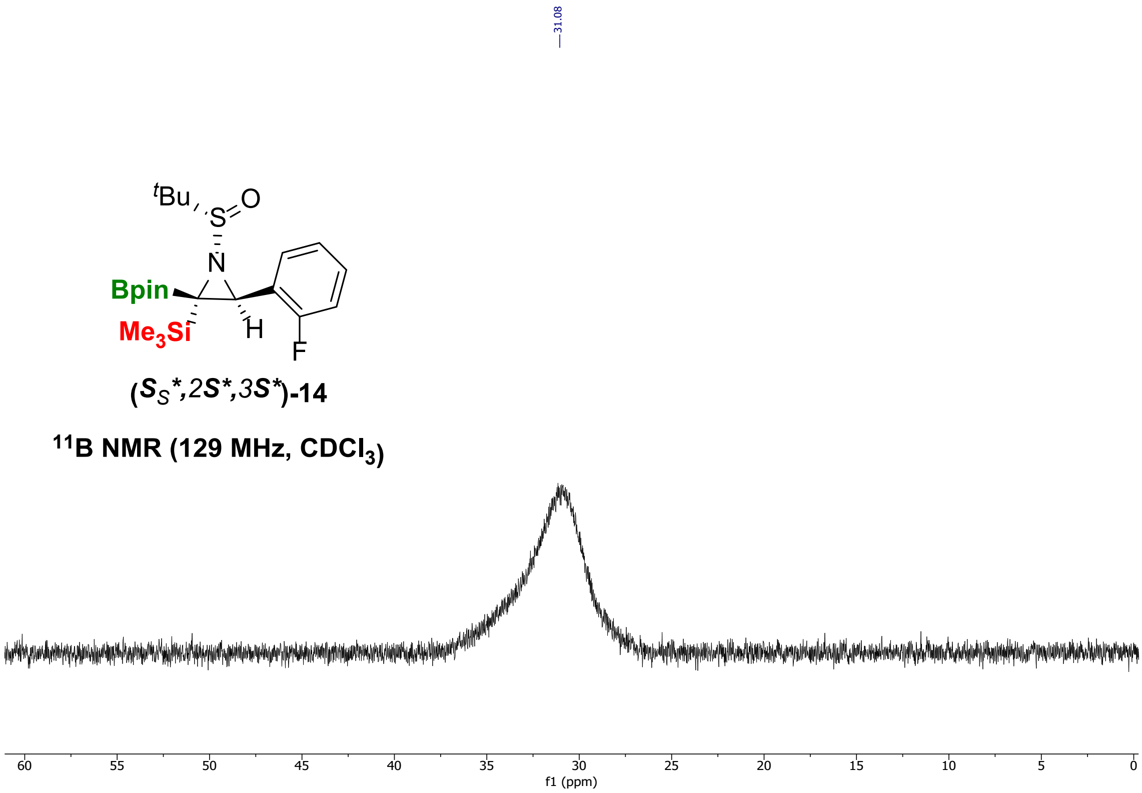

31.08
tBu
S
O
N
Bpin
Me3Si
H
F
(SS*,2S*,3S*)-14
11B NMR (129 MHz, CDCl3)
60
55
50
45
40
35
30
25
20
15
10
5
0
f1 (ppm)

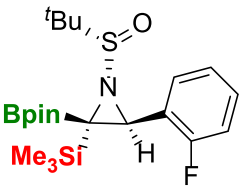

(S_S*,2S*,3S*)-14

^{19}F NMR (377 MHz, $CDCl_3$)

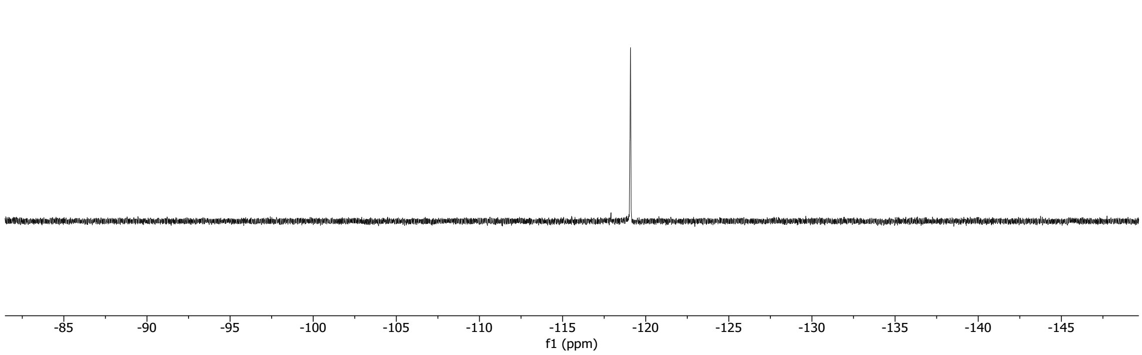

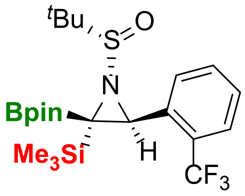

(S_S*,2S*,3S*)-16

^{1}H NMR (400 MHz, $CDCl_3$)

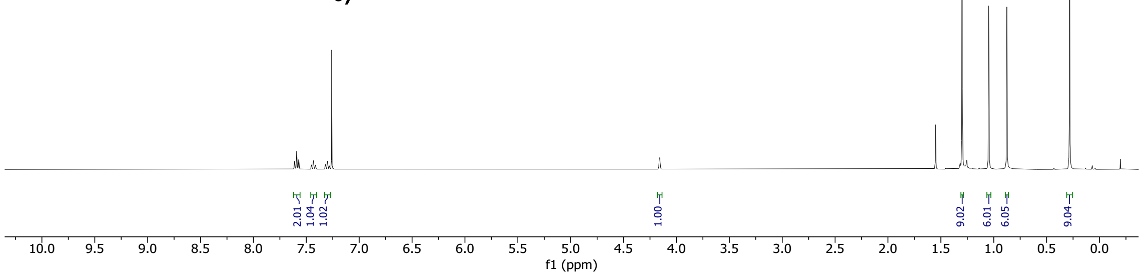

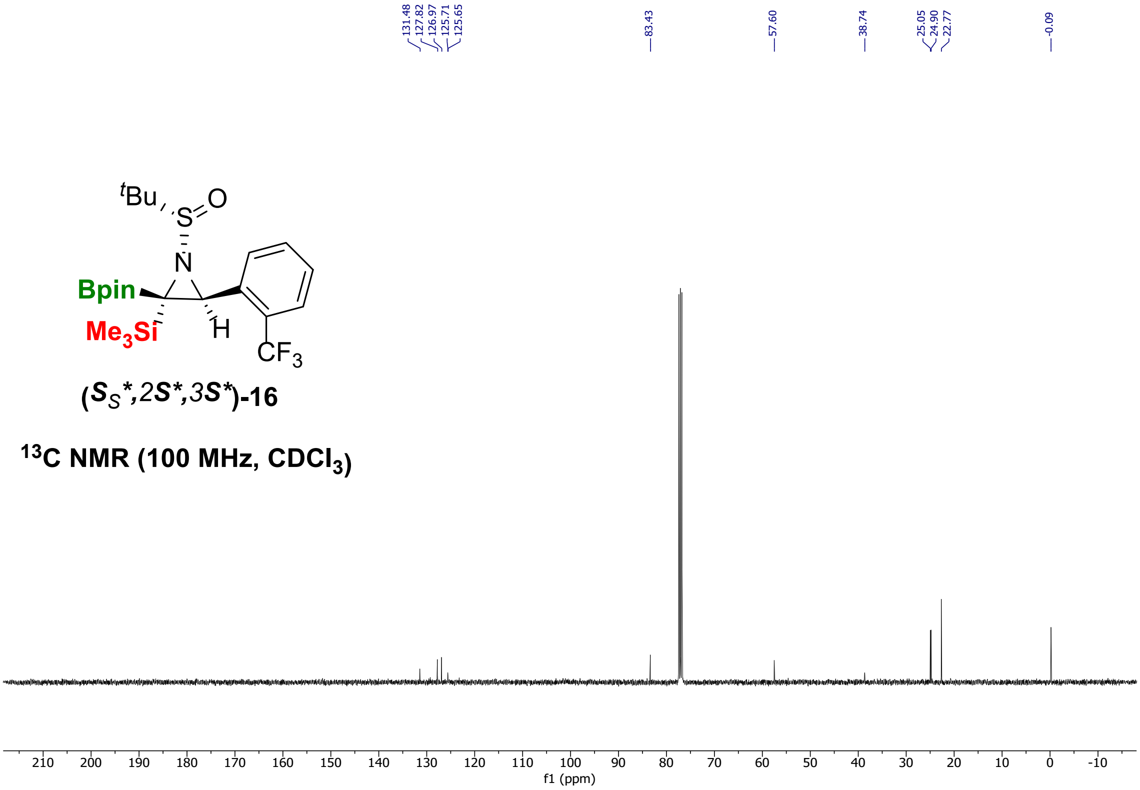

(S_S*,2*S,3*S**)-16**

^{13}C NMR (100 MHz, $CDCl_3$)

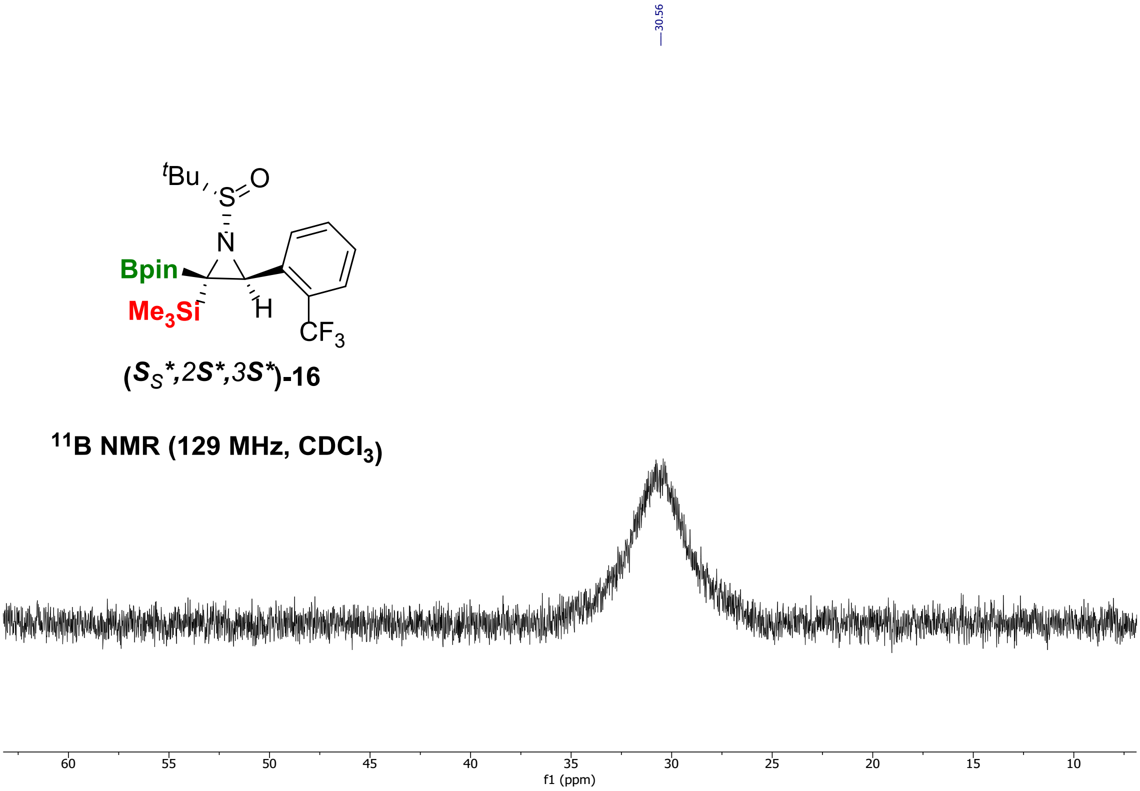

(S_S*,2*S,3*S**)-16**

^{11}B NMR (129 MHz, $CDCl_3$)

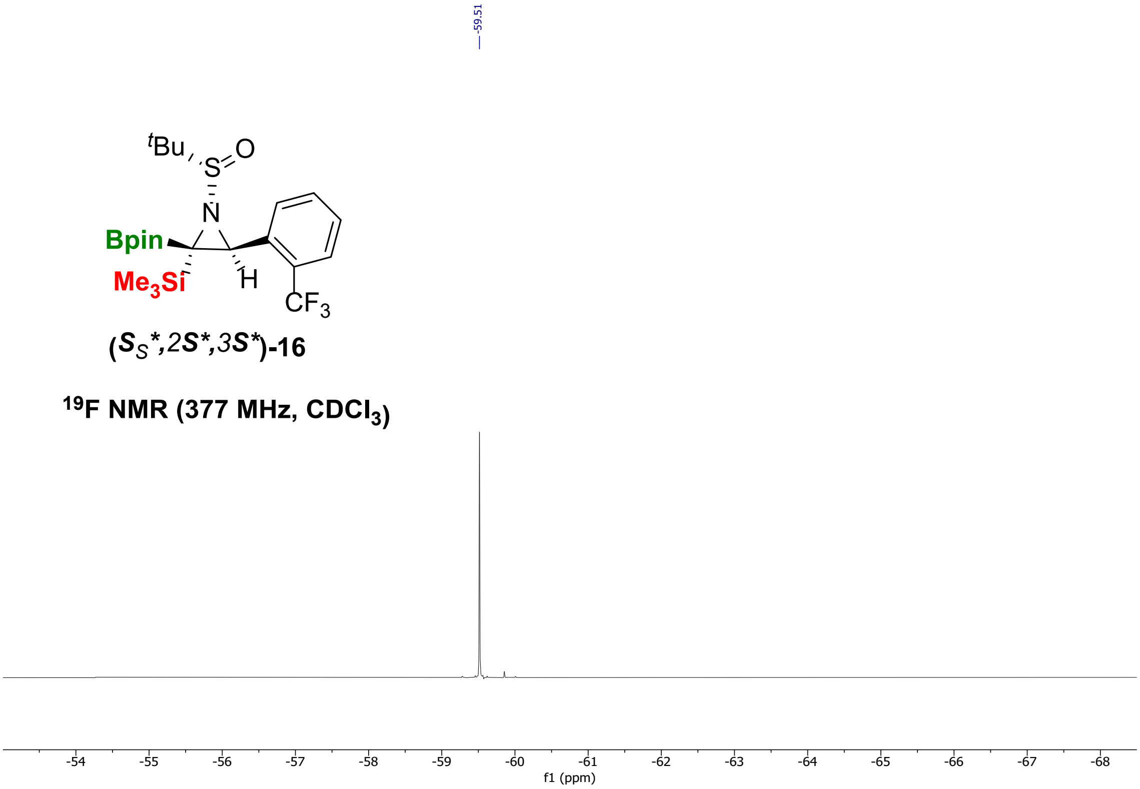

-59.51
tBu
S
O
N
Bpin
Me3Si
H
CF3
(SS*,2S*,3S*)-16
19F NMR (377 MHz, CDCl3)
f1 (ppm)

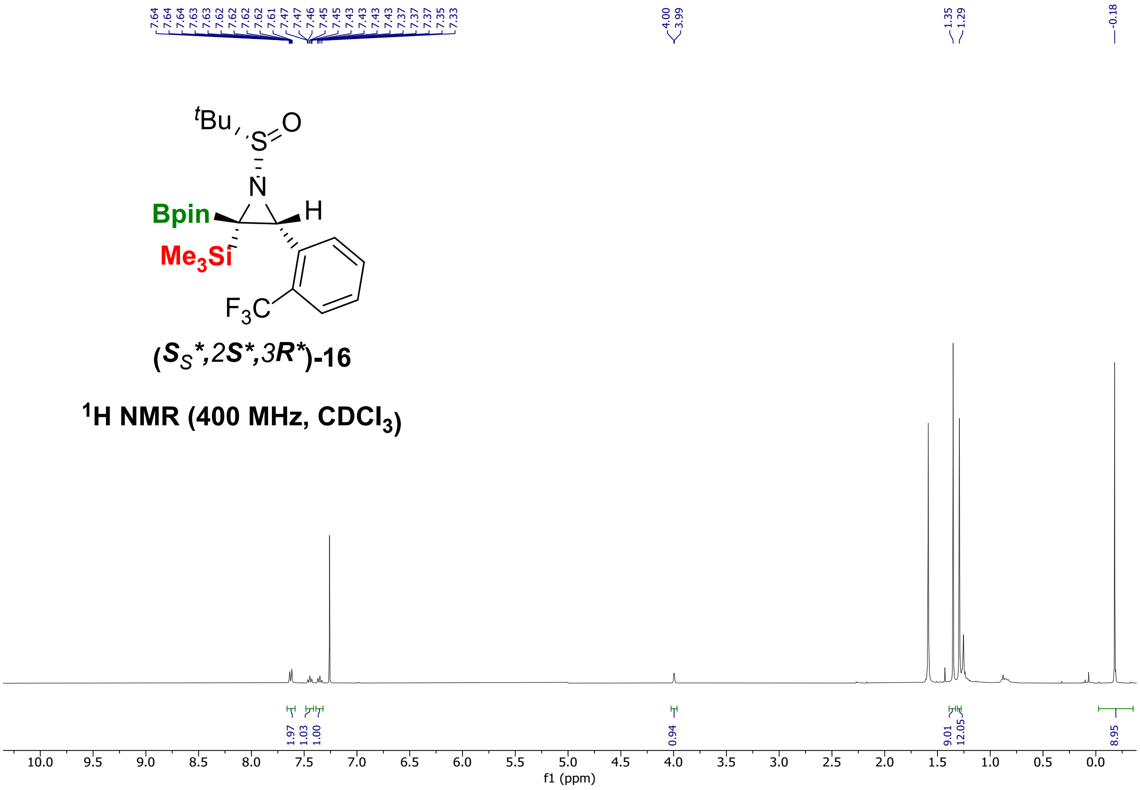

4.00
3.99
1.35
1.29
-0.18
tBu
S
O
N
Bpin
H
Me3Si
F3C
(SS*,2S*,3R*)-16
1H NMR (400 MHz, CDCl3)
1.97
1.03
1.00
0.94
9.01
12.05
8.95
f1 (ppm)

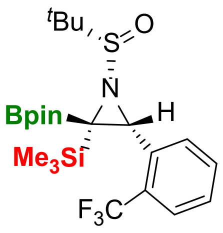

(S_S*,2*S**,3*R**)-16

^{13}C NMR (100 MHz, CDCl$_3$)

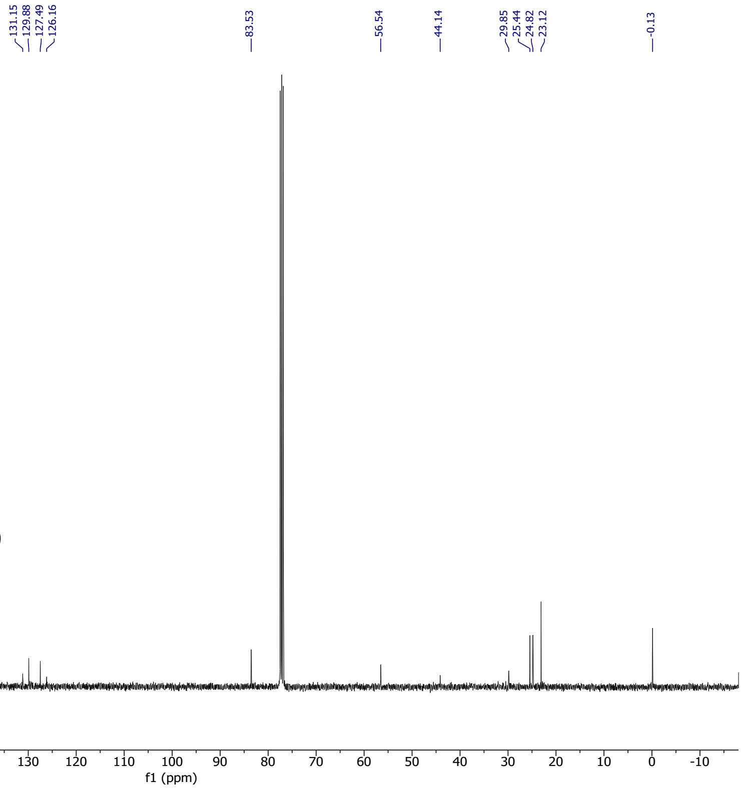

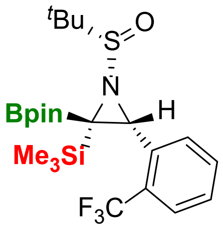

(S_S*,2*S**,3*R**)-16

^{11}B NMR (129 MHz, CDCl$_3$)

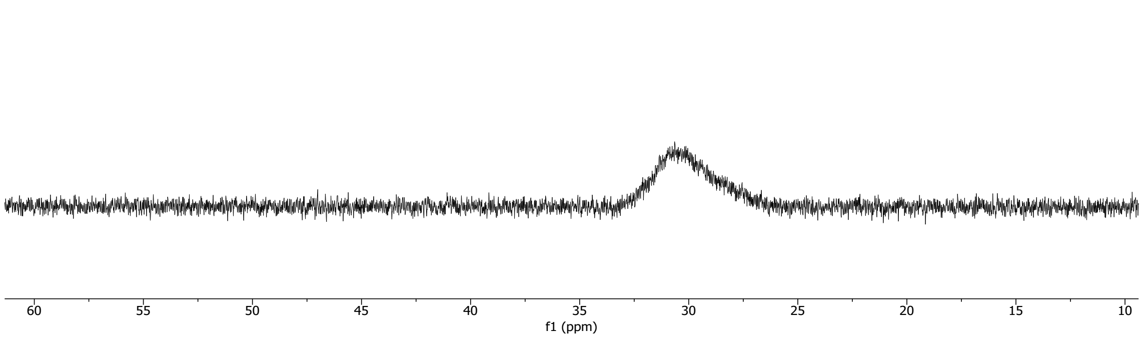

2D NMR NOESY experiment for

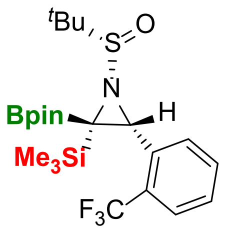

(S_S*,2S*,3R*)-16

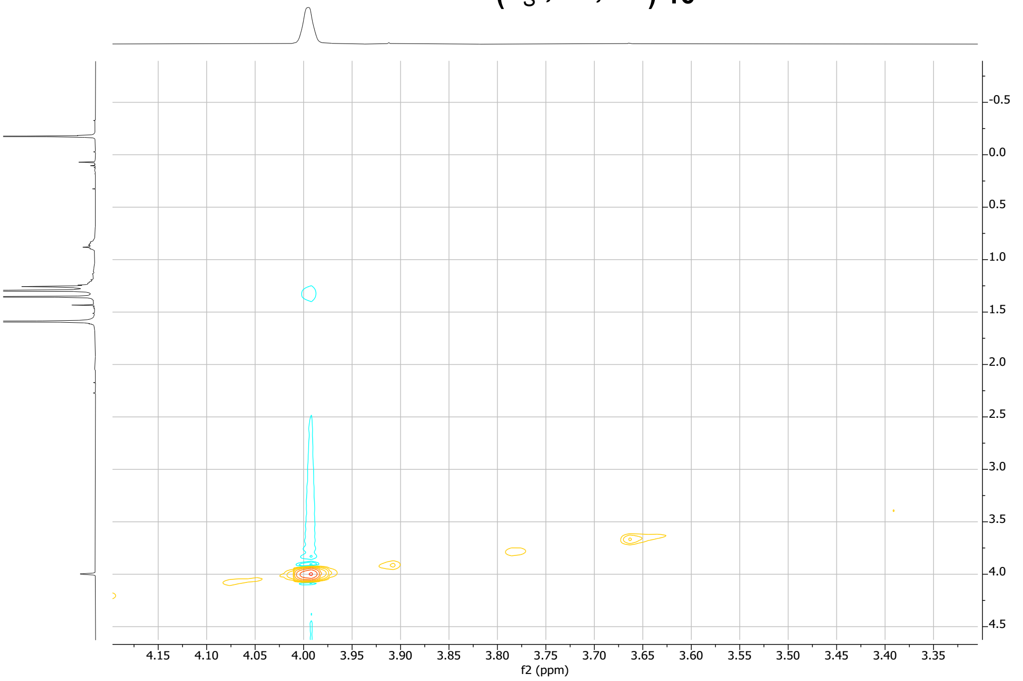

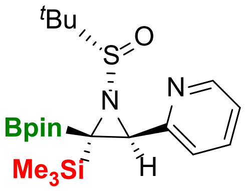

(S_S*,2S*,3S*)-18

^{1}H NMR (400 MHz, $CDCl_3$)

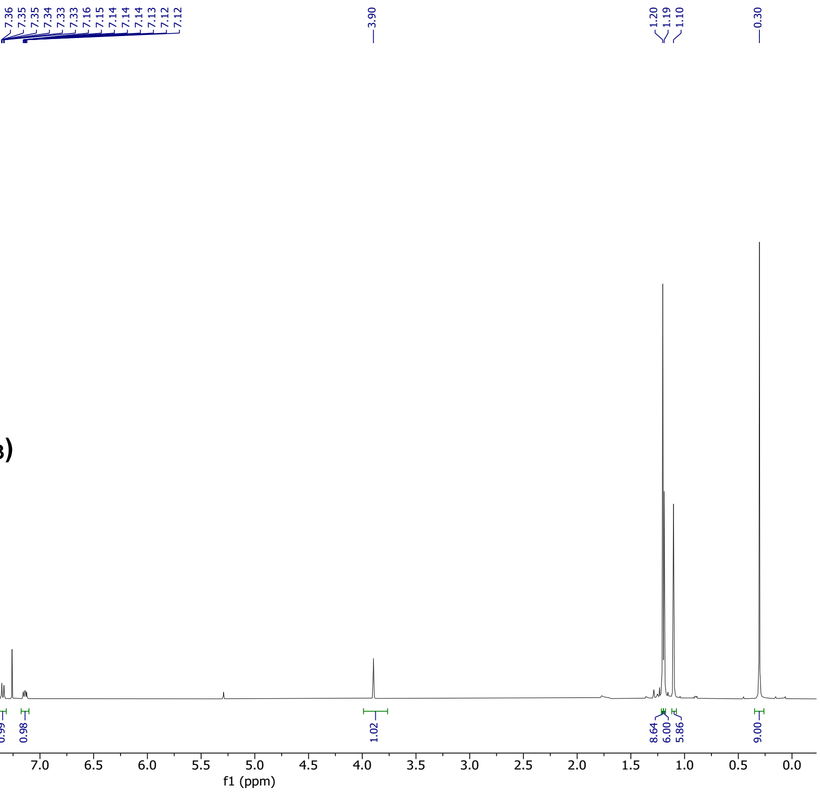

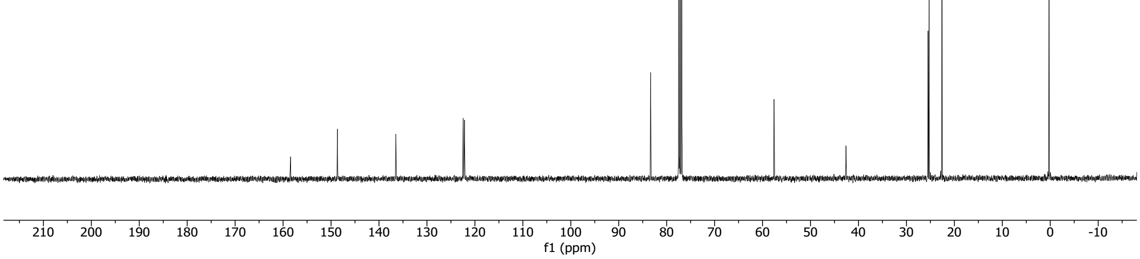

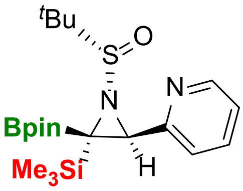

(S_S*,2*S**,3*S**)-18

^{13}C NMR (100 MHz, CDCl$_3$)

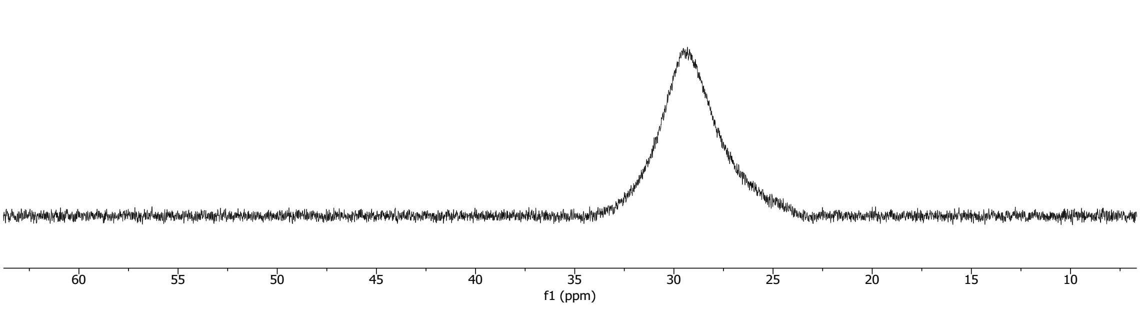

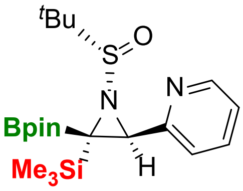

(S_S*,2*S**,3*S**)-18

^{11}B NMR (129 MHz, CDCl$_3$)

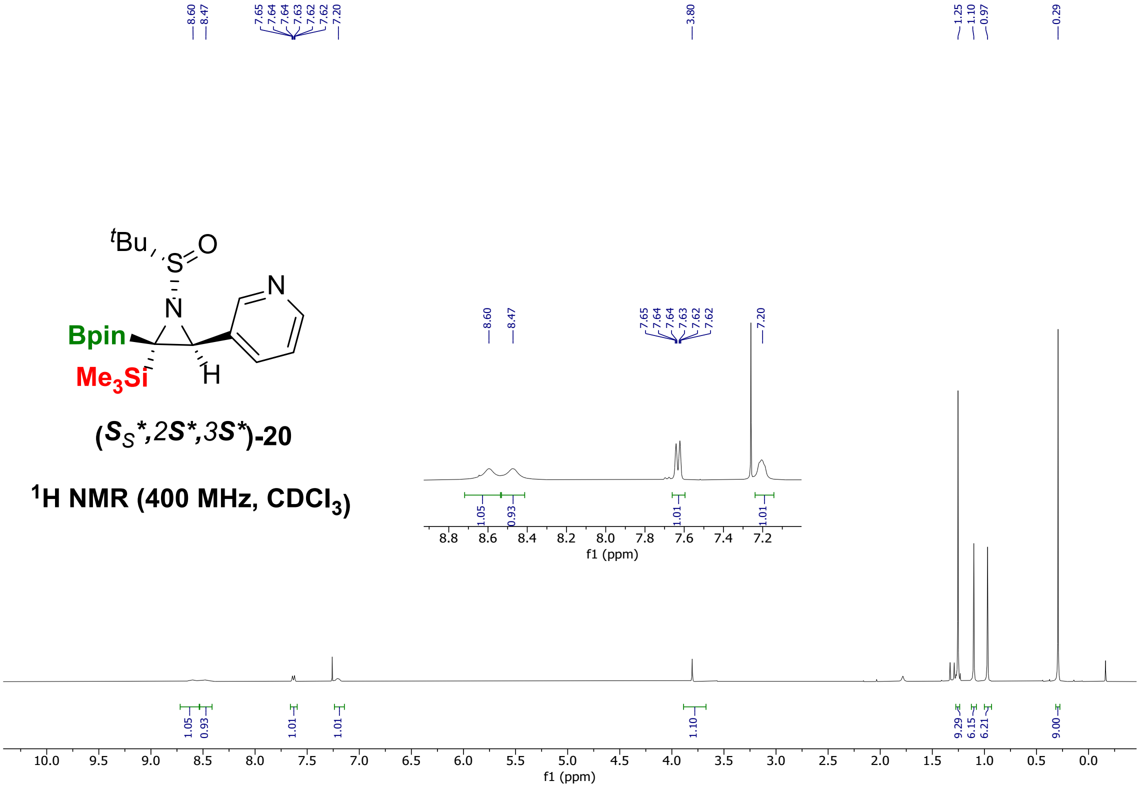

(S_S*,2*S**,3*S**)-**20**

^{1}H NMR (400 MHz, $CDCl_3$)

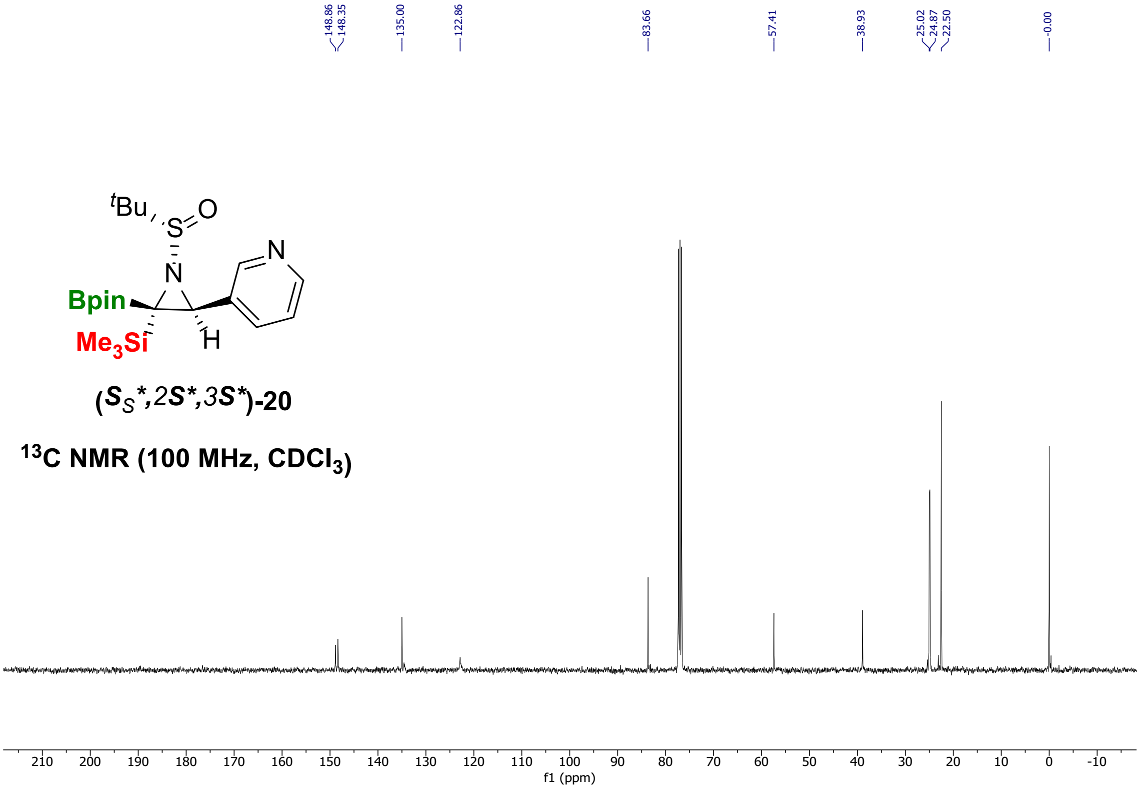

(S_S*,2*S**,3*S**)-**20**

^{13}C NMR (100 MHz, $CDCl_3$)

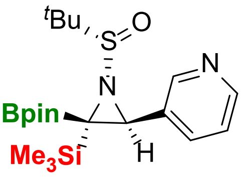

(S_S*,2S*,3S*)-20

^{11}B NMR (129 MHz, CDCl$_3$)

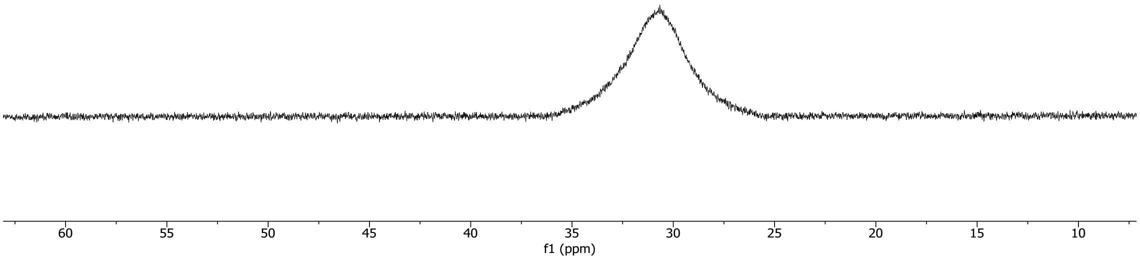

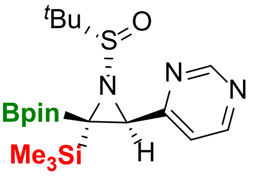

(S_S*,2S*,3S*)-22

^{1}H NMR (400 MHz, CDCl$_3$)

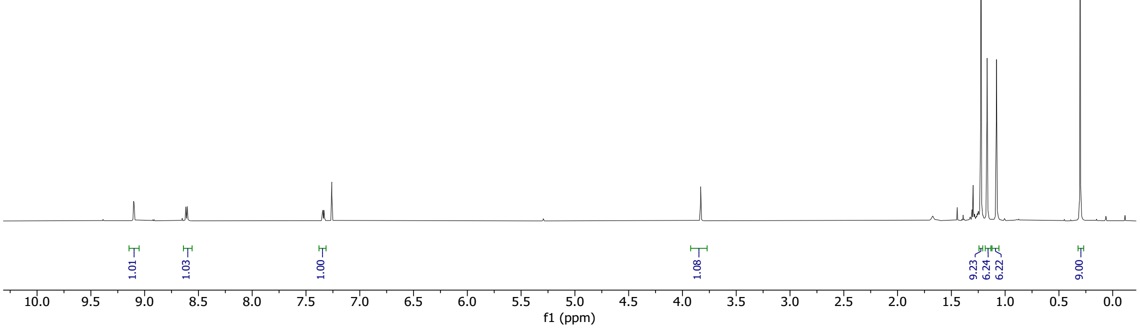

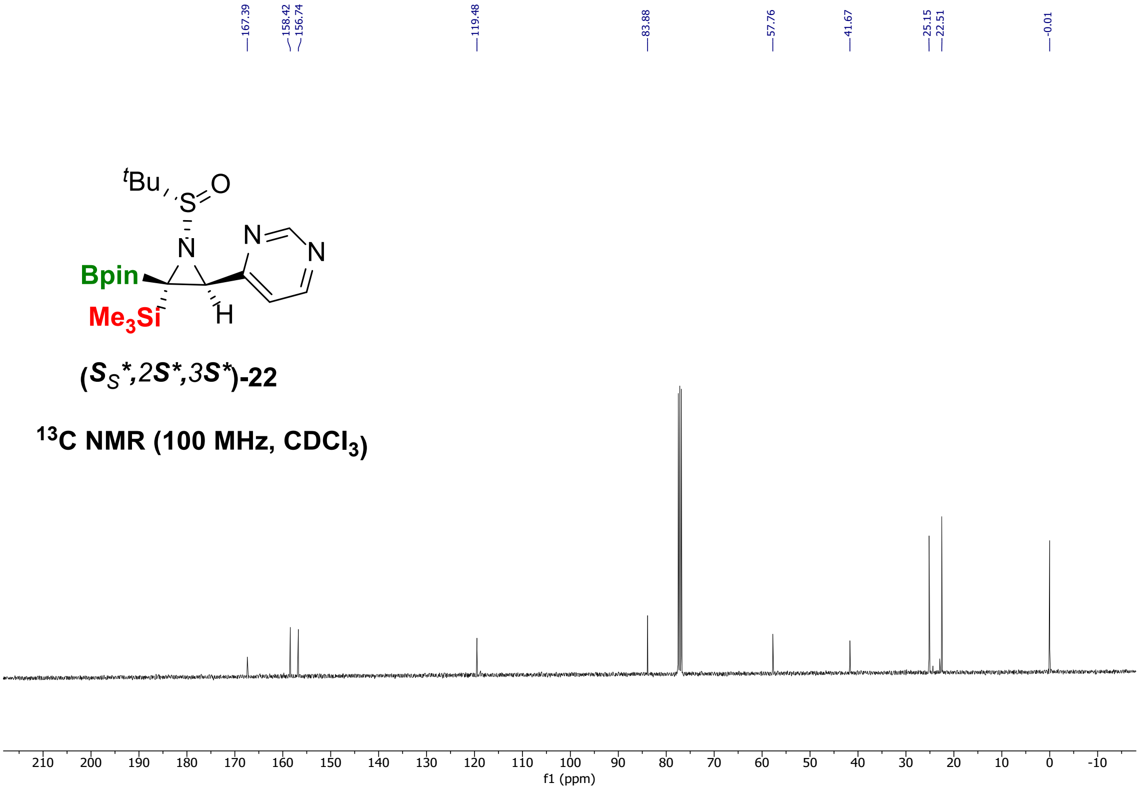

167.39
158.42
156.74
119.48
83.88
57.76
41.67
25.15
22.51
-0.01
tBu
S
O
N
N
N
Bpin
Me3Si
H
(S_S*,2S*,3S*)-22
^{13}C NMR (100 MHz, $CDCl_3$)
210 200 190 180 170 160 150 140 130 120 110 100 90 80 70 60 50 40 30 20 10 0 -10
f1 (ppm)

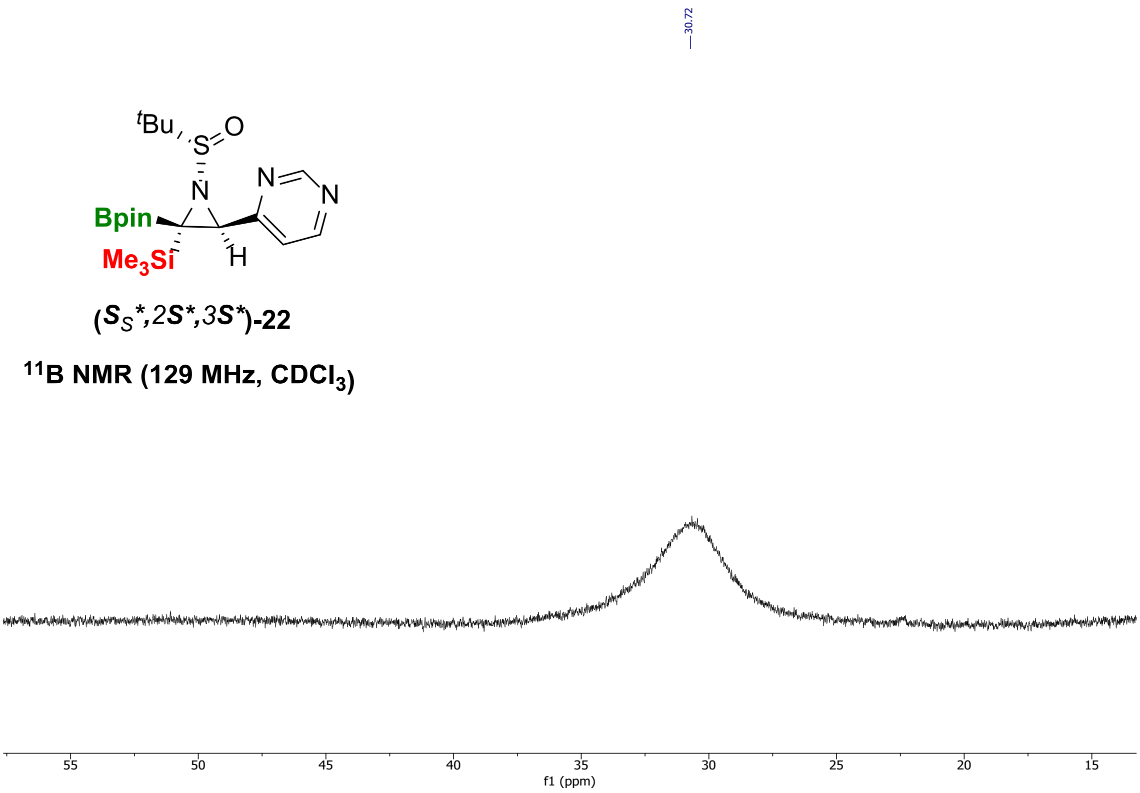

30.72
tBu
S
O
N
N
N
Bpin
Me3Si
H
(S_S*,2S*,3S*)-22
^{11}B NMR (129 MHz, $CDCl_3$)
55 50 45 40 35 30 25 20 15
f1 (ppm)

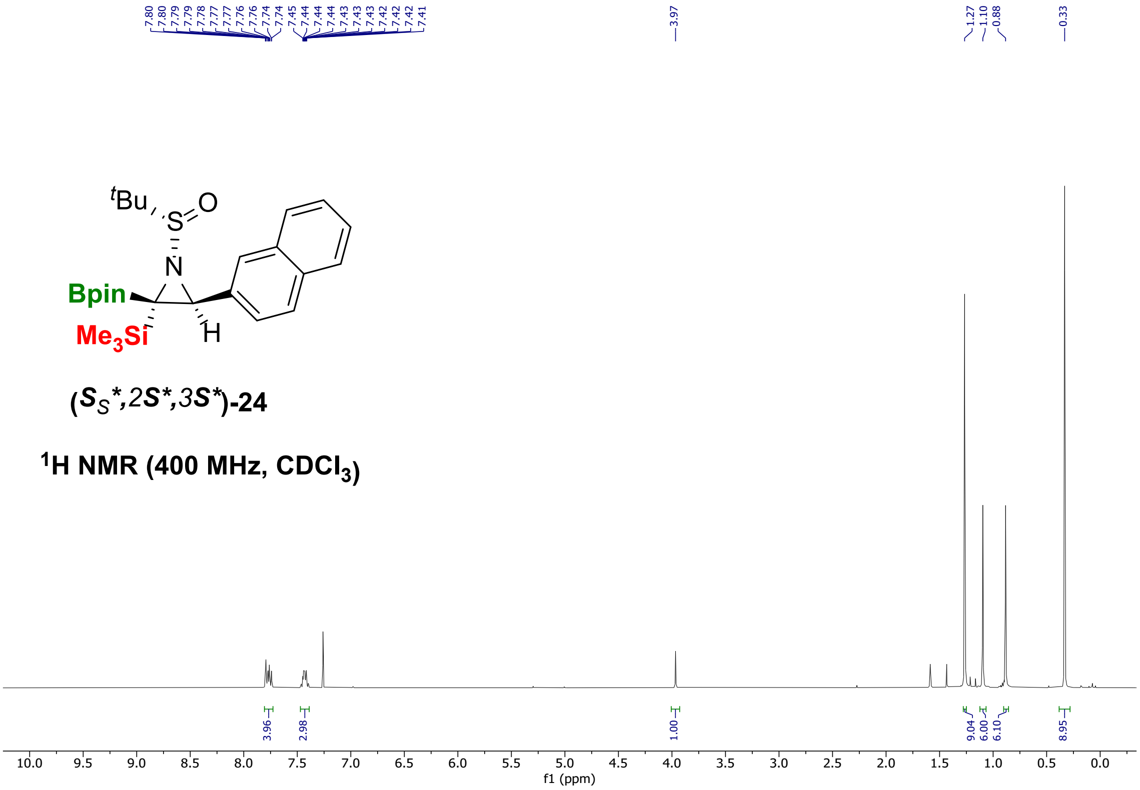
tBu
S
O
N
Bpin
Me3Si
H
(SS*,2S*,3S*)-24
1H NMR (400 MHz, CDCl3)
7.80 7.80 7.79 7.79 7.78 7.77 7.77 7.76 7.76 7.74 7.74 7.45 7.44 7.44 7.44 7.43 7.43 7.43 7.42 7.42 7.42 7.41
3.97
1.27 1.10 0.88
0.33
3.96 2.98 1.00 9.04 6.00 6.10 8.95
f1 (ppm)

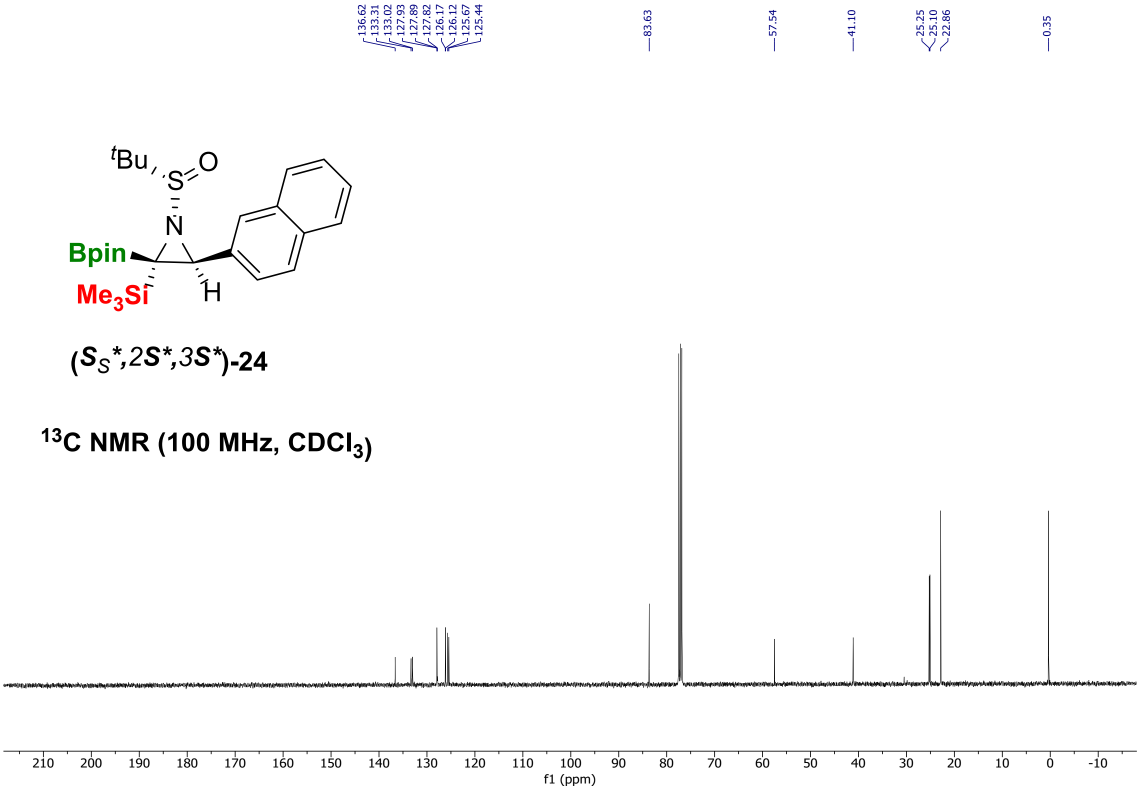
136.62 133.31 133.02 127.93 127.89 127.82 126.17 126.12 125.67 125.44
83.63
57.54
41.10
25.25 25.10 22.86
0.35
tBu
S
O
N
Bpin
Me3Si
H
(SS*,2S*,3S*)-24
13C NMR (100 MHz, CDCl3)
f1 (ppm)

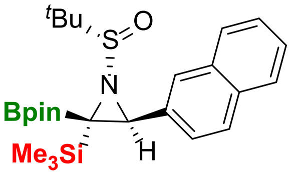

(S_S*,2S*,3S*)-24

^{11}B NMR (129 MHz, $CDCl_3$)

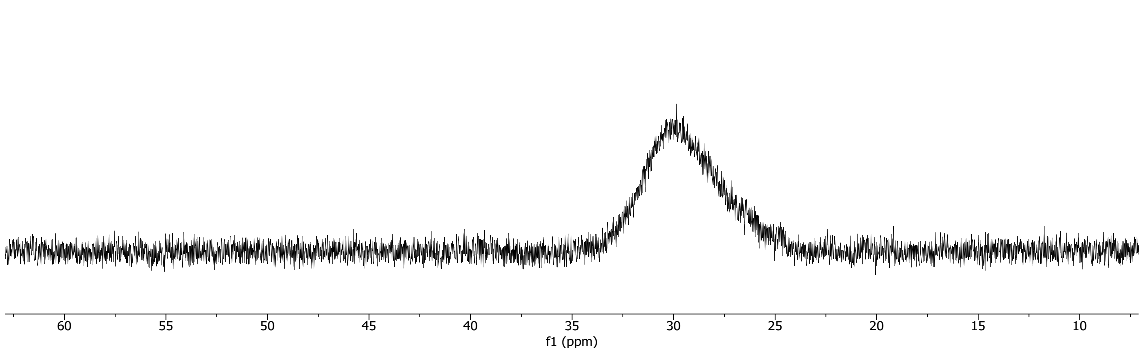

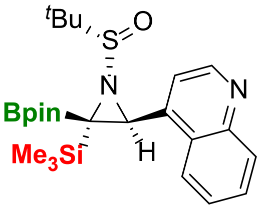

(S_S*,2S*,3S*)-26

^{1}H NMR (400 MHz, $CDCl_3$)

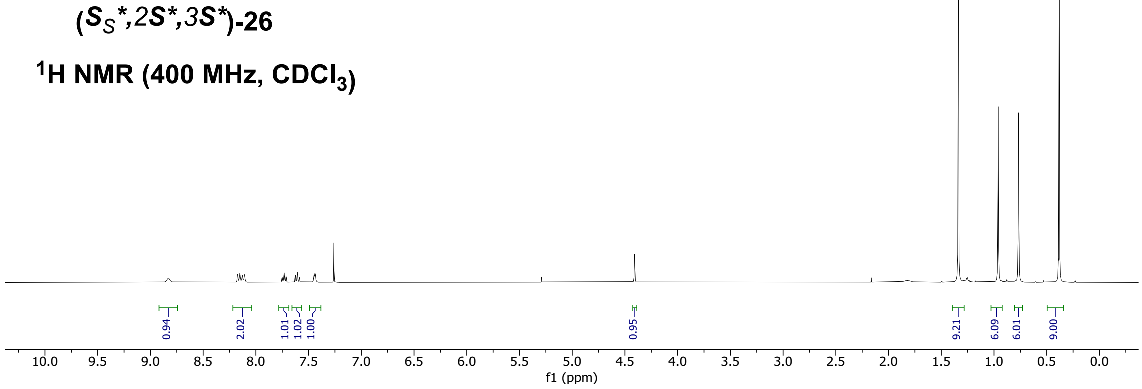

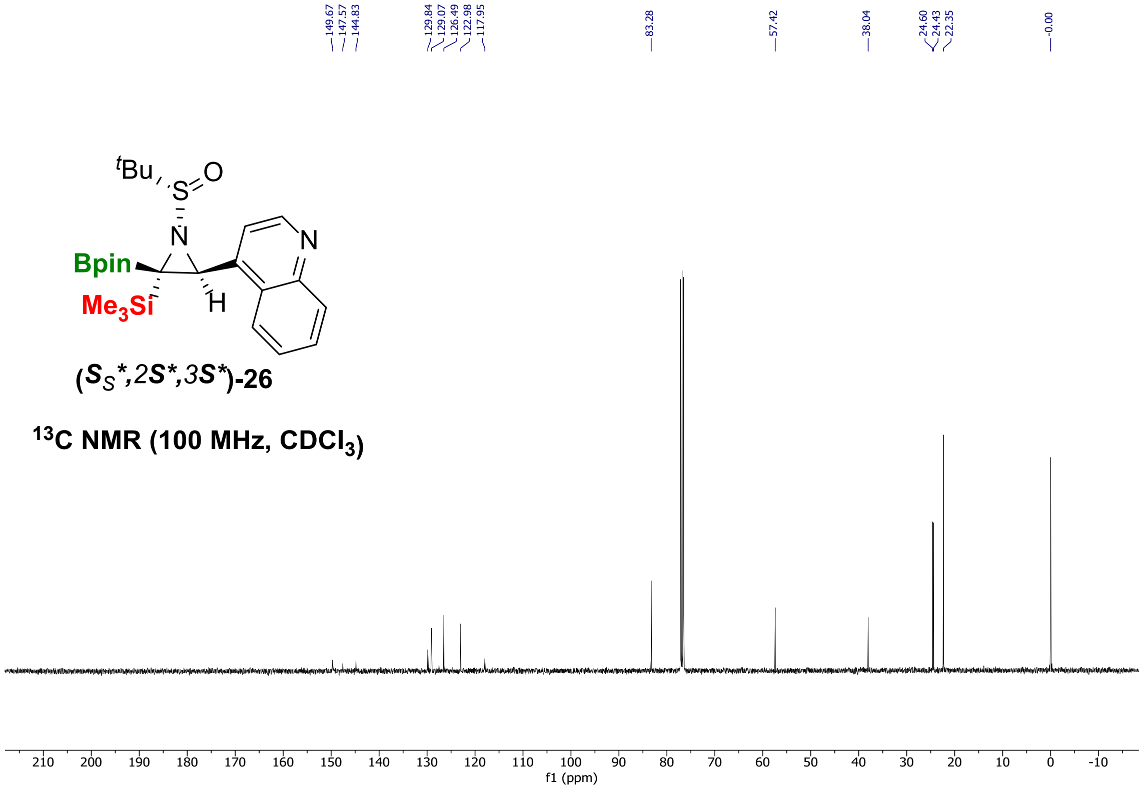
149.67
147.57
144.83
129.84
129.07
126.49
122.98
117.95
83.28
57.42
38.04
24.60
24.43
22.35
-0.00
tBu
S
O
N
N
Bpin
Me3Si
H
(SS*,2S*,3S*)-26
13C NMR (100 MHz, CDCl3)
f1 (ppm)

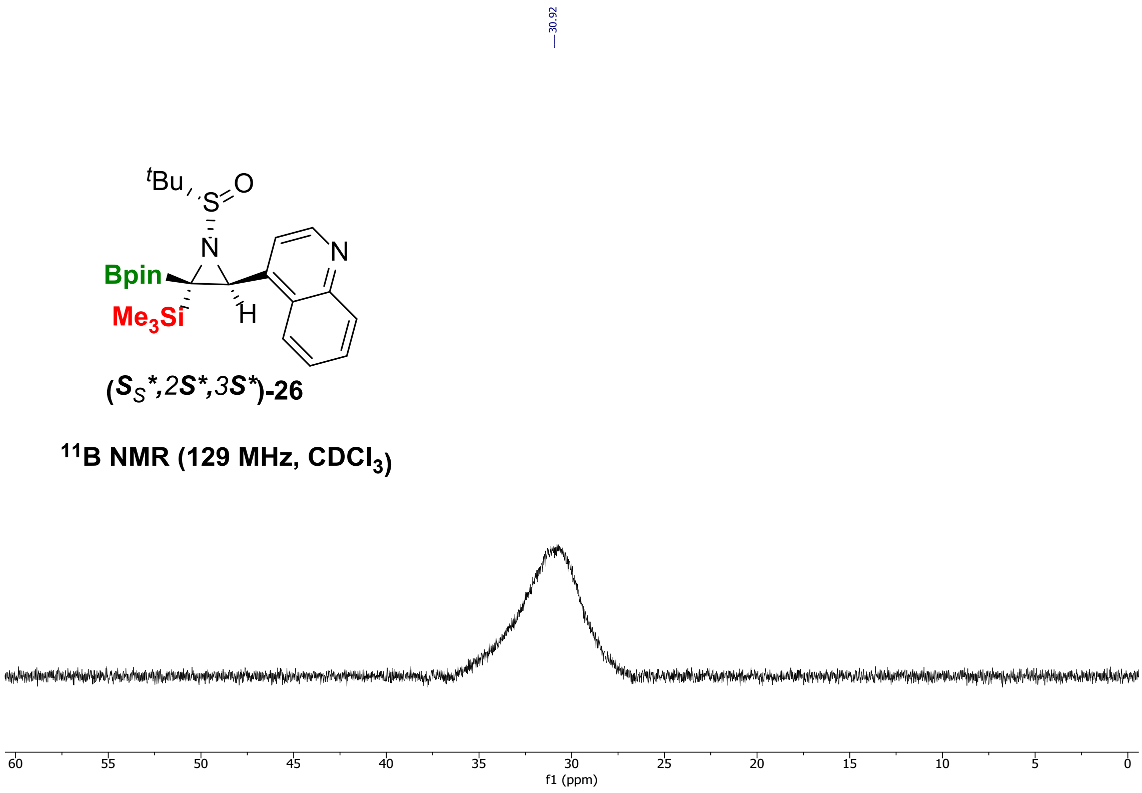
30.92
tBu
S
O
N
N
Bpin
Me3Si
H
(SS*,2S*,3S*)-26
11B NMR (129 MHz, CDCl3)
f1 (ppm)

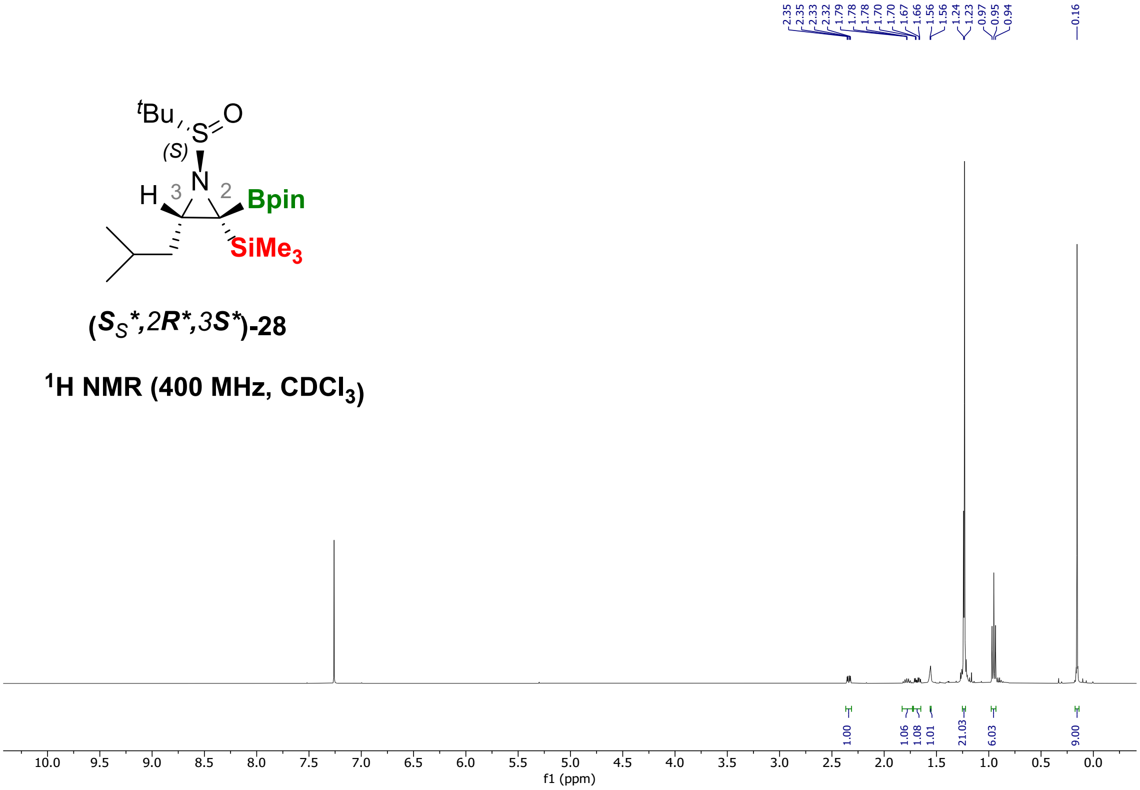

(S_S*,2R*,3S*)-28

^{1}H NMR (400 MHz, $CDCl_3$)

2D NOESY experiment for

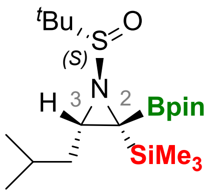

(S_S*,2R*,3S*)-28

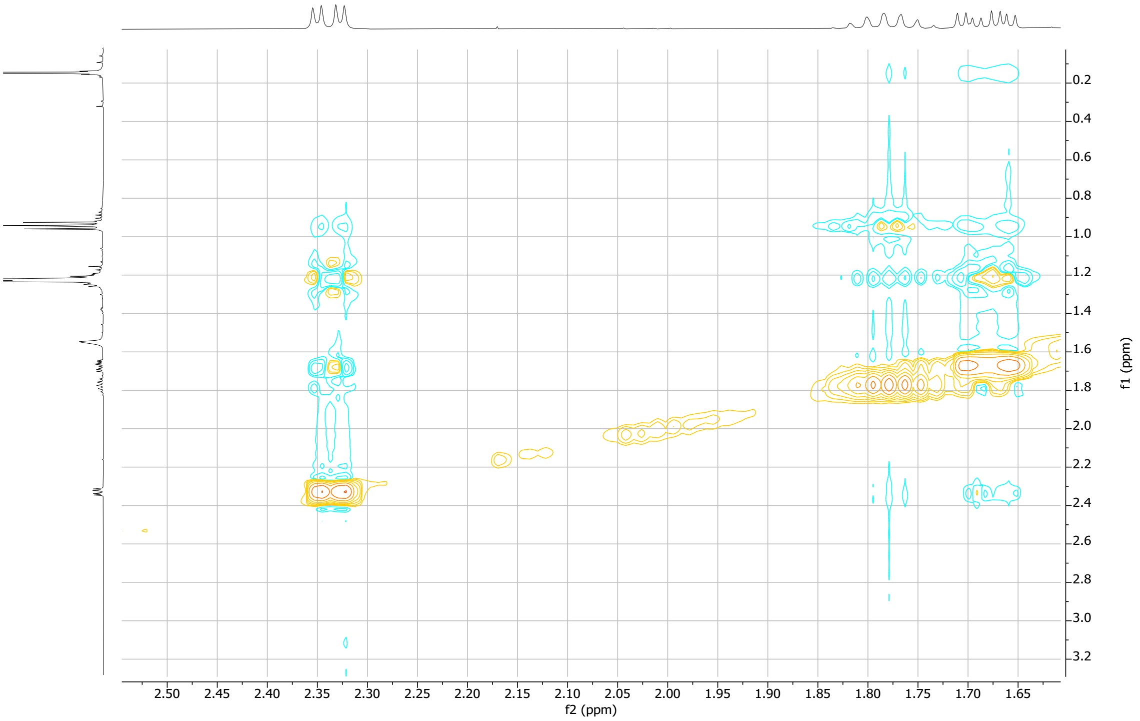

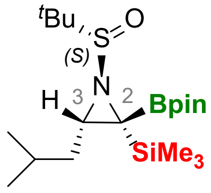

(S_S*,2R*,3S*)-28

^{13}C NMR (100 MHz, $CDCl_3$)

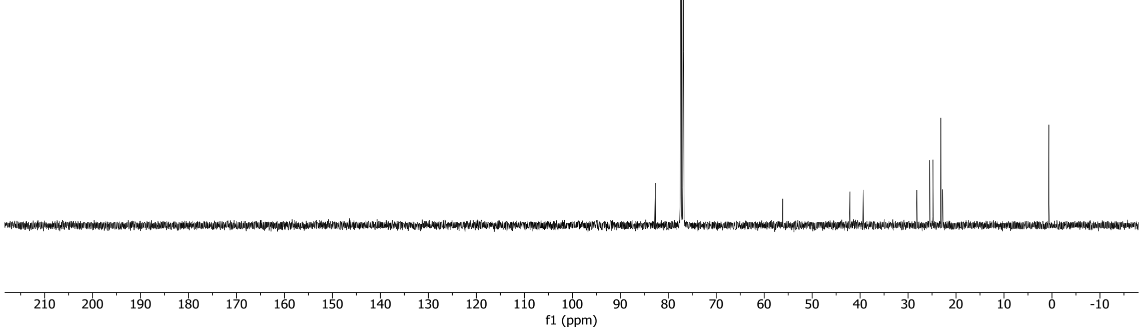

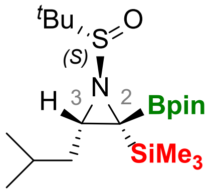

(S_S*,2R*,3S*)-28

^{11}B NMR (129 MHz, $CDCl_3$)

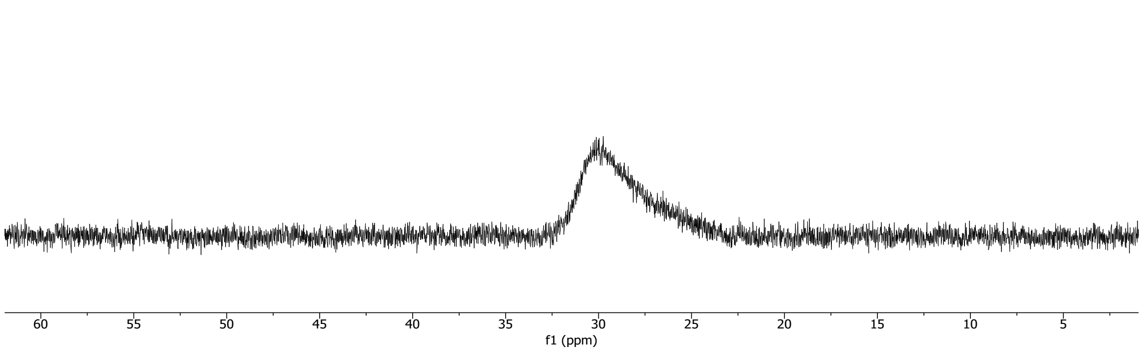

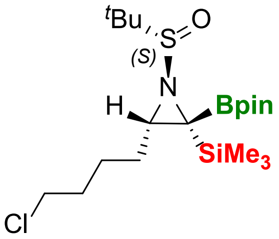

(S_S*,2R*,3S*)-30

^{1}H NMR (400 MHz, CDCl$_3$)

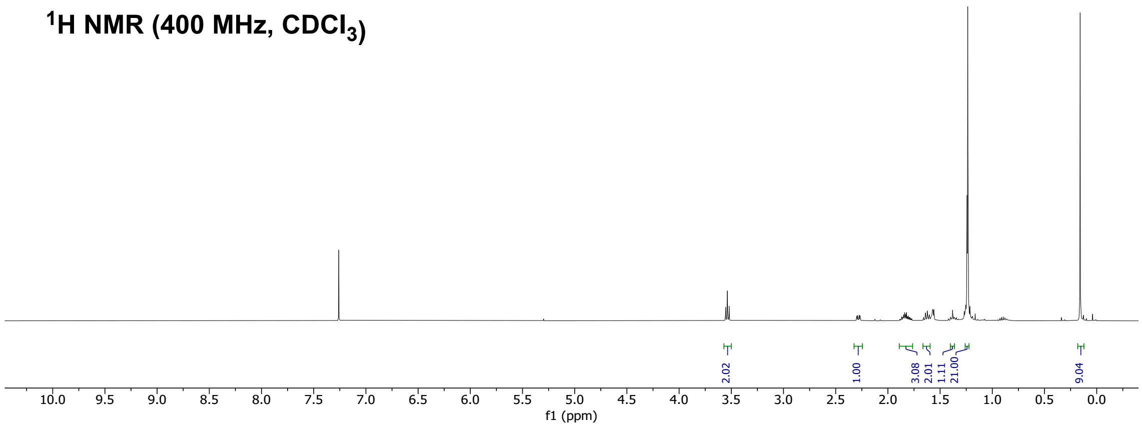

2D NOESY experiment for

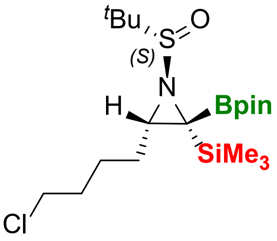

(S_S*,2R*,3S*)-30

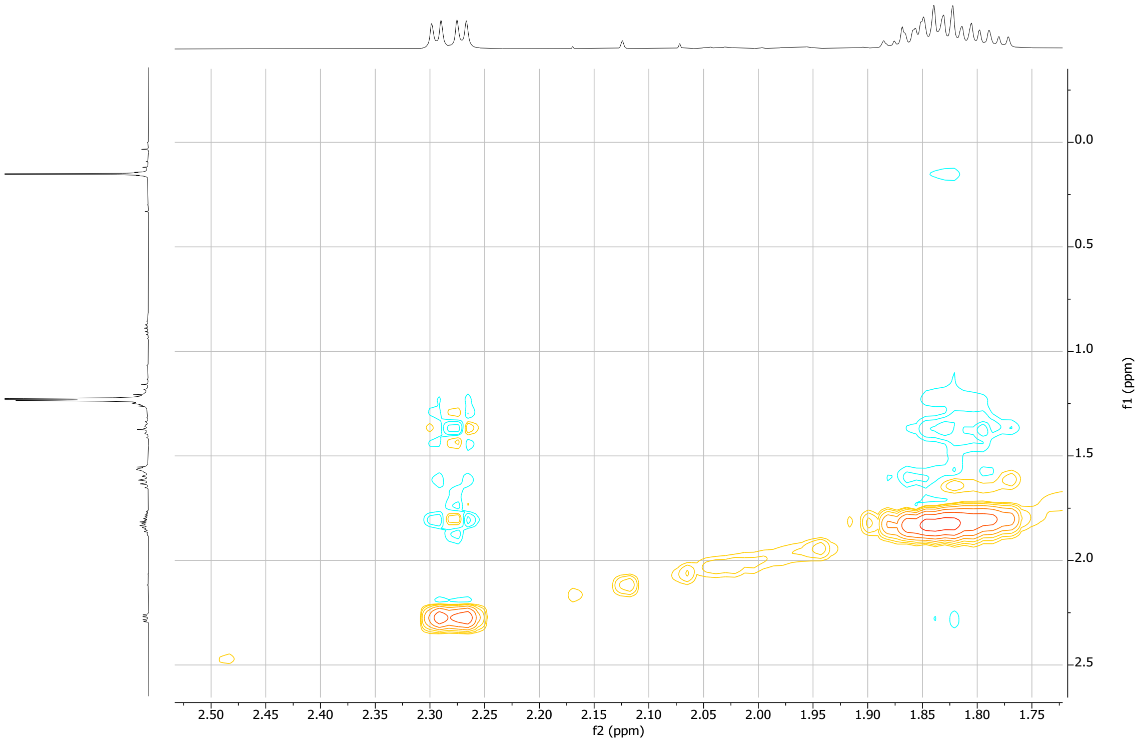

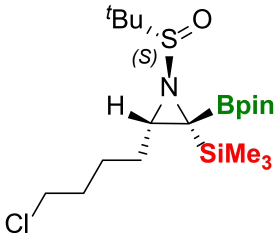

(S_S*,2R*,3S*)-30

^{13}C NMR (100 MHz, CDCl$_3$)

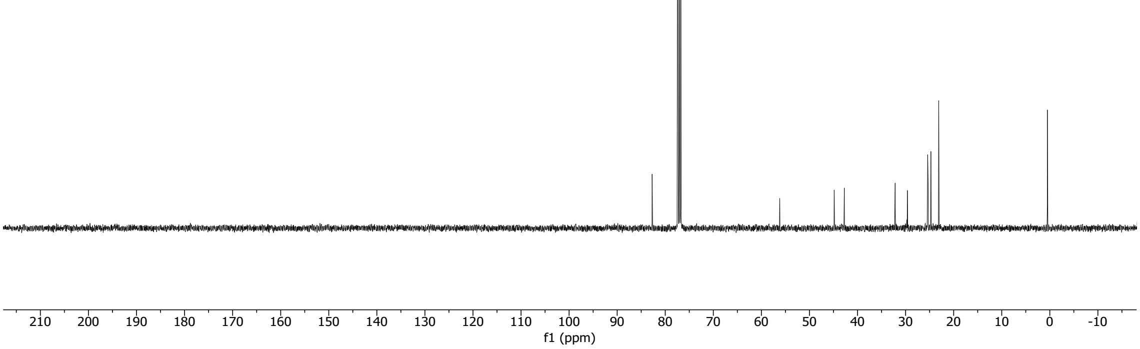

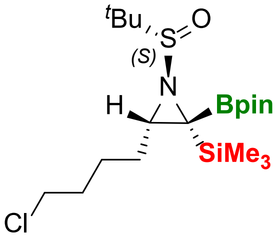

(S_S*,2R*,3S*)-30

^{11}B NMR (129 MHz, CDCl$_3$)

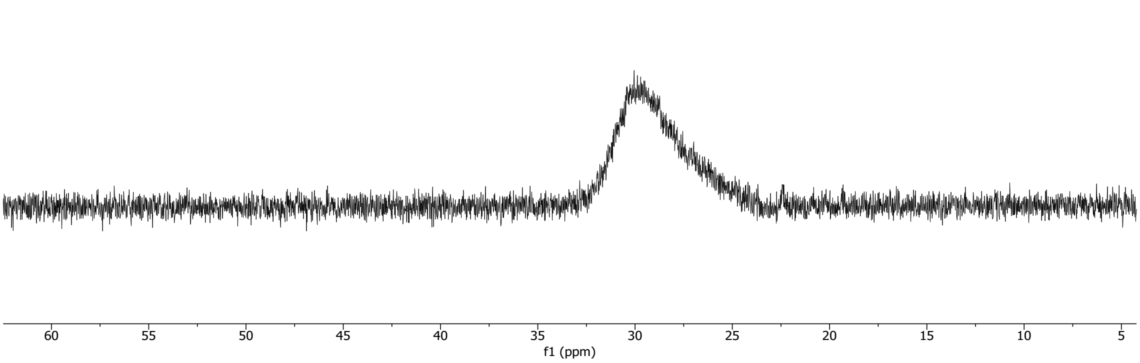

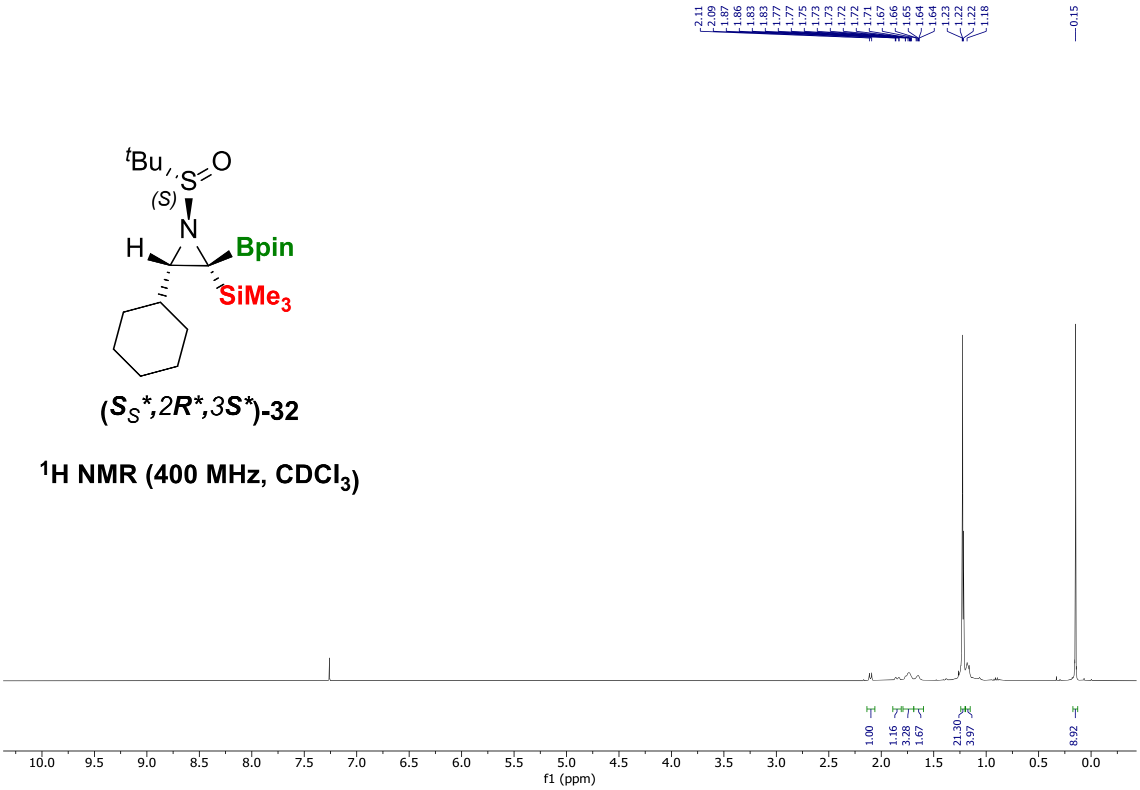

2.11
2.09
1.87
1.86
1.83
1.83
1.83
1.77
1.77
1.75
1.73
1.73
1.73
1.72
1.72
1.71
1.67
1.66
1.65
1.64
1.64
1.23
1.22
1.22
1.18
0.15
tBu
S
O
(S)
N
H
Bpin
SiMe3
(SS*,2R*,3S*)-32
1H NMR (400 MHz, CDCl3)
1.00
1.16
3.28
1.67
21.30
3.97
8.92
f1 (ppm)

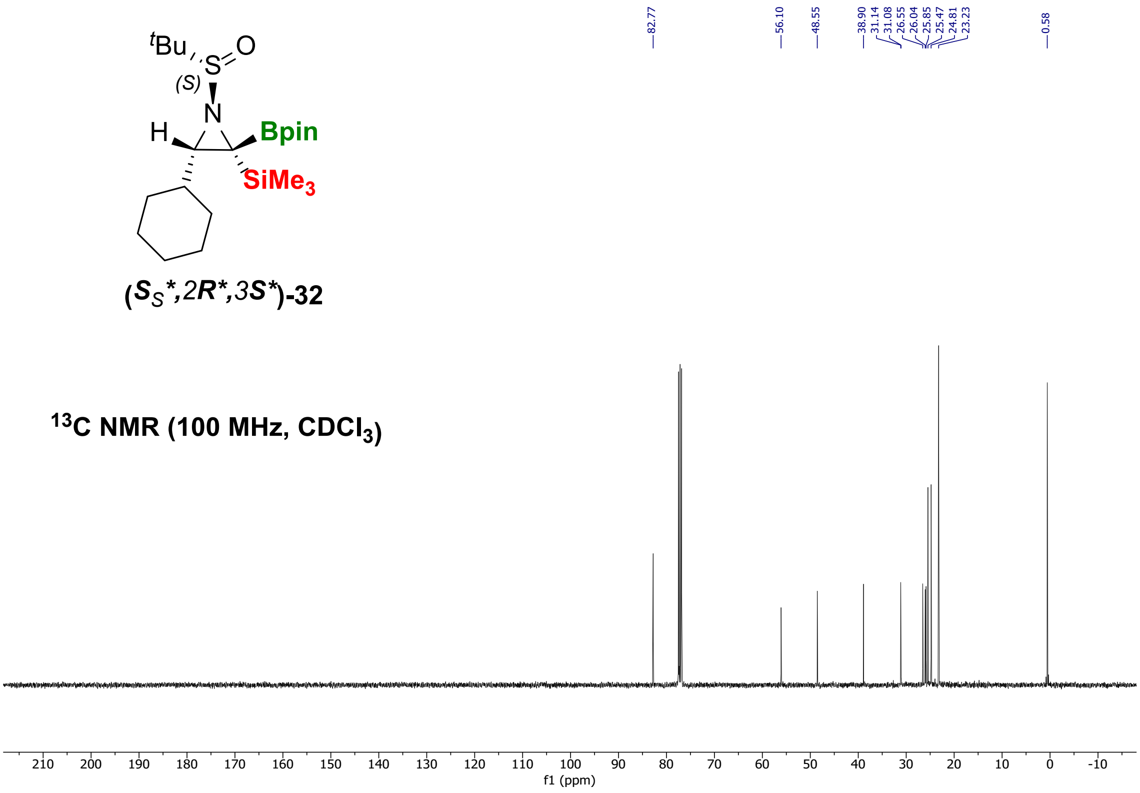

82.77
56.10
48.55
38.90
31.14
31.08
26.55
26.04
25.85
25.47
24.81
23.23
0.58
tBu
S
O
(S)
N
H
Bpin
SiMe3
(SS*,2R*,3S*)-32
13C NMR (100 MHz, CDCl3)
f1 (ppm)

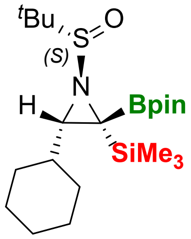

(S_S*,2*R,3*S**)-32**

^{11}B NMR (129 MHz, CDCl$_3$)

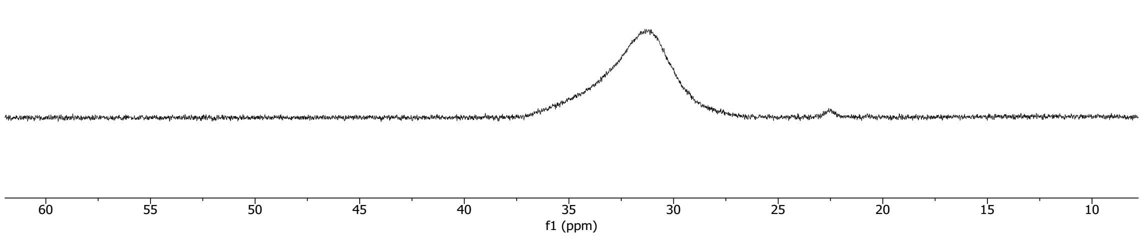

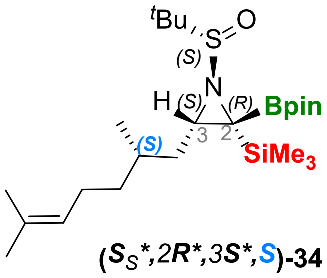

(S_S*,2*R,3*S**,*S*)-34**

^{1}H NMR (400 MHz, CDCl$_3$)

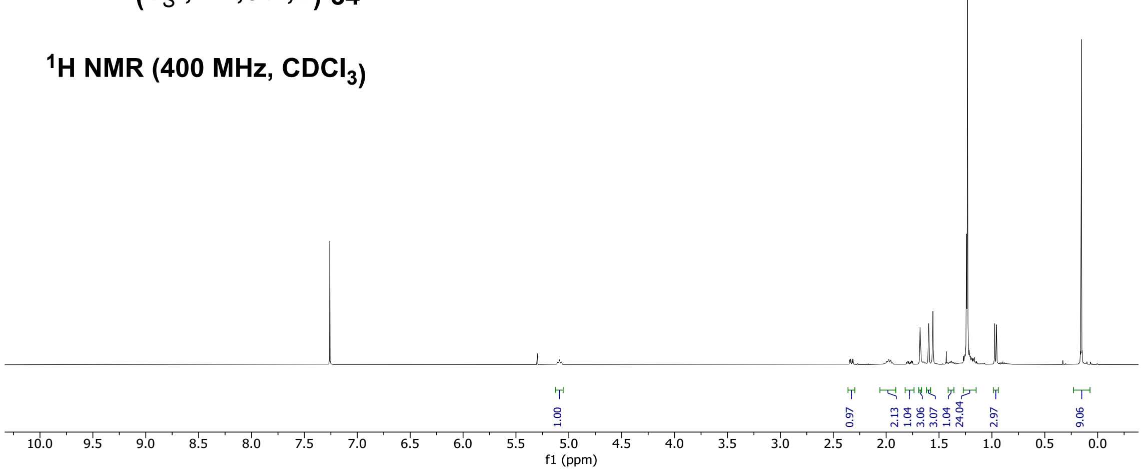

2D NOESY experiment for

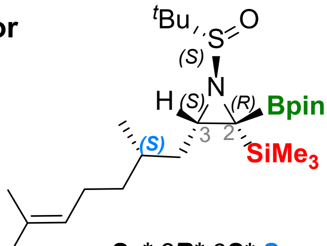

(S_S*,2*R,3*S**,S)-34**

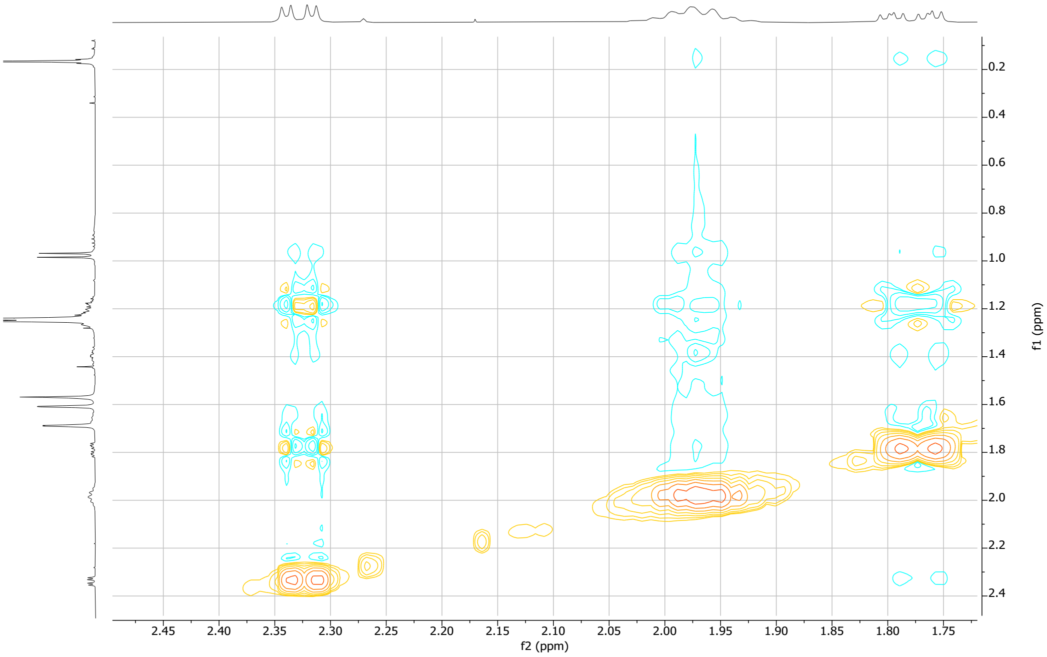

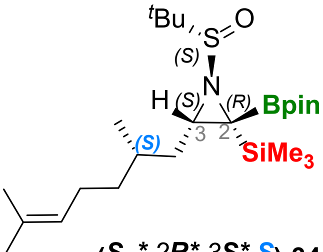

(S_S*,2*R,3*S**,S)-34**

^{13}C NMR (100 MHz, $CDCl_3$)

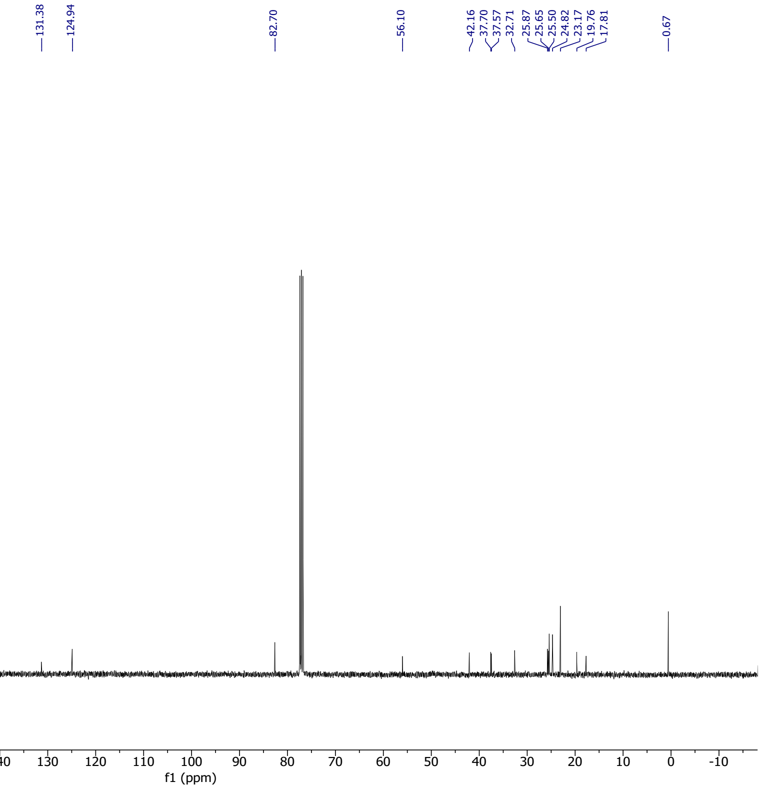

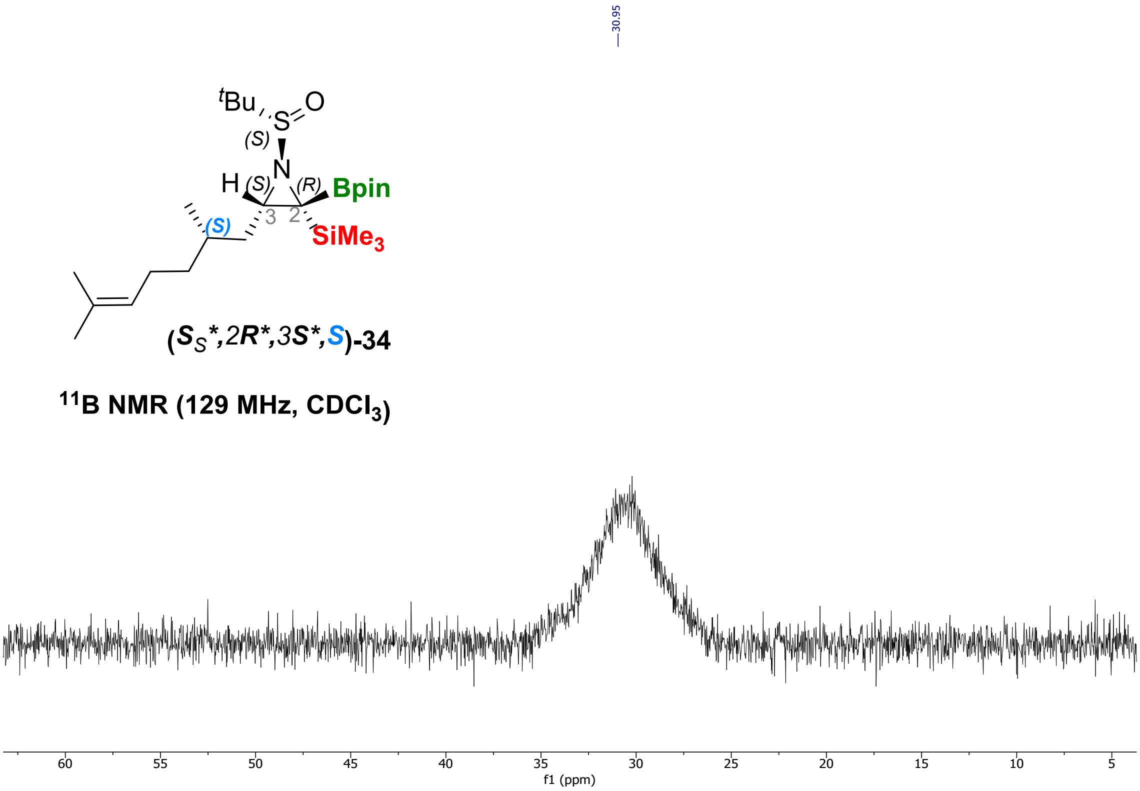
(S_S*,2R*,3S*,S)-34
^{11}B NMR (129 MHz, $CDCl_3$)

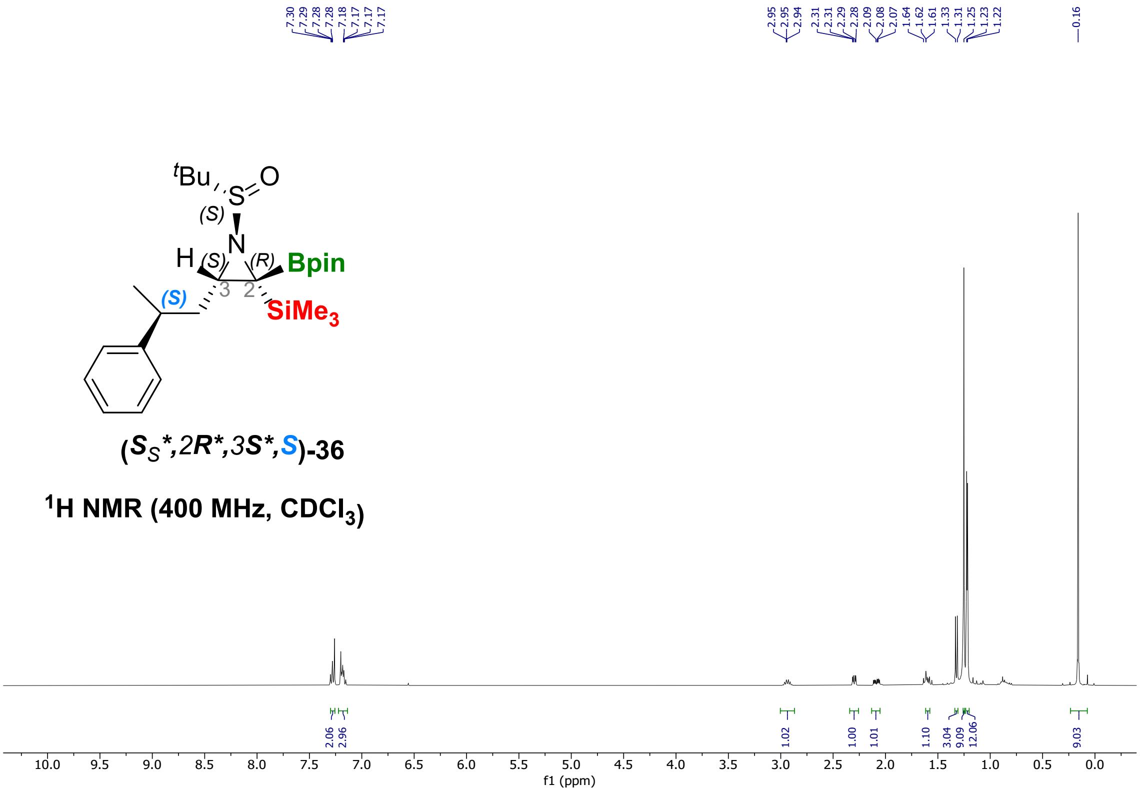
(S_S*,2R*,3S*,S)-36
^{1}H NMR (400 MHz, $CDCl_3$)

2D NMR NOESY experiment for

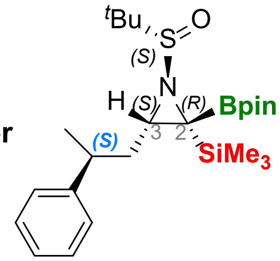

(S_S*,2*R,3*S**,*S*)-36**

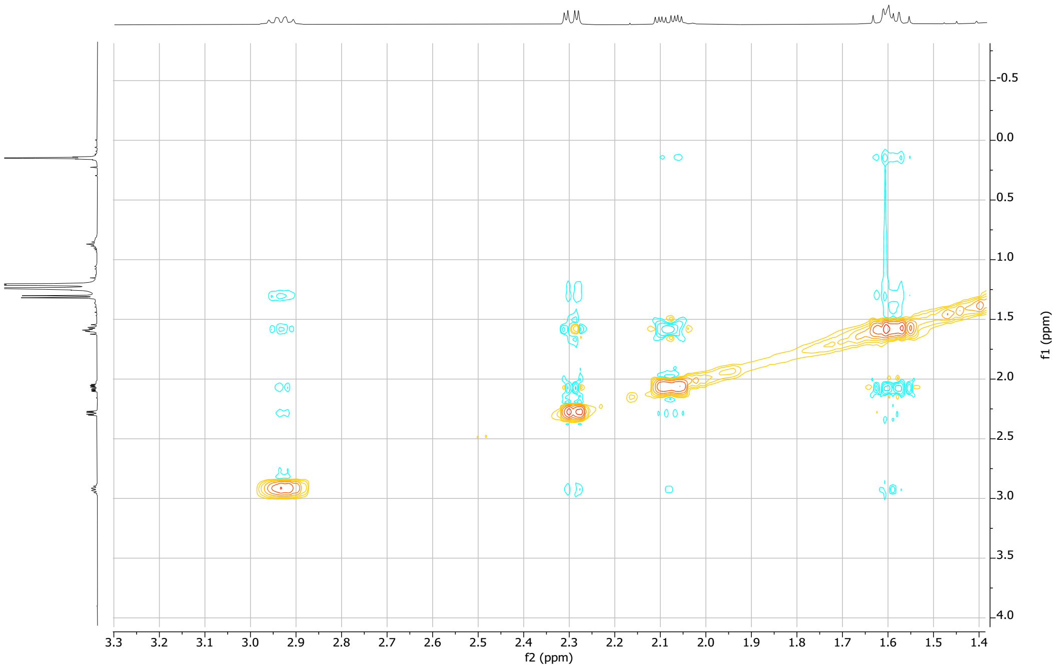

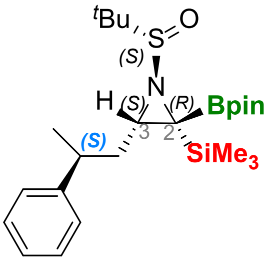

(S_S*,2*R,3*S**,*S*)-36**

^{13}C NMR (100 MHz, $CDCl_3$)

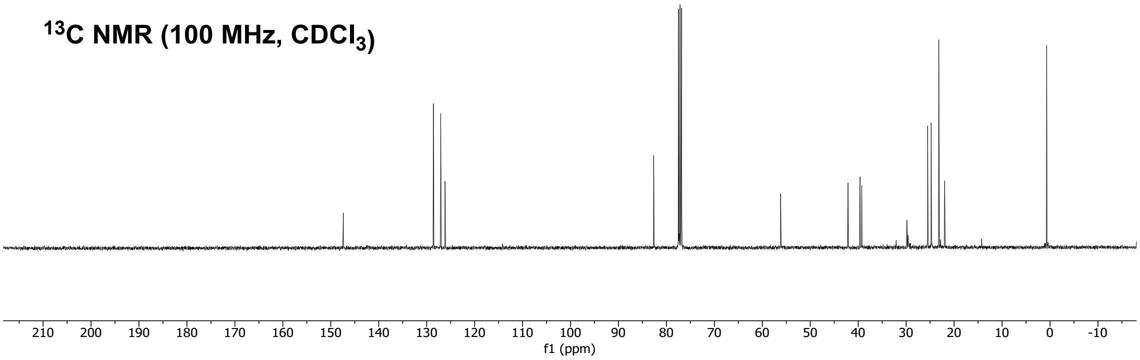

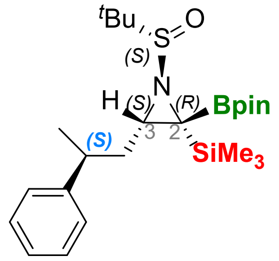

(S_S*,2R*,3S*,S)-36

^{11}B NMR (129 MHz, CDCl$_3$)

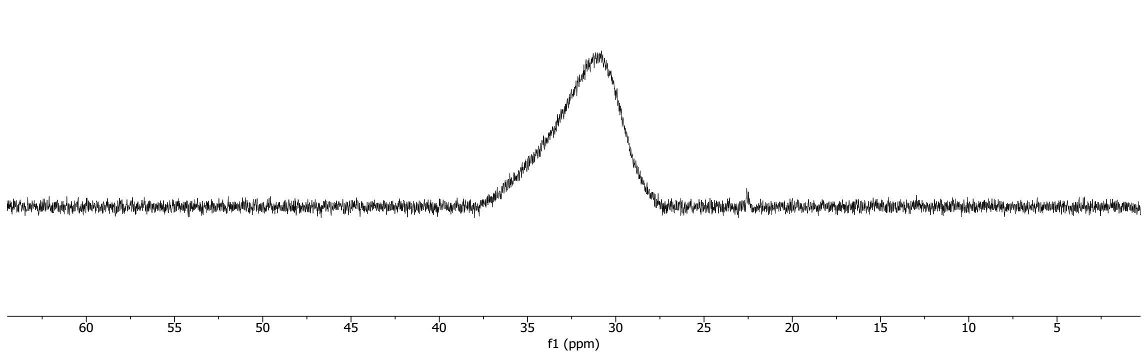

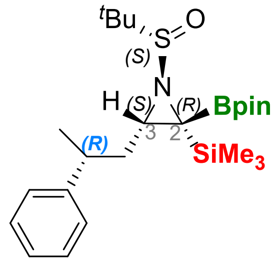

(S_S*,2R*,3S*,R)-36

^{1}H NMR (400 MHz, CDCl$_3$)

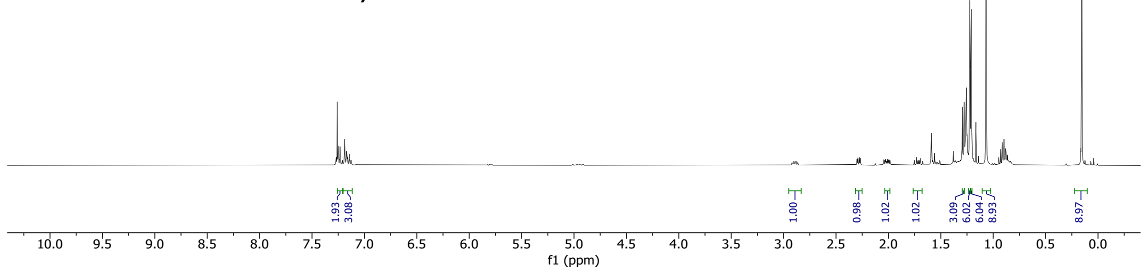

2D NOESY experiment for

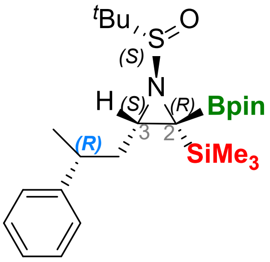

(S_S*,2R*,3S*,R)-36

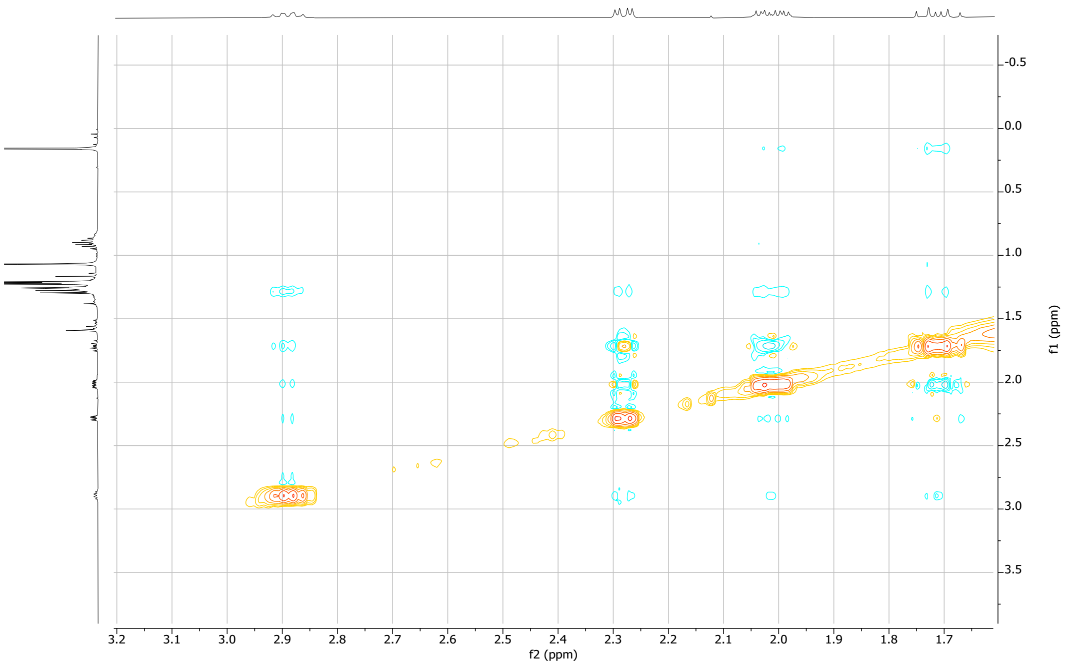

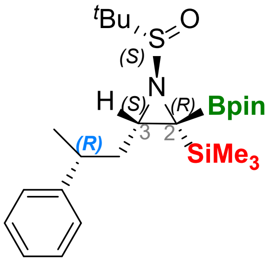

(S_S*,2R*,3S*,R)-36

^{13}C NMR (100 MHz, $CDCl_3$)

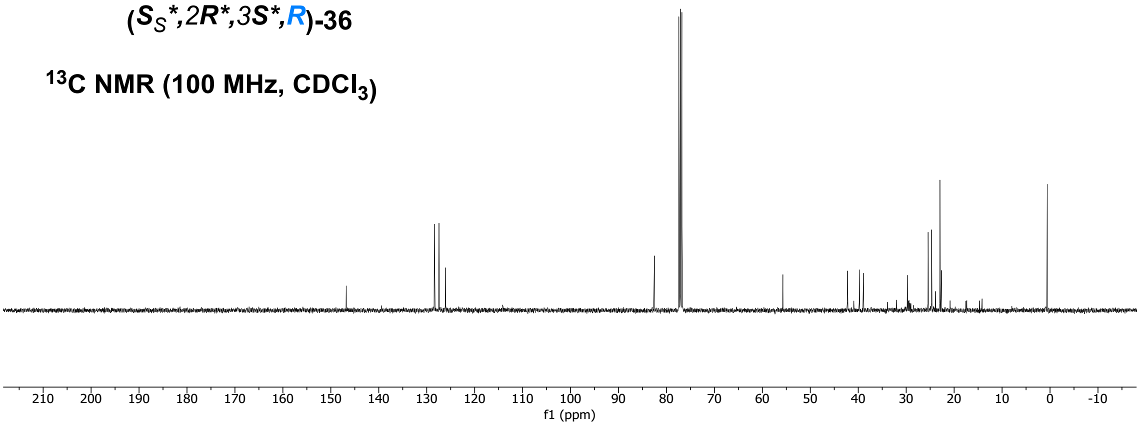

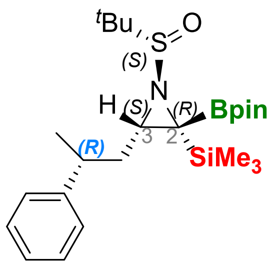

(S_S*,2R*,3S*,R)-36

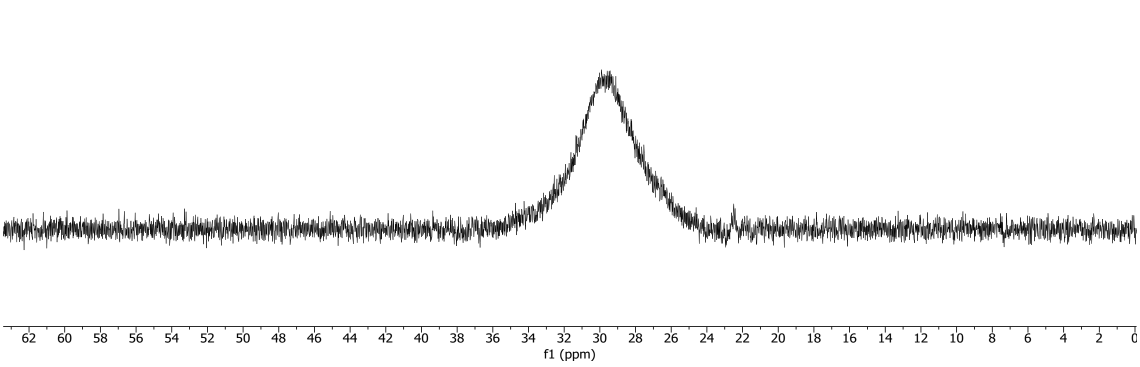

^{11}B NMR (129 MHz, $CDCl_3$)

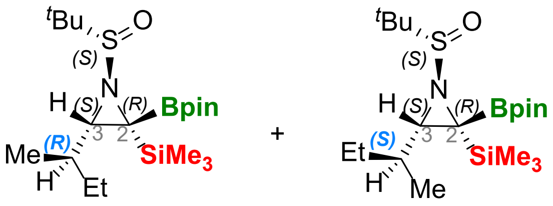

(S_S*,2R*,3S*,R)-38 + (S_S*,2R*,3S*,S)-38

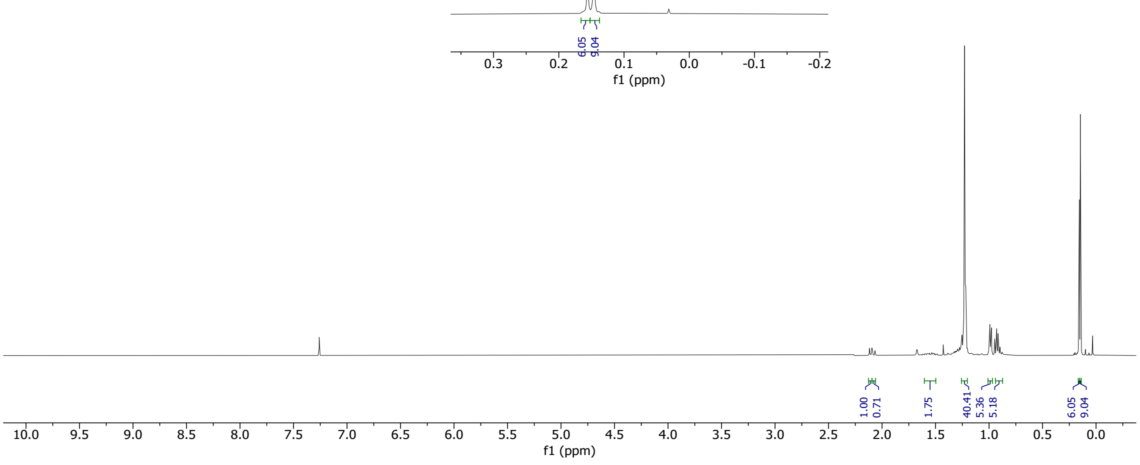

^{1}H NMR (400 MHz, $CDCl_3$)

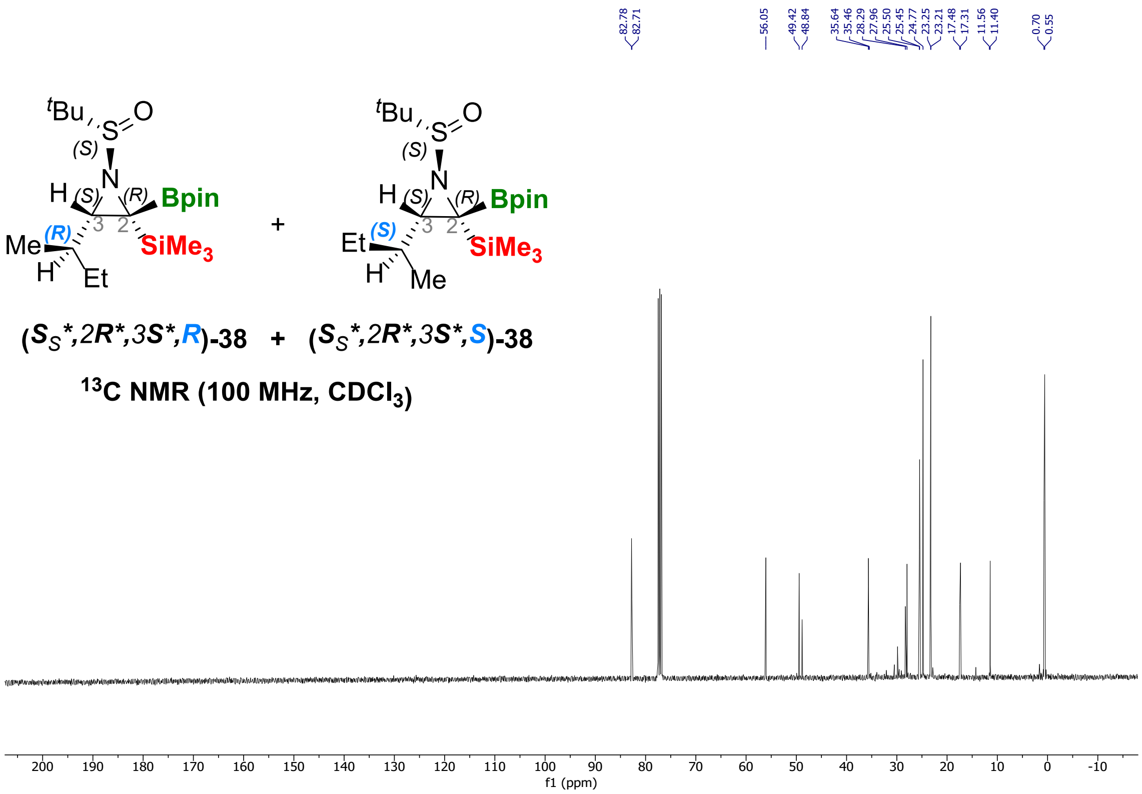
82.78
82.71
56.05
49.42
48.84
35.64
35.46
28.29
27.96
25.50
25.45
24.77
23.25
23.21
17.48
17.31
11.56
11.40
0.70
0.55
tBu
S
O
(S)
N
H
(S)
(R)
Bpin
3
2
Me
(R)
SiMe3
H
Et
+
Et
(S)
H
Me
(SS*,2R*,3S*,R)-38 + (SS*,2R*,3S*,S)-38
13C NMR (100 MHz, CDCl3)
f1 (ppm)

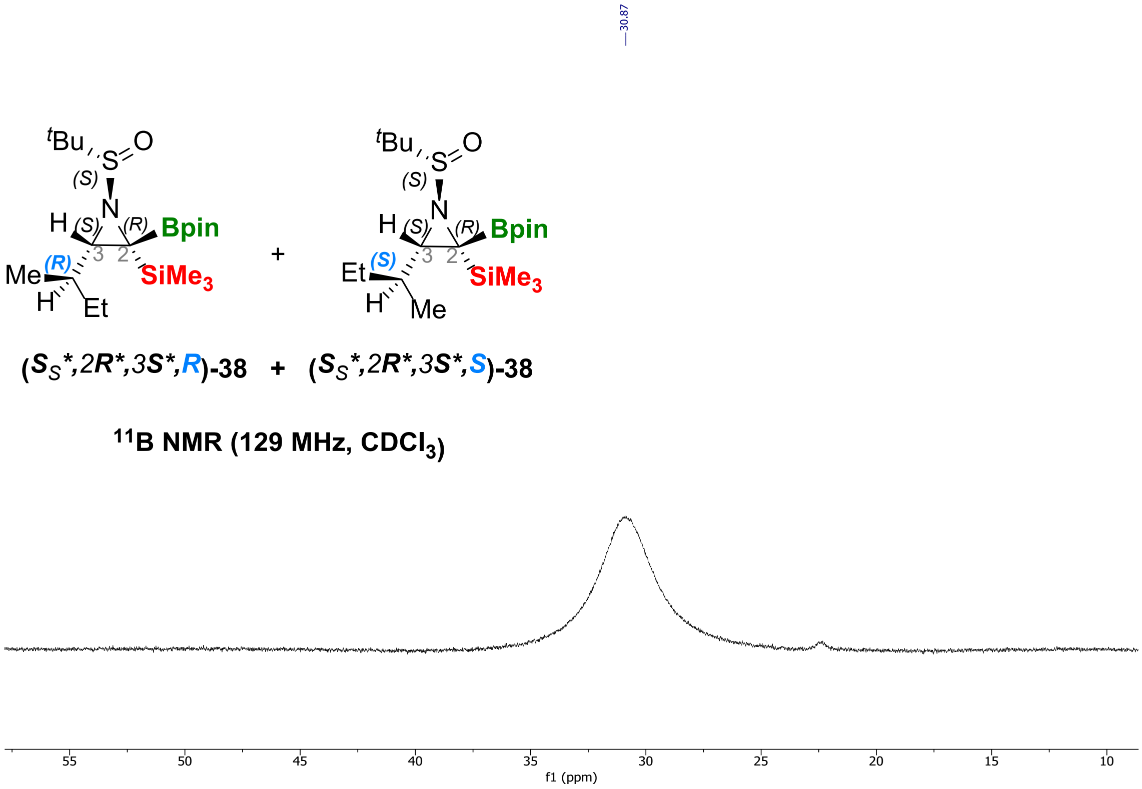
30.87
tBu
S
O
(S)
N
H
(S)
(R)
Bpin
3
2
Me
(R)
SiMe3
H
Et
+
Et
(S)
H
Me
(SS*,2R*,3S*,R)-38 + (SS*,2R*,3S*,S)-38
11B NMR (129 MHz, CDCl3)
f1 (ppm)

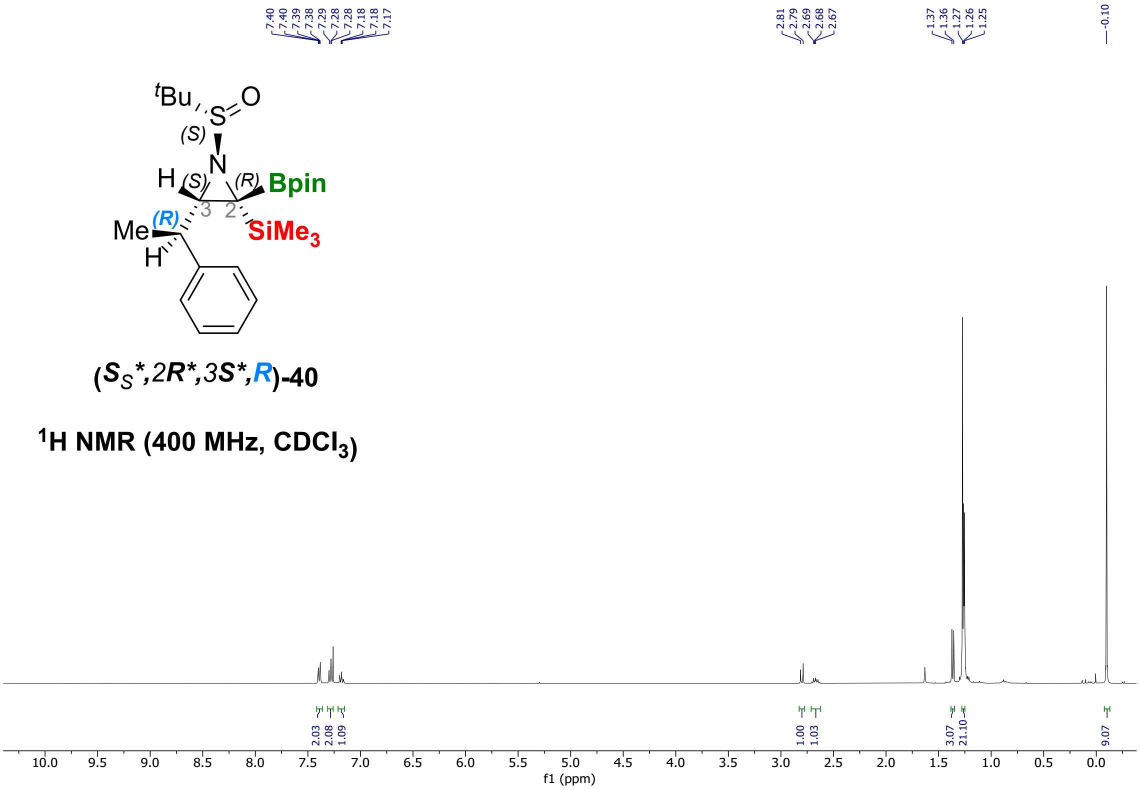

(S_S*,2R*,3S*,R)-40
1H NMR (400 MHz, CDCl3)
f1 (ppm)

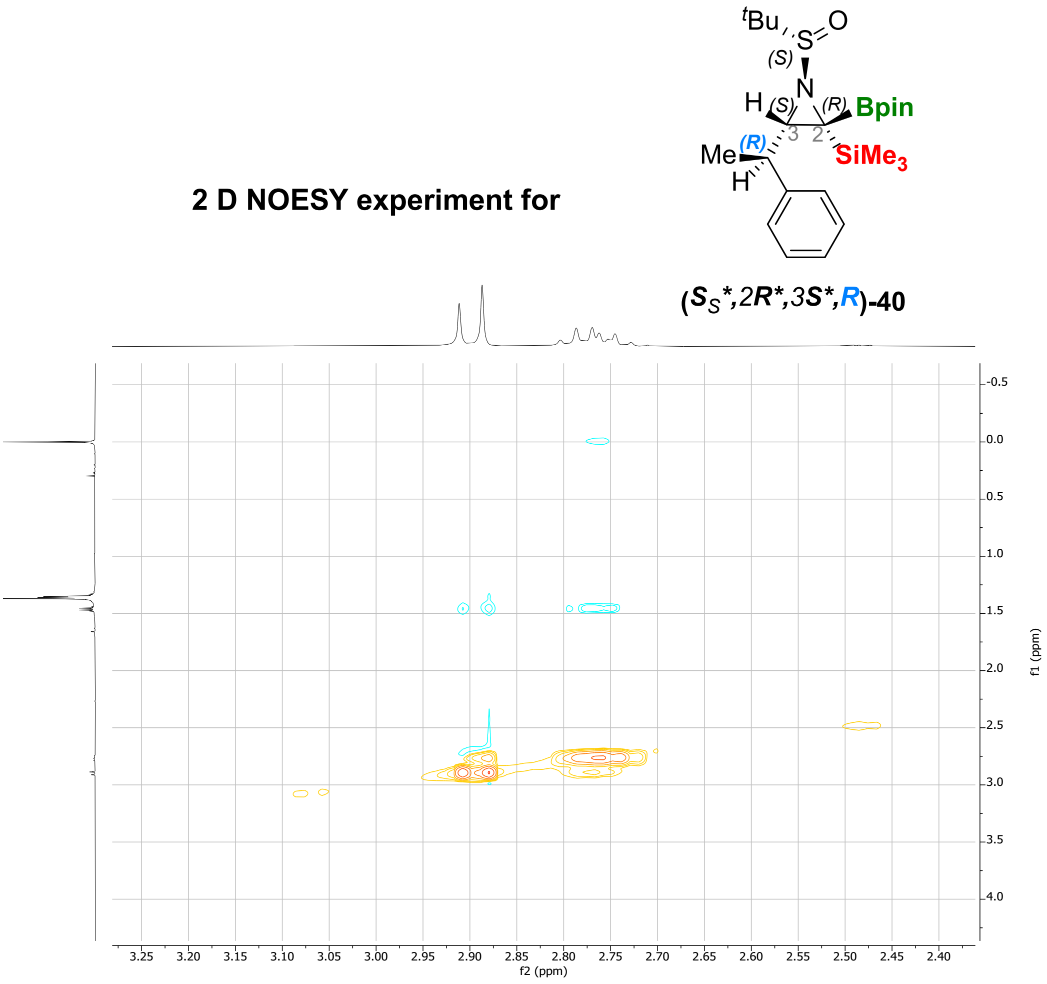

2 D NOESY experiment for
(S_S*,2R*,3S*,R)-40
f2 (ppm)
f1 (ppm)

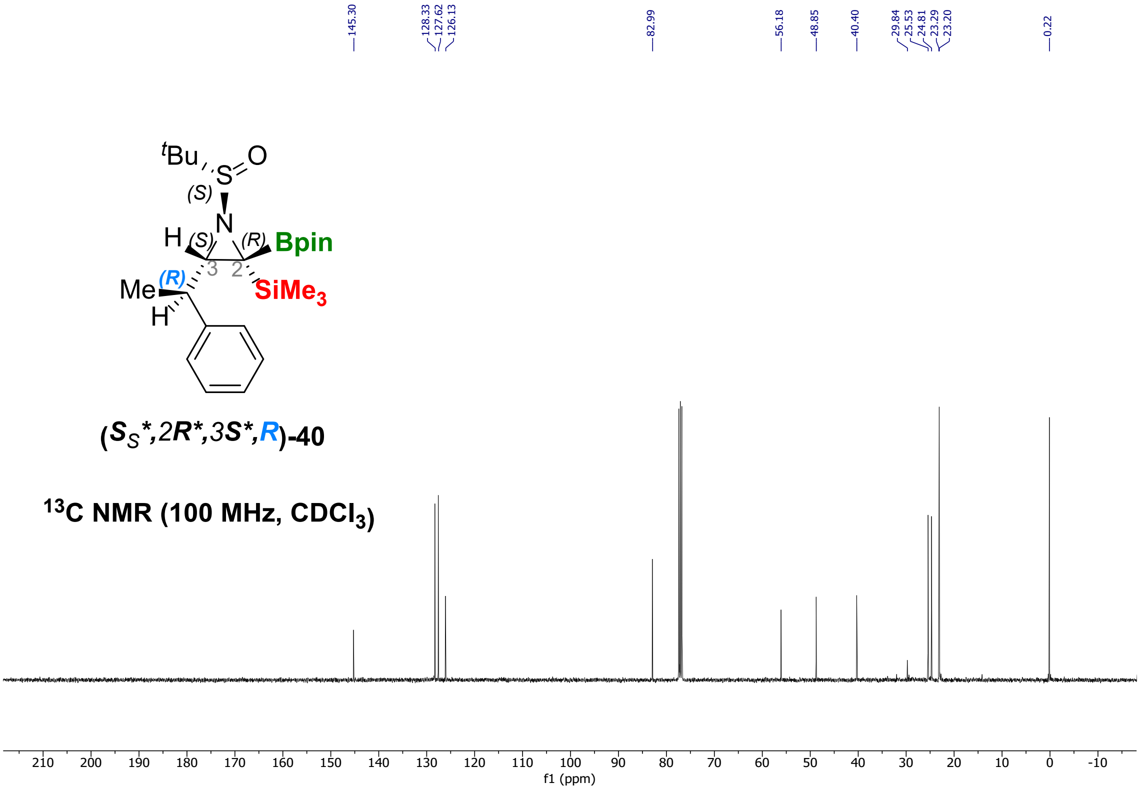
145.30
128.33
127.62
126.13
82.99
56.18
48.85
40.40
29.84
25.53
24.81
23.29
23.20
0.22
(S_S*,2R*,3S*,R)-40
13C NMR (100 MHz, CDCl3)
f1 (ppm)

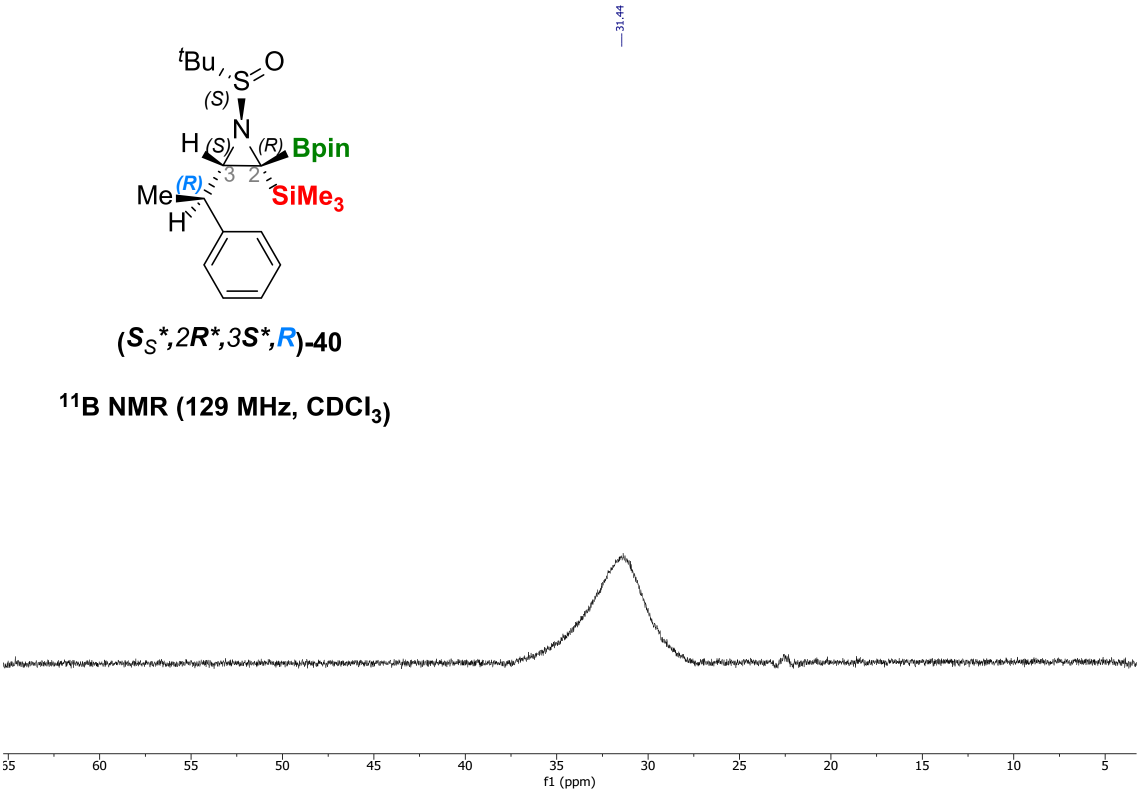
31.44
(S_S*,2R*,3S*,R)-40
11B NMR (129 MHz, CDCl3)
f1 (ppm)

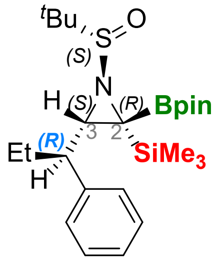

(S_S*,2*R,3*S**,*R*)-42**

^{1}H NMR (400 MHz, $CDCl_3$)

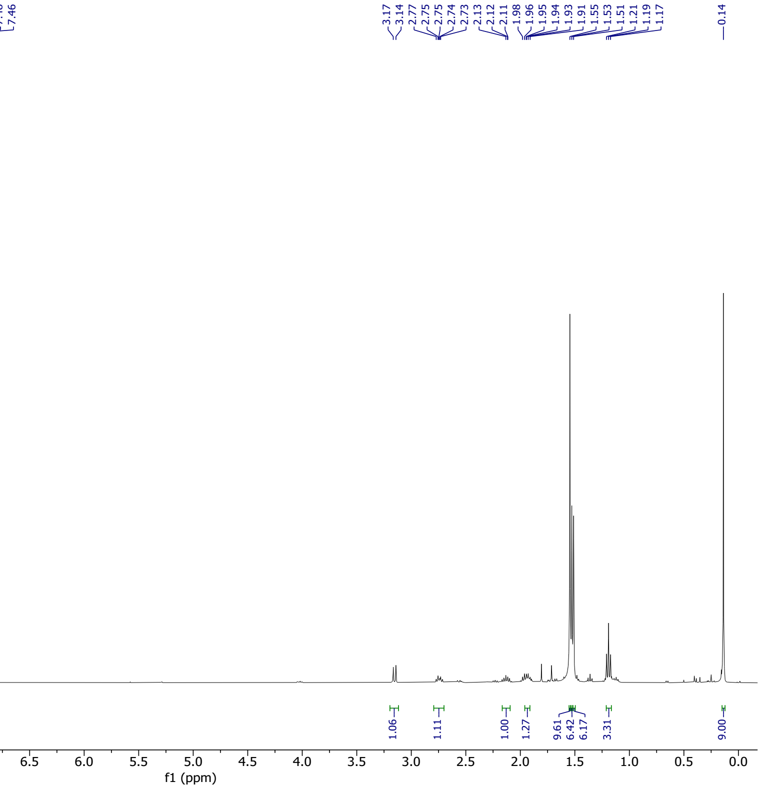

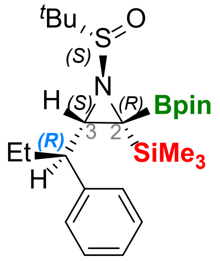

(S_S*,2*R,3*S**,*R*)-42**

^{13}C NMR (100 MHz, $CDCl_3$)

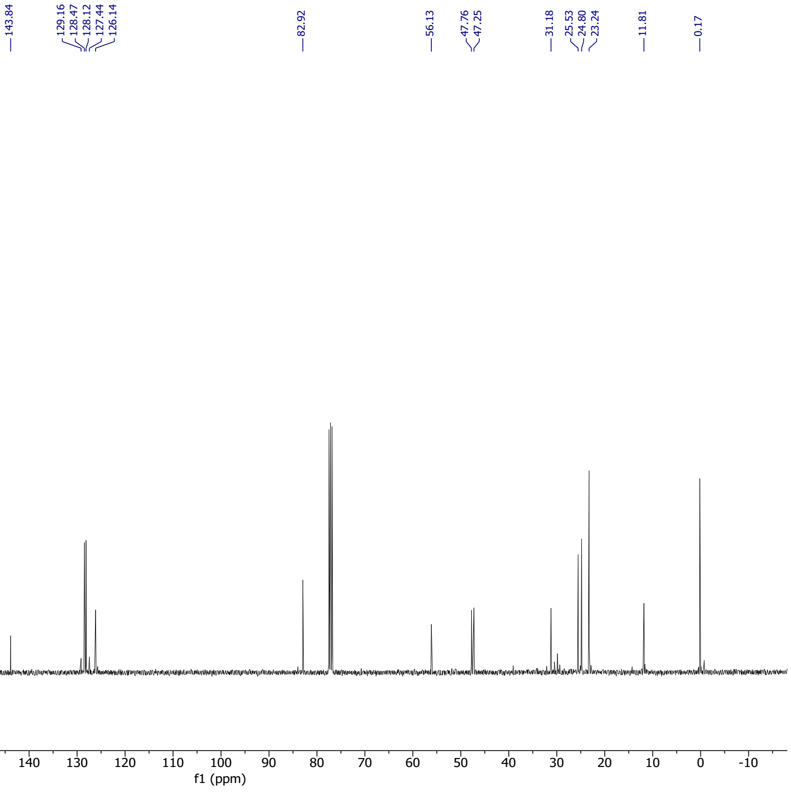

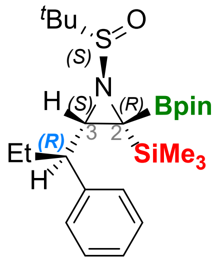

(S_S*,2*R,3*S**,*R*)-42**

^{11}B NMR (129 MHz, CDCl$_3$)

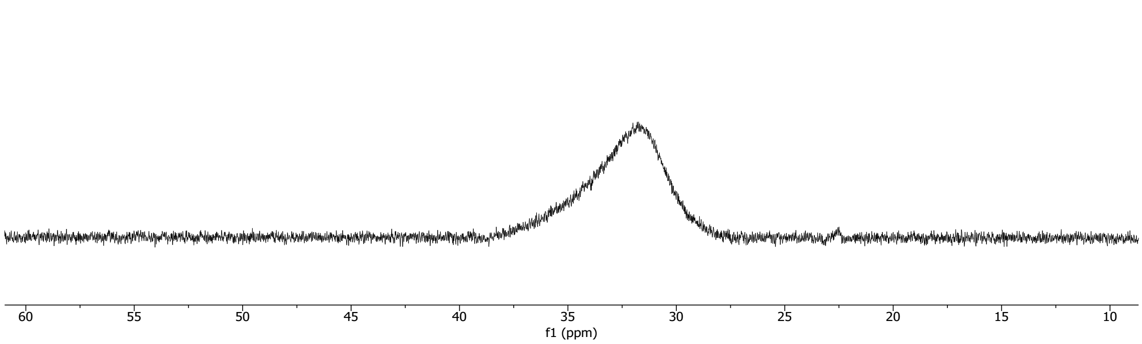

^{1}H, ^{13}C, ^{11}B NMR spectra for diversified aziridines

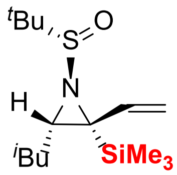

43

^{1}H NMR (400 MHz, CDCl$_3$)

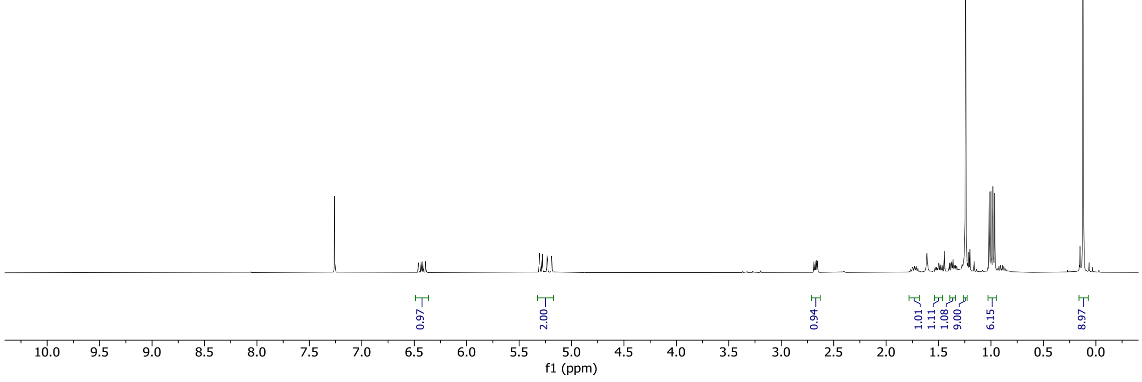

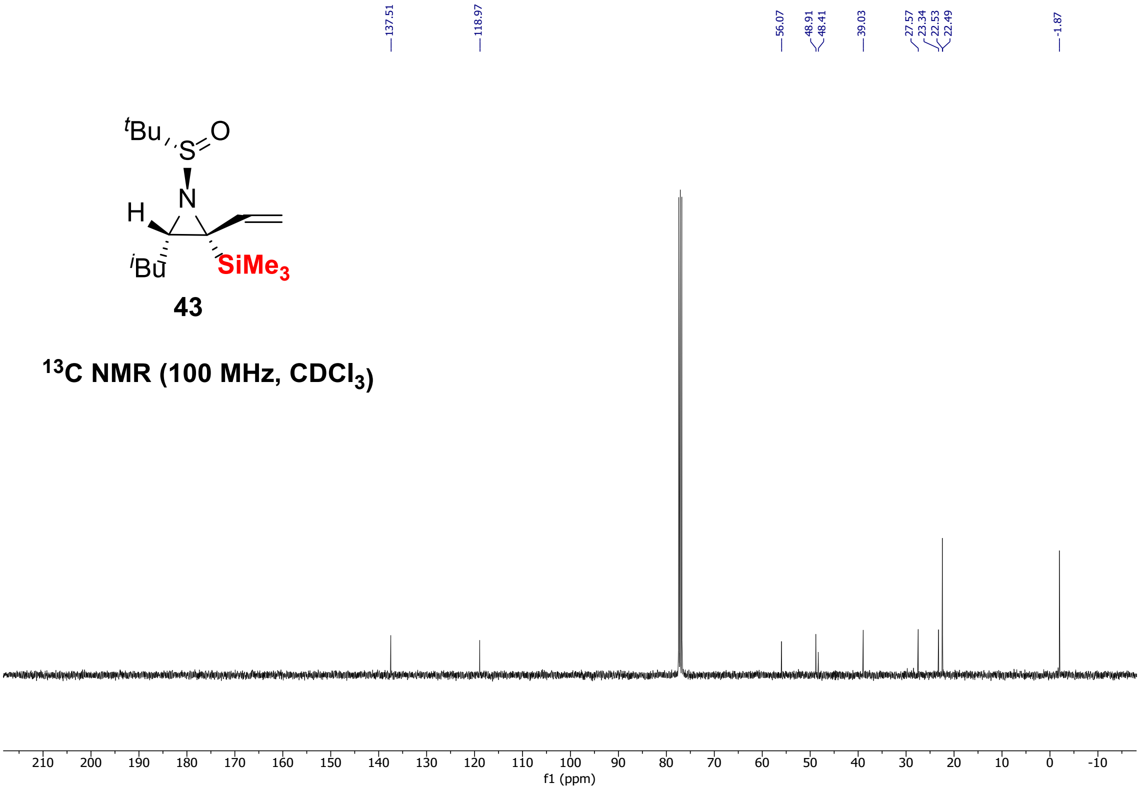

43

^{13}C NMR (100 MHz, CDCl$_3$)

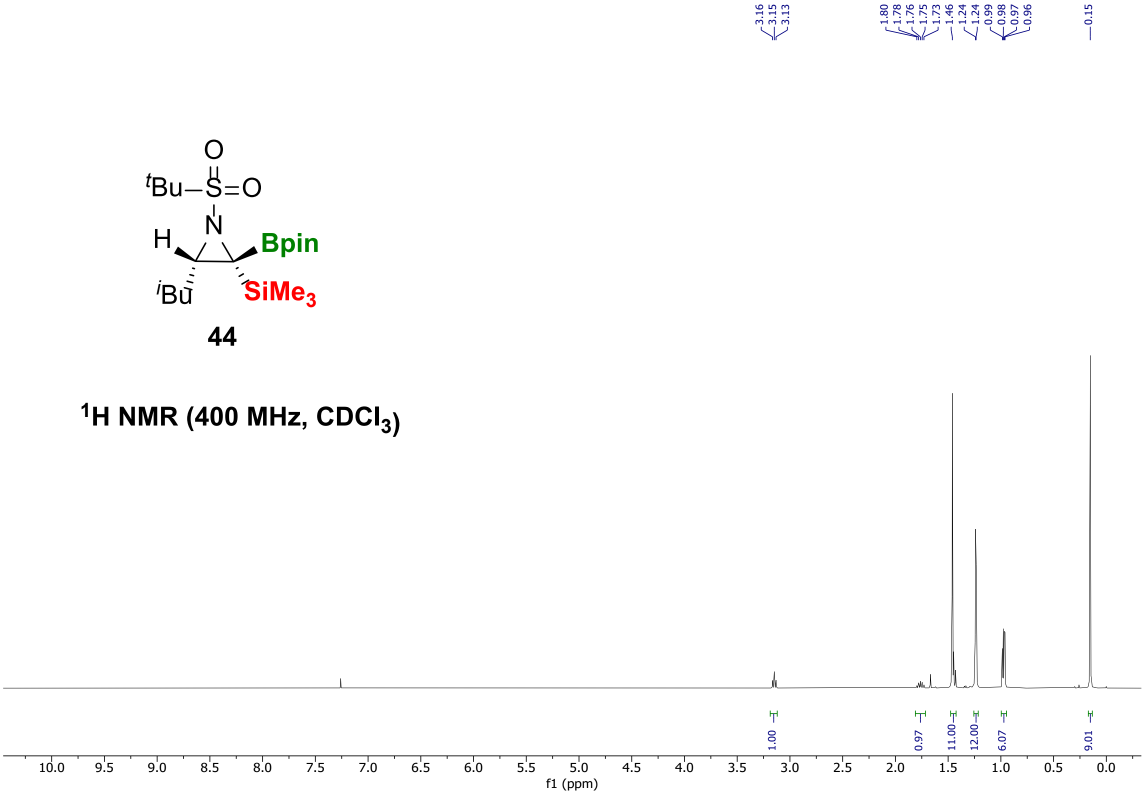

44

^{1}H NMR (400 MHz, CDCl$_3$)

44

^{13}C NMR (100 MHz, $CDCl_3$)

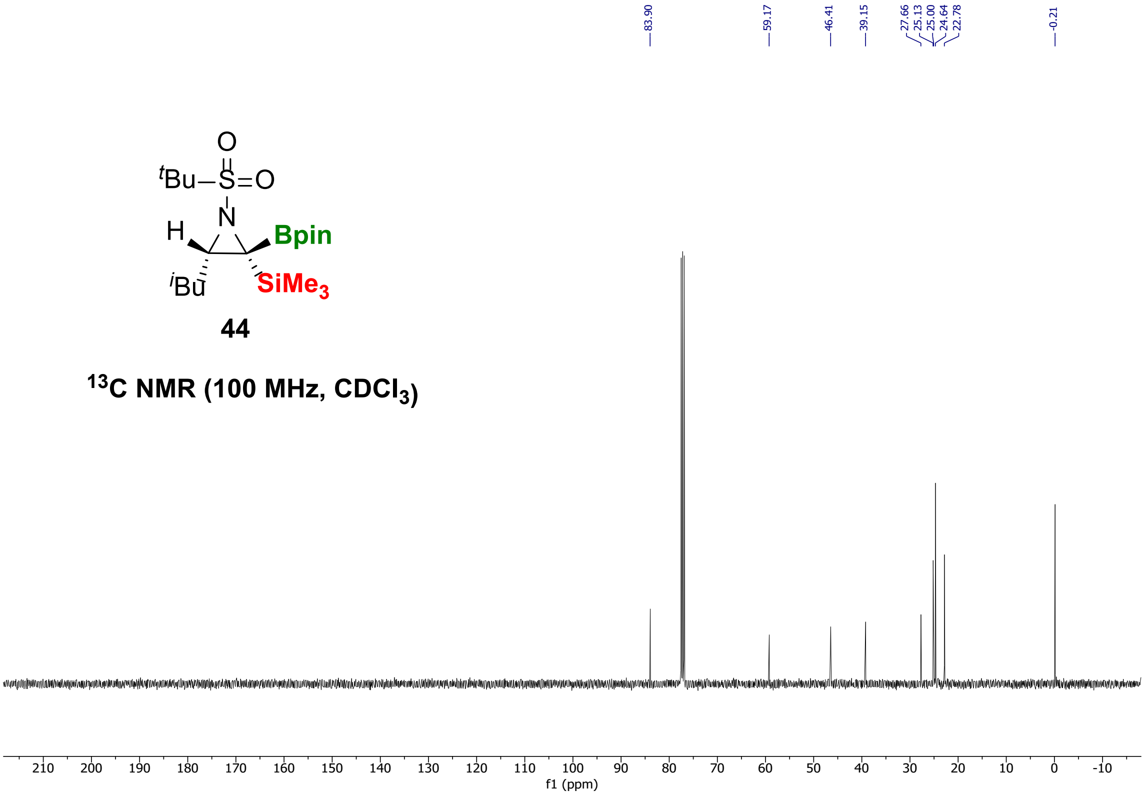

44

^{11}B NMR (129 MHz, $CDCl_3$)

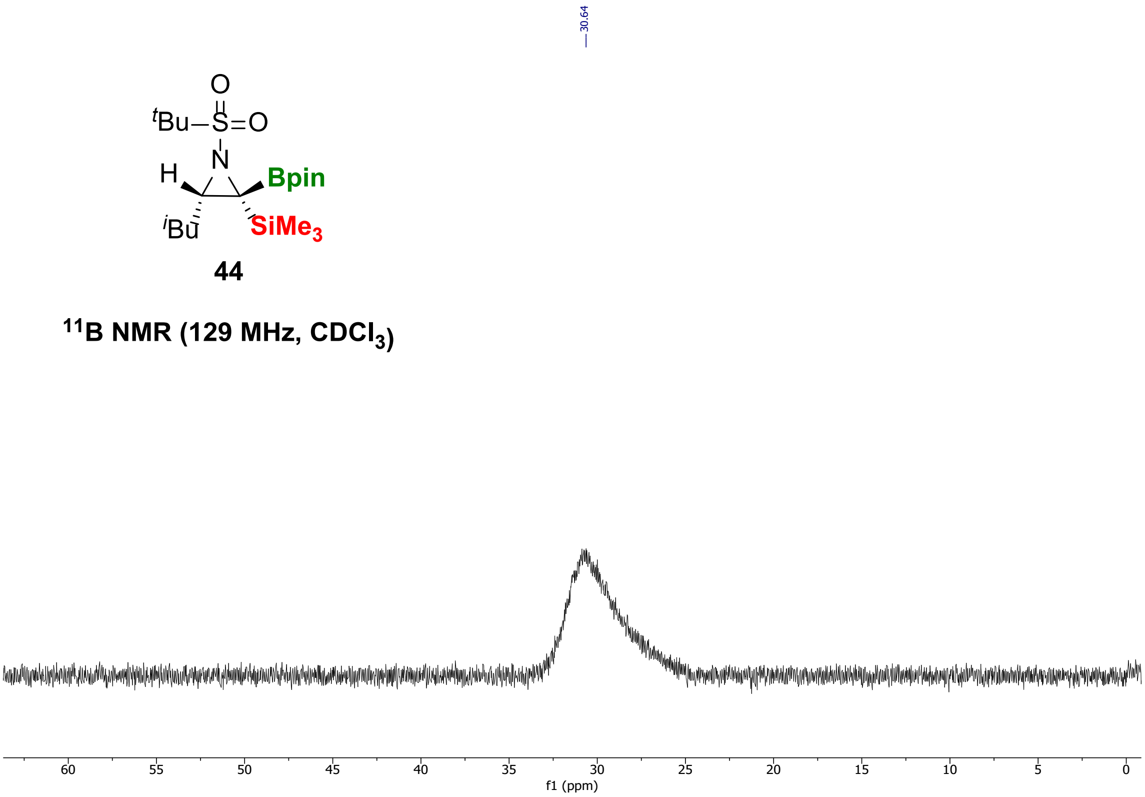

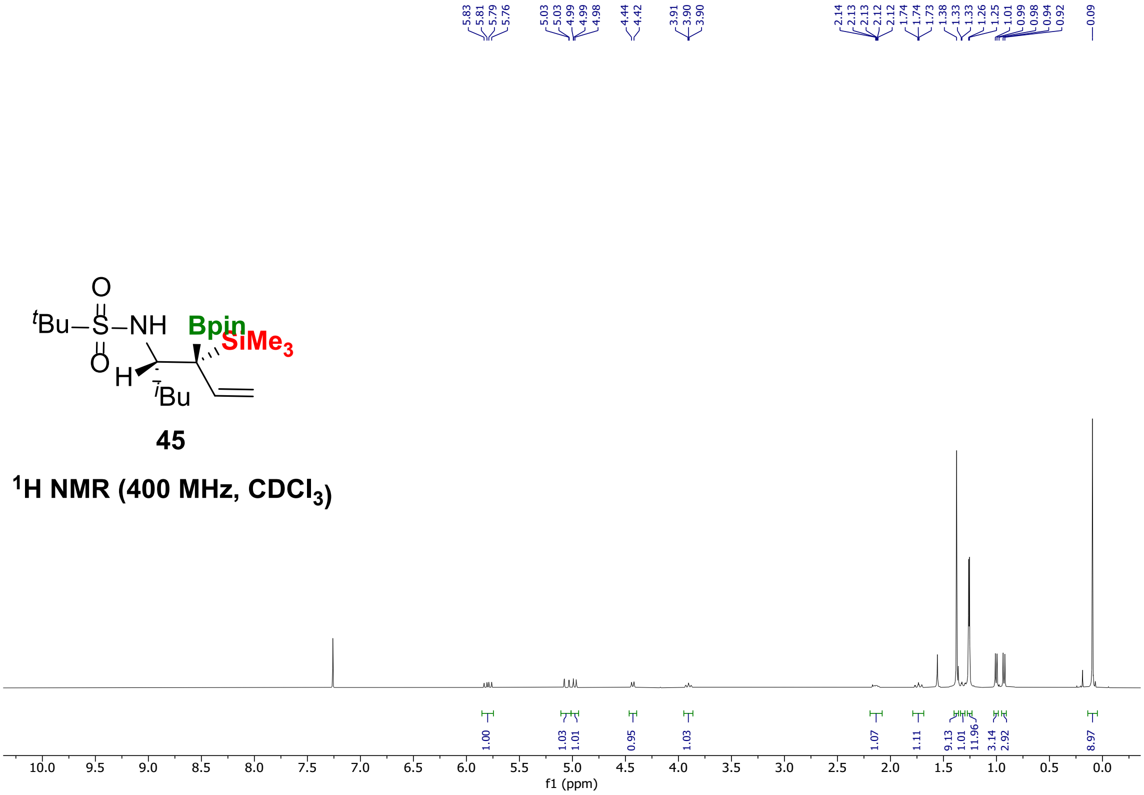

45

^{1}H NMR (400 MHz, $CDCl_3$)

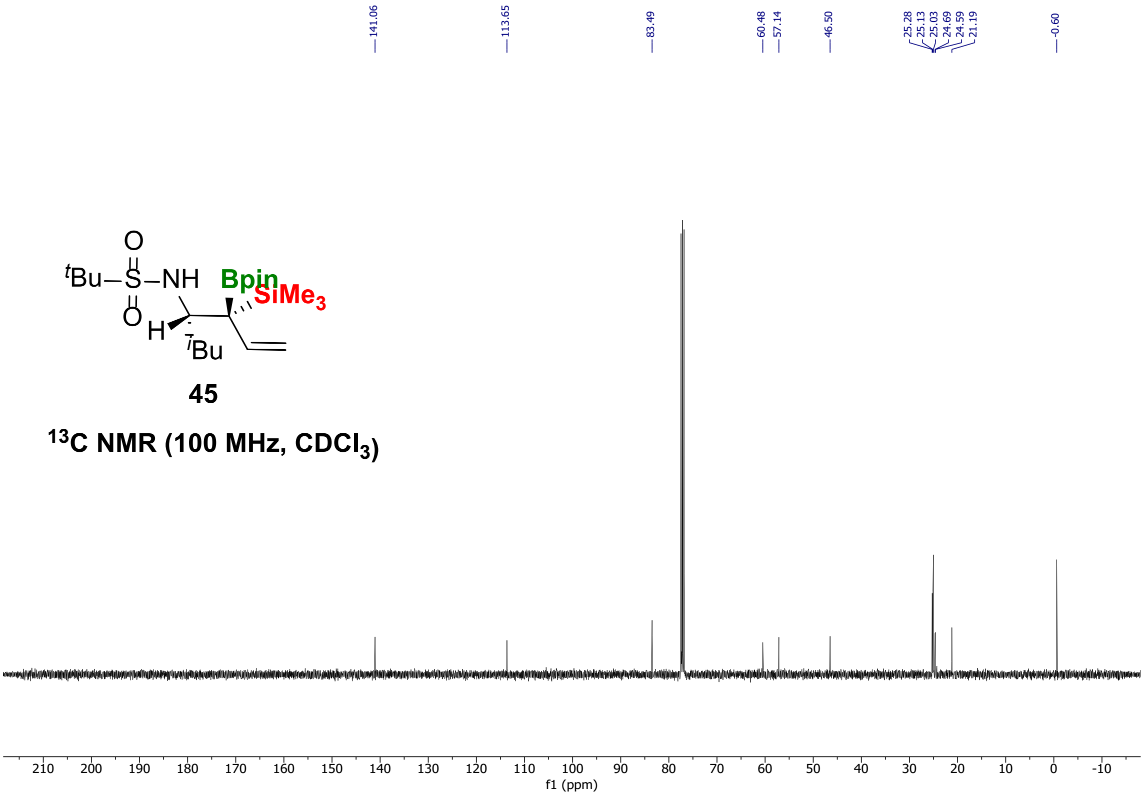

45

^{13}C NMR (100 MHz, $CDCl_3$)

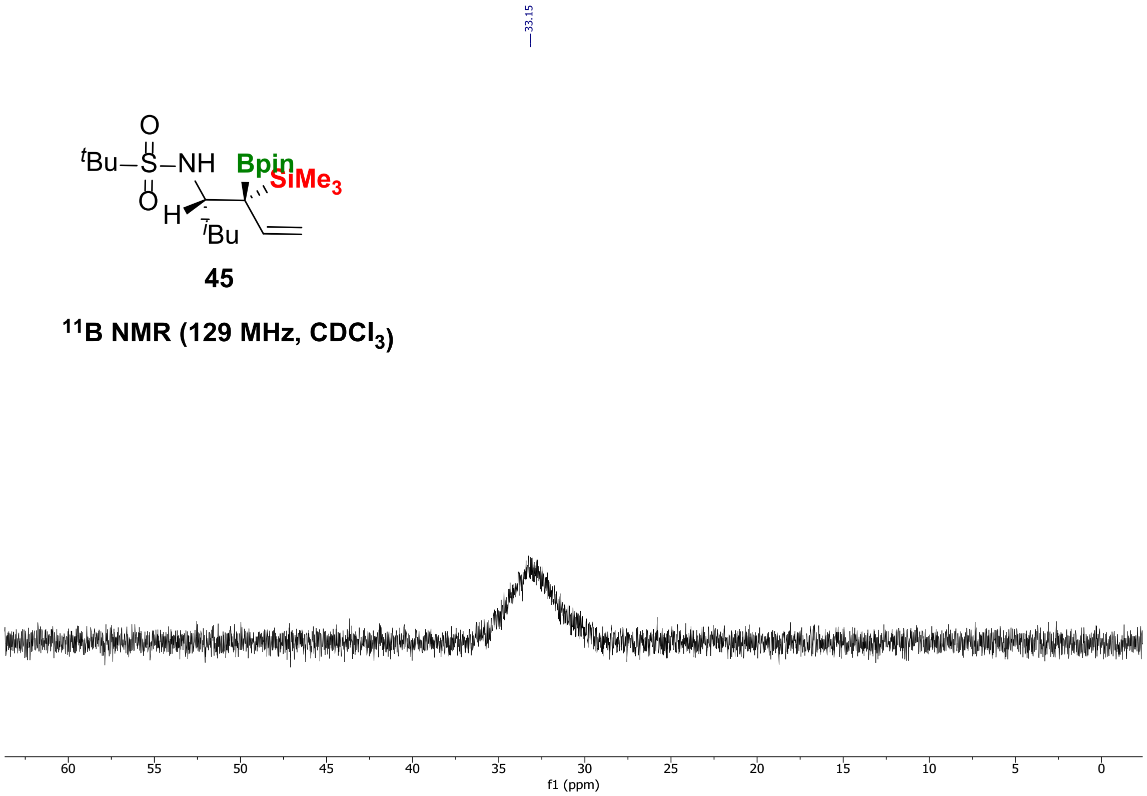

45

^{11}B NMR (129 MHz, CDCl$_3$)

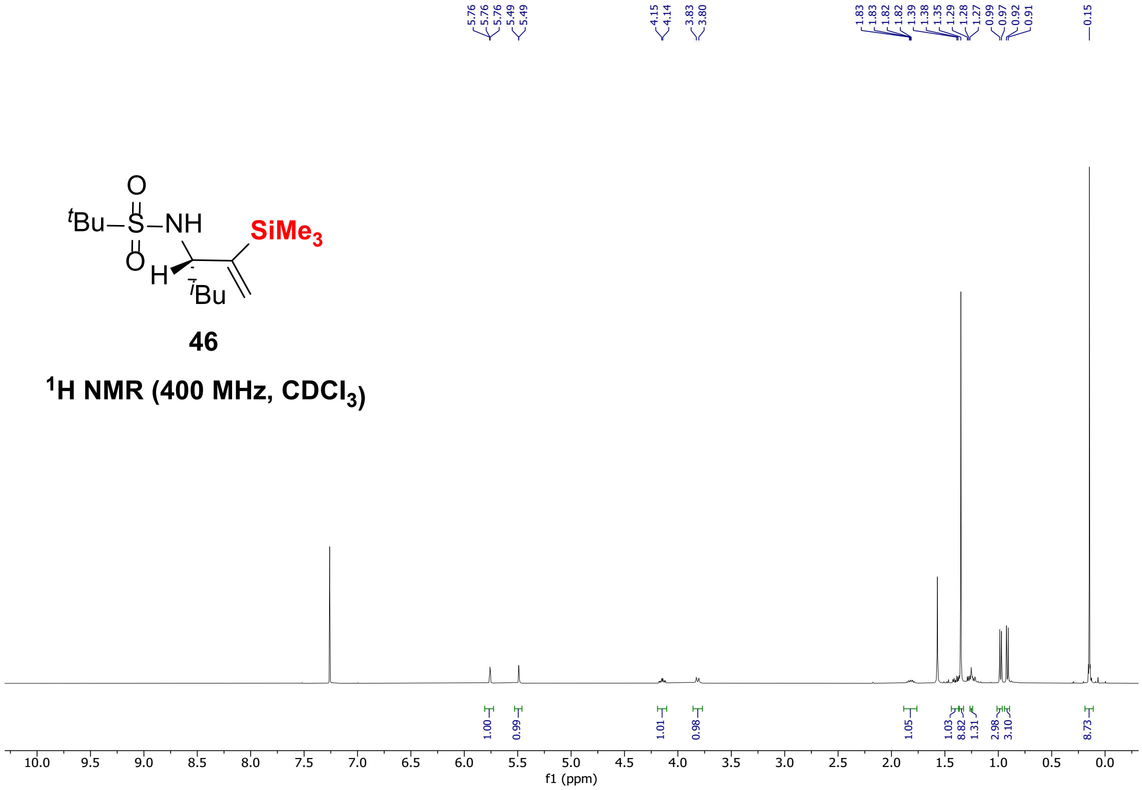

46

^{1}H NMR (400 MHz, CDCl$_3$)

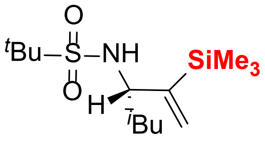

46

^{13}C NMR (100 MHz, CDCl$_3$)

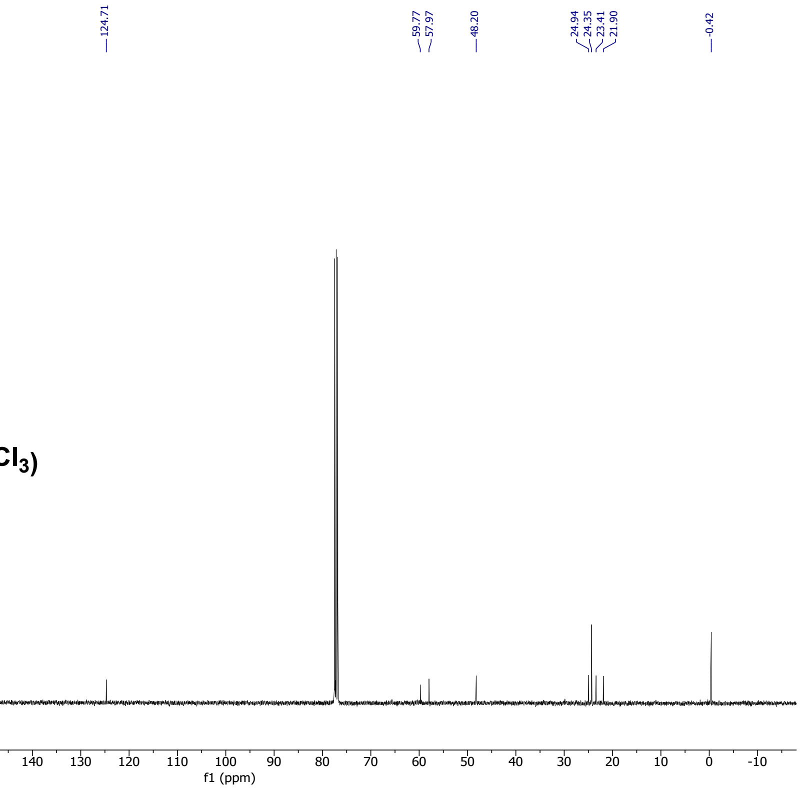

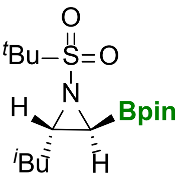

47

^{1}H NMR (400 MHz, CDCl$_3$)

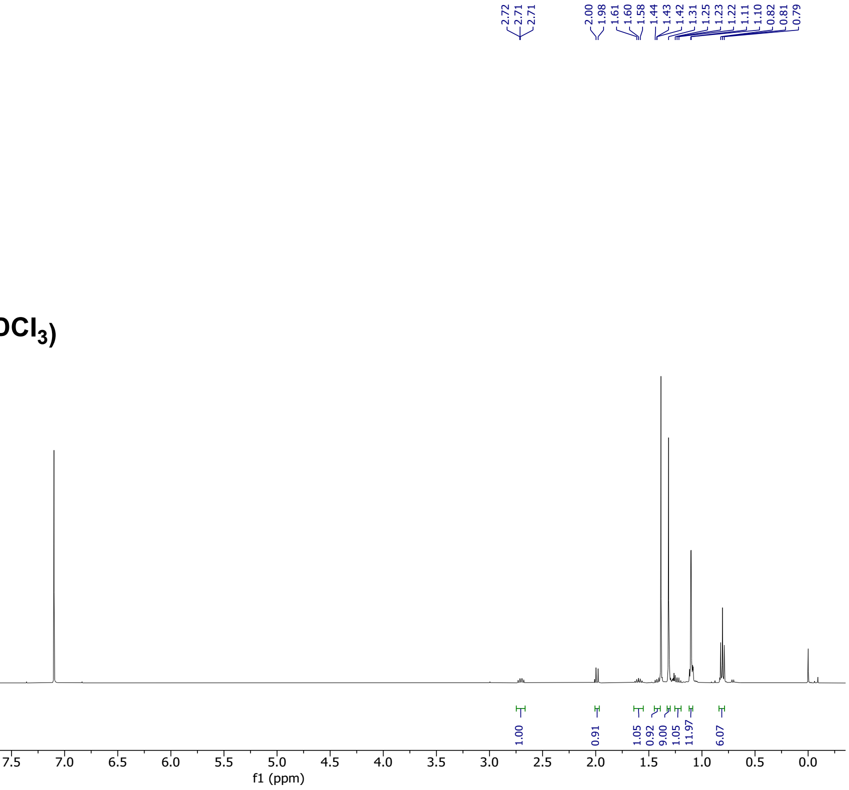

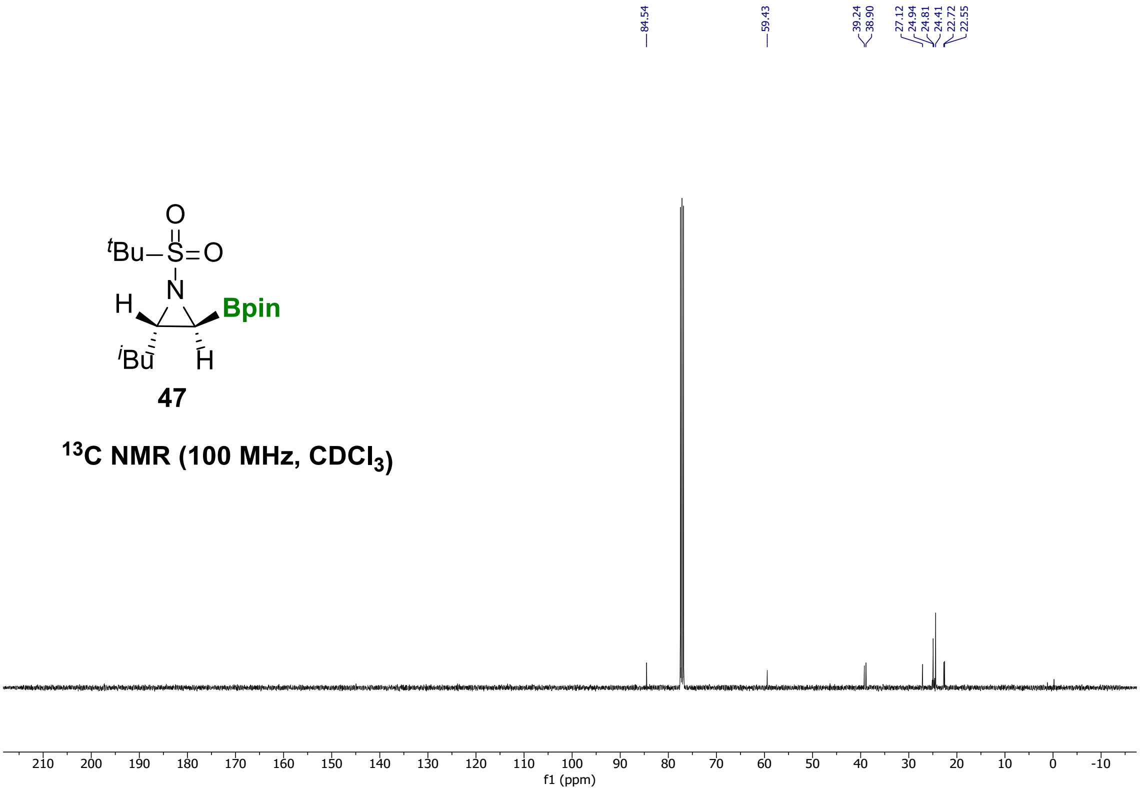
84.54
59.43
39.24
38.90
27.12
24.94
24.81
24.41
22.72
22.55
O
ᵗBu–S=O
N
H
Bpin
ⁱBu
H
47
13C NMR (100 MHz, CDCl3)
210 200 190 180 170 160 150 140 130 120 110 100 90 80 70 60 50 40 30 20 10 0 -10
f1 (ppm)

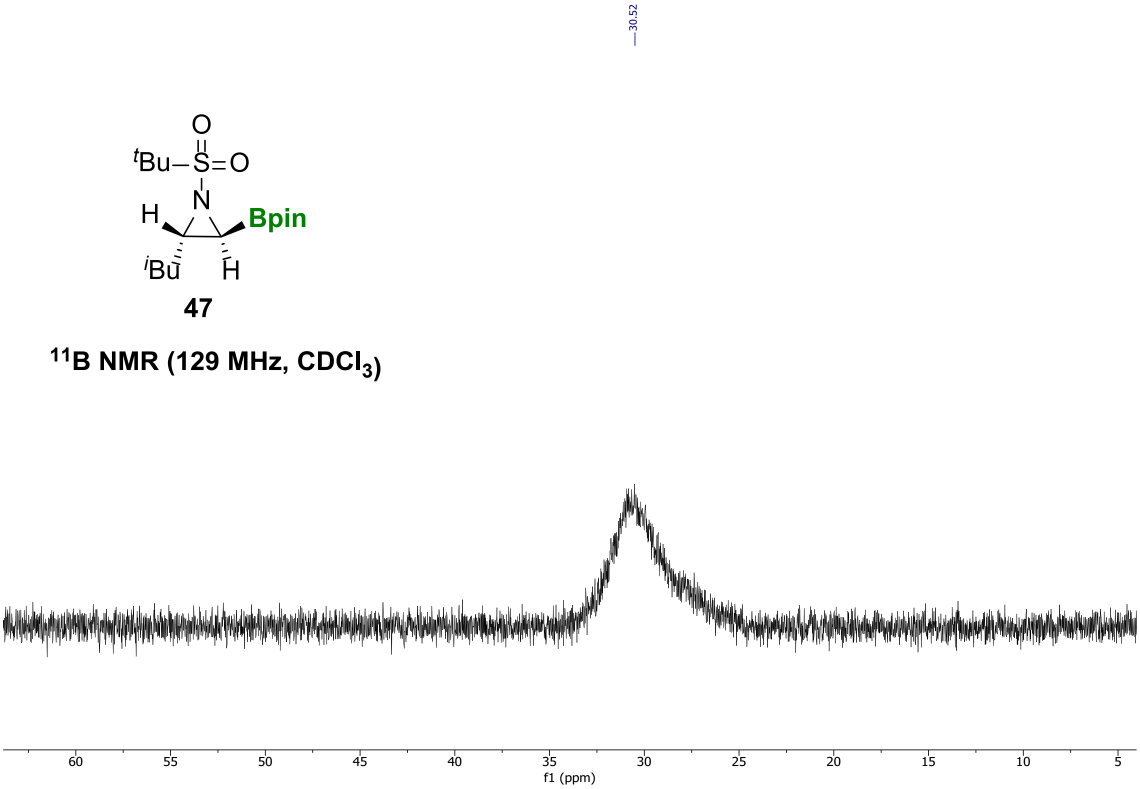
30.52
O
ᵗBu–S=O
N
H
Bpin
ⁱBu
H
47
11B NMR (129 MHz, CDCl3)
60 55 50 45 40 35 30 25 20 15 10 5
f1 (ppm)

(S_S*,2*R**,3*S**)-**48**

^{1}H NMR (400 MHz, $CDCl_3$)

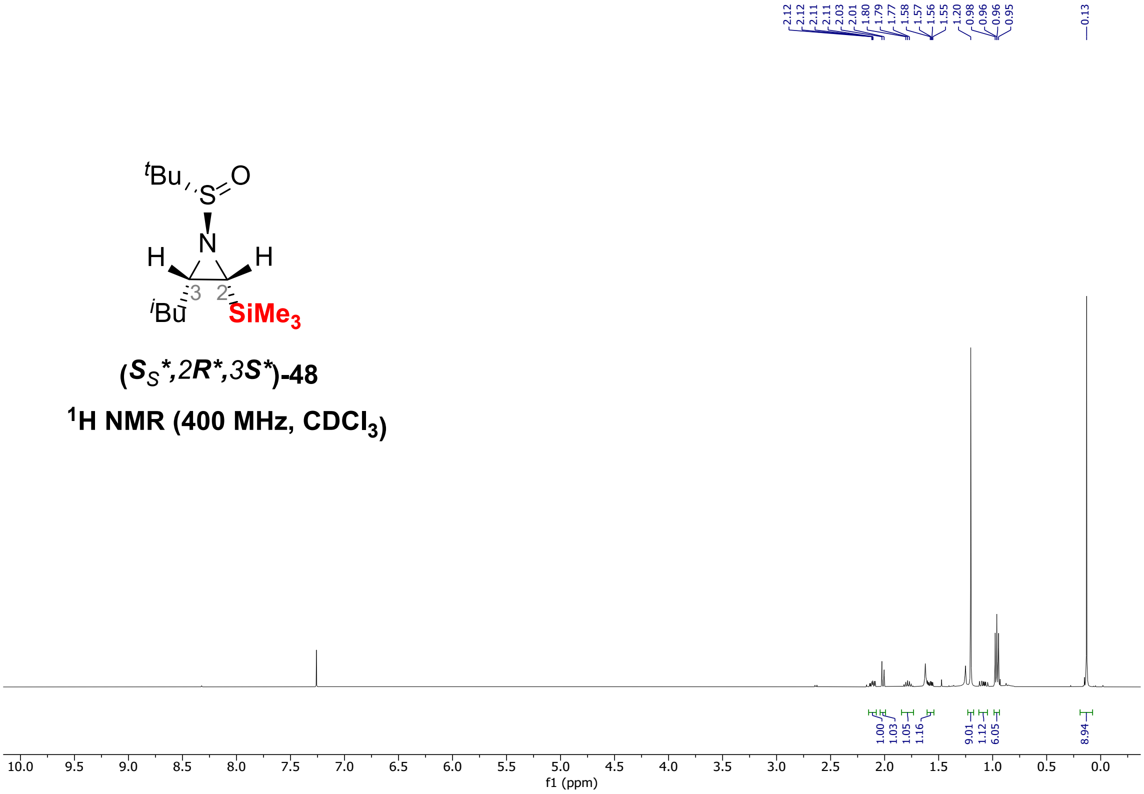

(S_S*,2*R**,3*S**)-**48**

^{13}C NMR (100 MHz, $CDCl_3$)

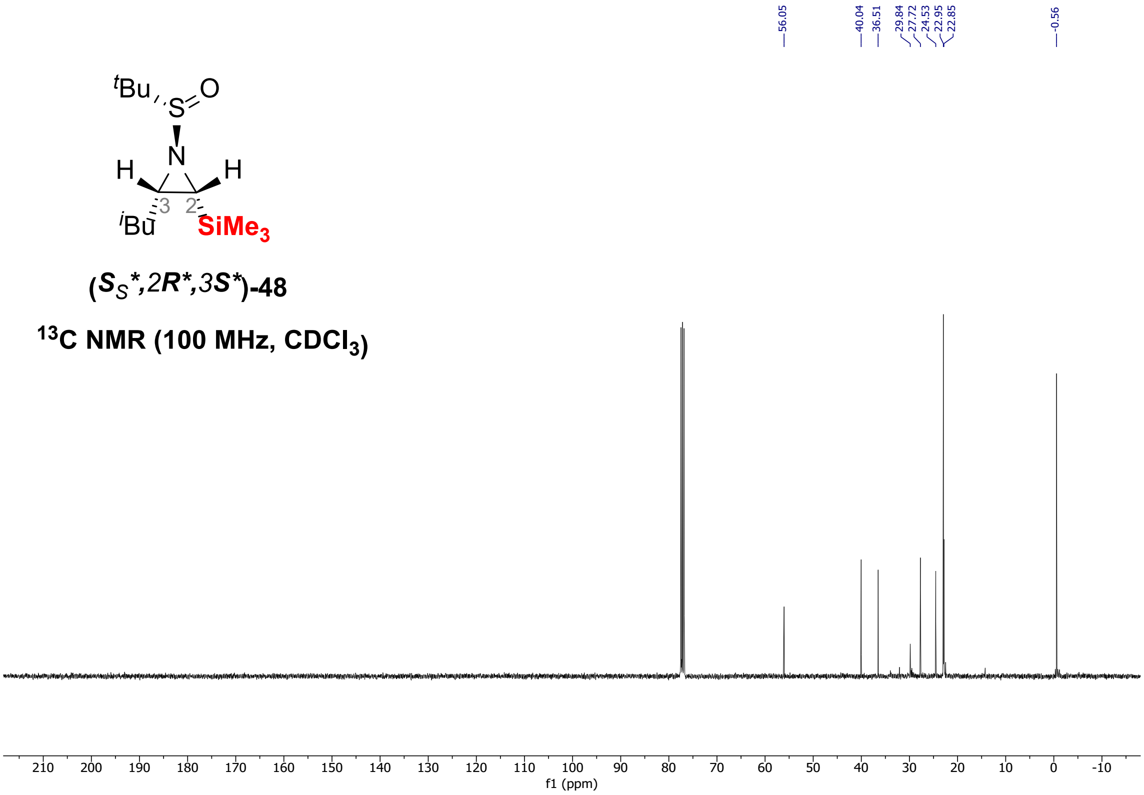

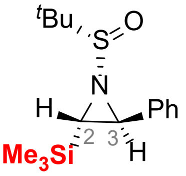

(S_S*,2S*,3S*)-49

^{1}H NMR (400 MHz, $CDCl_3$)

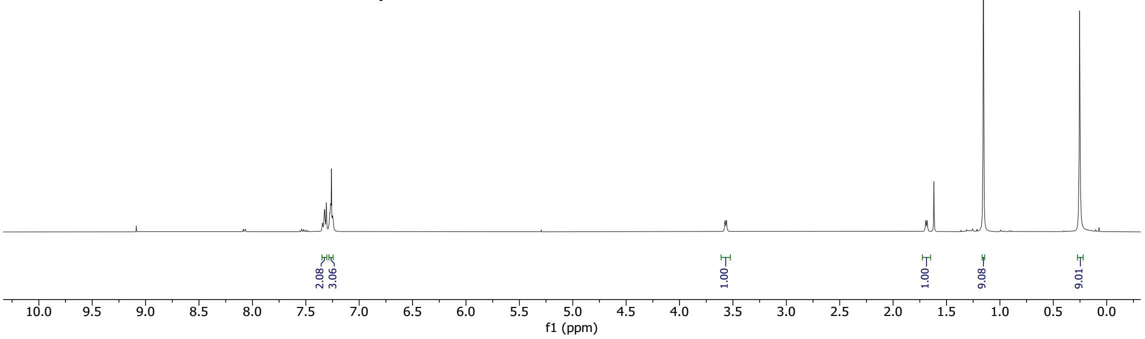

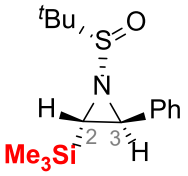

(S_S*,2S*,3S*)-49

^{13}C NMR (100 MHz, $CDCl_3$)

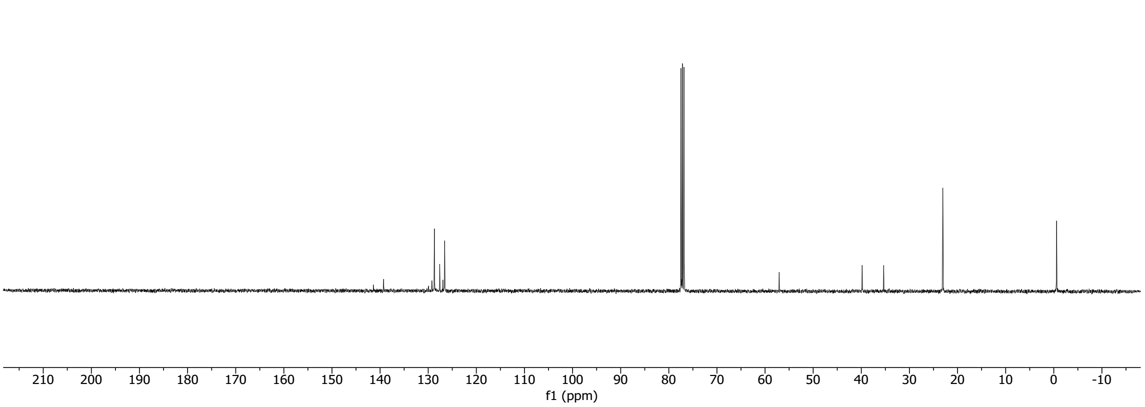

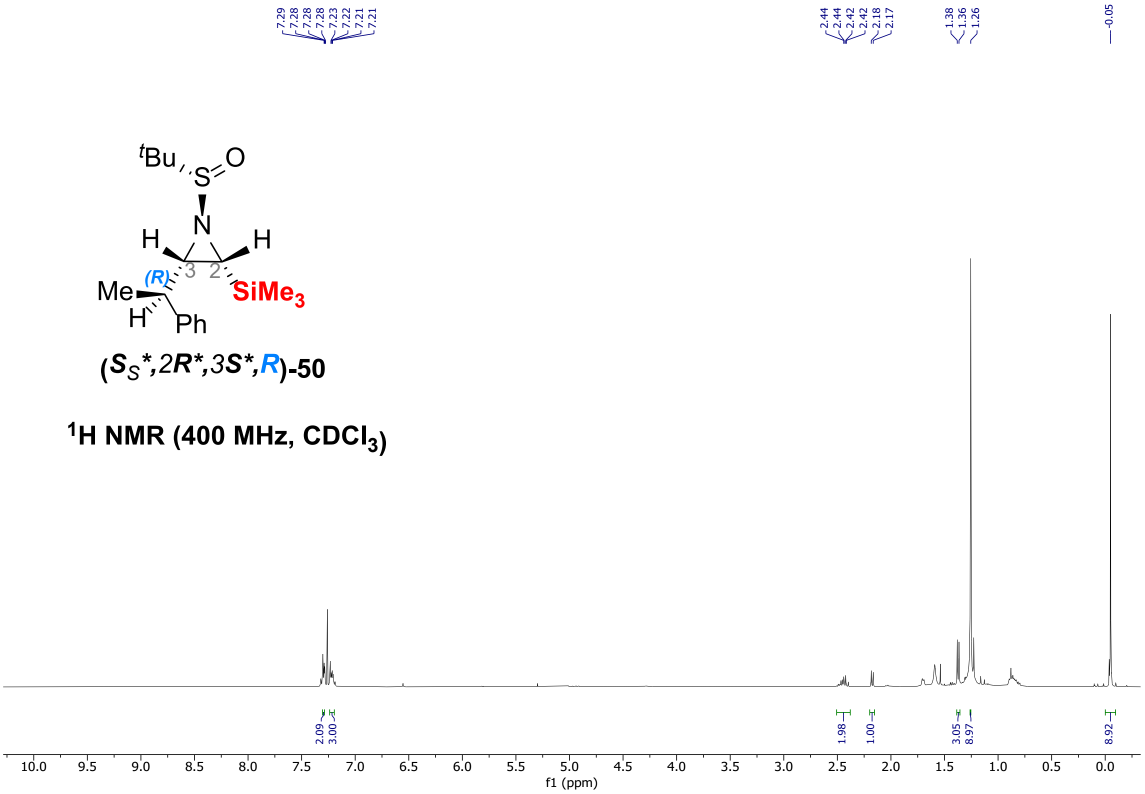

(S_S*,2*R,3*S**,*R*)-50**

^{1}H NMR (400 MHz, CDCl$_3$)

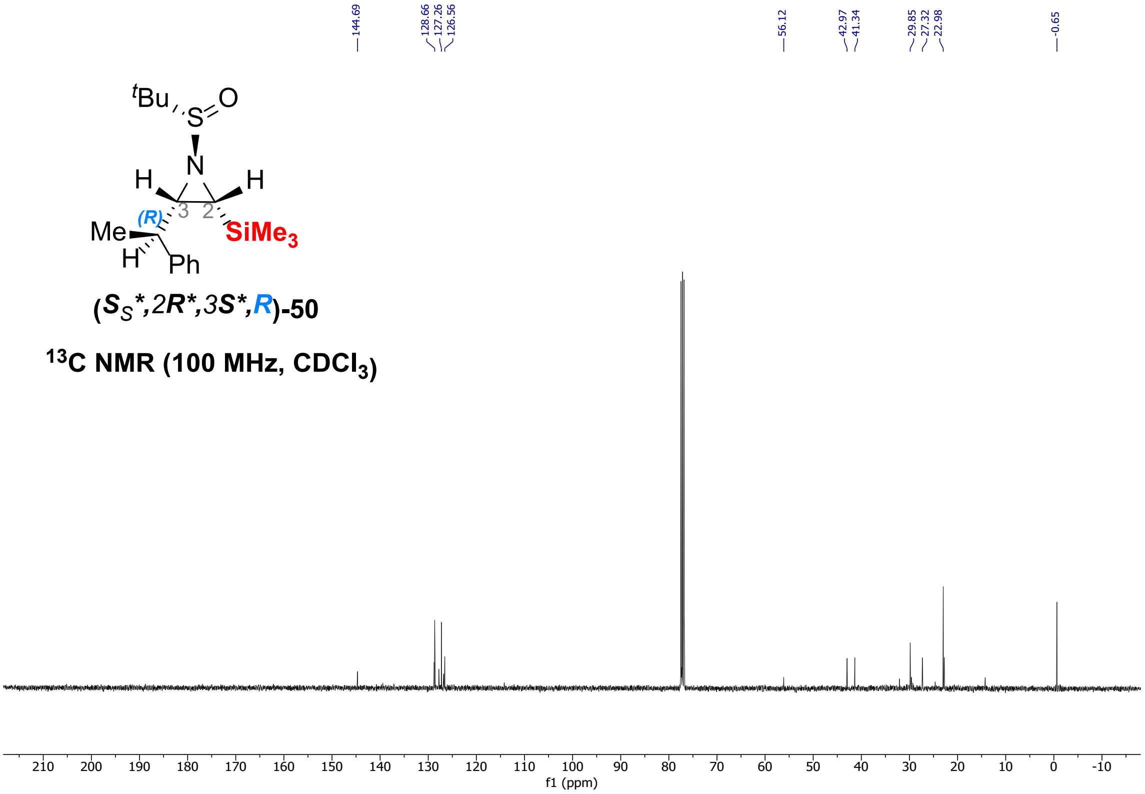

(S_S*,2*R,3*S**,*R*)-50**

^{13}C NMR (100 MHz, CDCl$_3$)

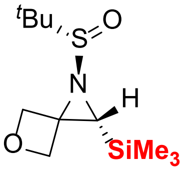

(S_S*,R*)-51

^{1}H NMR (400 MHz, CDCl$_3$)

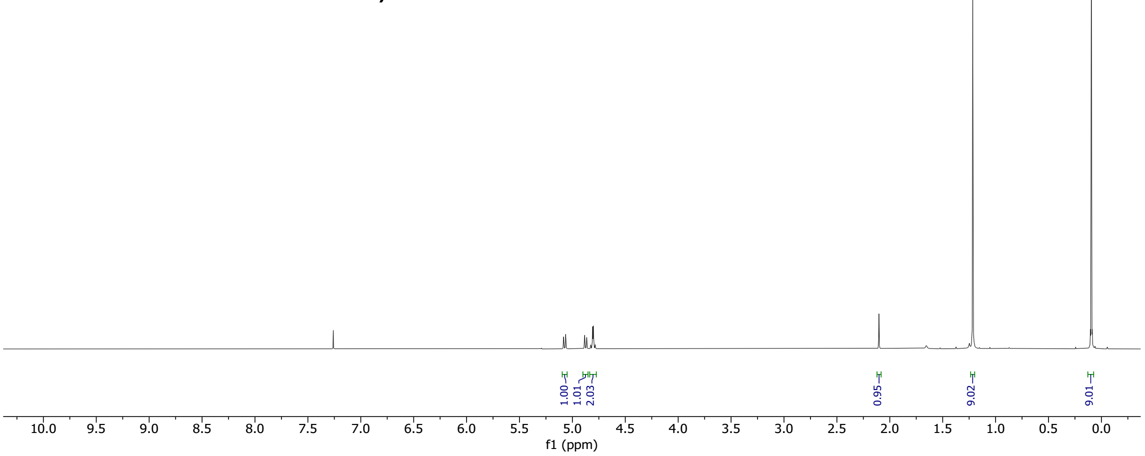

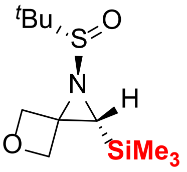

(S_S*,R*)-51

^{13}C NMR (100 MHz, CDCl$_3$)

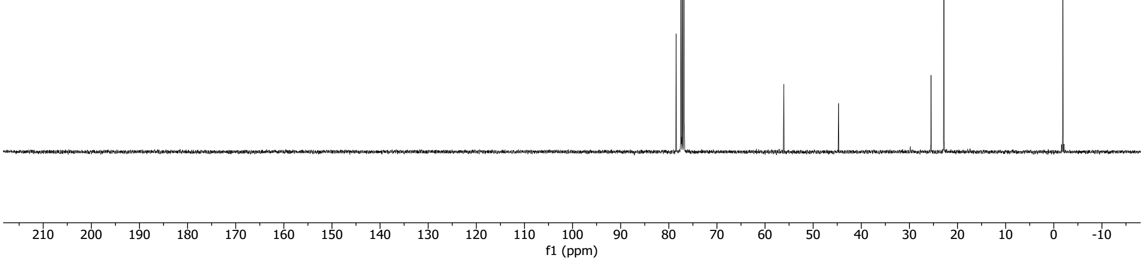

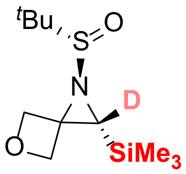

(S_S*,R*)-52

^{1}H NMR (400 MHz, $CDCl_3$)

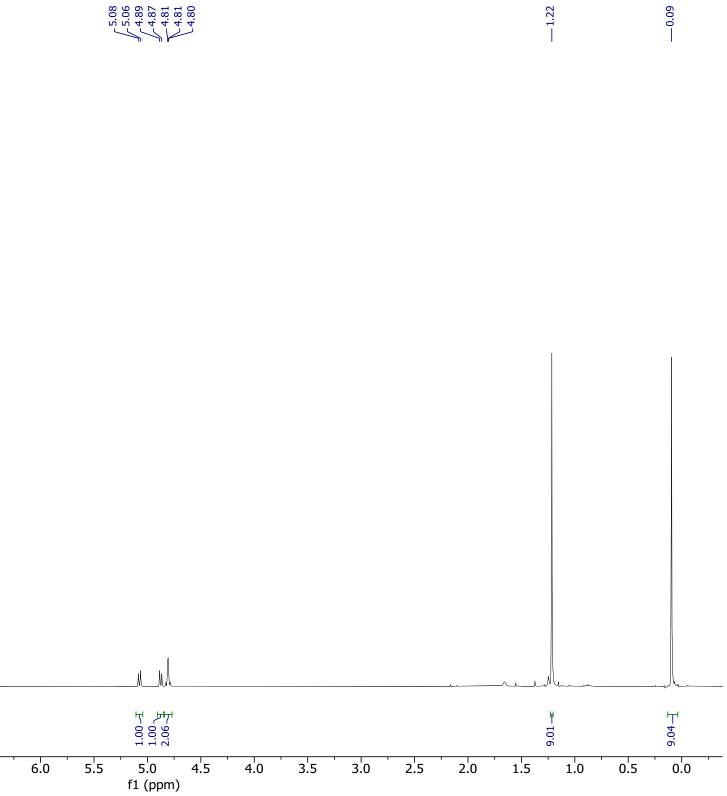

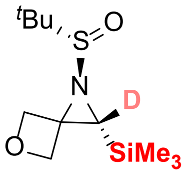

(S_S*,R*)-52

^{13}C NMR (100 MHz, $CDCl_3$)

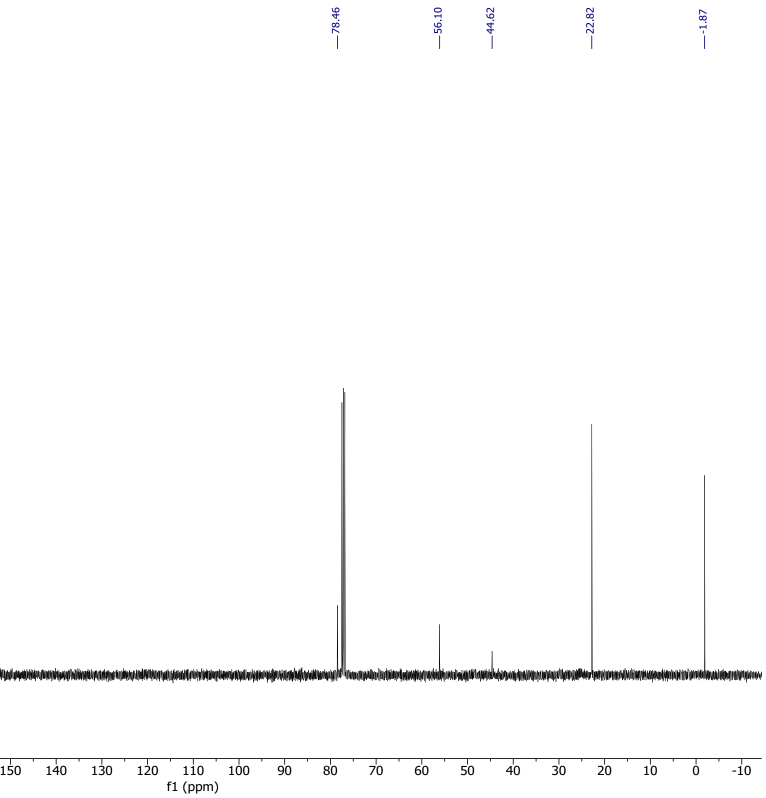

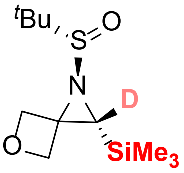

(S_S*,R*)-52

^{2}H NMR (61.285 MHz, $CDCl_3$)

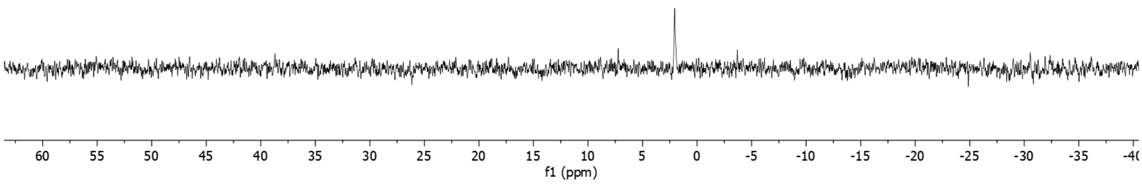

HLPC analysis of chiral aziridines

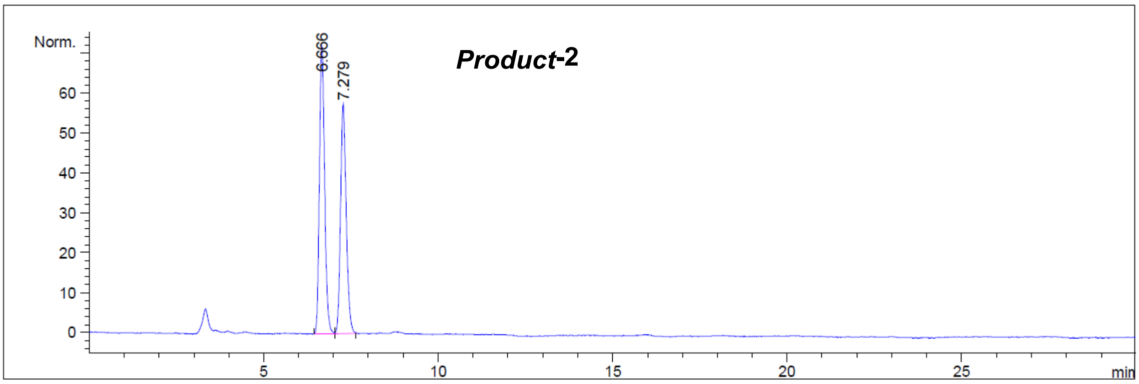

Peak #	RetTime [min]	Type	Width [min]	Area [mAU*s]	Height [mAU]	Area %
1	6.666	BB	0.1570	746.66931	72.01035	53.1206
2	7.279	BB	0.1757	658.94098	57.48581	46.8794

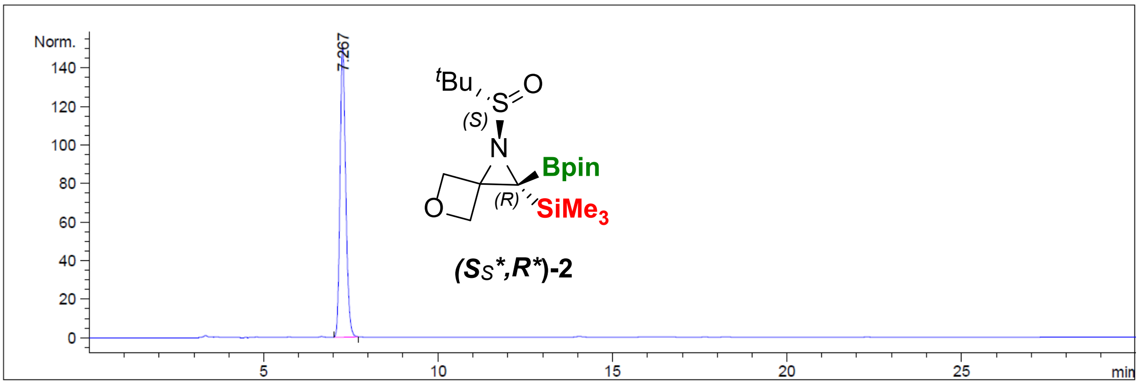

Peak #	RetTime [min]	Type	Width [min]	Area [mAU*s]	Height [mAU]	Area %
1	7.267	BB	0.1759	1728.50720	150.60753	100.0000

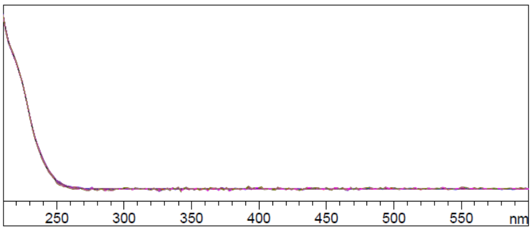

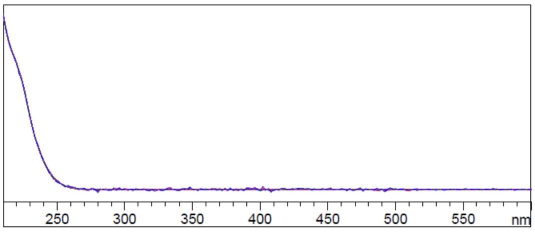

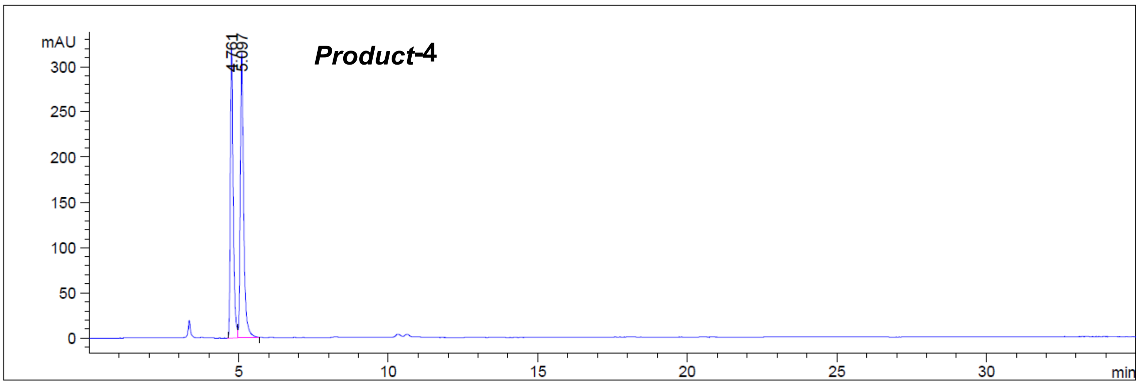

Peak #	RetTime [min]	Type	Width [min]	Area [mAU*s]	Height [mAU]	Area %
1	4.761	BV	0.0994	2132.46875	321.28055	46.2915
2	5.097	VB	0.1173	2474.14453	316.27698	53.7085

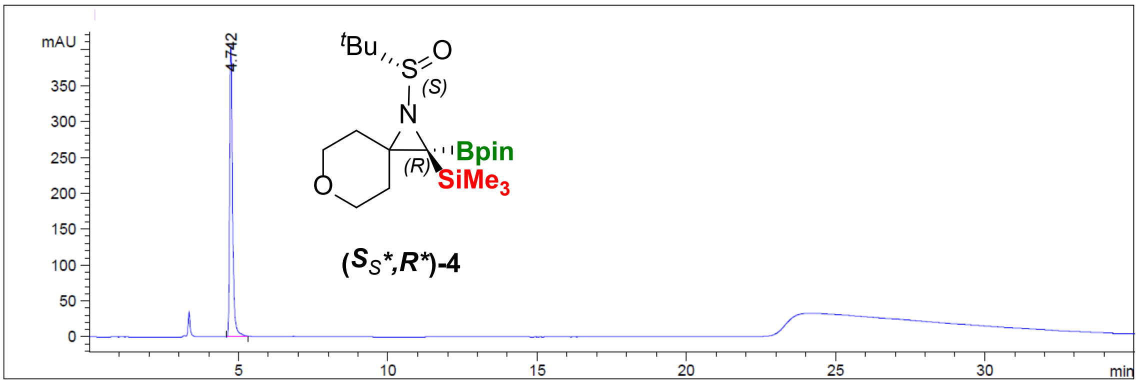

Peak #	RetTime [min]	Type	Width [min]	Area [mAU*s]	Height [mAU]	Area %
1	4.742	BB	0.1036	2756.10718	403.69949	100.0000

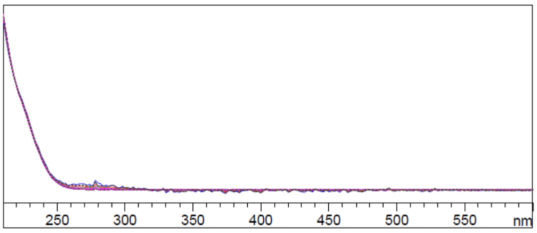

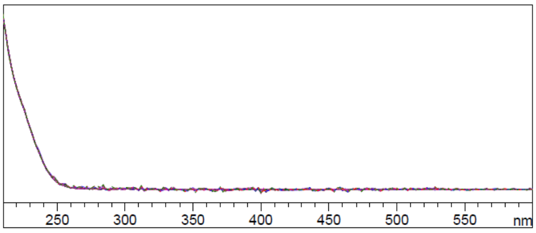

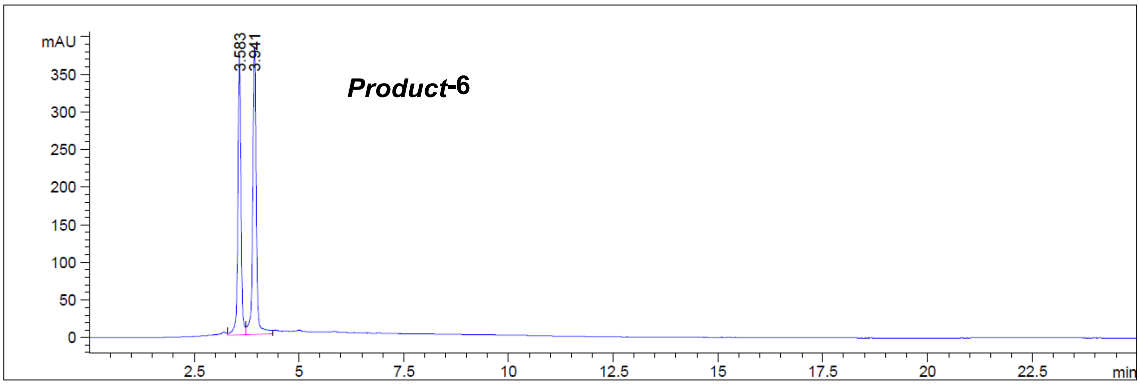

Peak #	RetTime [min]	Type	Width [min]	Area [mAU*s]	Height [mAU]	Area %
1	3.583	VV	0.0782	1927.00757	373.58936	45.9965
2	3.941	VB	0.0889	2262.46094	383.08841	54.0035

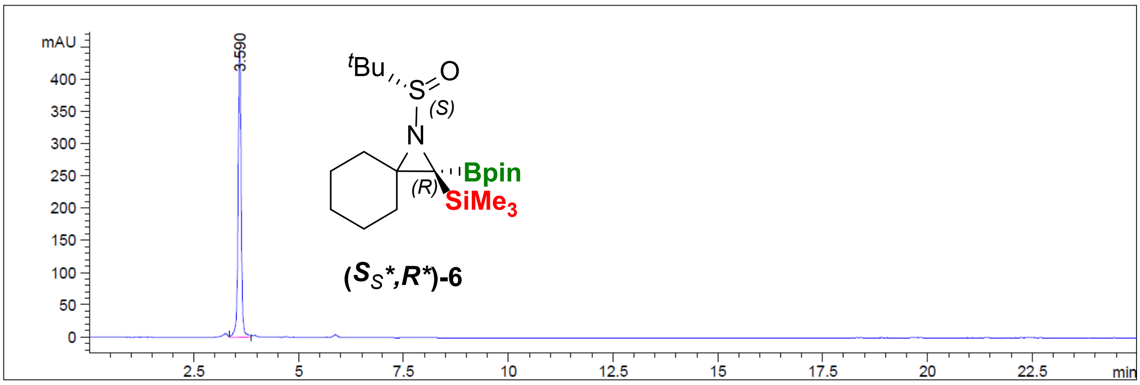

Peak #	RetTime [min]	Type	Width [min]	Area [mAU*s]	Height [mAU]	Area %
1	3.590	VB	0.0774	2297.82397	451.37506	100.0000

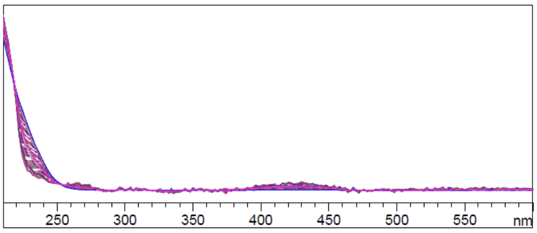

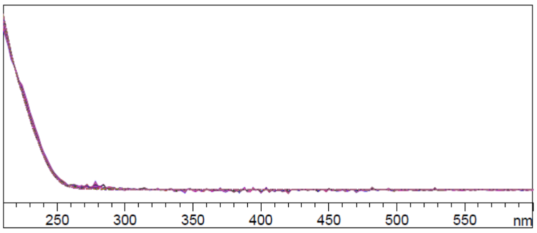

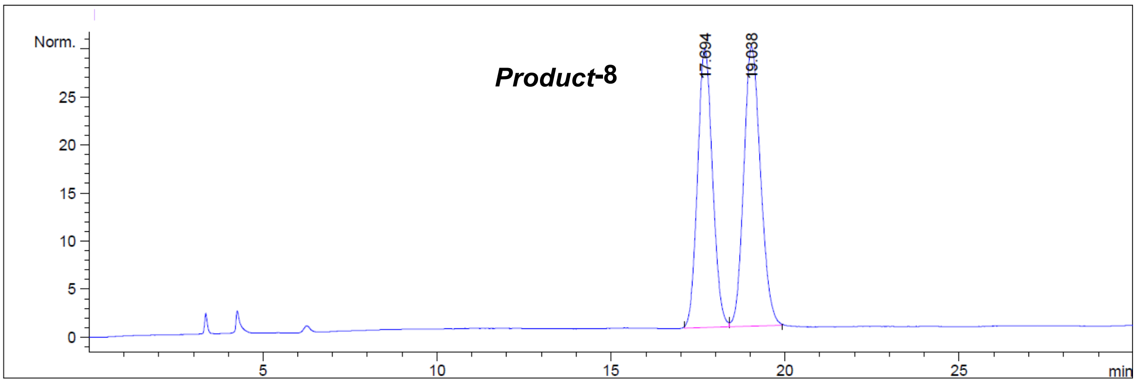

Peak #	RetTime [min]	Type	Width [min]	Area [mAU*s]	Height [mAU]	Area %
1	17.694	BV	0.4696	867.72131	28.95976	47.0359
2	19.038	VB	0.5204	977.08575	29.05088	52.9641

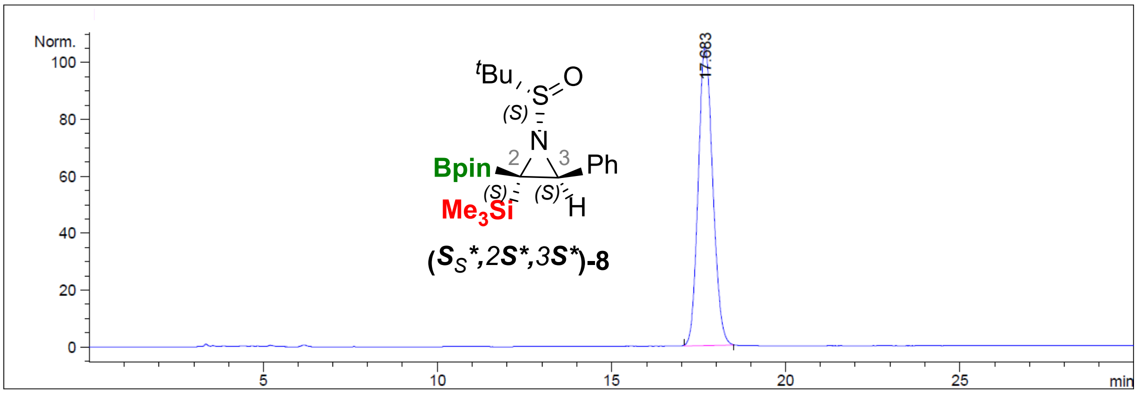

Peak #	RetTime [min]	Type	Width [min]	Area [mAU*s]	Height [mAU]	Area %
1	17.683	BB	0.4522	3061.33154	104.97874	100.0000

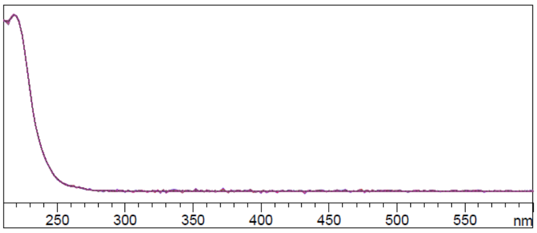

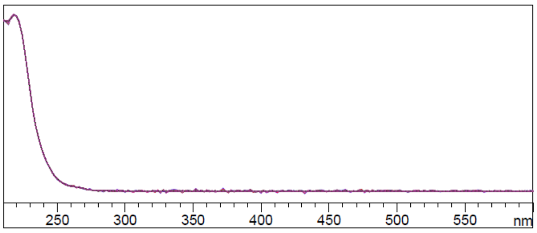

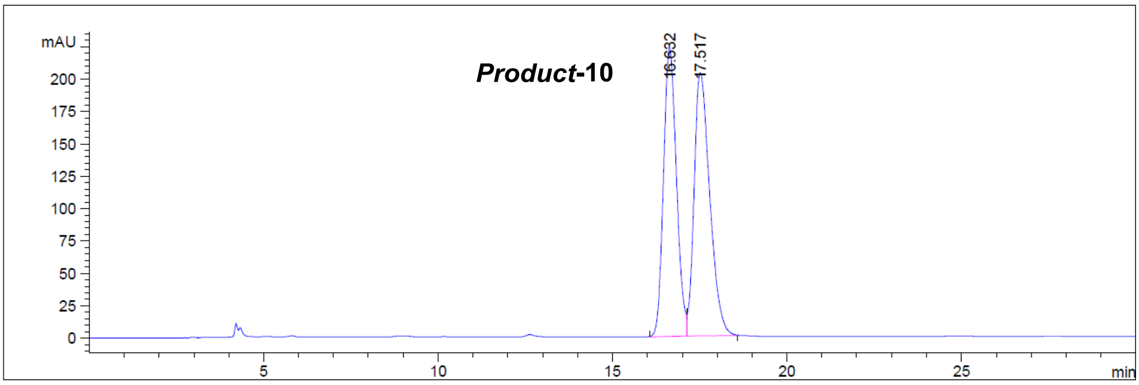

Peak #	RetTime [min]	Type	Width [min]	Area [mAU*s]	Height [mAU]	Area %
1	16.632	BV	0.3981	5754.78760	223.69461	47.5103
2	17.517	VB	0.4788	6357.92871	203.32726	52.4897

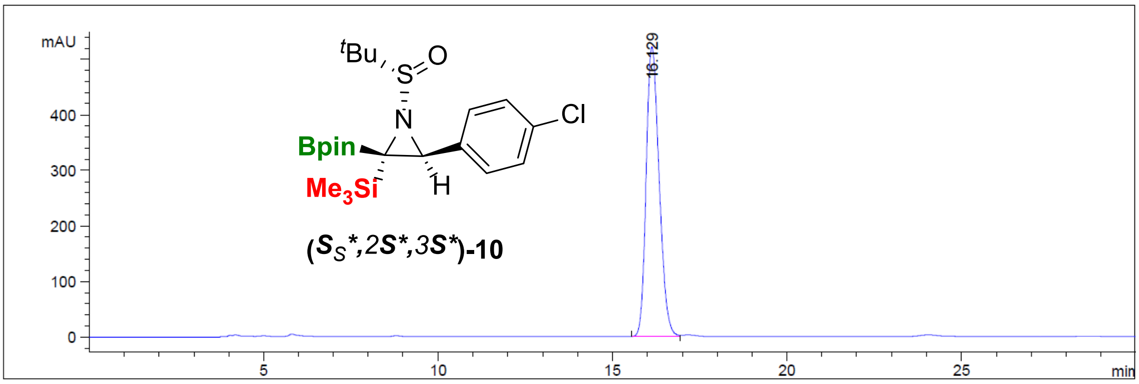

(S_S*,2S*,3S*)-**10**

Peak #	RetTime [min]	Type	Width [min]	Area [mAU*s]	Height [mAU]	Area %
1	16.129	BB	0.4024	1.35263e4	521.89777	100.0000

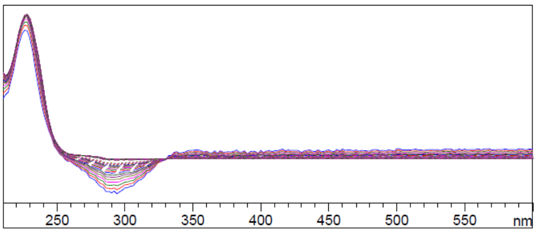

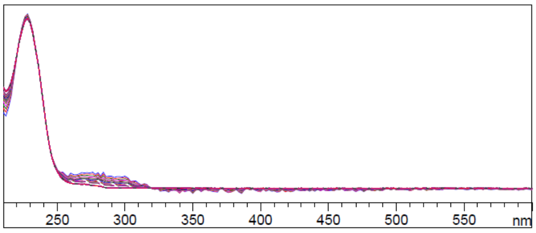

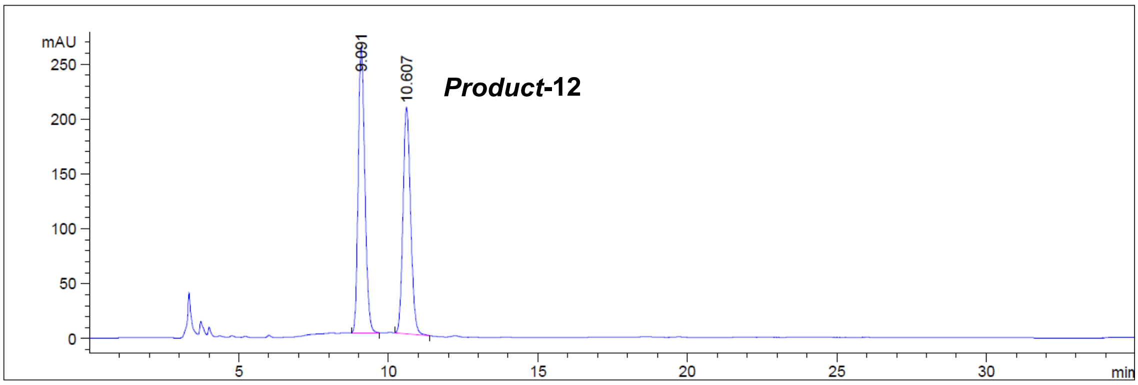

Peak #	RetTime [min]	Type	Width [min]	Area [mAU*s]	Height [mAU]	Area %
1	9.091	BB	0.2380	4020.44434	260.53870	52.3775
2	10.607	BB	0.2744	3655.45190	206.64877	47.6225

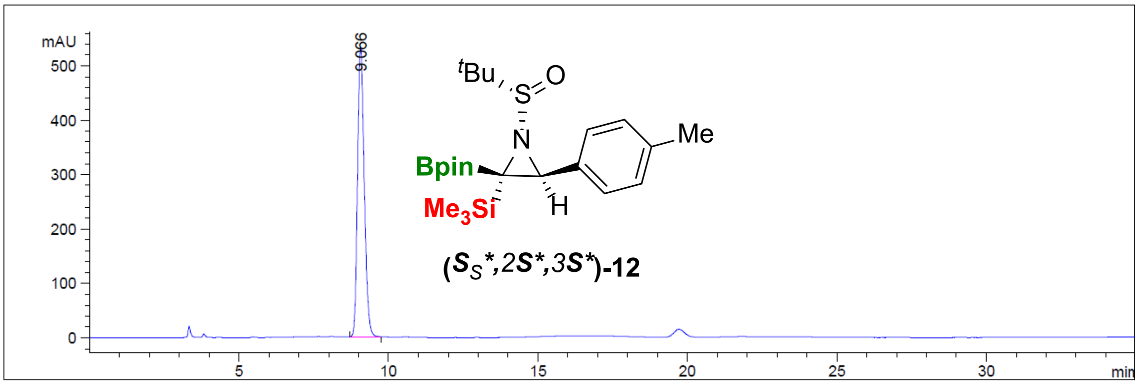

Peak #	RetTime [min]	Type	Width [min]	Area [mAU*s]	Height [mAU]	Area %
1	9.066	BB	0.2400	8327.95410	533.76080	100.0000

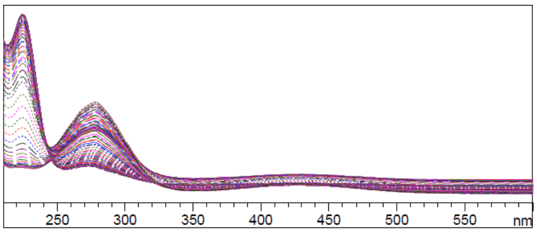

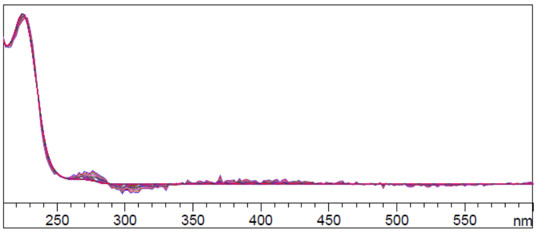

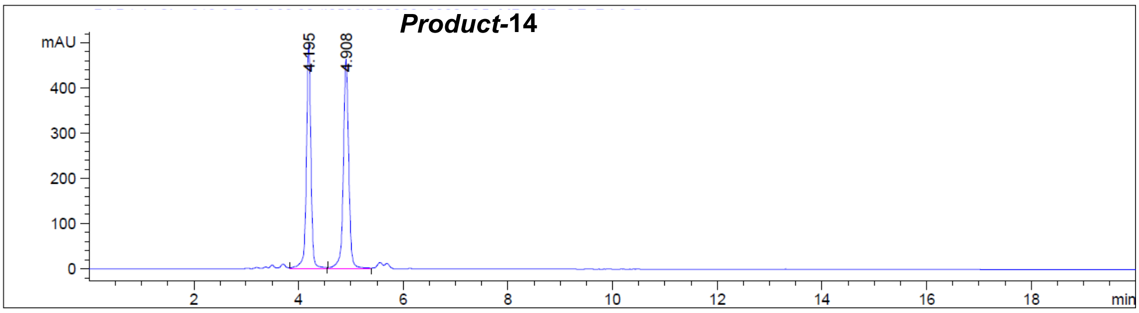

Peak #	RetTime [min]	Type	Width [min]	Area [mAU*s]	Height [mAU]	Area %
1	4.195	VB	0.0932	3041.57251	498.12170	47.5431
2	4.908	BB	0.1104	3355.93823	463.97791	52.4569

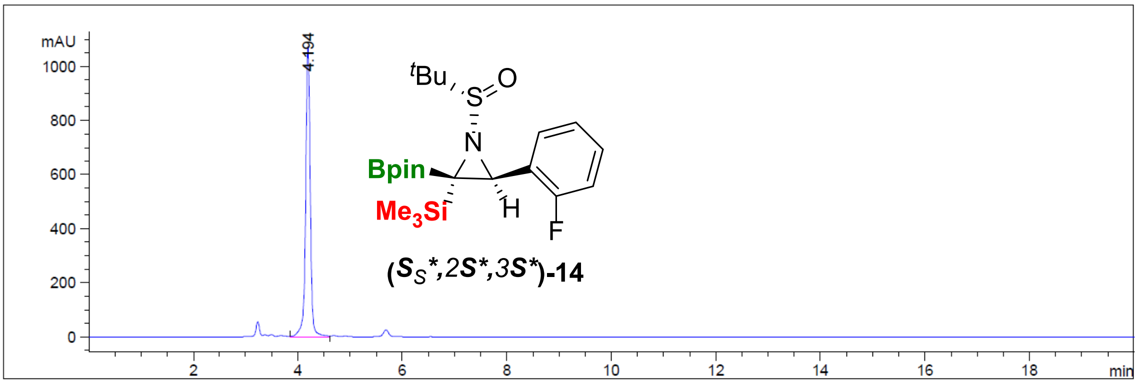

Peak #	RetTime [min]	Type	Width [min]	Area [mAU*s]	Height [mAU]	Area %
1	4.194	VV	0.0920	6643.65918	1076.30273	100.0000

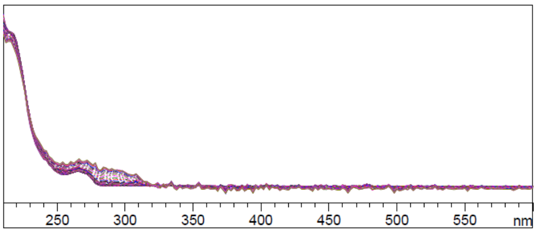

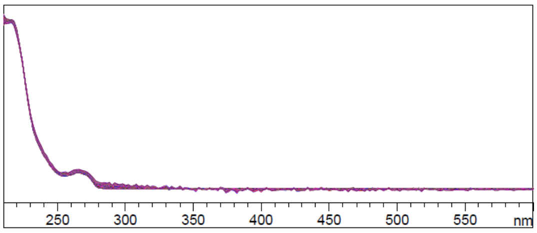

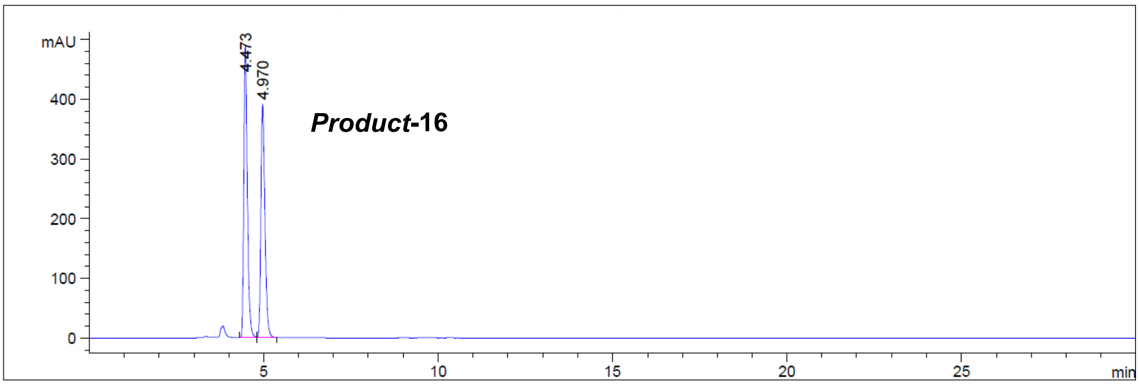

Peak #	RetTime [min]	Type	Width [min]	Area [mAU*s]	Height [mAU]	Area %
1	4.473	BV	0.1061	3425.95850	486.89554	52.6975
2	4.970	VB	0.1176	3075.22485	391.77634	47.3025

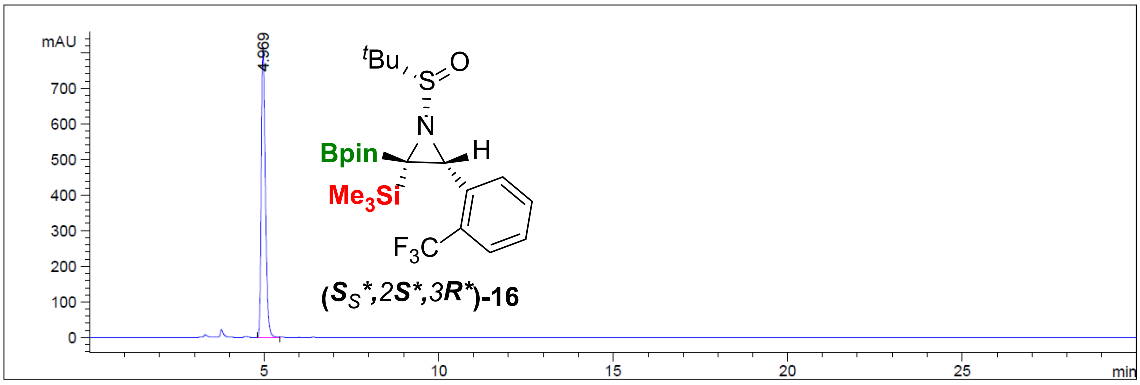

Peak #	RetTime [min]	Type	Width [min]	Area [mAU*s]	Height [mAU]	Area %
1	4.969	BB	0.1187	6487.87549	816.39484	100.0000

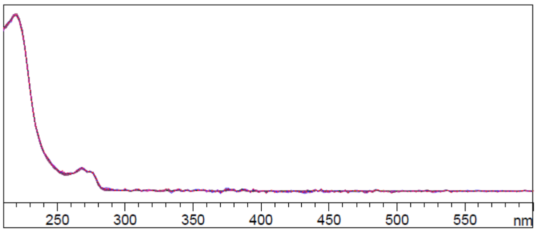

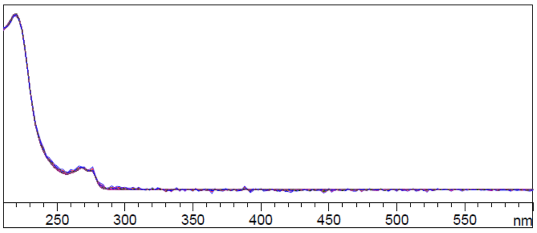

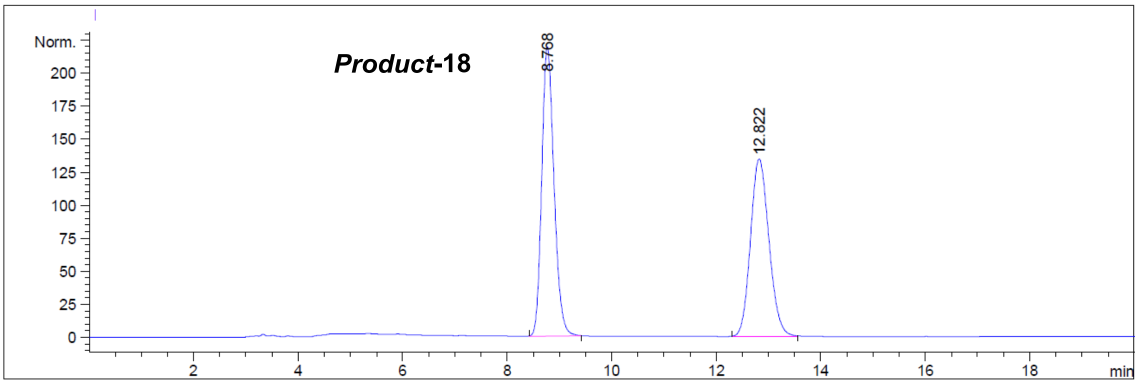

Peak #	RetTime [min]	Type	Width [min]	Area [mAU*s]	Height [mAU]	Area %
1	8.768	BB	0.2506	3577.17114	218.93332	52.0663
2	12.822	BB	0.3800	3293.24976	134.35019	47.9337

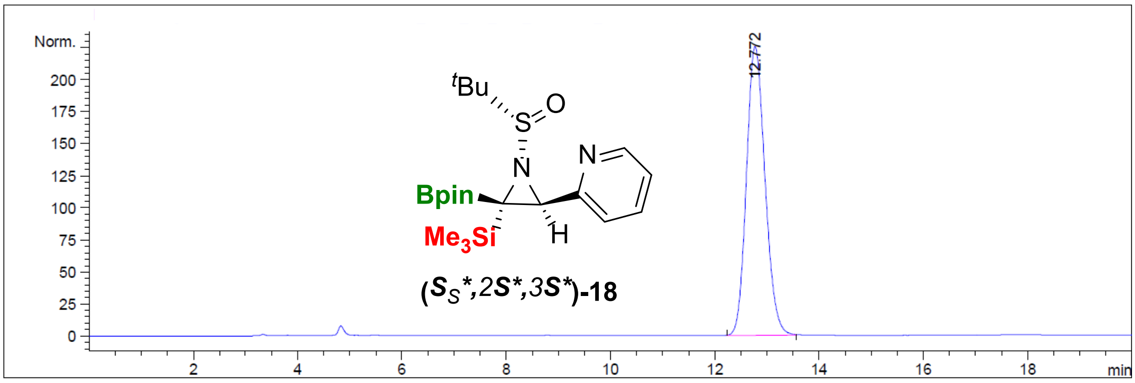

Peak #	RetTime [min]	Type	Width [min]	Area [mAU*s]	Height [mAU]	Area %
1	12.772	BB	0.3827	5576.12500	225.32788	100.0000

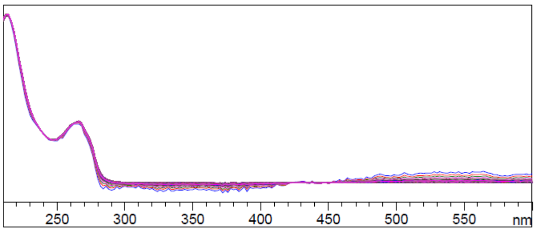

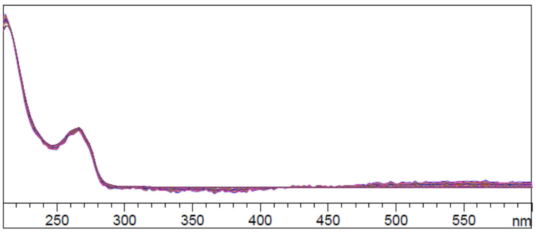

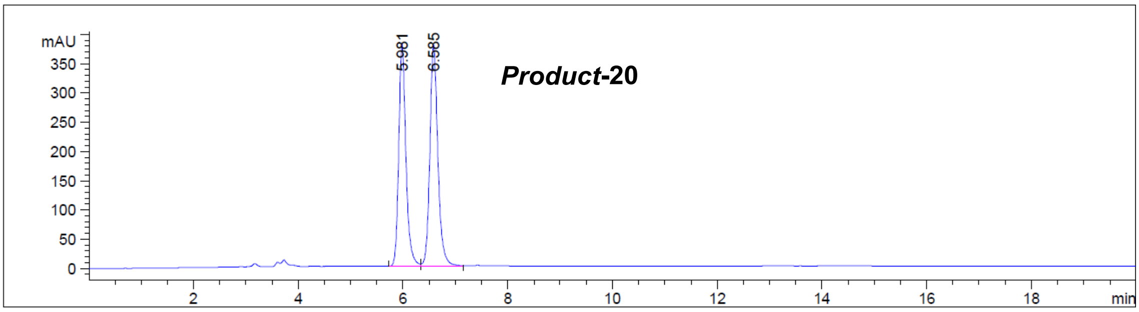

Peak #	RetTime [min]	Type	Width [min]	Area [mAU*s]	Height [mAU]	Area %
1	5.981	BV	0.1444	3608.14282	381.23334	47.2344
2	6.585	VB	0.1606	4030.65918	377.52063	52.7656

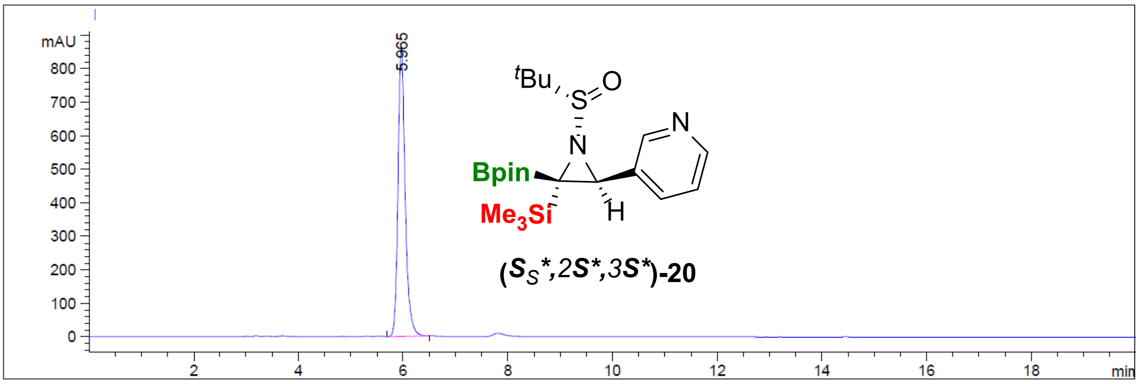

Peak #	RetTime [min]	Type	Width [min]	Area [mAU*s]	Height [mAU]	Area %
1	5.965	VB	0.1430	8237.04492	865.68536	100.0000

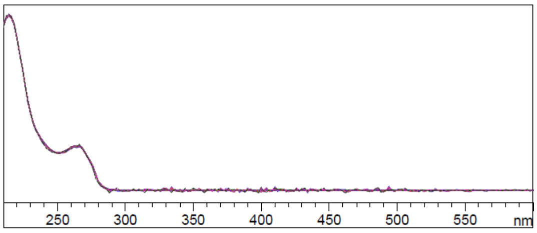

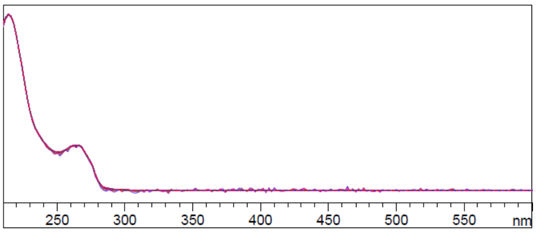

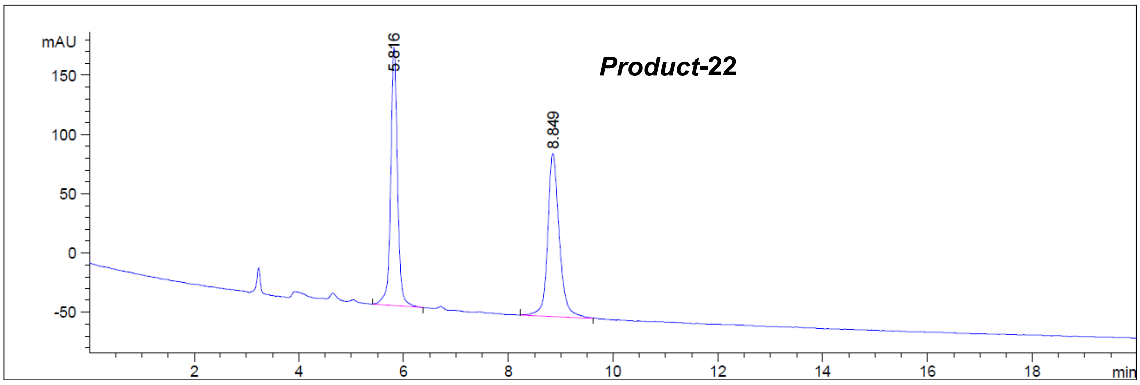

Peak #	RetTime [min]	Type	Width [min]	Area [mAU*s]	Height [mAU]	Area %
1	5.816	VB	0.1354	1971.14978	218.22327	48.1554
2	8.849	BB	0.2297	2122.15967	137.73914	51.8446

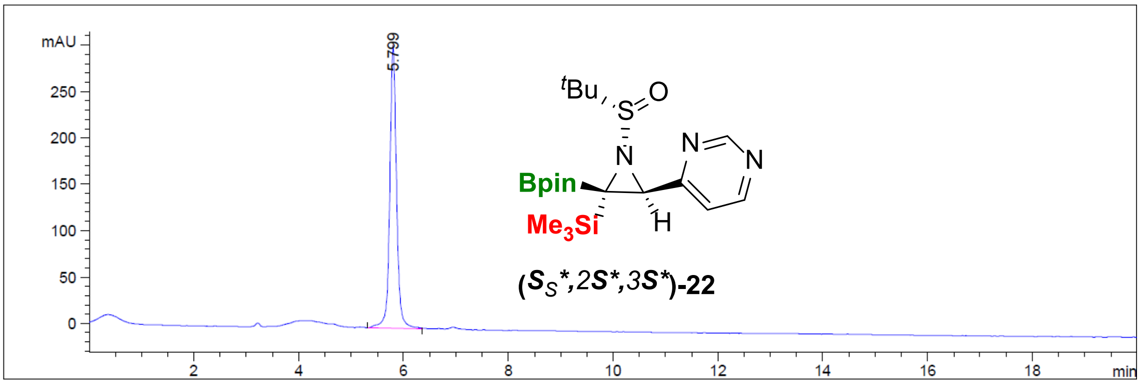

Peak #	RetTime [min]	Type	Width [min]	Area [mAU*s]	Height [mAU]	Area %
1	5.799	VB	0.1375	2745.46484	303.63528	100.0000

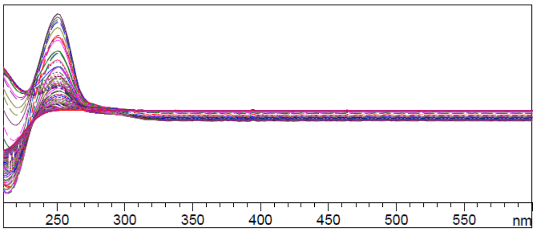

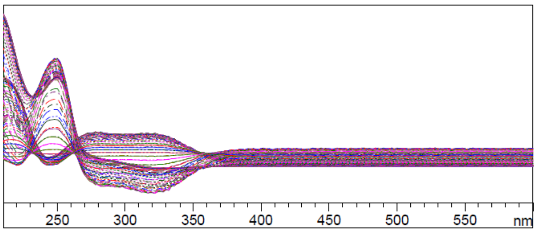

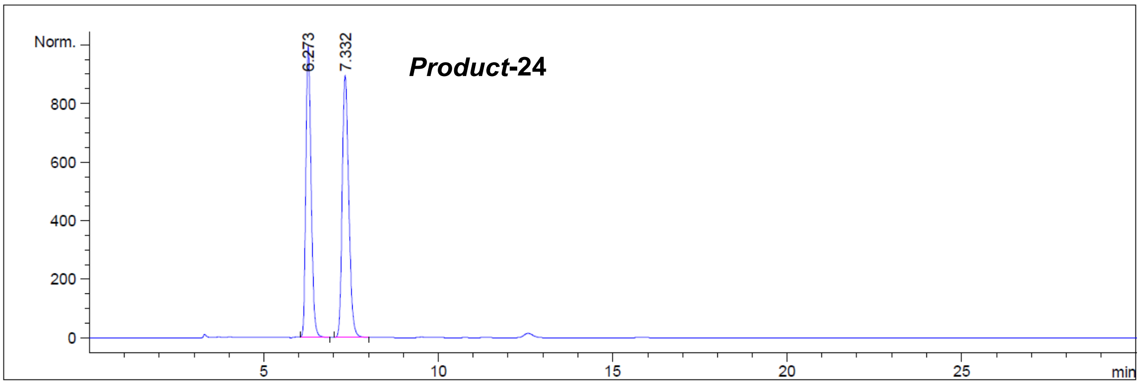

Peak #	RetTime [min]	Type	Width [min]	Area [mAU*s]	Height [mAU]	Area %
1	6.273	VB	0.1597	1.03556e4	992.83441	47.1162
2	7.332	BB	0.1997	1.16233e4	894.06555	52.8838

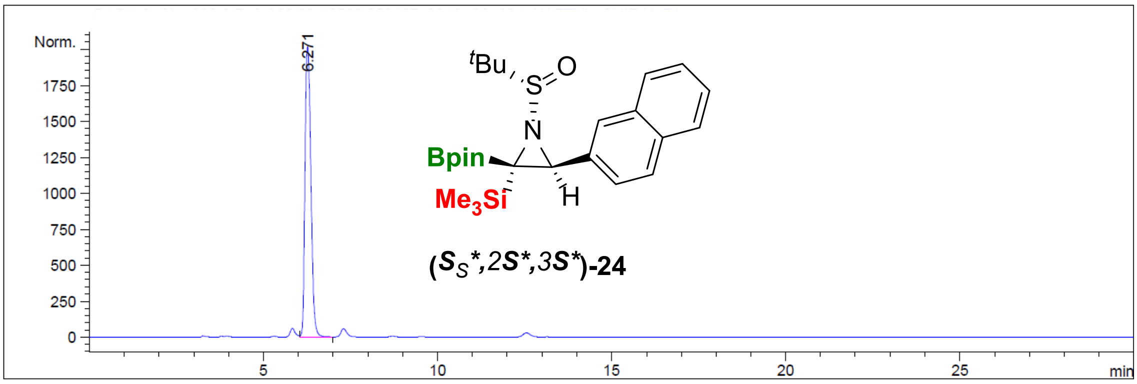

Peak #	RetTime [min]	Type	Width [min]	Area [mAU*s]	Height [mAU]	Area %
1	6.271	VB	0.1839	2.34946e4	2017.36609	100.0000

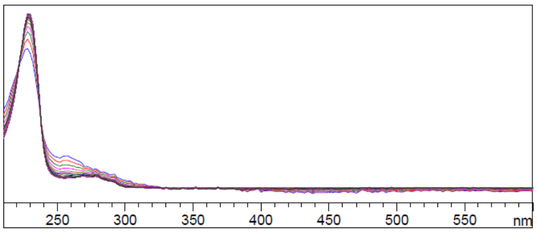

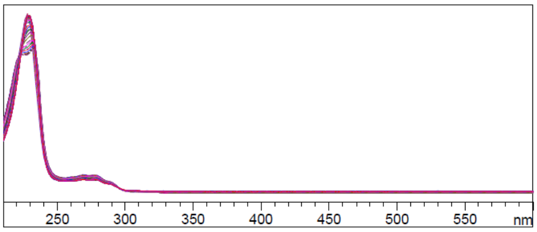

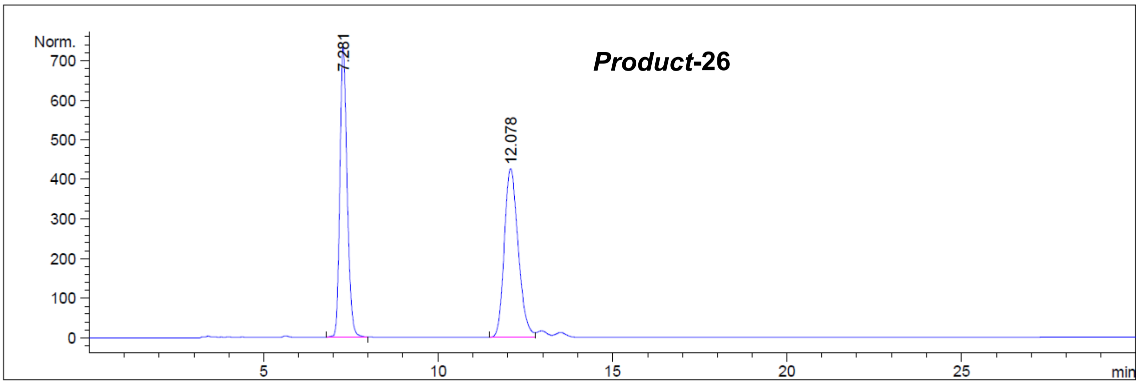

Peak #	RetTime [min]	Type	Width [min]	Area [mAU*s]	Height [mAU]	Area %
1	7.281	BB	0.2260	1.06942e4	733.71222	47.4606
2	12.078	BV	0.4299	1.18386e4	426.37903	52.5394

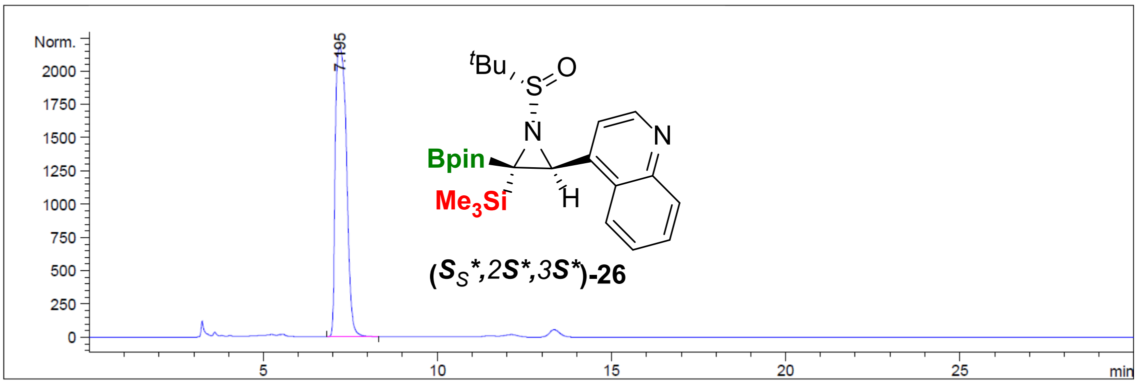

Peak #	RetTime [min]	Type	Width [min]	Area [mAU*s]	Height [mAU]	Area %
1	7.195	VB	0.3638	4.81432e4	2179.01978	100.0000

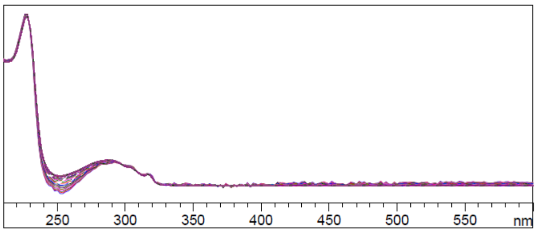

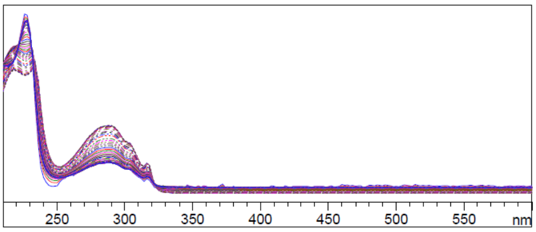

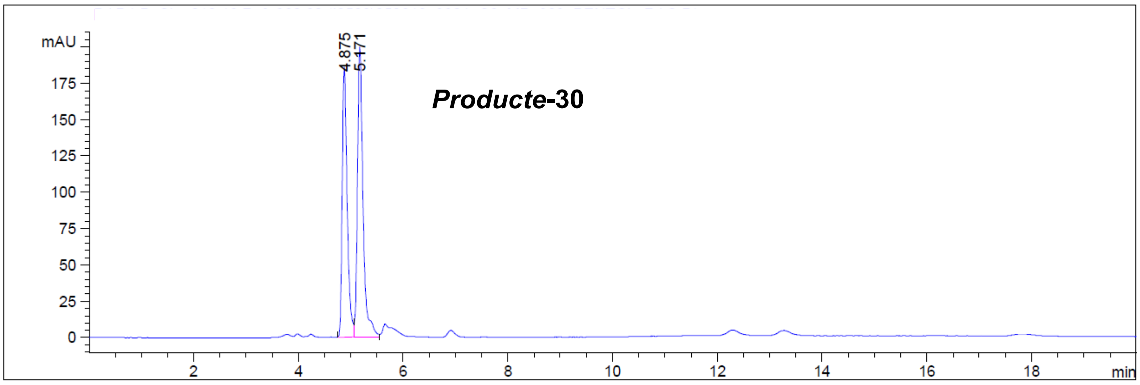

Peak #	RetTime [min]	Type	Width [min]	Area [mAU*s]	Height [mAU]	Area %
1	4.875	BV	0.0986	1223.25562	186.41304	45.3326
2	5.171	VV	0.1101	1475.14709	199.99069	54.6674

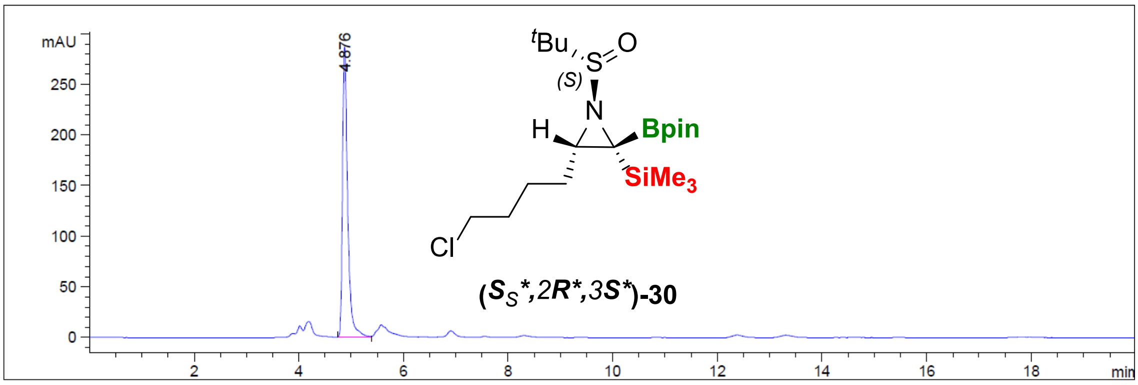

Peak #	RetTime [min]	Type	Width [min]	Area [mAU*s]	Height [mAU]	Area %
1	4.876	BV	0.1023	1977.32349	287.20380	100.0000

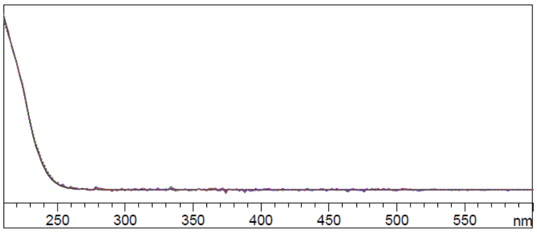

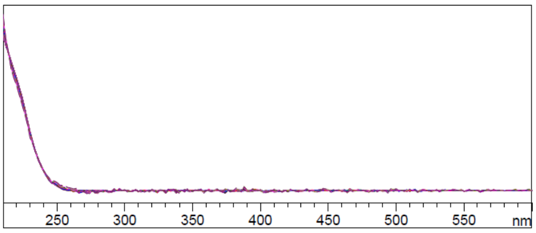

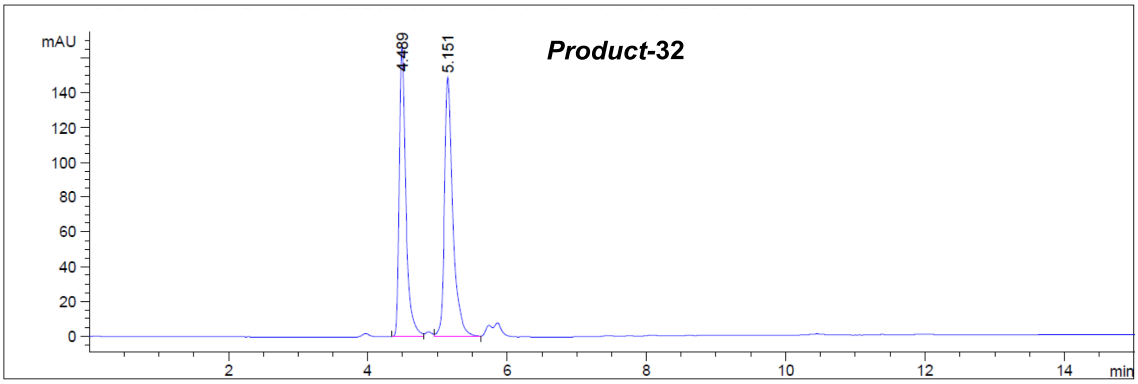

Peak #	RetTime [min]	Type	Width [min]	Area [mAU*s]	Height [mAU]	Area %
1	4.489	BV	0.0993	1106.79175	166.98595	46.9071
2	5.151	VV	0.1238	1252.74854	149.48329	53.0929

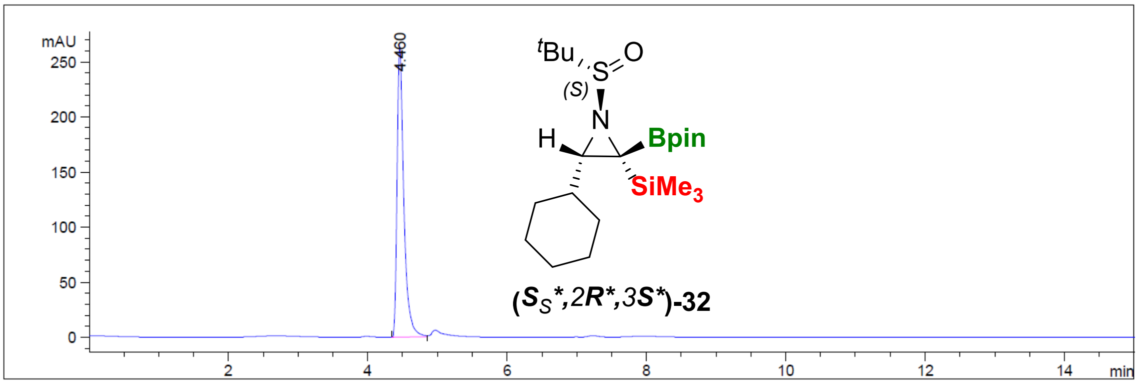

Peak #	RetTime [min]	Type	Width [min]	Area [mAU*s]	Height [mAU]	Area %
1	4.460	BV	0.0988	1735.00232	263.72305	100.0000

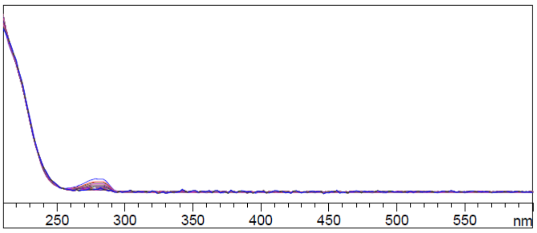

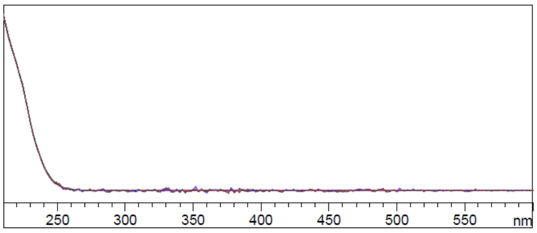

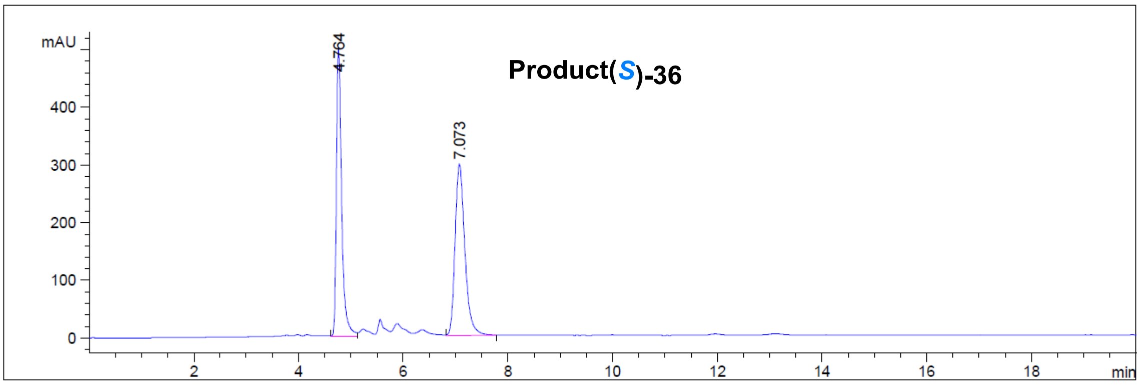

Peak #	RetTime [min]	Type	Width [min]	Area [mAU*s]	Height [mAU]	Area %
1	4.764	VV	0.1030	3489.47119	502.37268	47.3730
2	7.073	BB	0.2000	3876.47485	297.51785	52.6270

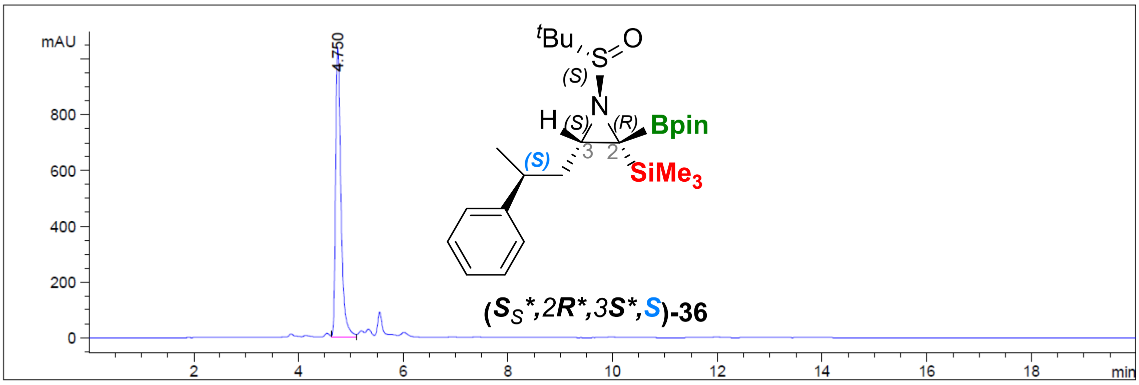

Peak #	RetTime [min]	Type	Width [min]	Area [mAU*s]	Height [mAU]	Area %
1	4.750	VV	0.1091	7432.17188	1042.80615	100.0000

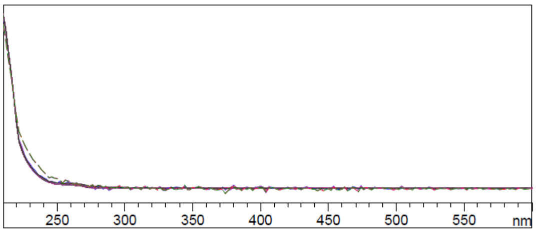

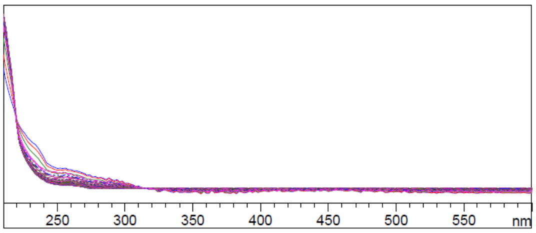

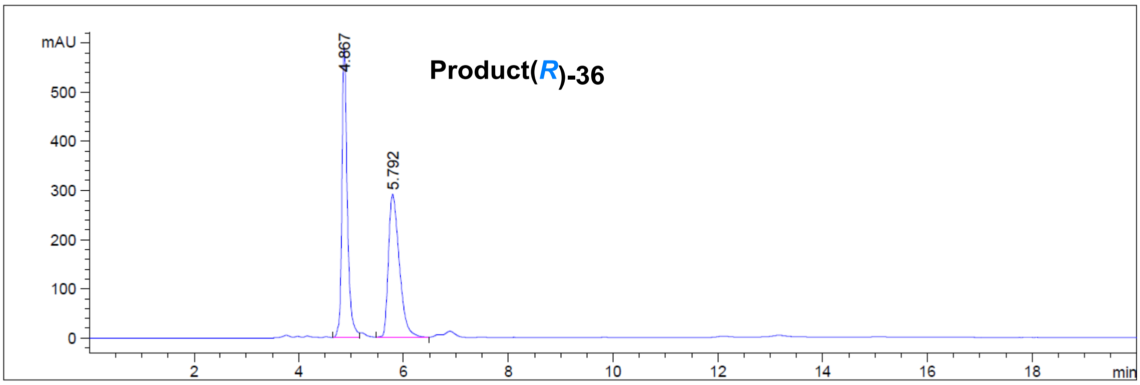

Peak #	RetTime [min]	Type	Width [min]	Area [mAU*s]	Height [mAU]	Area %
1	4.867	VV	0.1085	4287.09131	591.83551	50.2630
2	5.792	VB	0.2198	4242.21924	291.39072	49.7370

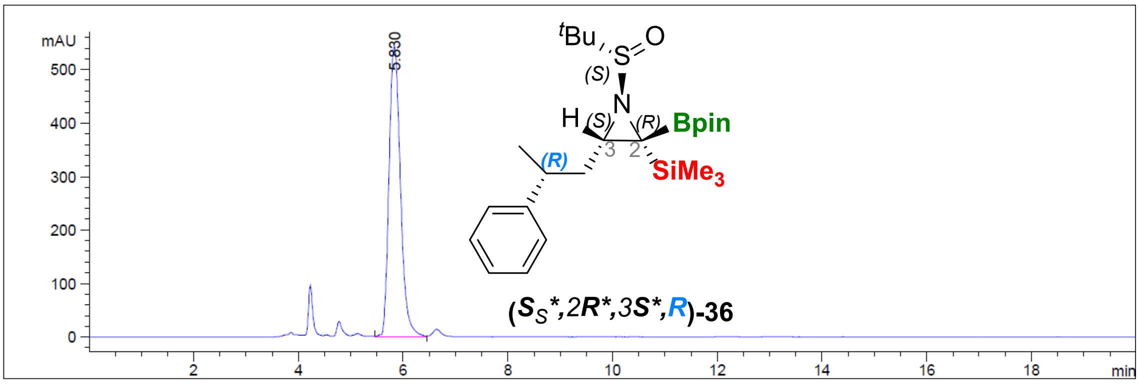

Peak #	RetTime [min]	Type	Width [min]	Area [mAU*s]	Height [mAU]	Area %
1	5.830	BV	0.2387	8235.91016	543.81891	100.0000

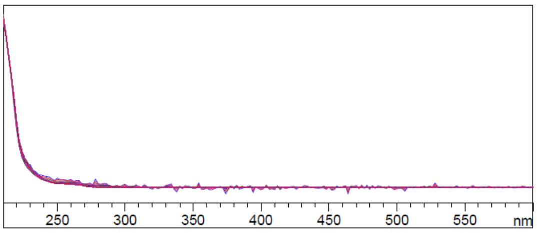

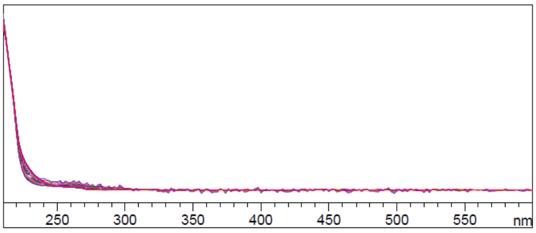

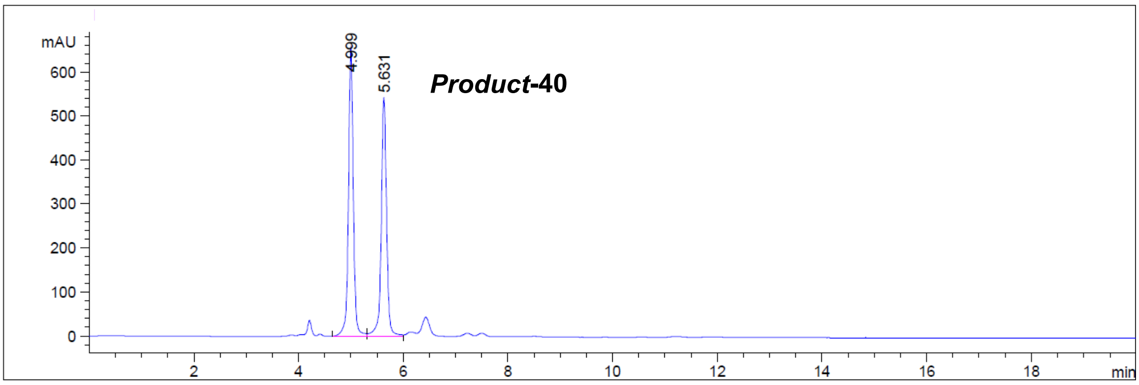

Peak #	RetTime [min]	Type	Width [min]	Area [mAU*s]	Height [mAU]	Area %
1	4.999	BV	0.0978	4282.35254	658.80554	52.2724
2	5.631	VV	0.1076	3910.02905	545.57025	47.7276

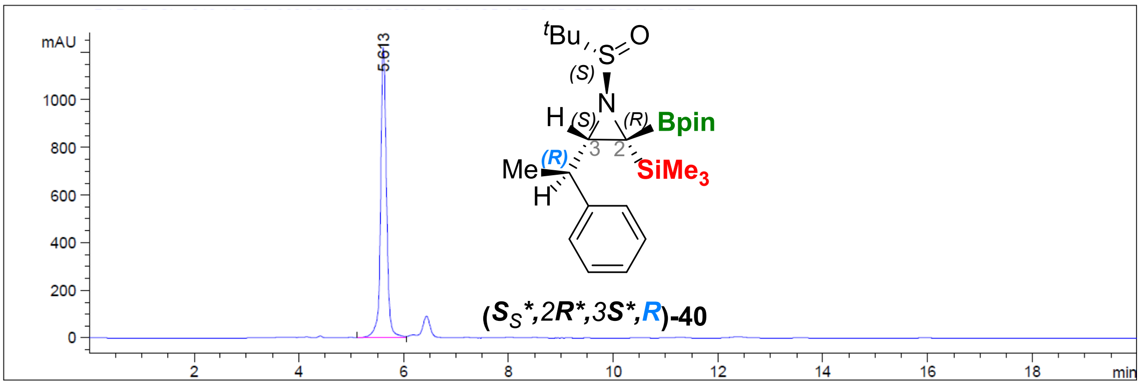

Peak #	RetTime [min]	Type	Width [min]	Area [mAU*s]	Height [mAU]	Area %
1	5.613	VV	0.1213	9577.00293	1223.90979	100.0000

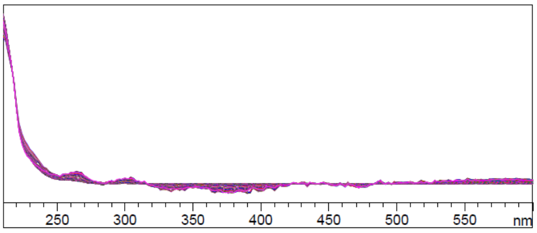

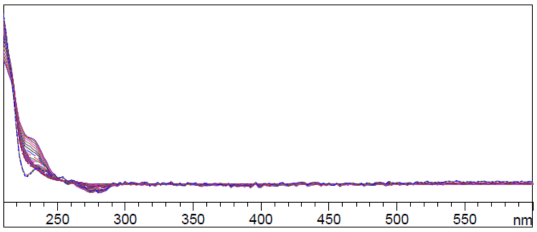

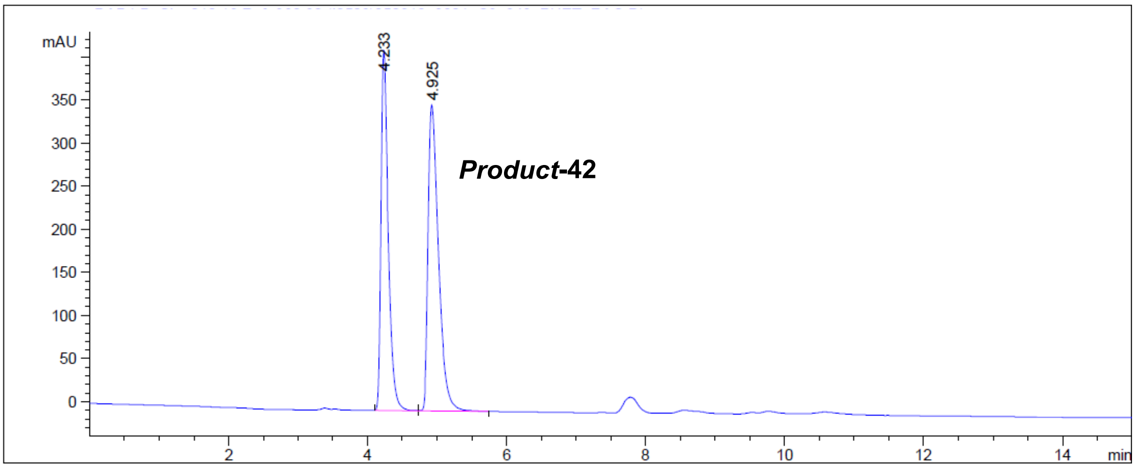

Peak #	RetTime [min]	Type	Width [min]	Area [mAU*s]	Height [mAU]	Area %
1	4.233	BV	0.1095	3061.68091	417.76492	45.4590
2	4.925	VB	0.1586	3673.34985	355.22791	54.5410

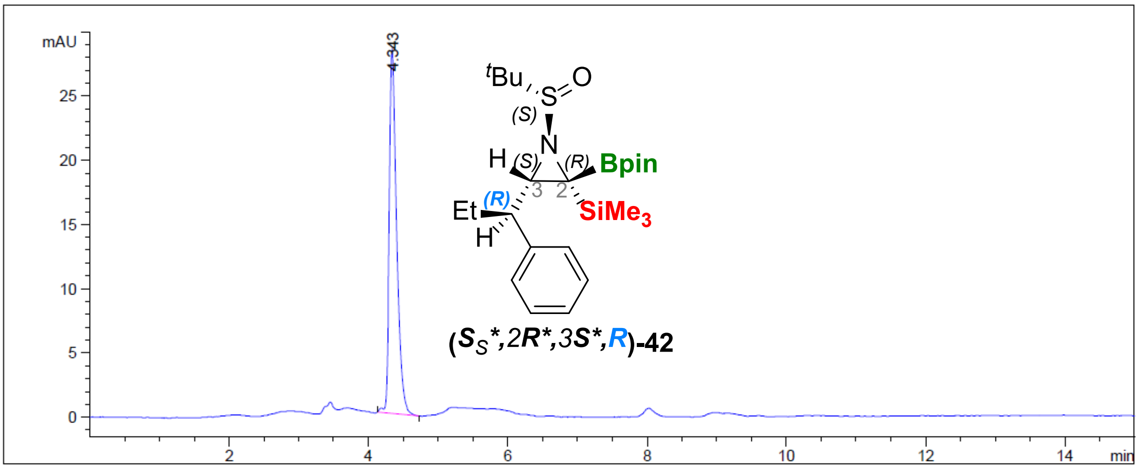

Peak #	RetTime [min]	Type	Width [min]	Area [mAU*s]	Height [mAU]	Area %
1	4.343	BB	0.1121	209.00146	28.33625	100.0000

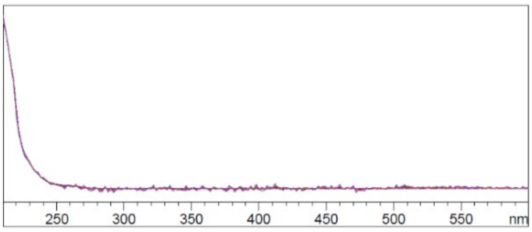

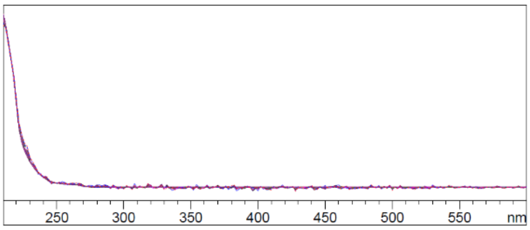

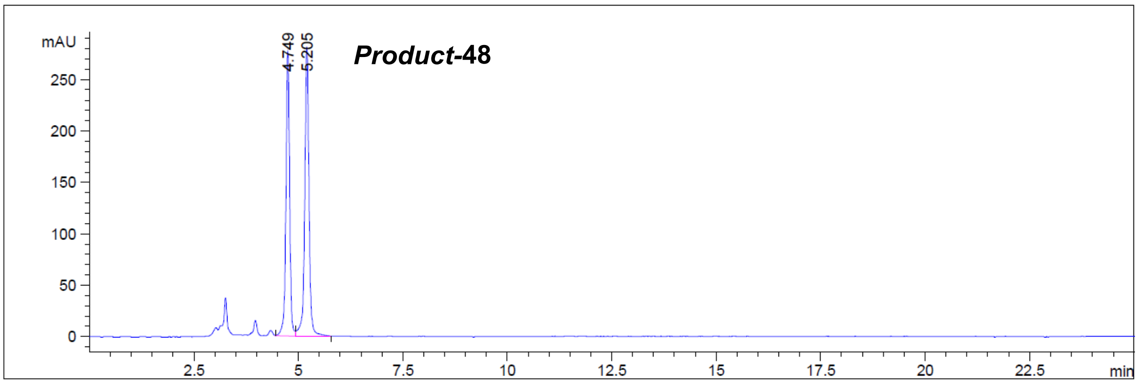

Peak #	RetTime [min]	Type	Width [min]	Area [mAU*s]	Height [mAU]	Area %
1	4.749	VV	0.0939	1702.92883	276.20187	45.8998
2	5.205	VB	0.1070	2007.16980	282.00668	54.1002

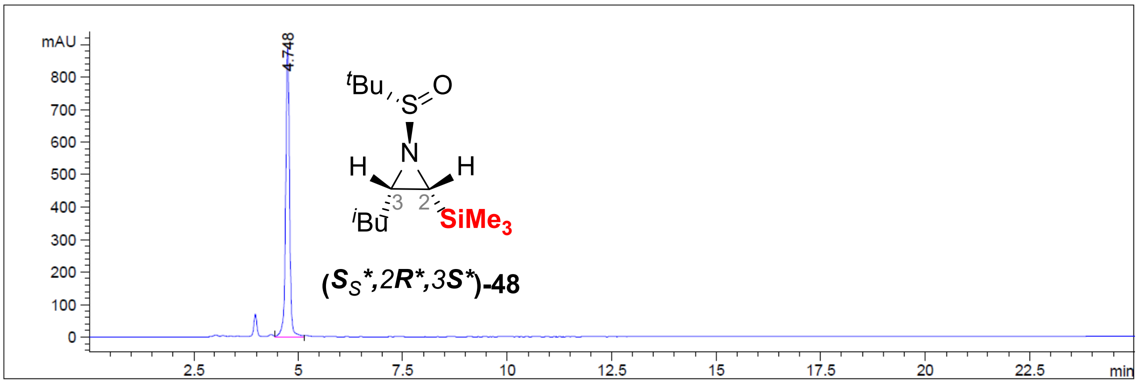

(S_S*,2R*,3S*)-48

Peak #	RetTime [min]	Type	Width [min]	Area [mAU*s]	Height [mAU]	Area %
1	4.748	VV	0.0988	5742.61865	895.70874	100.0000

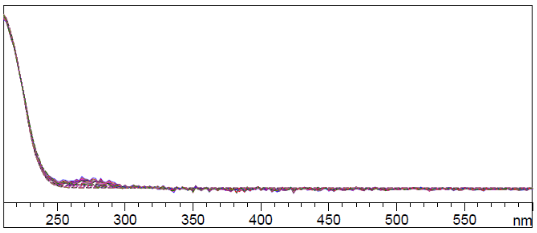
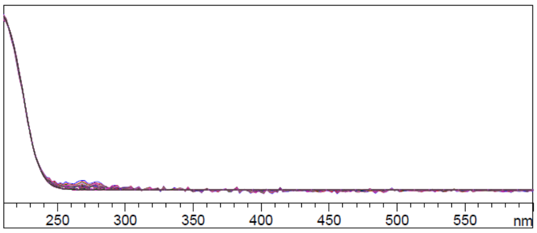

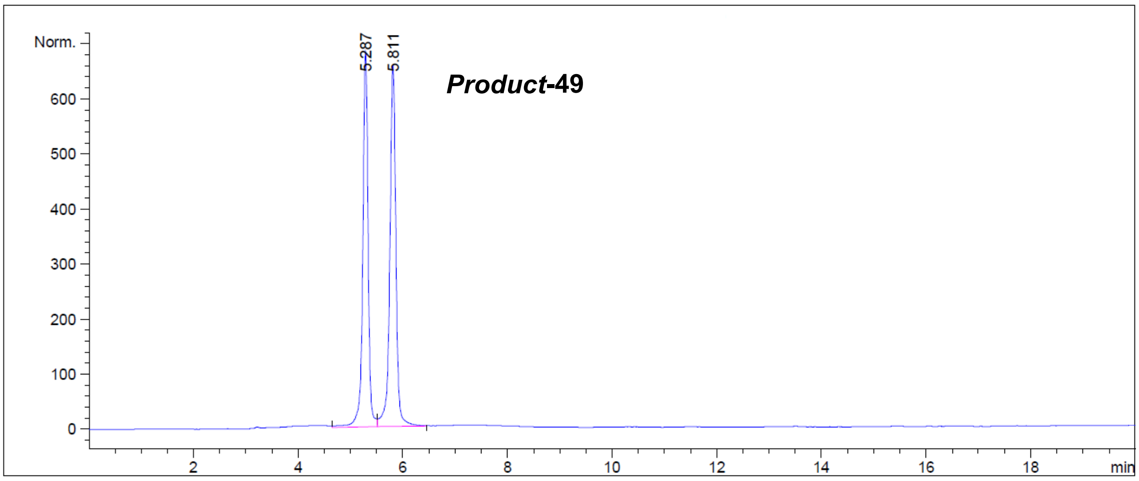

Peak #	RetTime [min]	Type	Width [min]	Area [mAU*s]	Height [mAU]	Area %
1	5.287	VV	0.1077	4896.65332	682.77472	47.7774
2	5.811	VB	0.1230	5352.23877	657.01483	52.2226

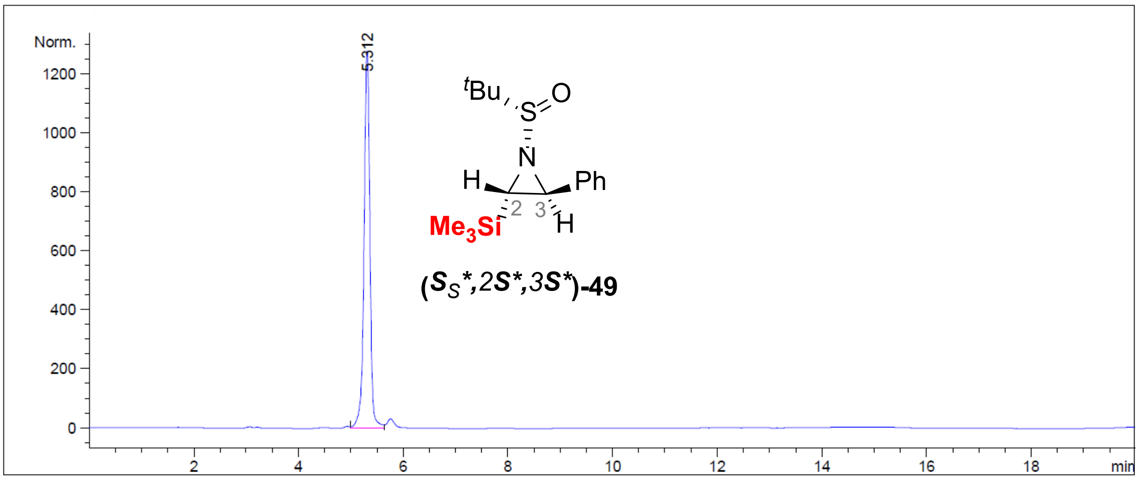

Peak #	RetTime [min]	Type	Width [min]	Area [mAU*s]	Height [mAU]	Area %
1	5.312	VV	0.1217	1.00558e4	1279.70093	100.0000

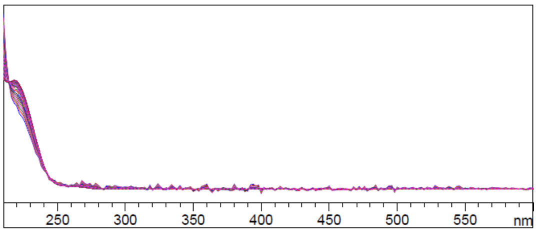

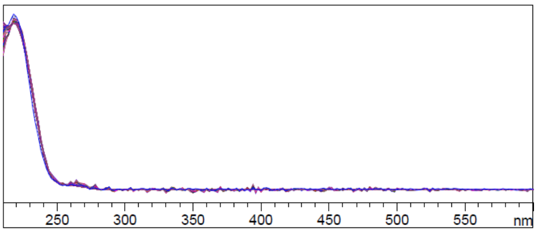

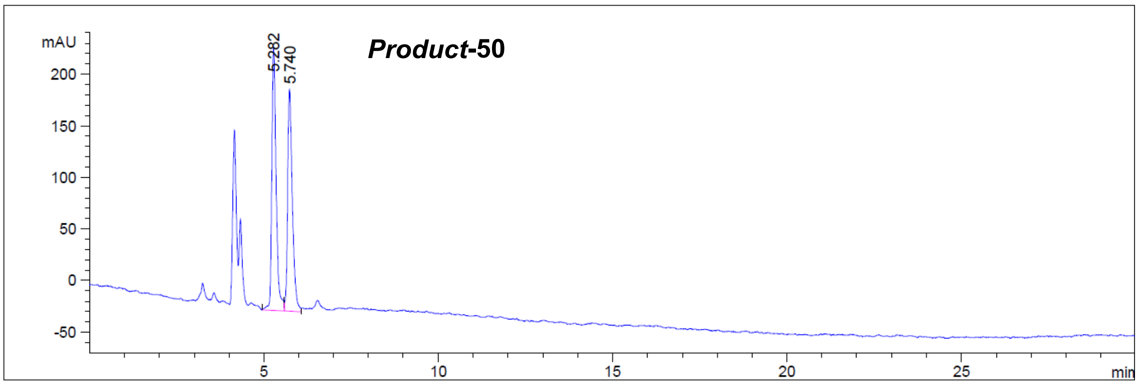

Peak #	RetTime [min]	Type	Width [min]	Area [mAU*s]	Height [mAU]	Area %
1	5.282	VV	0.1271	2173.01929	255.74249	51.8005
2	5.740	VV	0.1374	2021.95447	215.62259	48.1995

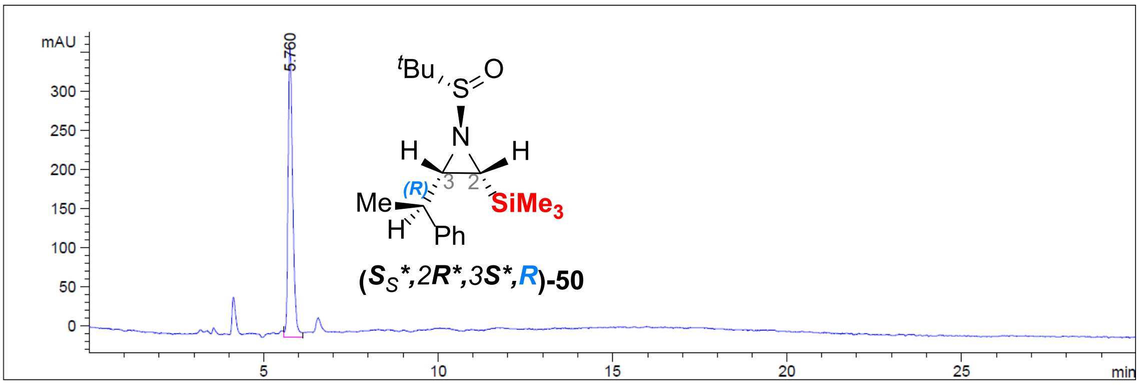

(S_S*,2R*,3S*,R)-50

Peak #	RetTime [min]	Type	Width [min]	Area [mAU*s]	Height [mAU]	Area %
1	5.760	VB	0.1372	3416.93994	371.87527	100.0000

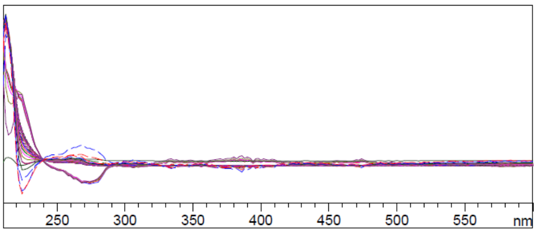

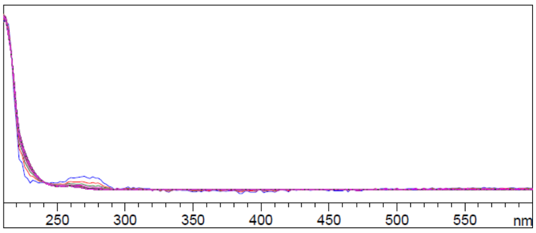

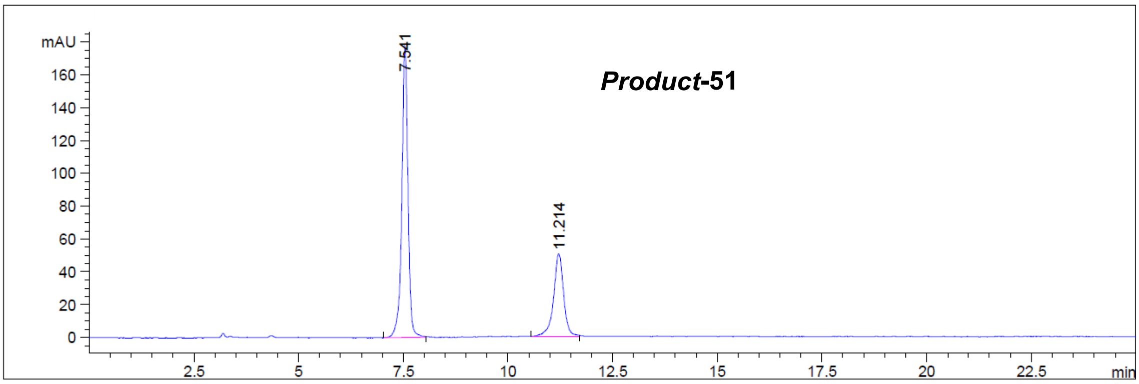

Peak #	RetTime [min]	Type	Width [min]	Area [mAU*s]	Height [mAU]	Area %
1	7.541	BB	0.1562	1822.47778	176.93500	68.6346
2	11.214	BB	0.2456	832.85504	50.18631	31.3654

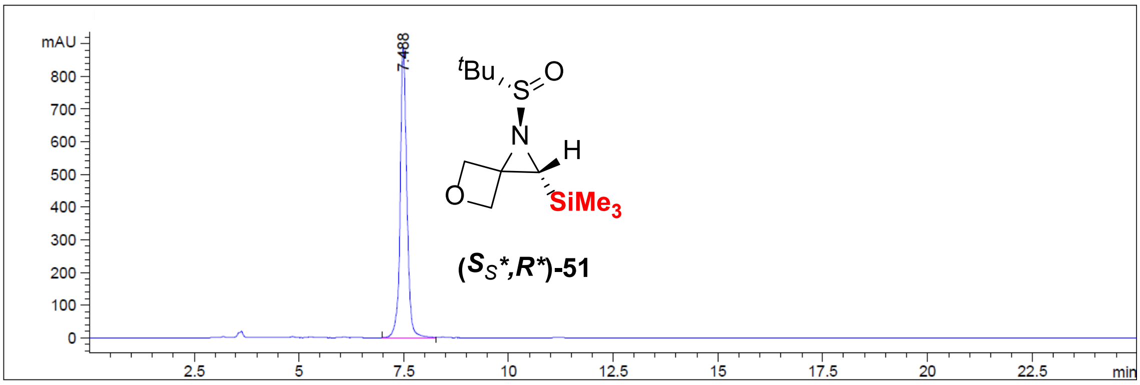

Peak #	RetTime [min]	Type	Width [min]	Area [mAU*s]	Height [mAU]	Area %
1	7.488	BB	0.1673	9869.80859	890.65790	100.0000

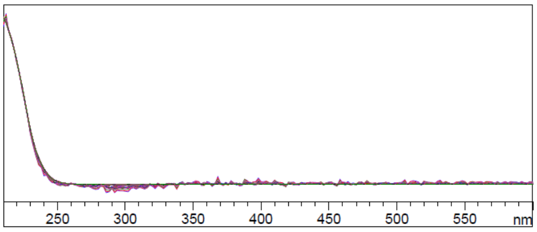

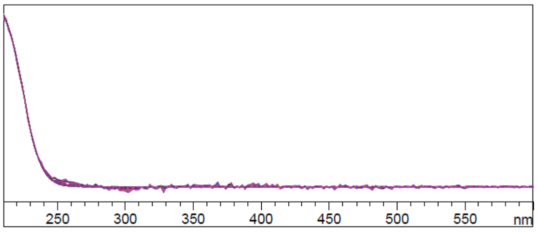

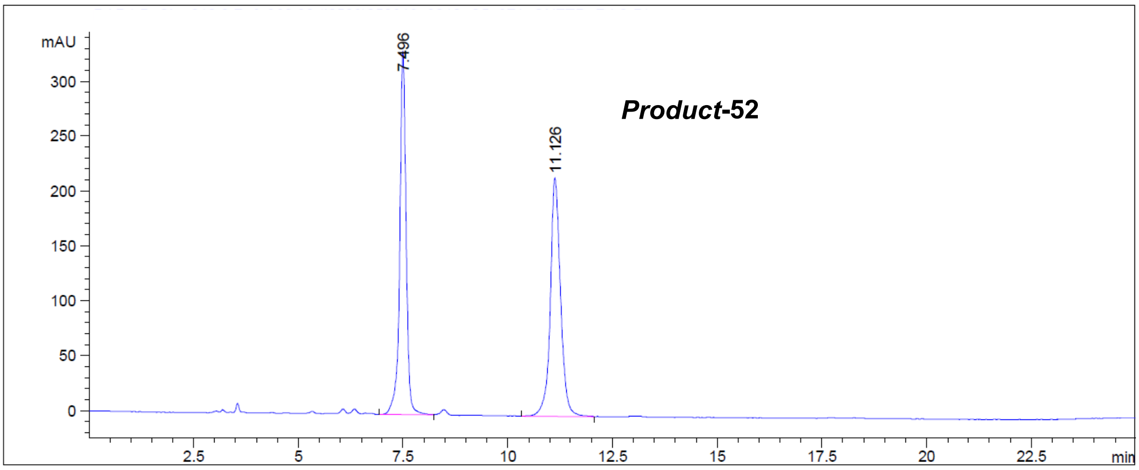

Peak #	RetTime [min]	Type	Width [min]	Area [mAU*s]	Height [mAU]	Area %
1	7.496	VV	0.1604	3536.77246	331.69476	48.7709
2	11.126	BV	0.2574	3715.04150	217.31384	51.2291

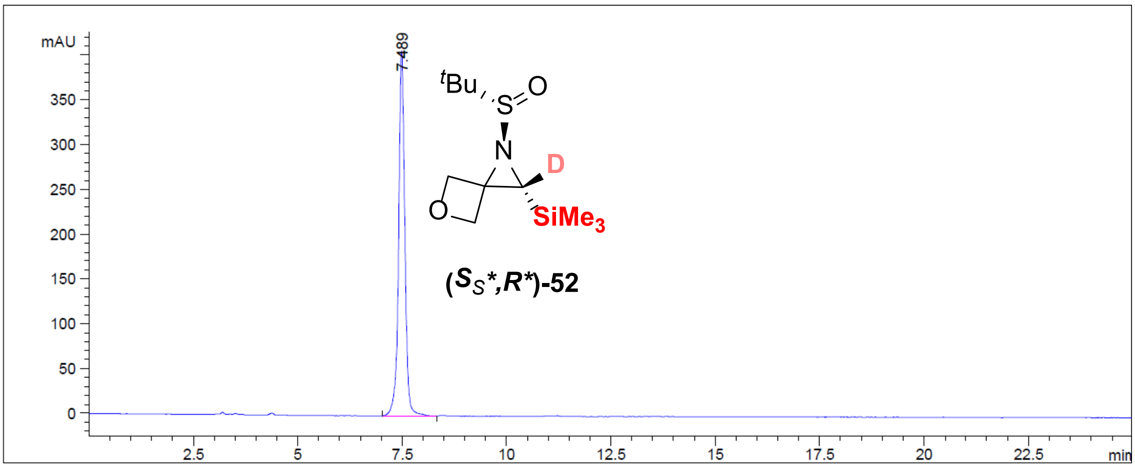

Peak #	RetTime [min]	Type	Width [min]	Area [mAU*s]	Height [mAU]	Area %
1	7.489	BV	0.1579	4262.16504	408.00632	100.0000

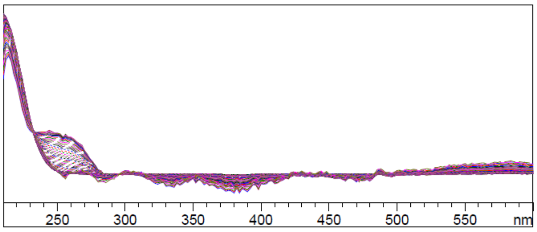

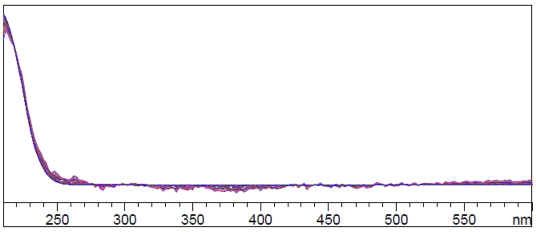

X-Ray single-crystal diffraction analysis

For product (*S*$_S$*,*R)-2** CCDC-2456375

Code (MP825F19_twin1_hklf5)

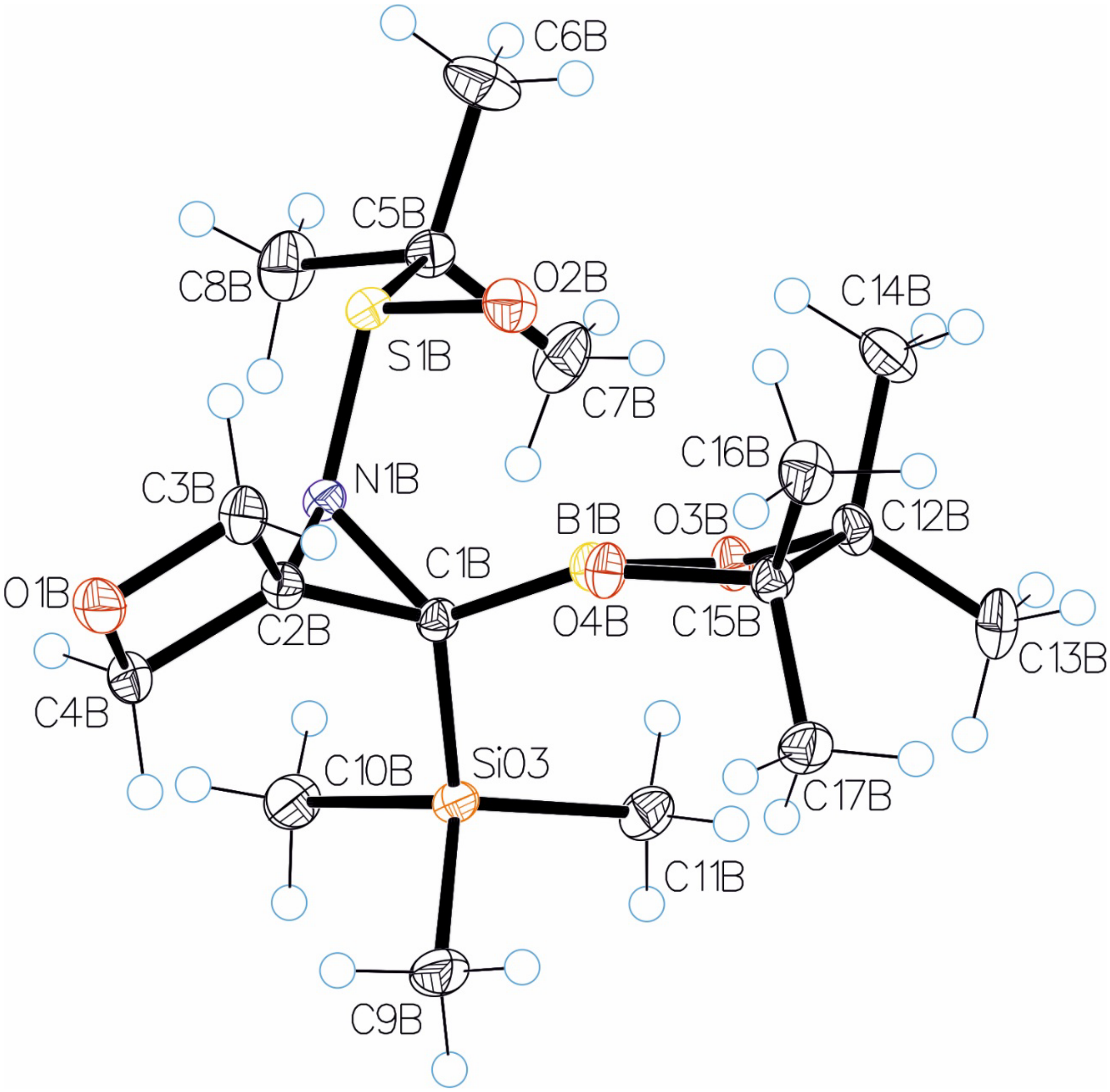

Table A.1 Crystal data and structure refinement for MP825F19_twin1_hklf5.

Identification code	MP825F19_twin1_hklf5
Empirical formula	$C_{34}H_{68}B_2N_2O_8S_2Si_2$
Formula weight	774.82
Temperature/K	100(1)
Crystal system	triclinic
Space group	P1
a/Å	9.6138(2)
b/Å	10.3698(3)

c/Å	11.6300(4)
α/°	90.818(3)
β/°	90.938(3)
γ/°	109.028(2)
Volume/Å^3	1095.70(6)
Z	1
ρ_{calc}g/cm^3	1.174
μ/mm^{-1}	0.222
F(000)	420.0
Crystal size/mm^3	0.2 × 0.2 × 0.2
Radiation	Mo Kα (λ = 0.71073)
2Θ range for data collection/°	5.022 to 63.094
Index ranges	-14 ≤ h ≤ 14, -15 ≤ k ≤ 15, -17 ≤ l ≤ 17
Reflections collected	23233
Independent reflections	23233 [R_{int} = ?, R_{sigma} = 0.0143]
Data/restraints/parameters	23233/3/471
Goodness-of-fit on F^2	1.059
Final R indexes [I>=2σ (I)]	R_1 = 0.0372, wR_2 = 0.1052
Final R indexes [all data]	R_1 = 0.0378, wR_2 = 0.1056
Largest diff. peak/hole / e Å^{-3}	0.32/-0.26

Table A.2 Fractional Atomic Coordinates (×10^4) and Equivalent Isotropic Displacement Parameters (Å^2×10^3) for MP825F19_twin1_hklf5. U_{eq} is defined as 1/3 of the trace of the orthogonalised U_{IJ} tensor.

Atom	*x*	*y*	*z*	U(eq)
S1B	5304.9(4)	8662.8(4)	8340.0(4)	14.63(9)
Si03	1220.2(6)	7101.7(5)	6302.4(5)	16.38(11)
O1B	1558.8(17)	8142(2)	10239.4(17)	25.1(3)
O2B	5349.6(18)	7237.0(16)	8417.4(17)	21.3(3)
O3B	3537.2(16)	5314.9(14)	6646.0(15)	16.8(3)
O4B	2726.1(16)	4909.3(14)	8492.6(15)	16.7(3)
N1B	3587.3(17)	8566.1(16)	7835.5(16)	14.0(3)

Table A.2 Fractional Atomic Coordinates (×10[4]) and Equivalent Isotropic Displacement Parameters (Å^2×10^3) for MP825F19_twin1_hklf5. U_{eq} is defined as 1/3 of the trace of the orthogonalised U_{IJ} tensor.

Atom	*x*	*y*	*z*	U(eq)
C1B	2549.6(19)	7123.5(18)	7540.0(18)	13.6(3)
C2B	2426(2)	7857.8(19)	8625.8(19)	15.2(3)
C3B	2639(2)	7517(2)	9873(2)	20.3(4)
C4B	1299(2)	8496(2)	9072(2)	20.4(4)
C5B	6347(2)	9431(2)	7078(2)	18.1(4)
C6B	7932(3)	9517(3)	7384(3)	34.7(6)
C7B	5771(3)	8566(3)	5997(2)	30.5(5)
C8B	6221(3)	10856(2)	6974(3)	28.1(5)
C9B	-646(2)	6012(3)	6757(3)	29.1(5)
C10B	1264(3)	8902(2)	6060(3)	26.8(5)
C11B	1684(2)	6367(2)	4943(2)	22.5(4)
C12B	3794(2)	4047.5(19)	6952(2)	17.2(3)
C13B	3291(3)	3053(2)	5938(2)	25.4(4)
C14B	5451(2)	4396(2)	7165(3)	27.7(5)
C15B	2857(2)	3620.5(19)	8059.3(19)	15.8(3)
C16B	3568(3)	3017(2)	8995(2)	25.1(4)
C17B	1292(2)	2669(2)	7806(2)	23.1(4)
B1B	3091(2)	5826(2)	7617(2)	15.2(4)
S1A	5326.2(5)	1661.4(4)	3581.0(4)	14.65(9)
Si1A	8853.6(6)	2737.9(6)	1082.4(6)	18.48(12)
O1A	9326(2)	2525(2)	5129.6(18)	30.2(4)
O2A	5338.4(19)	3104.8(16)	3476.6(17)	22.2(3)
O3A	6441.8(16)	4513.2(14)	1502.1(15)	17.8(3)
O4A	7907(2)	5337.2(16)	3107.2(17)	23.9(3)
N1A	6898.7(17)	1592.5(16)	2954.0(16)	14.8(3)
C1A	7795(2)	2932.4(19)	2399.0(18)	14.9(3)
C2A	8229(2)	2522(2)	3534.6(19)	16.6(4)
C3A	9453(2)	2010(2)	3978(2)	24.0(4)
C4A	8319(2)	3211(2)	4714(2)	21.4(4)
C5A	3989(2)	609(2)	2512(2)	17.9(4)

Table A.2 Fractional Atomic Coordinates (×10^4) and Equivalent Isotropic Displacement Parameters (Å^2×10^3) for MP825F19_twin1_hklf5. U_{eq} is defined as 1/3 of the trace of the orthogonalised U_{IJ} tensor.

Atom	*x*	*y*	*z*	U(eq)
C6A	4429(3)	1041(2)	1291(2)	23.8(4)
C7A	3920(3)	-857(2)	2735(2)	27.3(5)
C8A	2534(3)	826(3)	2815(3)	35.7(6)
C9A	9020(3)	989(3)	1024(3)	27.7(5)
C10A	7917(3)	3009(3)	-269(2)	31.6(5)
C11A	10692(3)	4077(3)	1264(3)	32.5(6)
C12A	6660(2)	5976(2)	1554(2)	20.2(4)
C13A	7781(3)	6627(3)	629(3)	33.6(6)
C14A	5209(3)	6195(3)	1268(3)	28.3(5)
C15A	7265(3)	6386(2)	2811(2)	27.9(5)
C16A	6026(5)	6271(3)	3672(3)	41.8(7)
C17A	8437(5)	7770(3)	2955(4)	53.4(11)
B1A	7207(3)	4215(2)	2413(2)	18.9(4)

Table A.3 Anisotropic Displacement Parameters (Å^2×10^3) for MP825F19_twin1_hklf5. The Anisotropic displacement factor exponent takes the form: $-2\pi^2[h^2a^{*2}U_{11}+2hka^*b^*U_{12}+\ldots]$.

Atom	U_{11}	U_{22}	U_{33}	U_{23}	U_{13}	U_{12}
S1B	14.55(17)	13.83(18)	14.5(2)	-0.04(14)	-1.17(14)	3.28(16)
Si03	14.7(2)	14.9(2)	18.8(3)	-1.75(18)	-2.80(17)	4.02(19)
O1B	21.6(7)	36.7(9)	17.8(9)	-5.2(6)	3.9(5)	10.7(7)
O2B	22.5(6)	16.5(6)	26.2(9)	5.0(6)	-0.8(6)	8.1(6)
O3B	20.8(6)	13.5(5)	17.5(8)	1.9(5)	4.6(5)	7.2(5)
O4B	22.8(6)	12.8(5)	15.0(7)	0.9(5)	3.7(5)	6.1(5)
N1B	13.7(6)	12.6(6)	14.6(8)	-0.5(5)	-0.2(5)	2.8(6)
C1B	14.1(7)	11.2(7)	14.8(9)	0.5(6)	0.8(6)	3.0(6)
C2B	16.0(7)	13.9(7)	15.4(9)	0.4(6)	2.4(6)	4.4(7)
C3B	25.1(9)	22.4(9)	13.9(10)	0.2(7)	4.2(7)	8.2(8)
C4B	19.2(8)	21.2(8)	21.6(11)	-3.8(7)	2.7(7)	7.8(8)
C5B	15.8(7)	14.8(8)	21.4(10)	1.1(7)	3.5(6)	1.5(7)

Table A.3 Anisotropic Displacement Parameters (Å^2×10^3) for MP825F19_twin1_hklf5. The Anisotropic displacement factor exponent takes the form: -2π^2[h^{2}a*^{2}U$_{11}$+2hka*b*U$_{12}$+…].

Atom	U_{11}	U_{22}	U_{33}	U_{23}	U_{13}	U_{12}
C6B	17.0(9)	41.1(14)	45.7(18)	8.6(12)	5.2(9)	8.6(10)
C7B	36.6(12)	28.3(11)	18.8(12)	-4.9(8)	9.9(9)	-0.4(10)
C8B	35.4(11)	15.9(9)	31.1(14)	5.9(8)	6.2(9)	5.5(9)
C9B	16.3(8)	30.5(11)	34.5(14)	-9.8(10)	1.5(8)	-0.1(9)
C10B	29.5(10)	20.4(9)	32.6(14)	-0.8(8)	-10.5(9)	11.4(9)
C11B	24.7(9)	24.0(9)	16.9(10)	-2.3(7)	-1.9(7)	5.7(8)
C12B	17.9(8)	13.2(7)	21.7(10)	1.1(6)	4.3(7)	6.7(7)
C13B	35.3(11)	19.3(9)	22.6(12)	-3.1(7)	8.4(9)	10.2(9)
C14B	17.3(8)	19.9(9)	47.6(17)	4.6(9)	6.5(9)	7.9(8)
C15B	17.4(7)	11.7(7)	18.1(10)	0.9(6)	1.2(6)	4.3(7)
C16B	34.5(11)	22.1(9)	22.5(12)	3.8(8)	-2.3(8)	14.6(9)
C17B	18.7(8)	20.3(9)	26.0(12)	-1.5(8)	4.3(7)	0.6(8)
B1B	17.2(8)	12.8(8)	16.1(10)	1.6(7)	2.1(7)	5.3(7)
S1A	17.01(18)	13.49(18)	12.9(2)	0.77(14)	2.28(14)	4.17(16)
Si1A	19.4(2)	20.1(2)	17.0(3)	3.05(19)	4.81(18)	7.6(2)
O1A	30.6(8)	42.0(10)	19.3(9)	2.9(7)	-8.0(6)	13.9(8)
O2A	30.0(7)	15.2(6)	23.3(9)	-0.2(5)	5.6(6)	9.8(6)
O3A	22.5(6)	14.5(6)	17.3(8)	-0.5(5)	-2.0(5)	7.5(5)
O4A	31.8(8)	14.0(6)	24.4(9)	-1.4(5)	-11.0(6)	6.0(6)
N1A	15.1(6)	14.4(6)	14.1(8)	1.1(5)	0.6(5)	3.6(6)
C1A	15.2(7)	14.8(7)	13.9(9)	1.8(6)	1.1(6)	3.8(7)
C2A	15.4(7)	16.4(8)	16.4(10)	2.1(6)	-1.6(6)	2.9(7)
C3A	20.2(8)	27.5(10)	24.4(12)	4.9(8)	-3.2(7)	8.0(8)
C4A	25.1(9)	21.6(9)	14.8(10)	0.3(7)	-3.7(7)	4.0(8)
C5A	16.2(7)	16.3(8)	19.4(10)	0.7(7)	-1.0(6)	3.0(7)
C6A	27.3(9)	21.5(9)	17.7(11)	2.6(7)	-4.3(8)	1.4(8)
C7A	36.9(12)	14.5(8)	24.1(12)	1.6(7)	-5.9(9)	0.1(9)
C8A	19.4(9)	45.9(15)	41.5(17)	-3.4(12)	0.4(9)	10.5(10)
C9A	32.2(11)	25.7(10)	29.6(14)	1.0(9)	7.1(9)	15.3(10)
C10A	40.3(12)	48.6(14)	15.4(12)	4.8(10)	5.3(9)	27.1(12)

Table A.3 Anisotropic Displacement Parameters (Å^2×10^3) for MP825F19_twin1_hklf5. The Anisotropic displacement factor exponent takes the form: -2π^2[h^{2}a*^{2}U$_{11}$+2hka*b*U$_{12}$+…].

Atom	U_{11}	U_{22}	U_{33}	U_{23}	U_{13}	U_{12}
C11A	26.6(10)	29.0(11)	34.9(15)	1.8(10)	11.1(10)	-1.1(10)
C12A	26.7(9)	15.4(8)	19.6(10)	1.8(7)	-3.3(7)	8.6(8)
C13A	36.1(12)	27.6(11)	35.1(16)	11.6(10)	7.6(10)	7.1(11)
C14A	34.8(11)	29.2(10)	26.7(14)	-2.4(9)	-3.6(9)	18.8(10)
C15A	47.5(13)	13.7(8)	23.7(12)	-3.0(7)	-12.7(10)	12.5(9)
C16A	85(2)	34.0(13)	20.2(14)	-2.1(9)	-0.5(13)	38.1(15)
C17A	77(2)	15.5(10)	59(2)	-2.9(11)	-39(2)	5.1(13)
B1A	23.4(9)	15.3(9)	18.9(11)	-1.3(7)	-2.7(8)	7.9(8)

Table A.4 Bond Lengths for MP825F19_twin1_hklf5.

Atom	Atom	Length/Å	Atom	Atom	Length/Å
S1B	O2B	1.4970(17)	S1A	O2A	1.4996(17)
S1B	N1B	1.7145(17)	S1A	N1A	1.7109(18)
S1B	C5B	1.831(2)	S1A	C5A	1.839(2)
Si03	C1B	1.904(2)	Si1A	C1A	1.897(2)
Si03	C9B	1.871(2)	Si1A	C9A	1.872(3)
Si03	C10B	1.879(2)	Si1A	C10A	1.869(3)
Si03	C11B	1.868(3)	Si1A	C11A	1.864(2)
O1B	C3B	1.458(3)	O1A	C3A	1.457(3)
O1B	C4B	1.450(3)	O1A	C4A	1.457(3)
O3B	C12B	1.462(2)	O3A	C12A	1.462(2)
O3B	B1B	1.373(3)	O3A	B1A	1.375(3)
O4B	C15B	1.466(2)	O4A	C15A	1.457(3)
O4B	B1B	1.373(3)	O4A	B1A	1.380(3)
N1B	C1B	1.534(2)	N1A	C1A	1.534(2)
N1B	C2B	1.466(3)	N1A	C2A	1.473(2)
C1B	C2B	1.492(3)	C1A	C2A	1.488(3)
C1B	B1B	1.597(3)	C1A	B1A	1.605(3)
C2B	C3B	1.524(3)	C2A	C3A	1.526(3)
C2B	C4B	1.536(3)	C2A	C4A	1.525(3)

Table A.4 Bond Lengths for MP825F19_twin1_hklf5.

Atom	Atom	Length/Å	Atom	Atom	Length/Å
C5B	C6B	1.533(3)	C5A	C6A	1.521(3)
C5B	C7B	1.521(3)	C5A	C7A	1.526(3)
C5B	C8B	1.527(3)	C5A	C8A	1.534(3)
C12B	C13B	1.520(3)	C12A	C13A	1.536(4)
C12B	C14B	1.529(3)	C12A	C14A	1.517(3)
C12B	C15B	1.569(3)	C12A	C15A	1.565(4)
C15B	C16B	1.522(3)	C15A	C16A	1.545(5)
C15B	C17B	1.527(3)	C15A	C17A	1.513(3)

Table A.5 Bond Angles for MP825F19_twin1_hklf5.

Atom	Atom	Atom	Angle/˚	Atom	Atom	Atom	Angle/˚
O2B	S1B	N1B	107.68(9)	O2A	S1A	N1A	106.29(9)
O2B	S1B	C5B	107.58(10)	O2A	S1A	C5A	107.82(10)
N1B	S1B	C5B	98.01(9)	N1A	S1A	C5A	98.18(9)
C9B	Si03	C1B	106.62(11)	C9A	Si1A	C1A	109.92(11)
C9B	Si03	C10B	110.91(12)	C10A	Si1A	C1A	111.27(11)
C10B	Si03	C1B	108.27(10)	C10A	Si1A	C9A	108.89(14)
C11B	Si03	C1B	112.64(10)	C11A	Si1A	C1A	105.13(12)
C11B	Si03	C9B	108.79(11)	C11A	Si1A	C9A	111.32(13)
C11B	Si03	C10B	109.59(12)	C11A	Si1A	C10A	110.29(14)
C4B	O1B	C3B	92.31(16)	C4A	O1A	C3A	91.57(17)
B1B	O3B	C12B	107.71(16)	B1A	O3A	C12A	108.00(16)
B1B	O4B	C15B	107.52(17)	B1A	O4A	C15A	106.54(18)
C1B	N1B	S1B	115.43(13)	C1A	N1A	S1A	113.55(13)
C2B	N1B	S1B	112.33(14)	C2A	N1A	S1A	111.82(14)
C2B	N1B	C1B	59.59(12)	C2A	N1A	C1A	59.27(12)
N1B	C1B	Si03	112.37(13)	N1A	C1A	Si1A	115.25(13)
N1B	C1B	B1B	121.49(16)	N1A	C1A	B1A	120.21(17)
C2B	C1B	Si03	117.87(14)	C2A	C1A	Si1A	118.78(15)
C2B	C1B	N1B	57.94(12)	C2A	C1A	N1A	58.34(12)
C2B	C1B	B1B	118.67(18)	C2A	C1A	B1A	116.18(18)
B1B	C1B	Si03	116.02(14)	B1A	C1A	Si1A	115.93(15)

Table A.5 Bond Angles for MP825F19_twin1_hklf5.

Atom	Atom	Atom	Angle/˚	Atom	Atom	Atom	Angle/˚
N1B	C2B	C1B	62.47(12)	N1A	C2A	C1A	62.39(13)
N1B	C2B	C3B	126.50(17)	N1A	C2A	C3A	121.29(18)
N1B	C2B	C4B	122.36(18)	N1A	C2A	C4A	126.73(18)
C1B	C2B	C3B	130.08(18)	C1A	C2A	C3A	135.5(2)
C1B	C2B	C4B	133.89(18)	C1A	C2A	C4A	129.41(19)
C3B	C2B	C4B	86.51(17)	C4A	C2A	C3A	86.38(18)
O1B	C3B	C2B	90.68(17)	O1A	C3A	C2A	90.47(18)
O1B	C4B	C2B	90.50(17)	O1A	C4A	C2A	90.49(18)
C6B	C5B	S1B	104.01(18)	C6A	C5A	S1A	111.75(14)
C7B	C5B	S1B	111.37(14)	C6A	C5A	C7A	112.7(2)
C7B	C5B	C6B	111.9(2)	C6A	C5A	C8A	111.4(2)
C7B	C5B	C8B	111.5(2)	C7A	C5A	S1A	105.33(15)
C8B	C5B	S1B	106.91(16)	C7A	C5A	C8A	110.9(2)
C8B	C5B	C6B	110.78(19)	C8A	C5A	S1A	104.36(17)
O3B	C12B	C13B	107.92(19)	O3A	C12A	C13A	106.35(19)
O3B	C12B	C14B	106.96(16)	O3A	C12A	C14A	108.84(17)
O3B	C12B	C15B	102.44(15)	O3A	C12A	C15A	102.62(17)
C13B	C12B	C14B	110.14(19)	C13A	C12A	C15A	113.5(2)
C13B	C12B	C15B	114.68(16)	C14A	C12A	C13A	109.5(2)
C14B	C12B	C15B	114.0(2)	C14A	C12A	C15A	115.3(2)
O4B	C15B	C12B	103.42(15)	O4A	C15A	C12A	103.29(19)
O4B	C15B	C16B	108.73(18)	O4A	C15A	C16A	106.7(2)
O4B	C15B	C17B	106.58(16)	O4A	C15A	C17A	108.8(2)
C16B	C15B	C12B	114.70(18)	C16A	C15A	C12A	112.5(2)
C16B	C15B	C17B	109.52(18)	C17A	C15A	C12A	115.6(2)
C17B	C15B	C12B	113.32(18)	C17A	C15A	C16A	109.5(3)
O3B	B1B	O4B	113.31(17)	O3A	B1A	O4A	113.06(19)
O3B	B1B	C1B	120.17(18)	O3A	B1A	C1A	122.83(19)
O4B	B1B	C1B	123.39(18)	O4A	B1A	C1A	119.6(2)

Table A.6 Torsion Angles for MP825F19_twin1_hklf5.

A	B	C	D	Angle/˚	A	B	C	D	Angle/˚
S1B	N1B	C1B	Si03	-148.16(10)	S1A	N1A	C2A	C4A	15.0(3)
S1B	N1B	C1B	C2B	102.04(16)	Si1A	C1A	C2A	N1A	-103.48(16)
S1B	N1B	C1B	B1B	-4.2(2)	Si1A	C1A	C2A	C3A	4.2(3)
S1B	N1B	C2B	C1B	-107.29(15)	Si1A	C1A	C2A	C4A	140.17(18)
S1B	N1B	C2B	C3B	13.9(2)	Si1A	C1A	B1A	O3A	49.5(2)
S1B	N1B	C2B	C4B	126.17(18)	Si1A	C1A	B1A	O4A	-105.1(2)
Si03	C1B	C2B	N1B	-100.18(15)	O2A	S1A	N1A	C1A	-4.24(16)
Si03	C1B	C2B	C3B	143.84(17)	O2A	S1A	N1A	C2A	60.56(16)
Si03	C1B	C2B	C4B	9.5(3)	O2A	S1A	C5A	C6A	62.51(19)
Si03	C1B	B1B	O3B	45.5(2)	O2A	S1A	C5A	C7A	-174.82(16)
Si03	C1B	B1B	O4B	-113.1(2)	O2A	S1A	C5A	C8A	-58.00(19)
O2B	S1B	N1B	C1B	-2.41(17)	O3A	C12A	C15A	O4A	24.2(2)
O2B	S1B	N1B	C2B	63.34(16)	O3A	C12A	C15A	C16A	-90.4(2)
O2B	S1B	C5B	C6B	-65.74(18)	O3A	C12A	C15A	C17A	142.9(3)
O2B	S1B	C5B	C7B	55.0(2)	N1A	S1A	C5A	C6A	-47.61(18)
O2B	S1B	C5B	C8B	176.99(15)	N1A	S1A	C5A	C7A	75.07(17)
O3B	C12B	C15B	O4B	-22.85(19)	N1A	S1A	C5A	C8A	-168.11(17)
O3B	C12B	C15B	C16B	-141.09(17)	N1A	C1A	C2A	C3A	107.7(3)
O3B	C12B	C15B	C17B	92.13(19)	N1A	C1A	C2A	C4A	-116.3(2)
N1B	S1B	C5B	C6B	-177.23(17)	N1A	C1A	B1A	O3A	-96.8(3)
N1B	S1B	C5B	C7B	-56.54(19)	N1A	C1A	B1A	O4A	108.6(2)
N1B	S1B	C5B	C8B	65.50(16)	N1A	C2A	C3A	O1A	-123.7(2)
N1B	C1B	C2B	C3B	-116.0(2)	N1A	C2A	C4A	O1A	119.3(2)
N1B	C1B	C2B	C4B	109.6(2)	C1A	N1A	C2A	C3A	-128.6(2)
N1B	C1B	B1B	O3B	-97.2(2)	C1A	N1A	C2A	C4A	120.2(2)
N1B	C1B	B1B	O4B	104.2(2)	C1A	C2A	C3A	O1A	155.2(2)
N1B	C2B	C3B	O1B	128.3(2)	C1A	C2A	C4A	O1A	-158.52(19)
N1B	C2B	C4B	O1B	-131.71(19)	C2A	N1A	C1A	Si1A	109.55(16)
C1B	N1B	C2B	C3B	121.2(2)	C2A	N1A	C1A	B1A	-103.9(2)
C1B	N1B	C2B	C4B	-126.5(2)	C2A	C1A	B1A	O3A	-163.84(18)
C1B	C2B	C3B	O1B	-149.05(19)	C2A	C1A	B1A	O4A	41.6(3)
C1B	C2B	C4B	O1B	146.9(2)	C3A	O1A	C4A	C2A	8.12(16)

Table A.6 Torsion Angles for MP825F19_twin1_hklf5.

A	B	C	D	Angle/˚	A	B	C	D	Angle/˚
C2B	N1B	C1B	Si03	109.79(15)	C3A	C2A	C4A	O1A	-7.77(15)
C2B	N1B	C1B	B1B	-106.3(2)	C4A	O1A	C3A	C2A	-8.12(16)
C2B	C1B	B1B	O3B	-165.17(16)	C4A	C2A	C3A	O1A	7.77(15)
C2B	C1B	B1B	O4B	36.2(3)	C5A	S1A	N1A	C1A	107.12(14)
C3B	O1B	C4B	C2B	-0.12(15)	C5A	S1A	N1A	C2A	171.91(14)
C3B	C2B	C4B	O1B	0.11(15)	C9A	Si1A	C1A	N1A	-17.27(18)
C4B	O1B	C3B	C2B	0.12(15)	C9A	Si1A	C1A	C2A	48.97(18)
C4B	C2B	C3B	O1B	-0.11(15)	C9A	Si1A	C1A	B1A	-165.26(16)
C5B	S1B	N1B	C1B	108.99(15)	C10A	Si1A	C1A	N1A	103.44(17)
C5B	S1B	N1B	C2B	174.75(14)	C10A	Si1A	C1A	C2A	169.67(16)
C12B	O3B	B1B	O4B	-12.6(2)	C10A	Si1A	C1A	B1A	-44.55(19)
C12B	O3B	B1B	C1B	-173.23(16)	C11A	Si1A	C1A	N1A	-137.17(16)
C13B	C12B	C15B	O4B	-139.47(19)	C11A	Si1A	C1A	C2A	-70.94(18)
C13B	C12B	C15B	C16B	102.3(2)	C11A	Si1A	C1A	B1A	74.83(18)
C13B	C12B	C15B	C17B	-24.5(3)	C12A	O3A	B1A	O4A	1.5(3)
C14B	C12B	C15B	O4B	92.30(19)	C12A	O3A	B1A	C1A	-154.53(19)
C14B	C12B	C15B	C16B	-25.9(2)	C13A	C12A	C15A	O4A	-90.1(2)
C14B	C12B	C15B	C17B	-152.73(18)	C13A	C12A	C15A	C16A	155.2(2)
C15B	O4B	B1B	O3B	-3.4(2)	C13A	C12A	C15A	C17A	28.5(4)
C15B	O4B	B1B	C1B	156.57(18)	C14A	C12A	C15A	O4A	142.4(2)
B1B	O3B	C12B	C13B	142.95(17)	C14A	C12A	C15A	C16A	27.8(3)
B1B	O3B	C12B	C14B	-98.6(2)	C14A	C12A	C15A	C17A	-98.9(3)
B1B	O3B	C12B	C15B	21.6(2)	C15A	O4A	B1A	O3A	15.2(3)
B1B	O4B	C15B	C12B	16.5(2)	C15A	O4A	B1A	C1A	172.1(2)
B1B	O4B	C15B	C16B	138.79(18)	B1A	O3A	C12A	C13A	103.4(2)
B1B	O4B	C15B	C17B	-103.24(19)	B1A	O3A	C12A	C14A	-138.8(2)
B1B	C1B	C2B	N1B	111.10(18)	B1A	O3A	C12A	C15A	-16.1(2)
B1B	C1B	C2B	C3B	-4.9(3)	B1A	O4A	C15A	C12A	-24.0(3)
B1B	C1B	C2B	C4B	-139.3(2)	B1A	O4A	C15A	C16A	94.8(2)
S1A	N1A	C1A	Si1A	-148.18(11)	B1A	O4A	C15A	C17A	-147.3(3)
S1A	N1A	C1A	C2A	102.27(16)	B1A	C1A	C2A	N1A	110.83(18)
S1A	N1A	C1A	B1A	-1.7(2)	B1A	C1A	C2A	C3A	-141.5(2)

Table A.6 Torsion Angles for MP825F19_twin1_hklf5.

A	B	C	D	Angle/˚	A	B	C	D	Angle/˚
S1A	N1A	C2A	C1A	-105.23(15)	B1A	C1A	C2A	C4A	-5.5(3)
S1A	N1A	C2A	C3A	126.19(19)					

Table A.7 Hydrogen Atom Coordinates (Å×10^4) and Isotropic Displacement Parameters (Å^2×10^3) for MP825F19_twin1_hklf5.

Atom	*x*	*y*	*z*	U(eq)
H3BA	2338.12	6525.17	10011.86	24
H3BB	3642.66	7987.3	10187.92	24
H4BA	1584.89	9491.3	8954.2	24
H4BB	283.15	8026.74	8779.93	24
H6BA	8591.31	10018.54	6789.21	52
H6BB	7993.52	8594.3	7431.2	52
H6BC	8225.53	9993.6	8127.8	52
H7BA	6421.72	8940.39	5357.66	46
H7BB	4776.48	8574.75	5807.6	46
H7BC	5741.77	7626.89	6129.13	46
H8BA	6971.64	11394.26	6453.66	42
H8BB	6368.02	11305.49	7734.35	42
H8BC	5241.5	10781.37	6667.38	42
H9BA	-1367.16	5960.68	6138.1	44
H9BB	-919.05	6411.44	7450.64	44
H9BC	-627.77	5092.7	6919.66	44
H10A	472.03	8897.01	5516.56	40
H10B	2215.18	9428.47	5743.76	40
H10C	1127.77	9319.47	6792.2	40
H11A	1024.31	6451.32	4319.23	34
H11B	1566.93	5401.43	5049.55	34
H11C	2705.28	6862.04	4748.61	34
H13A	3401.2	2175.9	6137.76	38
H13B	3892.41	3420.64	5270.78	38
H13C	2255.57	2920.73	5750.29	38
H14A	5664.26	3576.06	7402.97	42

Table A.7 Hydrogen Atom Coordinates (Å×10[4]) and Isotropic Displacement Parameters (Å[2]×10[3]) for MP825F19_twin1_hklf5.

Atom	*x*	*y*	*z*	U(eq)
H14B	5778.68	5100.23	7773.06	42
H14C	5970.41	4735	6456.22	42
H16A	3731.19	2191.89	8694.88	38
H16B	2917.4	2785.84	9654.85	38
H16C	4512.03	3686.09	9237.77	38
H17A	1323.65	1781.94	7530.24	35
H17B	828.09	3067.31	7214.16	35
H17C	720.52	2548.52	8509.35	35
H3AA	9199.07	1005.33	3928.52	29
H3AB	10417.64	2464.72	3627.8	29
H4AA	8762.77	4216.72	4701.23	26
H4AB	7369.67	2942.17	5115.17	26
H6AA	3612.23	581.16	756.97	36
H6AB	5294.07	789	1090.48	36
H6AC	4664.02	2031.21	1236.85	36
H7AA	3149.26	-1482.72	2243.21	41
H7AB	3696.11	-1064.75	3544.43	41
H7AC	4871.26	-963.68	2562.08	41
H8AA	1732.57	208.86	2341.98	53
H8AB	2603.38	1772.75	2667.66	53
H8AC	2335.13	634.7	3630.19	53
H9AA	9678.02	935.66	403.93	41
H9AB	8046.63	312.57	879.51	41
H9AC	9423.78	804.56	1759.58	41
H10D	8482.4	2894.97	-933.98	47
H10E	7852.84	3933.35	-257.51	47
H10F	6923.96	2341.58	-325.19	47
H11D	11258.26	4109.21	565.65	49
H11E	11220.79	3860.76	1921.36	49
H11F	10562.76	4965.76	1399.82	49
H13D	7965.4	7614.12	629.32	50

Table A.7 Hydrogen Atom Coordinates (Å×10^4) and Isotropic Displacement Parameters (Å^2×10^3) for MP825F19_twin1_hklf5.

Atom	*x*	*y*	*z*	U(eq)
H13E	7388.73	6233.93	-127.47	50
H13F	8703.74	6449.18	793.86	50
H14D	5326.94	7167.17	1352.71	42
H14E	4455.6	5670.96	1793.49	42
H14F	4906.82	5889.52	474.28	42
H16D	5599.97	6995.83	3532.14	63
H16E	6432.21	6365.81	4458.75	63
H16F	5260.15	5380.3	3570.94	63
H17D	7994.62	8486.56	2831.1	80
H17E	9208.04	7853.71	2392.29	80
H17F	8867.13	7866.76	3733.88	80

Crystal Data for $C_{34}H_{68}B_2N_2O_8S_2Si_2$ (*M* =774.82 g/mol): triclinic, space group P1 (no. 1), *a* = 9.6138(2) Å, *b* = 10.3698(3) Å, *c* = 11.6300(4) Å, *α* = 90.818(3)°, *β* = 90.938(3)°, *γ* = 109.028(2)°, *V* = 1095.70(6) Å^3, *Z* = 1, *T* = 100(1) K, μ(Mo Kα) = 0.222 mm^{-1}, *Dcalc* = 1.174 g/cm^3, 23233 reflections measured (5.022° ≤ 2Θ ≤ 63.094°), 23233 unique (R_{int} = ?, R_{sigma} = 0.0143) which were used in all calculations. The final R_1 was 0.0372 (I > 2σ(I)) and wR_2 was 0.1056 (all data).

For product (*S*S*,2*S,3*S**)-8** CCDC-2456376

code(mo_MP825F17_0m)

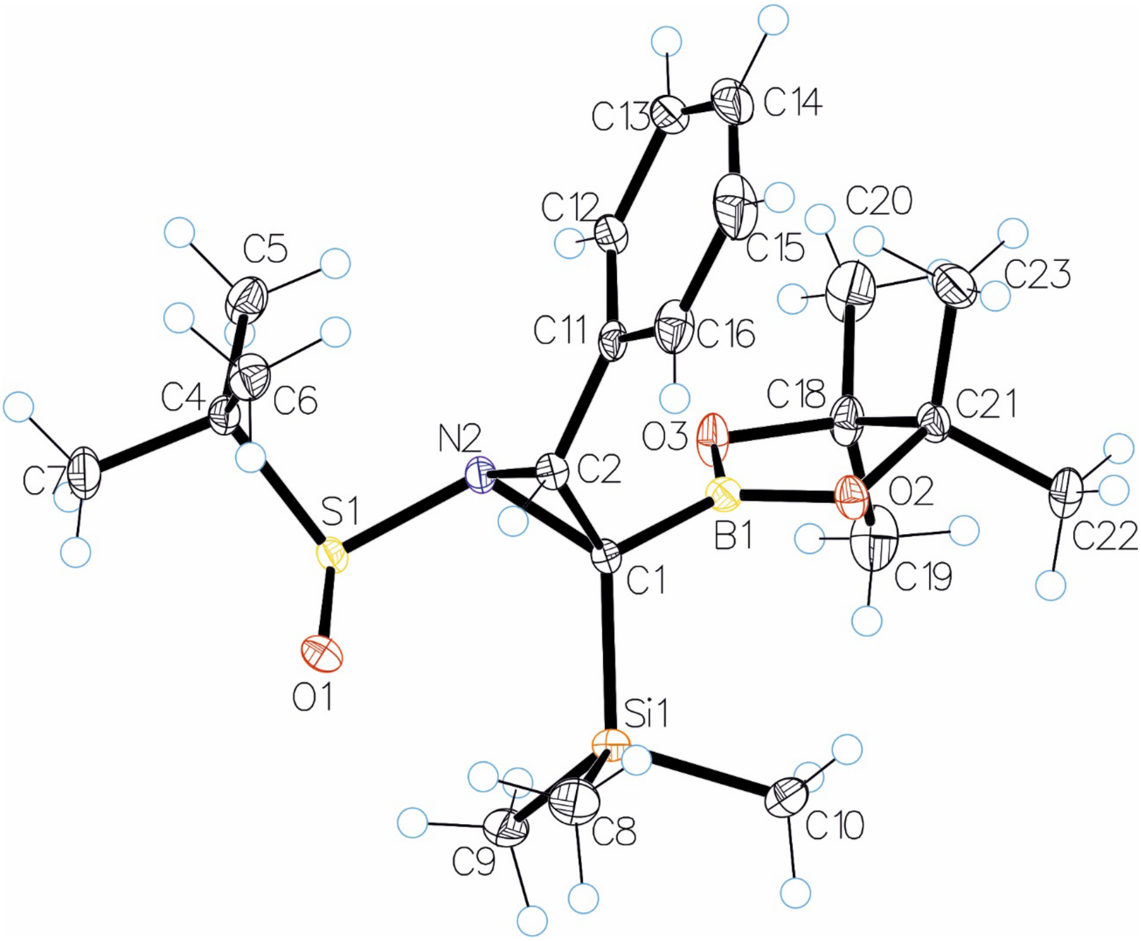

The asymmetric unit contains one molecule of the organic compound. The absolute structure could be determined reliable with a Flack value of -0.014(76) and Flack value based on Parsons' quotients of 0.021(39) (references: Flack H.D., Acta Cryst. A39 (1983) 876, Parsons S., Flack H., Acta Cryst. A39 (2004) S61, Parson, Flack and Wagner, Acta Cryst. B69 (2013) 249-259; Flack X determined using 2649 quotients [(I+)-(I-)]/[(I+)+(I-)]). The Flack (Parsons) parameter value for the correct absolute structure determination should be 0; the inverted structure would give 1; always taking in account the standard deviation. Be aware that the absolute configuration was calculated and interpreted based on the one crystal measured, if the ee% is < 98 the determination of the absolute configuration could be wrong. The absolute configuration based on the absolute structure of the measured crystals was determined with *R*(C1), *R*(C2). Be aware that I determined the absolute configuration manually using the Cahn-Ingold-Prelog rules, you should check the absolute configuration assigned. The structure is of excellent quality (no A- or B-alerts) and publishable with a R1 value of 4.12 %.

Table B.1 Crystal data and structure refinement for mo_MP825F17_0m.

Identification code	mo_MP825F17_0m
Empirical formula	$C_{21}H_{36}BNO_3SSi$
Formula weight	421.47
Temperature/K	99.78
Crystal system	monoclinic
Space group	$P2_1$
a/Å	10.4429(6)
b/Å	10.0669(6)
c/Å	11.4652(6)
α/°	90
β/°	92.4680(18)
γ/°	90
Volume/Å^3	1204.19(12)
Z	2
ρ_{calc}g/cm^3	1.162
μ/mm^{-1}	0.204
F(000)	456.0
Crystal size/mm^3	0.1 × 0.1 × 0.1
Radiation	MoKα (λ = 0.71073)
2Θ range for data collection/°	3.904 to 63.152
Index ranges	-15 ≤ h ≤ 15, -14 ≤ k ≤ 14, -12 ≤ l ≤ 16
Reflections collected	17686
Independent reflections	7896 [R_{int} = 0.0430, R_{sigma} = 0.0638]
Data/restraints/parameters	7896/1/266
Goodness-of-fit on F^2	1.020
Final R indexes [I>=2σ (I)]	R_1 = 0.0412, wR_2 = 0.0868
Final R indexes [all data]	R_1 = 0.0529, wR_2 = 0.0928
Largest diff. peak/hole / e Å^{-3}	0.38/-0.25

Table B.2 Fractional Atomic Coordinates (×10^4) and Equivalent Isotropic Displacement Parameters ($Å^2×10^3$) for mo_MP825F17_0m. U_{eq} is defined as 1/3 of the trace of the orthogonalised U_{IJ} tensor.

Atom	*x*	*y*	*z*	U(eq)
S1	9527.3(4)	6387.4(6)	4299.2(4)	14.45(11)
Si1	8958.9(5)	6551.7(7)	1337.0(5)	15.84(13)
O1	9903.4(15)	7677.1(19)	3778.9(14)	21.9(4)
O2	5986.1(15)	4901.9(17)	1157.4(14)	15.1(3)
O3	7002.7(16)	3491.2(17)	2452.3(14)	19.0(3)
N2	8055.6(16)	5907(2)	3761.5(15)	13.0(3)
C1	7785.0(19)	5935(2)	2455.2(18)	12.4(4)
C7	10397(2)	7099(3)	6418(2)	27.7(6)
C8	9012(3)	8400(3)	1225(2)	27.1(6)
C9	10575(2)	5786(3)	1598(2)	25.7(6)
C10	8341(2)	5939(3)	-126(2)	23.3(5)
C11	5821.7(19)	6807(2)	3609.1(18)	14.5(4)
C12	5399(2)	5734(3)	4248.2(19)	18.0(5)
C13	4122(2)	5642(3)	4534(2)	27.6(6)
C14	3258(2)	6606(4)	4164(2)	33.3(7)
C15	3662(2)	7671(3)	3524(3)	34.3(7)
C16	4952(2)	7787(3)	3253(2)	22.3(5)
C18	6216(2)	2644(3)	1674.6(19)	18.9(5)
C19	7128(3)	1997(3)	839(2)	28.5(6)
C20	5577(3)	1598(3)	2402(2)	34.5(7)
C21	5277(2)	3656(2)	1059.1(19)	15.4(4)
C22	4981(2)	3374(3)	-227(2)	21.3(5)
C23	4035(2)	3863(3)	1684(2)	26.8(6)
B1	6906(2)	4749(3)	2039(2)	13.0(5)
C2	7185(2)	6924(2)	3276.9(18)	12.9(4)
C4	9096(2)	6776(2)	5807.8(18)	15.6(4)

Table B.2 Fractional Atomic Coordinates (×10^4) and Equivalent Isotropic Displacement Parameters (Å^2×10^3) for mo_MP825F17_0m. U_{eq} is defined as 1/3 of the trace of the orthogonalised U_{IJ} tensor.

Atom	*x*	*y*	*z*	U(eq)
C5	8499(2)	5555(3)	6331(2)	22.3(5)
C6	8221(2)	7985(3)	5825(2)	20.3(5)

Table B.3 Anisotropic Displacement Parameters (Å^2×10^3) for mo_MP825F17_0m. The Anisotropic displacement factor exponent takes the form: -2π^2[h^{2}a*^{2}U$_{11}$+2hka*b*U$_{12}$+…].

Atom	U_{11}	U_{22}	U_{33}	U_{23}	U_{13}	U_{12}
S1	9.13(19)	19.8(3)	14.3(2)	-3.1(2)	-0.64(15)	-0.9(2)
Si1	13.4(2)	20.8(3)	13.5(3)	2.0(3)	2.31(19)	-2.0(3)
O1	18.6(8)	27.0(10)	20.1(8)	0.9(7)	2.0(6)	-11.2(7)
O2	16.5(7)	11.1(8)	17.2(7)	-0.8(6)	-3.7(6)	-2.0(6)
O3	24.4(8)	13.2(8)	18.4(8)	0.2(7)	-9.5(6)	-2.3(7)
N2	11.1(7)	15.4(9)	12.4(8)	-2.0(7)	-1.1(6)	0.2(7)
C1	10.5(8)	14.4(10)	12.3(9)	-0.9(8)	-1.1(7)	-1.3(8)
C7	18.2(11)	44.0(18)	20.6(12)	-11.4(11)	-5.1(9)	-0.4(11)
C8	32.3(13)	23.0(14)	25.9(13)	6.1(11)	1.0(10)	-8.6(11)
C9	14.9(10)	42.9(17)	19.7(11)	0.4(11)	3.7(8)	1.4(11)
C10	21.0(11)	34.4(15)	14.7(10)	1.2(10)	2.5(8)	-0.6(10)
C11	12.4(9)	18.3(12)	12.4(9)	-7.0(8)	-2.0(7)	1.8(8)
C12	12.9(9)	24.6(13)	16.2(10)	-3.9(9)	-0.9(8)	-3.2(9)
C13	18.1(11)	45.9(19)	19.2(11)	-9.5(12)	3.9(9)	-12.9(11)
C14	12.4(10)	58(2)	29.3(12)	-22.1(15)	3.6(9)	-0.4(13)
C15	19.9(12)	46(2)	36.5(15)	-16.7(14)	-9.0(10)	17.6(13)
C16	20.8(11)	24.1(14)	21.6(11)	-5.3(10)	-4.9(9)	7.3(10)
C18	25.2(11)	13.4(12)	17.3(10)	-0.7(9)	-6.3(8)	-3.6(9)
C19	33.9(14)	18.8(13)	32.0(14)	-8.2(11)	-7.1(11)	8.9(11)
C20	52.8(17)	20.0(15)	29.7(13)	7.2(12)	-9.7(12)	-15.0(14)

Table B.3 Anisotropic Displacement Parameters (Å^2×10^3) for mo_MP825F17_0m. The Anisotropic displacement factor exponent takes the form: -2π^2[h^{2}a*^{2}U$_{11}$+2hka*b*U$_{12}$+…].

Atom	U_{11}	U_{22}	U_{33}	U_{23}	U_{13}	U_{12}
C21	15.6(9)	14.5(11)	15.9(10)	-1.3(8)	-1.8(8)	-3.5(8)
C22	23.6(11)	23.8(13)	15.9(11)	-1.9(10)	-6.0(9)	-1.8(10)
C23	18.2(11)	34.5(16)	27.9(13)	-11.8(12)	4.4(9)	-7.0(11)
B1	12.6(10)	12.7(12)	13.8(10)	-2.1(9)	2.0(8)	0.4(9)
C2	12.5(9)	12.9(10)	13.3(9)	-0.3(8)	-0.8(7)	-0.4(8)
C4	13.4(9)	19.9(12)	13.3(9)	-4.2(8)	-1.1(7)	-0.6(8)
C5	28.8(12)	21.3(14)	16.5(11)	2.2(9)	-2.5(9)	-1.8(10)
C6	21.3(11)	19.5(13)	20.1(11)	-6.8(9)	0.6(9)	1.9(9)

Table B.4 Bond Lengths for mo_MP825F17_0m.

Atom	Atom	Length/Å	Atom	Atom	Length/Å
S1	O1	1.4886(19)	C7	C4	1.535(3)
S1	N2	1.7012(18)	C11	C12	1.387(3)
S1	C4	1.848(2)	C11	C16	1.391(3)
Si1	C1	1.915(2)	C11	C2	1.494(3)
Si1	C8	1.866(3)	C12	C13	1.390(3)
Si1	C9	1.867(3)	C13	C14	1.379(4)
Si1	C10	1.876(2)	C14	C15	1.376(5)
O2	C21	1.458(3)	C15	C16	1.401(4)
O2	B1	1.373(3)	C18	C19	1.526(4)
O3	C18	1.461(3)	C18	C20	1.517(4)
O3	B1	1.354(3)	C18	C21	1.561(3)
N2	C1	1.512(3)	C21	C22	1.521(3)
N2	C2	1.462(3)	C21	C23	1.522(3)
C1	B1	1.568(3)	C4	C5	1.515(3)
C1	C2	1.524(3)	C4	C6	1.522(3)

Table B.5 Bond Angles for mo_MP825F17_0m.

Atom	Atom	Atom	Angle/˚	Atom	Atom	Atom	Angle/˚
O1	S1	N2	110.62(10)	C14	C15	C16	120.2(3)
O1	S1	C4	105.63(10)	C11	C16	C15	119.8(3)
N2	S1	C4	98.42(9)	O3	C18	C19	106.42(19)
C8	Si1	C1	113.03(11)	O3	C18	C20	108.60(19)
C8	Si1	C9	113.17(13)	O3	C18	C21	102.77(19)
C8	Si1	C10	106.06(13)	C19	C18	C21	113.07(19)
C9	Si1	C1	111.04(11)	C20	C18	C19	110.5(2)
C9	Si1	C10	106.32(12)	C20	C18	C21	114.8(2)
C10	Si1	C1	106.64(10)	O2	C21	C18	102.66(16)
B1	O2	C21	107.28(17)	O2	C21	C22	108.47(19)
B1	O3	C18	107.47(18)	O2	C21	C23	106.62(19)
C1	N2	S1	118.76(13)	C22	C21	C18	114.4(2)
C2	N2	S1	118.42(15)	C22	C21	C23	109.92(19)
C2	N2	C1	61.60(14)	C23	C21	C18	114.1(2)
N2	C1	Si1	124.83(14)	O2	B1	C1	121.4(2)
N2	C1	B1	111.69(17)	O3	B1	O2	113.7(2)
N2	C1	C2	57.58(13)	O3	B1	C1	124.8(2)
B1	C1	Si1	115.25(14)	N2	C2	C1	60.82(13)
C2	C1	Si1	119.60(16)	N2	C2	C11	115.55(19)
C2	C1	B1	115.78(17)	C11	C2	C1	121.83(19)
C12	C11	C16	119.3(2)	C7	C4	S1	102.97(15)
C12	C11	C2	121.6(2)	C5	C4	S1	108.67(16)
C16	C11	C2	119.1(2)	C5	C4	C7	111.2(2)
C11	C12	C13	120.5(2)	C5	C4	C6	112.73(19)
C14	C13	C12	120.1(3)	C6	C4	S1	110.63(16)
C15	C14	C13	120.1(2)	C6	C4	C7	110.2(2)

Table B.6 Torsion Angles for mo_MP825F17_0m.

A	B	C	D	Angle/˚	A	B	C	D	Angle/˚
S1	N2	C1	Si1	-3.0(3)	C14	C15	C16	C11	1.5(4)
S1	N2	C1	B1	143.74(16)	C16	C11	C12	C13	-0.2(3)
S1	N2	C1	C2	-108.70(18)	C16	C11	C2	N2	175.5(2)
S1	N2	C2	C1	109.22(16)	C16	C11	C2	C1	-114.4(2)
S1	N2	C2	C11	-137.04(16)	C18	O3	B1	O2	9.2(3)
Si1	C1	B1	O2	-66.8(2)	C18	O3	B1	C1	-167.8(2)
Si1	C1	B1	O3	110.0(2)	C19	C18	C21	O2	-90.1(2)
Si1	C1	C2	N2	-114.67(17)	C19	C18	C21	C22	27.2(3)
Si1	C1	C2	C11	141.76(18)	C19	C18	C21	C23	155.0(2)
O1	S1	N2	C1	48.75(19)	C20	C18	C21	O2	141.9(2)
O1	S1	N2	C2	-22.60(18)	C20	C18	C21	C22	-100.8(2)
O1	S1	C4	C7	-70.99(19)	C20	C18	C21	C23	27.0(3)
O1	S1	C4	C5	170.95(15)	C21	O2	B1	O3	7.7(2)
O1	S1	C4	C6	46.69(18)	C21	O2	B1	C1	-175.21(19)
O3	C18	C21	O2	24.2(2)	B1	O2	C21	C18	-19.7(2)
O3	C18	C21	C22	141.51(19)	B1	O2	C21	C22	-141.17(19)
O3	C18	C21	C23	-90.7(2)	B1	O2	C21	C23	100.5(2)
N2	S1	C4	C7	174.73(18)	B1	O3	C18	C19	98.4(2)
N2	S1	C4	C5	56.68(17)	B1	O3	C18	C20	-142.6(2)
N2	S1	C4	C6	-67.59(17)	B1	O3	C18	C21	-20.6(2)
N2	C1	B1	O2	143.04(19)	B1	C1	C2	N2	100.3(2)
N2	C1	B1	O3	-40.2(3)	B1	C1	C2	C11	-3.2(3)
N2	C1	C2	C11	-103.6(2)	C2	N2	C1	Si1	105.7(2)
C1	N2	C2	C11	113.7(2)	C2	N2	C1	B1	-107.57(19)
C11	C12	C13	C14	1.2(4)	C2	C1	B1	O2	79.7(3)
C12	C11	C16	C15	-1.1(3)	C2	C1	B1	O3	-103.5(3)
C12	C11	C2	N2	-5.5(3)	C2	C11	C12	C13	-179.2(2)
C12	C11	C2	C1	64.6(3)	C2	C11	C16	C15	177.9(2)

Table B.6 Torsion Angles for mo_MP825F17_0m.

A	B	C	D	Angle/˚	A	B	C	D	Angle/˚
C12	C13	C14	C15	-0.9(4)	C4	S1	N2	C1	159.04(17)
C13	C14	C15	C16	-0.5(4)	C4	S1	N2	C2	87.69(16)

Table B.7 Hydrogen Atom Coordinates (Å×10^4) and Isotropic Displacement Parameters (Å^2×10^3) for mo_MP825F17_0m.

Atom	*x*	*y*	*z*	U(eq)
H7A	10950.81	6313.74	6403.01	42
H7B	10268.32	7354.79	7228.91	42
H7C	10801.08	7833.21	6010.13	42
H8A	9586.32	8656.2	609.91	41
H8B	9328.38	8774.09	1971.84	41
H8C	8148.48	8738.92	1034.2	41
H9A	11064.2	5866.88	890.84	39
H9B	10476.66	4844.78	1793.56	39
H9C	11030.79	6243.93	2245.17	39
H10A	8877.19	6289.74	-735.37	35
H10B	7455.48	6240.03	-268.99	35
H10C	8366.38	4965.54	-138.65	35
H12	5986.62	5058.67	4492.23	22
H13	3843.72	4913.2	4985.25	33
H14	2383.72	6533.7	4350.59	40
H15	3063.36	8329.76	3265.18	41
H16	5232.96	8534.64	2826.95	27
H19A	6639.82	1423.23	290.87	43
H19B	7762.61	1463.25	1285.25	43
H19C	7565.86	2687.41	405.22	43
H20A	4990.11	1068.07	1900.21	52
H20B	5094.79	2031.44	3010.54	52

Table B.7 Hydrogen Atom Coordinates (Å×10^4) and Isotropic Displacement Parameters (Å^2×10^3) for mo_MP825F17_0m.

Atom	*x*	*y*	*z*	U(eq)
H20C	6232.14	1016.62	2766.76	52
H22A	4395.29	4053.42	-550.22	32
H22B	4580	2497.23	-312.76	32
H22C	5778.57	3388.66	-647.94	32
H23A	3568.1	4618.76	1334.98	40
H23B	4232.97	4041.91	2512.45	40
H23C	3506.07	3061.36	1606.72	40
H2	7530(30)	7810(30)	3240(20)	19
H5A	7654.44	5396.05	5950.18	33
H5B	8403.4	5693.82	7169.28	33
H5C	9051.27	4783.77	6213.75	33
H6A	7366.65	7742.78	5508.27	30
H6B	8574.99	8693.11	5348.35	30
H6C	8159.5	8298.24	6630.19	30

Crystal Data for $C_{21}H_{36}BNO_3SSi$ (*M* =421.47 g/mol): monoclinic, space group $P2_1$ (no. 4), *a* = 10.4429(6) Å, *b* = 10.0669(6) Å, *c* = 11.4652(6) Å, *β* = 92.4680(18)°, *V* = 1204.19(12) Å^3, *Z* = 2, *T* = 99.78 K, μ(MoKα) = 0.204 mm^{-1}, *Dcalc* = 1.162 g/cm^3, 17686 reflections measured (3.904° ≤ 2Θ ≤ 63.152°), 7896 unique (R_{int} = 0.0430, R_{sigma} = 0.0638) which were used in all calculations. The final R_1 was 0.0412 (I > 2σ(I)) and wR_2 was 0.0928 (all data).

For product (S_S^*,2R^*,3S^*)-28 CCDC-2471995

Code(mo_MP902_0m_a)

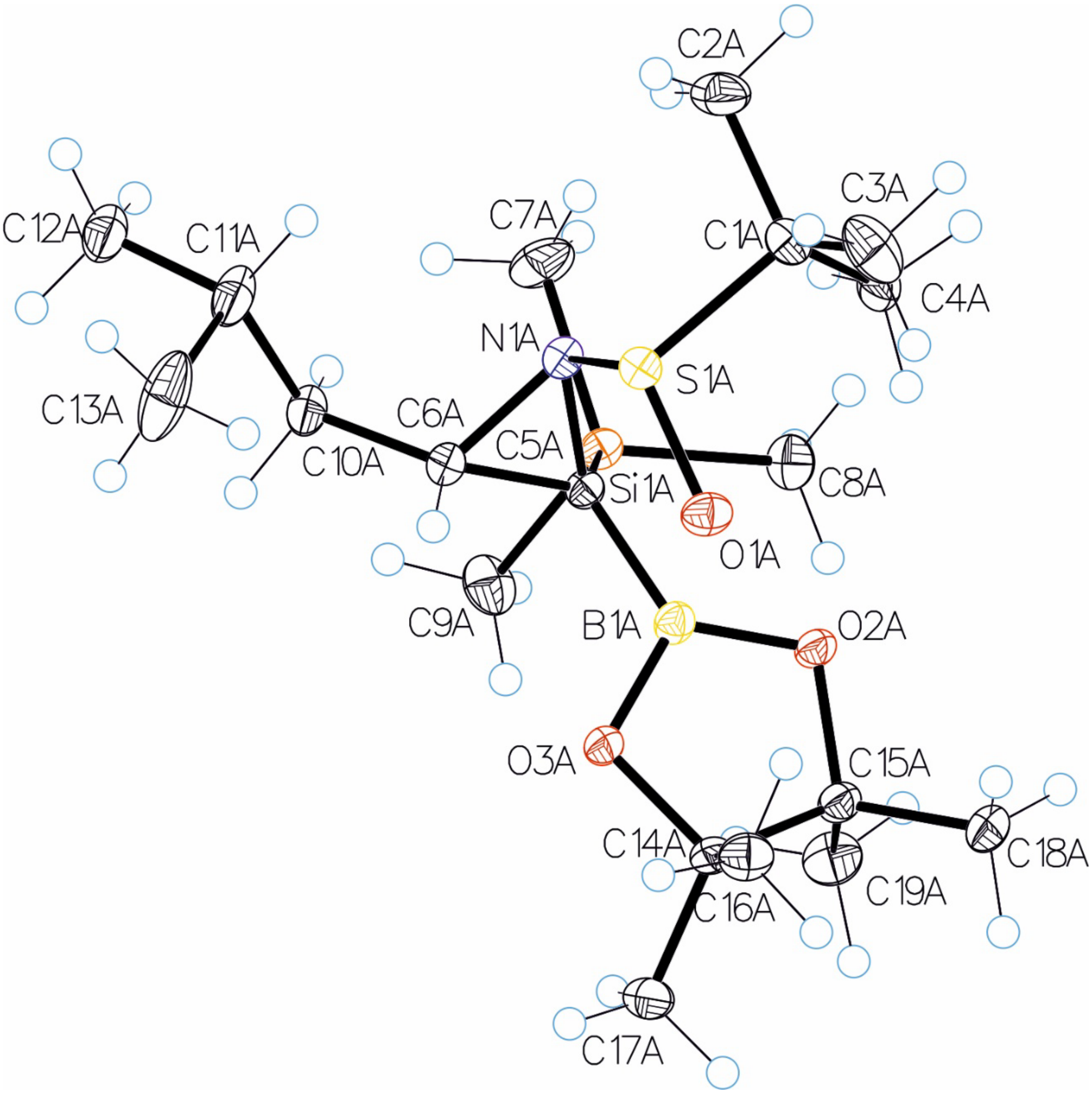

The asymmetric unit contains two independent molecules of the same compound, all exhibiting identical stereochemistry. The absolute structure was reliably determined, with a Flack value of 0.001(38) and a Flack value based on Parsons' quotients of -0.007(15) (References: Flack H.D., *Acta Cryst.* A39 (1983) 876; Parsons S., Flack H., *Acta Cryst.* A39 (2004) S61; Parsons, Flack and Wagner, *Acta Cryst.* B69 (2013) 249–259). The Flack X parameter was determined using 6192 quotients of $[(I^+)-(I^-)]/[(I^+)+(I^-)]$. The expected value for the Flack (Parsons) parameter for the correct absolute structure is 0; the inverted structure would yield 1, always considering the standard deviation. Please

note that the absolute configuration was determined based on a single crystal measured; if the enantiomeric excess (ee%) is below 98%, the assignment could be incorrect. Based on the measured crystal, the absolute configuration was determined as *R*(C5), and *S*(C6), and was identical for all two independent molecules (A and B). This configuration was assigned manually using the Cahn–Ingold–Prelog rules; you are advised to double-check the assigned configuration. The structure is of excellent quality (no A- or B-level alerts) and is suitable for publication, with an R1 value of 3.09 %.

Table C.1 Crystal data and structure refinement for mo_MP902_0m_a.

Identification code	mo_MP902_0m_a
Empirical formula	$C_{38}H_{80}B_2N_2O_6S_2Si_2$
Formula weight	802.96
Temperature/K	100(2)
Crystal system	monoclinic
Space group	$P2_1$
a/Å	10.7909(8)
b/Å	22.3729(17)
c/Å	10.8729(8)
α/°	90
β/°	111.262(2)
γ/°	90
Volume/Å^3	2446.3(3)
Z	2
ρ_{calc}g/cm^3	1.090
μ/mm^{-1}	0.198
F(000)	880.0
Crystal size/mm^3	0.2 × 0.2 × 0.15
Radiation	MoKα (λ = 0.71073)
2Θ range for data collection/°	3.64 to 63.3
Index ranges	-15 ≤ h ≤ 14, -32 ≤ k ≤ 30, -15 ≤ l ≤ 16
Reflections collected	47255
Independent reflections	15526 [R_{int} = 0.0277, R_{sigma} = 0.0337]
Data/restraints/parameters	15526/1/504
Goodness-of-fit on F^2	1.034

Final R indexes [I>=2σ (I)]	R_1 = 0.0309, wR_2 = 0.0745
Final R indexes [all data]	R_1 = 0.0344, wR_2 = 0.0764
Largest diff. peak/hole / e Å^{-3}	0.31/-0.20

Table C.2 Fractional Atomic Coordinates (×10^4) and Equivalent Isotropic Displacement Parameters (Å^2×10^3) for mo_MP902_0m_a. U_{eq} is defined as 1/3 of the trace of the orthogonalised U_{IJ} tensor.

Atom	*x*	*y*	*z*	U(eq)
S1A	5388.1(4)	4685.7(2)	5959.4(3)	14.68(7)
Si1A	3521.5(4)	3109.1(2)	3698.8(4)	14.83(8)
O1A	6608.9(12)	4524.3(6)	5675.6(12)	19.9(2)
O2A	6374.0(11)	3720.8(5)	3581.7(11)	16.4(2)
O3A	7348.9(11)	3298.6(5)	5627.7(11)	16.7(2)
N1A	4363.1(14)	4077.1(6)	5564.7(12)	14.5(2)
C1A	4405.2(17)	5187.4(7)	4630.4(15)	17.9(3)
C2A	3126(2)	5325.3(9)	4873.0(18)	26.5(4)
C3A	5279(2)	5743.4(9)	4831(2)	33.3(4)
C4A	4129.5(19)	4903.2(8)	3289.2(15)	22.2(3)
C5A	4873.9(16)	3555.8(7)	4984.3(14)	13.7(3)
C6A	4992.7(17)	3560.8(7)	6411.8(14)	15.5(3)
C7A	1832.1(19)	3279.7(11)	3713(2)	30.3(4)
C8A	3445.7(18)	3281.5(8)	1997.7(15)	21.5(3)
C9A	3955(2)	2306.1(8)	4083.9(18)	26.7(4)
C10A	4134.4(18)	3218.1(7)	7001.1(16)	19.5(3)
C11A	3689(2)	3586.1(9)	7943.2(19)	27.5(4)
C12A	2697(2)	3244.9(10)	8370.3(18)	28.3(4)
C14A	8397.1(16)	3339.8(7)	5085.1(15)	16.6(3)
C15A	7592.4(17)	3442.8(7)	3580.4(15)	16.7(3)
C16A	9287.6(18)	3864.8(8)	5751.5(17)	22.6(3)
C17A	9182.4(19)	2760.0(8)	5404.0(18)	23.8(3)
C18A	8240.2(19)	3858.0(9)	2888.0(17)	23.4(3)
C19A	7193(2)	2861.4(9)	2806.8(18)	26.7(4)

Table C.2 Fractional Atomic Coordinates (×10^4) and Equivalent Isotropic Displacement Parameters (Å^2×10^3) for mo_MP902_0m_a. U_{eq} is defined as 1/3 of the trace of the orthogonalised U_{IJ} tensor.

Atom	*x*	*y*	*z*	U(eq)
B1A	6255.6(18)	3596.9(8)	4775.1(16)	16.1(3)
C13A	5106(14)	3609(10)	9287(8)	42(3)
C13'	4688(10)	3888(5)	9022(9)	44(2)
S1B	9832.4(4)	6911.2(2)	9181.1(4)	16.85(8)
Si04	9809.7(5)	4982.4(2)	9838.6(4)	14.30(8)
O1B	8543.6(13)	6989.2(6)	9372.6(12)	22.2(2)
O2B	6597.2(12)	5978.3(6)	8665.5(10)	16.8(2)
O3B	7906.8(12)	5966.4(5)	10860.2(10)	16.4(2)
N1B	10054.8(14)	6172.2(6)	8952.4(12)	14.1(2)
C1B	11148.3(18)	7023.4(8)	10801.5(17)	21.3(3)
C2B	10902(2)	6642.4(9)	11844.5(18)	31.8(4)
C3B	11061(3)	7688.9(9)	11071(2)	34.1(5)
C4B	12471(2)	6881.5(13)	10656(3)	45.3(6)
C5B	9062.5(15)	5736.8(6)	9164.2(13)	12.6(3)
C6B	8947.8(16)	5931.7(7)	7803.3(14)	13.4(3)
C7B	10184.0(18)	4940.6(8)	11652.2(15)	21.3(3)
C8B	8526(2)	4410.8(8)	9012.3(18)	27.0(4)
C9B	11405(2)	4830.9(9)	9577.2(18)	28.2(4)
C10B	9248.9(16)	5556.6(7)	6798.4(14)	14.4(3)
C11B	9413(2)	5923.6(8)	5682.7(16)	22.2(3)
C12B	10678(3)	6291.7(13)	6168(3)	53.8(8)
C13B	9414(2)	5513.2(9)	4559.8(17)	27.6(4)
C14B	5723.7(17)	6148.2(8)	9368.6(15)	18.6(3)
C15B	6538.9(17)	5944.7(8)	10808.4(15)	18.5(3)
C16B	4393.0(19)	5839.2(11)	8720.9(19)	29.7(4)
C17B	5546(2)	6824.3(9)	9237.8(18)	25.9(4)
C18B	6269(2)	5297.9(9)	11072(2)	29.2(4)
C19B	6416(2)	6347.0(10)	11881.6(17)	28.0(4)
B1B	7868.6(18)	5952.2(8)	9582.4(16)	14.4(3)

Table C.3 Anisotropic Displacement Parameters (Å^2×10^3) for mo_MP902_0m_a. The Anisotropic displacement factor exponent takes the form: $-2\pi^2[h^2a^{*2}U_{11}+2hka^*b^*U_{12}+\ldots]$.

Atom	U_{11}	U_{22}	U_{33}	U_{23}	U_{13}	U_{12}
S1A	14.77(18)	14.19(16)	13.78(14)	-2.08(11)	3.61(12)	-1.45(13)
Si1A	13.0(2)	15.06(19)	16.62(18)	-1.80(14)	5.57(15)	-2.37(15)
O1A	13.7(6)	21.4(6)	23.9(5)	-1.3(4)	6.0(4)	-1.4(4)
O2A	12.0(5)	22.5(6)	15.6(5)	1.2(4)	5.8(4)	1.2(4)
O3A	13.0(5)	21.1(5)	17.1(5)	3.6(4)	6.7(4)	1.5(4)
N1A	15.4(7)	14.0(6)	14.9(5)	-1.9(4)	6.4(5)	-1.9(4)
C1A	19.3(8)	15.0(7)	17.2(6)	-0.6(5)	4.1(6)	-0.5(6)
C2A	24.6(10)	30.2(9)	23.5(8)	-3.5(6)	7.1(7)	9.7(7)
C3A	36.1(12)	17.8(8)	36.9(10)	4.7(7)	2.5(8)	-6.6(7)
C4A	27.8(9)	23.6(8)	15.3(6)	1.3(6)	8.2(6)	3.3(7)
C5A	13.4(7)	13.9(6)	14.2(6)	-0.2(5)	5.4(5)	-0.5(5)
C6A	16.1(7)	16.7(7)	14.8(6)	0.1(5)	6.8(5)	-0.8(5)
C7A	14.7(9)	43.3(11)	33.2(9)	-5.5(8)	9.1(7)	-2.6(8)
C8A	23.1(9)	23.4(8)	15.9(6)	-3.1(6)	4.4(6)	-4.0(6)
C9A	36.3(11)	15.1(7)	27.6(8)	-0.7(6)	10.4(8)	-2.7(7)
C10A	24.4(9)	18.5(7)	18.8(7)	0.4(5)	11.7(6)	-2.2(6)
C11A	42.2(12)	21.9(8)	28.6(8)	-1.7(6)	25.3(8)	-3.9(8)
C12A	29.2(10)	37.6(11)	22.4(8)	4.1(7)	14.5(7)	0.3(8)
C14A	11.7(7)	18.7(7)	19.9(6)	1.3(5)	6.6(5)	0.8(5)
C15A	14.8(8)	18.1(7)	18.8(6)	-0.1(5)	7.9(6)	-0.2(5)
C16A	16.3(8)	24.2(8)	25.4(8)	-2.1(6)	5.3(6)	-2.3(6)
C17A	20.0(9)	22.0(8)	31.0(8)	5.9(6)	11.3(7)	5.6(6)
C18A	19.5(9)	29.4(9)	25.2(8)	6.0(6)	12.9(7)	1.3(7)
C19A	28.2(10)	25.3(9)	26.6(8)	-7.0(7)	10.0(7)	-1.0(7)
B1A	13.4(8)	19.4(8)	15.4(7)	0.5(6)	5.2(6)	-1.6(6)
C13A	42(5)	69(8)	18(3)	-11(4)	14(3)	-30(5)
C13'	55(4)	55(4)	33(3)	-22(3)	28(3)	-29(4)
S1B	21.9(2)	10.97(15)	18.54(16)	-1.72(12)	8.39(14)	-1.36(13)
Si04	16.8(2)	12.39(18)	13.10(16)	1.13(13)	4.66(15)	1.98(15)
O1B	22.0(6)	18.5(6)	25.4(6)	-2.7(4)	7.7(5)	4.5(5)
O2B	15.0(6)	23.4(6)	13.2(4)	-1.1(4)	6.5(4)	1.9(4)

Table C.3 Anisotropic Displacement Parameters (Å^2×10^3) for mo_MP902_0m_a. The Anisotropic displacement factor exponent takes the form: $-2\pi^2[h^2a^{*2}U_{11}+2hka^*b^*U_{12}+\ldots]$.

Atom	U_{11}	U_{22}	U_{33}	U_{23}	U_{13}	U_{12}
O3B	15.6(6)	22.2(5)	12.8(4)	1.3(4)	6.6(4)	3.3(4)
N1B	16.8(7)	10.5(5)	15.4(5)	-1.8(4)	6.2(5)	-2.1(4)
C1B	20.2(8)	18.0(8)	24.0(7)	-8.3(6)	6.0(6)	-3.7(6)
C2B	40.6(12)	25.0(9)	20.0(8)	-2.2(6)	-0.9(7)	-6.9(8)
C3B	49.8(14)	19.4(9)	32.9(9)	-11.4(7)	14.8(9)	-9.0(8)
C4B	21.7(11)	63.0(16)	50.5(13)	-31.8(12)	12.3(9)	-8.7(10)
C5B	14.1(7)	11.4(6)	12.3(6)	-0.4(4)	4.9(5)	-0.4(5)
C6B	14.5(7)	13.3(6)	12.7(6)	-0.4(5)	5.3(5)	-0.4(5)
C7B	25.0(9)	21.9(8)	14.8(6)	4.8(6)	4.8(6)	2.6(6)
C8B	35.9(11)	14.8(8)	24.4(8)	-1.2(6)	3.8(7)	-5.0(7)
C9B	25.3(10)	34.6(10)	27.6(8)	8.9(7)	13.2(7)	15.7(7)
C10B	16.8(7)	14.0(6)	12.8(6)	-1.4(5)	5.7(5)	-1.3(5)
C11B	35.4(10)	18.5(7)	17.3(7)	2.7(5)	15.1(7)	2.8(7)
C12B	84(2)	47.5(14)	53.0(14)	-22.5(12)	52.5(16)	-43.4(15)
C13B	39.5(11)	29.0(9)	18.3(7)	0.2(6)	15.1(7)	4.5(8)
C14B	15.9(8)	26.2(8)	15.0(6)	-0.1(5)	7.4(6)	2.4(6)
C15B	19.9(8)	22.2(7)	16.2(6)	1.6(5)	10.0(6)	3.4(6)
C16B	18.5(9)	44.6(12)	27.6(8)	-7.4(8)	10.2(7)	-3.7(8)
C17B	29.3(10)	26.9(9)	24.3(8)	7.0(6)	13.2(7)	10.4(7)
C18B	32.1(11)	25.0(9)	35.6(10)	7.6(7)	18.3(8)	0.0(8)
C19B	30.8(10)	38.5(10)	17.9(7)	-0.8(7)	12.5(7)	9.9(8)
B1B	16.2(8)	14.1(7)	13.5(6)	0.8(5)	6.1(6)	1.5(6)

Table C.4 Bond Lengths for mo_MP902_0m_a.

Atom	Atom	Length/Å	Atom	Atom	Length/Å
S1A	O1A	1.5019(13)	S1B	O1B	1.4893(14)
S1A	N1A	1.7081(14)	S1B	N1B	1.7017(13)
S1A	C1A	1.8335(17)	S1B	C1B	1.8355(18)
Si1A	C5A	1.8982(16)	Si04	C5B	1.8995(15)
Si1A	C7A	1.868(2)	Si04	C7B	1.8661(16)
Si1A	C8A	1.8620(17)	Si04	C8B	1.8595(19)

Table C.4 Bond Lengths for mo_MP902_0m_a.

Atom	Atom	Length/Å	Atom	Atom	Length/Å
Si1A	C9A	1.8652(19)	Si04	C9B	1.874(2)
O2A	C15A	1.455(2)	O2B	C14B	1.4628(19)
O2A	B1A	1.377(2)	O2B	B1B	1.374(2)
O3A	C14A	1.4560(19)	O3B	C15B	1.457(2)
O3A	B1A	1.380(2)	O3B	B1B	1.3752(19)
N1A	C5A	1.521(2)	N1B	C5B	1.526(2)
N1A	C6A	1.480(2)	N1B	C6B	1.4815(19)
C1A	C2A	1.527(3)	C1B	C2B	1.517(3)
C1A	C3A	1.528(3)	C1B	C3B	1.527(3)
C1A	C4A	1.518(2)	C1B	C4B	1.525(3)
C5A	C6A	1.510(2)	C5B	C6B	1.5040(19)
C5A	B1A	1.590(2)	C5B	B1B	1.589(2)
C6A	C10A	1.512(2)	C6B	C10B	1.504(2)
C10A	C11A	1.522(2)	C10B	C11B	1.527(2)
C11A	C12A	1.518(3)	C11B	C12B	1.517(3)
C11A	C13A	1.690(12)	C11B	C13B	1.528(2)
C11A	C13'	1.440(6)	C14B	C15B	1.560(2)
C14A	C15A	1.566(2)	C14B	C16B	1.517(3)
C14A	C16A	1.524(2)	C14B	C17B	1.525(3)
C14A	C17A	1.519(2)	C15B	C18B	1.524(3)
C15A	C18A	1.517(2)	C15B	C19B	1.517(2)
C15A	C19A	1.524(2)			

Table C.5 Bond Angles for mo_MP902_0m_a.

Atom	Atom	Atom	Angle/˚	Atom	Atom	Atom	Angle/˚
O1A	S1A	N1A	107.04(7)	O3A	B1A	C5A	119.57(13)
O1A	S1A	C1A	106.57(8)	O1B	S1B	N1B	108.83(7)
N1A	S1A	C1A	98.81(7)	O1B	S1B	C1B	106.64(8)
C7A	Si1A	C5A	112.23(8)	N1B	S1B	C1B	99.92(7)
C8A	Si1A	C5A	111.56(7)	C7B	Si04	C5B	110.57(7)
C8A	Si1A	C7A	105.92(9)	C7B	Si04	C9B	107.00(8)
C8A	Si1A	C9A	109.86(8)	C8B	Si04	C5B	106.69(8)

Table C.5 Bond Angles for mo_MP902_0m_a.

Atom	Atom	Atom	Angle/˚	Atom	Atom	Atom	Angle/˚
C9A	Si1A	C5A	106.17(8)	C8B	Si04	C7B	108.30(9)
C9A	Si1A	C7A	111.16(10)	C8B	Si04	C9B	111.49(10)
B1A	O2A	C15A	107.91(12)	C9B	Si04	C5B	112.75(8)
B1A	O3A	C14A	107.32(12)	B1B	O2B	C14B	107.15(12)
C5A	N1A	S1A	114.60(10)	B1B	O3B	C15B	107.54(12)
C6A	N1A	S1A	110.88(10)	C5B	N1B	S1B	117.02(10)
C6A	N1A	C5A	60.41(9)	C6B	N1B	S1B	111.27(10)
C2A	C1A	S1A	106.75(12)	C6B	N1B	C5B	60.00(9)
C2A	C1A	C3A	111.14(16)	C2B	C1B	S1B	110.88(13)
C3A	C1A	S1A	103.59(12)	C2B	C1B	C3B	111.41(16)
C4A	C1A	S1A	110.89(12)	C2B	C1B	C4B	112.61(19)
C4A	C1A	C2A	112.08(14)	C3B	C1B	S1B	103.73(14)
C4A	C1A	C3A	111.95(15)	C4B	C1B	S1B	107.43(13)
N1A	C5A	Si1A	114.30(11)	C4B	C1B	C3B	110.35(18)
N1A	C5A	B1A	120.97(13)	N1B	C5B	Si04	113.75(10)
C6A	C5A	Si1A	121.11(11)	N1B	C5B	B1B	122.41(12)
C6A	C5A	N1A	58.44(9)	C6B	C5B	Si04	120.62(10)
C6A	C5A	B1A	114.33(13)	C6B	C5B	N1B	58.55(9)
B1A	C5A	Si1A	115.82(10)	C6B	C5B	B1B	114.06(12)
N1A	C6A	C5A	61.15(10)	B1B	C5B	Si04	115.71(10)
N1A	C6A	C10A	116.48(14)	N1B	C6B	C5B	61.45(9)
C5A	C6A	C10A	126.51(14)	N1B	C6B	C10B	119.66(13)
C6A	C10A	C11A	113.73(14)	C10B	C6B	C5B	126.20(13)
C10A	C11A	C13A	101.0(6)	C6B	C10B	C11B	113.26(13)
C12A	C11A	C10A	111.33(16)	C10B	C11B	C13B	110.22(14)
C12A	C11A	C13A	105.5(4)	C12B	C11B	C10B	111.37(16)
C13'	C11A	C10A	118.4(4)	C12B	C11B	C13B	110.04(17)
C13'	C11A	C12A	112.9(3)	O2B	C14B	C15B	102.29(13)
O3A	C14A	C15A	102.40(12)	O2B	C14B	C16B	108.65(13)
O3A	C14A	C16A	107.74(13)	O2B	C14B	C17B	106.98(14)
O3A	C14A	C17A	107.73(13)	C16B	C14B	C15B	115.29(15)
C16A	C14A	C15A	113.47(13)	C16B	C14B	C17B	110.05(16)

Table C.5 Bond Angles for mo_MP902_0m_a.

Atom	Atom	Atom	Angle/˚	Atom	Atom	Atom	Angle/˚
C17A	C14A	C15A	114.59(14)	C17B	C14B	C15B	112.90(14)
C17A	C14A	C16A	110.25(14)	O3B	C15B	C14B	102.92(12)
O2A	C15A	C14A	102.75(12)	O3B	C15B	C18B	106.52(14)
O2A	C15A	C18A	108.53(13)	O3B	C15B	C19B	108.67(15)
O2A	C15A	C19A	106.90(14)	C18B	C15B	C14B	112.91(15)
C18A	C15A	C14A	115.33(14)	C19B	C15B	C14B	115.09(14)
C18A	C15A	C19A	109.74(14)	C19B	C15B	C18B	110.05(15)
C19A	C15A	C14A	112.94(14)	O2B	B1B	O3B	112.85(14)
O2A	B1A	O3A	112.43(14)	O2B	B1B	C5B	120.35(13)
O2A	B1A	C5A	123.95(14)	O3B	B1B	C5B	124.46(14)

Table C.6 Torsion Angles for mo_MP902_0m_a.

A	B	C	D	Angle/˚	A	B	C	D	Angle/˚
S1A	N1A	C5A	Si1A	-146.17(8)	S1B	N1B	C5B	Si04	-147.35(8)
S1A	N1A	C5A	C6A	101.01(12)	S1B	N1B	C5B	C6B	100.12(12)
S1A	N1A	C5A	B1A	-0.25(17)	S1B	N1B	C5B	B1B	-0.12(17)
S1A	N1A	C6A	C5A	-107.20(11)	S1B	N1B	C6B	C5B	-109.77(11)
S1A	N1A	C6A	C10A	133.83(12)	S1B	N1B	C6B	C10B	132.59(12)
Si1A	C5A	C6A	N1A	-101.16(13)	Si04	C5B	C6B	N1B	-100.75(12)
Si1A	C5A	C6A	C10A	1.9(2)	Si04	C5B	C6B	C10B	6.7(2)
Si1A	C5A	B1A	O2A	45.7(2)	Si04	C5B	B1B	O2B	-110.41(14)
Si1A	C5A	B1A	O3A	-109.93(14)	Si04	C5B	B1B	O3B	50.95(19)
O1A	S1A	N1A	C5A	-2.84(12)	O1B	S1B	N1B	C5B	-7.99(12)
O1A	S1A	N1A	C6A	63.17(11)	O1B	S1B	N1B	C6B	58.20(11)
O1A	S1A	C1A	C2A	176.94(11)	O1B	S1B	C1B	C2B	50.76(15)
O1A	S1A	C1A	C3A	-65.66(14)	O1B	S1B	C1B	C3B	-68.93(14)
O1A	S1A	C1A	C4A	54.60(14)	O1B	S1B	C1B	C4B	174.20(15)
O3A	C14A	C15A	O2A	26.06(15)	O2B	C14B	C15B	O3B	26.22(16)
O3A	C14A	C15A	C18A	143.94(14)	O2B	C14B	C15B	C18B	-88.22(17)
O3A	C14A	C15A	C19A	-88.74(16)	O2B	C14B	C15B	C19B	144.26(16)
N1A	S1A	C1A	C2A	66.12(12)	N1B	S1B	C1B	C2B	-62.45(15)
N1A	S1A	C1A	C3A	-176.48(13)	N1B	S1B	C1B	C3B	177.86(13)

Table C.6 Torsion Angles for mo_MP902_0m_a.

A	B	C	D	Angle/˚	A	B	C	D	Angle/˚
N1A	S1A	C1A	C4A	-56.22(13)	N1B	S1B	C1B	C4B	60.99(16)
N1A	C5A	C6A	C10A	103.04(18)	N1B	C5B	C6B	C10B	107.45(17)
N1A	C5A	B1A	O2A	-99.75(18)	N1B	C5B	B1B	O2B	102.95(17)
N1A	C5A	B1A	O3A	104.64(18)	N1B	C5B	B1B	O3B	-95.69(18)
N1A	C6A	C10A	C11A	-63.27(19)	N1B	C6B	C10B	C11B	-89.22(17)
C1A	S1A	N1A	C5A	107.60(11)	C1B	S1B	N1B	C5B	103.53(11)
C1A	S1A	N1A	C6A	173.62(10)	C1B	S1B	N1B	C6B	169.71(11)
C5A	N1A	C6A	C10A	-118.97(15)	C5B	N1B	C6B	C10B	-117.64(15)
C5A	C6A	C10A	C11A	-135.69(17)	C5B	C6B	C10B	C11B	-163.87(15)
C6A	N1A	C5A	Si1A	112.82(12)	C6B	N1B	C5B	Si04	112.53(11)
C6A	N1A	C5A	B1A	-101.26(15)	C6B	N1B	C5B	B1B	-100.24(14)
C6A	C5A	B1A	O2A	-166.26(15)	C6B	C5B	B1B	O2B	36.1(2)
C6A	C5A	B1A	O3A	38.1(2)	C6B	C5B	B1B	O3B	-162.52(15)
C6A	C10A	C11A	C12A	172.98(16)	C6B	C10B	C11B	C12B	69.8(2)
C6A	C10A	C11A	C13A	-75.4(6)	C6B	C10B	C11B	C13B	-167.76(15)
C6A	C10A	C11A	C13'	-53.7(6)	C7B	Si04	C5B	N1B	100.43(11)
C7A	Si1A	C5A	N1A	-14.36(14)	C7B	Si04	C5B	C6B	166.73(12)
C7A	Si1A	C5A	C6A	52.18(15)	C7B	Si04	C5B	B1B	-49.09(13)
C7A	Si1A	C5A	B1A	-162.09(12)	C8B	Si04	C5B	N1B	-142.01(11)
C8A	Si1A	C5A	N1A	104.34(11)	C8B	Si04	C5B	C6B	-75.71(14)
C8A	Si1A	C5A	C6A	170.87(12)	C8B	Si04	C5B	B1B	68.47(13)
C8A	Si1A	C5A	B1A	-43.40(14)	C9B	Si04	C5B	N1B	-19.30(13)
C9A	Si1A	C5A	N1A	-136.00(11)	C9B	Si04	C5B	C6B	46.99(15)
C9A	Si1A	C5A	C6A	-69.46(14)	C9B	Si04	C5B	B1B	-168.83(12)
C9A	Si1A	C5A	B1A	76.27(13)	C14B	O2B	B1B	O3B	12.55(18)
C14A	O3A	B1A	O2A	13.19(18)	C14B	O2B	B1B	C5B	175.93(14)
C14A	O3A	B1A	C5A	171.44(13)	C15B	O3B	B1B	O2B	5.62(18)
C15A	O2A	B1A	O3A	4.96(18)	C15B	O3B	B1B	C5B	-156.96(15)
C15A	O2A	B1A	C5A	-152.18(15)	C16B	C14B	C15B	O3B	143.93(15)
C16A	C14A	C15A	O2A	-89.76(15)	C16B	C14B	C15B	C18B	29.5(2)
C16A	C14A	C15A	C18A	28.1(2)	C16B	C14B	C15B	C19B	-98.0(2)
C16A	C14A	C15A	C19A	155.44(15)	C17B	C14B	C15B	O3B	-88.40(16)

Table C.6 Torsion Angles for mo_MP902_0m_a.

A	B	C	D	Angle/˚	A	B	C	D	Angle/˚
C17A	C14A	C15A	O2A	142.38(14)	C17B	C14B	C15B	C18B	157.16(16)
C17A	C14A	C15A	C18A	-99.73(18)	C17B	C14B	C15B	C19B	29.6(2)
C17A	C14A	C15A	C19A	27.6(2)	B1B	O2B	C14B	C15B	-23.71(17)
B1A	O2A	C15A	C14A	-19.32(16)	B1B	O2B	C14B	C16B	-146.06(15)
B1A	O2A	C15A	C18A	-141.91(14)	B1B	O2B	C14B	C17B	95.17(15)
B1A	O2A	C15A	C19A	99.78(15)	B1B	O3B	C15B	C14B	-19.88(16)
B1A	O3A	C14A	C15A	-23.97(16)	B1B	O3B	C15B	C18B	99.11(15)
B1A	O3A	C14A	C16A	95.93(15)	B1B	O3B	C15B	C19B	-142.35(15)
B1A	O3A	C14A	C17A	-145.13(14)	B1B	C5B	C6B	N1B	114.52(14)
B1A	C5A	C6A	N1A	112.64(14)	B1B	C5B	C6B	C10B	-138.03(16)
B1A	C5A	C6A	C10A	-144.32(16)					

Table C.7 Hydrogen Atom Coordinates (Å×10^4) and Isotropic Displacement Parameters (Å^2×10^3) for mo_MP902_0m_a.

Atom	*x*	*y*	*z*	U(eq)
H2AA	2621.29	5632.59	4250.31	40
H2AB	3348.25	5470.5	5778.05	40
H2AC	2589.08	4961.31	4746.17	40
H3AA	6093.58	5642.31	4674.54	50
H3AB	5510.98	5889.01	5736.99	50
H3AC	4794.26	6055.3	4210.25	50
H4AA	3731.27	5200.49	2595.75	33
H4AB	3514.4	4567.3	3172.35	33
H4AC	4964.74	4758.48	3233.69	33
H6A	5913.05	3641.95	7048.69	19
H7AA	1739.97	3712.3	3792.9	45
H7AB	1715.18	3077.83	4463.27	45
H7AC	1154.98	3138.54	2889.69	45
H8AA	2768.9	3030.07	1363.2	32
H8AB	4313.59	3201.88	1934.27	32
H8AC	3214.77	3703.45	1800.16	32
H9AA	3454.71	2060.52	3318.27	40

Table C.7 Hydrogen Atom Coordinates (Å×10^4) and Isotropic Displacement Parameters (Å^2×10^3) for mo_MP902_0m_a.

Atom	*x*	*y*	*z*	U(eq)
H9AB	3727.39	2186.44	4843.15	40
H9AC	4910.09	2249.6	4292.66	40
H10A	4637.78	2866.52	7480.39	23
H10B	3337.39	3069.07	6276.54	23
H11A	3363.06	3992.53	7589.75	33
H11B	3154.24	3917.86	7382.74	33
H12A	1951.48	3113.65	7588.24	43
H12B	2365.86	3505.4	8907.71	43
H12C	3133.66	2895.31	8890.62	43
H16A	10022.79	3893.86	5427.59	34
H16B	9644.72	3802.87	6708.93	34
H16C	8767.72	4235.18	5546.49	34
H17A	8573.23	2420.91	5094.45	36
H17B	9651.18	2728.87	6360.36	36
H17C	9829.53	2756.1	4963.04	36
H18A	7654.79	3905.01	1963.27	35
H18B	9091.71	3688.43	2929.27	35
H18C	8392.67	4249	3324.09	35
H19A	6551.81	2947.05	1920.62	40
H19B	6787.48	2592.11	3264.57	40
H19C	7983.34	2671.18	2737.61	40
H13A	5448.36	3202.78	9520.16	64
H13B	4921.54	3786.68	10027.26	64
H13C	5768.72	3852.04	9093.49	64
H13D	5179.53	4166.33	8672.75	67
H13E	5303.69	3592.47	9588.54	67
H13F	4259.79	4108.86	9537.03	67
H2BA	10020.48	6733.2	11862.99	48
H2BB	11581.71	6729.26	12709.84	48
H2BC	10944.84	6218.78	11634.45	48
H3BA	11163.26	7922.1	10350.16	51

Table C.7 Hydrogen Atom Coordinates (Å×10^4) and Isotropic Displacement Parameters (Å^2×10^3) for mo_MP902_0m_a.

Atom	*x*	*y*	*z*	U(eq)
H3BB	11769.67	7795.72	11904.13	51
H3BC	10194.74	7776.17	11130.2	51
H4BA	12516.95	6452.78	10492.75	68
H4BB	13203.66	6991.97	11469.31	68
H4BC	12542.56	7108.47	9914.18	68
H6B	8218.92	6228	7400.27	16
H7BA	9389.88	5051.11	11836.65	32
H7BB	10909.7	5216.74	12111.15	32
H7BC	10449.16	4532.09	11961.17	32
H8BA	8339.25	4416.55	8060.48	41
H8BB	7709.9	4500.08	9174.86	41
H8BC	8853.71	4014.45	9366.07	41
H9BA	11207.4	4684.96	8676.21	42
H9BB	11916.11	4528.16	10209	42
H9BC	11924.45	5200.5	9709.64	42
H10C	8519.74	5265.02	6416.49	17
H10D	10076.68	5328.51	7246.45	17
H11C	8639.44	6202.6	5334.63	27
H12D	10775.2	6508.42	5425.15	81
H12E	11443.77	6026.61	6560.81	81
H12F	10634.18	6578.06	6833.44	81
H13G	8581.61	5285.96	4234.74	41
H13H	10166.82	5236.2	4884.17	41
H13I	9495.26	5754.41	3840.28	41
H16D	3950.61	5998.31	7828.28	45
H16E	3834.54	5911.35	9243.5	45
H16F	4534.25	5408.52	8672.5	45
H17D	5161.33	6932.51	8301.74	39
H17E	6412.31	7019.72	9643.83	39
H17F	4950.43	6955.33	9683.96	39
H18D	5366.04	5264.02	11078.73	44

Table C.7 Hydrogen Atom Coordinates (Å×10^4) and Isotropic Displacement Parameters (Å^2×10^3) for mo_MP902_0m_a.

Atom	*x*	*y*	*z*	U(eq)
H18E	6911.81	5172.54	11929.86	44
H18F	6355.7	5041.23	10377.38	44
H19D	6723.32	6750	11781.59	42
H19E	6962.09	6187.04	12748.29	42
H19F	5484.75	6363.98	11808.23	42

Table 8 Atomic Occupancy for mo_MP902_0m_a.

Atom	*Occupancy*	Atom	*Occupancy*	Atom	*Occupancy*
H11A	0.38(3)	H11B	0.62(3)	C13A	0.38(3)
H13A	0.38(3)	H13B	0.38(3)	H13C	0.38(3)
C13'	0.62(3)	H13D	0.62(3)	H13E	0.62(3)
H13F	0.62(3)				

Crystal Data for $C_{38}H_{80}B_2N_2O_6S_2Si_2$ (*M* =802.96 g/mol): monoclinic, space group P2_1 (no. 4), *a* = 10.7909(8) Å, *b* = 22.3729(17) Å, *c* = 10.8729(8) Å, *β* = 111.262(2)°, *V* = 2446.3(3) Å^3, *Z* = 2, *T* = 100(2) K, μ(MoKα) = 0.198 mm^{-1}, *Dcalc* = 1.090 g/cm^3, 47255 reflections measured (3.64° ≤ 2Θ ≤ 63.3°), 15526 unique (R_{int} = 0.0277, R_{sigma} = 0.0337) which were used in all calculations. The final R_1 was 0.0309 (I > 2σ(I)) and wR_2 was 0.0764 (all data).

For product (*S*$_S$*,2*R,3*S**,*R*)-40** CCDC-2471994

Code(mo_MP915_0m_a)

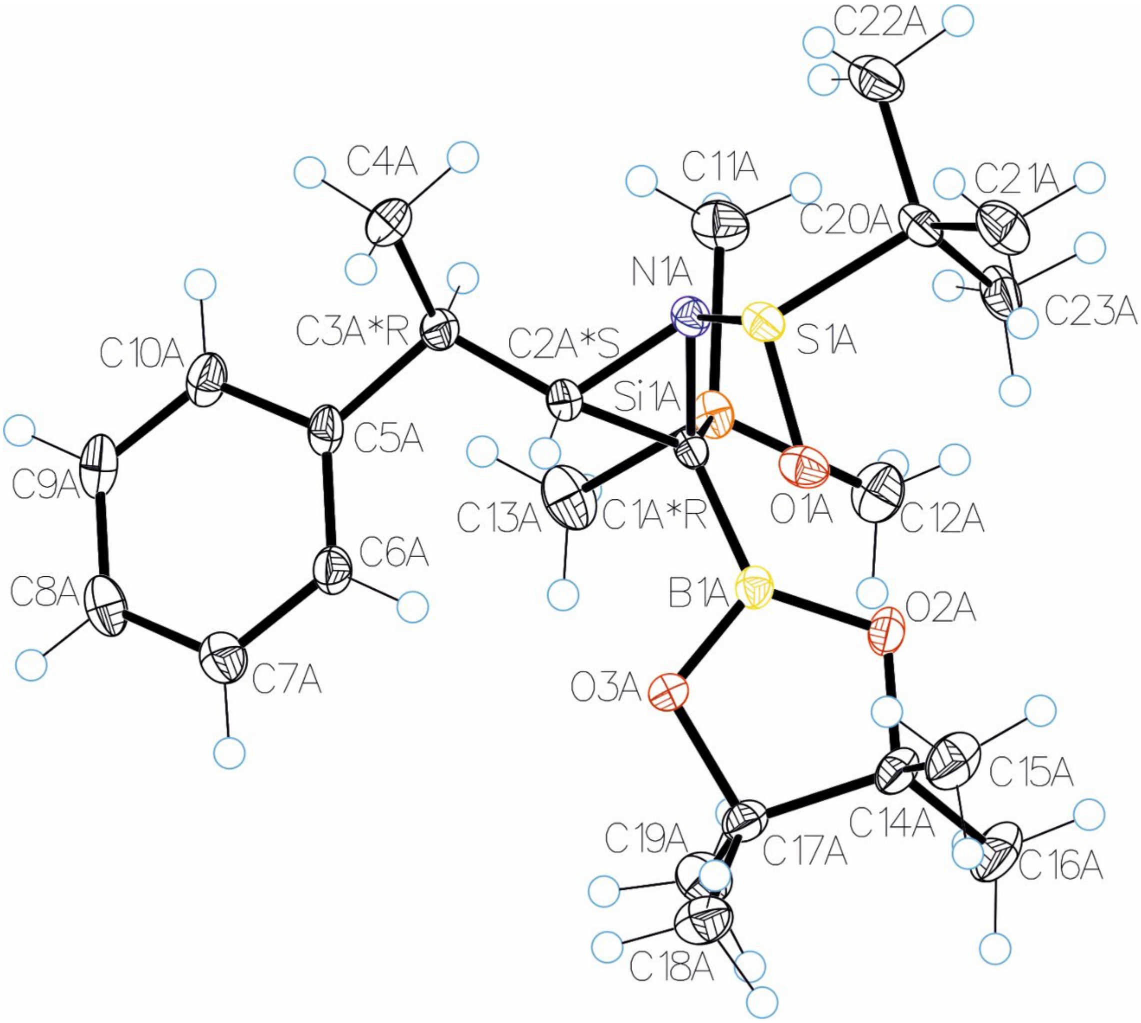

The asymmetric unit contains three independent molecules of the same compound, all exhibiting identical stereochemistry. The absolute structure was reliably determined, with a Flack value of 0.006(55) and a Flack value based on Parsons' quotients of -0.004(35) (References: Flack H.D., *Acta Cryst.* A39 (1983) 876; Parsons S., Flack H., *Acta Cryst.* A39 (2004) S61; Parsons, Flack and Wagner, *Acta Cryst.* B69 (2013) 249–259). The Flack X parameter was determined using 2649 quotients of $[(I^+)-(I^-)]/[(I^+)+(I^-)]$. The expected value for the Flack (Parsons) parameter for the correct absolute structure is 0; the inverted structure would yield 1, always considering the standard deviation. Please note that the absolute configuration was determined based on a single crystal measured; if the enantiomeric excess (ee%) is below 98%, the assignment could be incorrect.

Based on the measured crystal, the absolute configuration was determined as *R*(C1), *S*(C2), and *R*(C3), and was identical for all three independent molecules (A, B, and C). This configuration was assigned manually using the Cahn–Ingold–Prelog

rules; you are advised to double-check the assigned configuration. The structure is of excellent quality (no A- or B-level alerts) and is suitable for publication, with an R1 value of 5.21%.

Table D.1 Crystal data and structure refinement for mo_MP915_0m_a.

Identification code	mo_MP915_0m_a
Empirical formula	$C_{69}H_{120}B_3N_3O_9S_3Si_3$
Formula weight	1348.55
Temperature/K	100(2)
Crystal system	monoclinic
Space group	$P2_1$
a/Å	16.7481(16)
b/Å	10.5412(7)
c/Å	22.3460(15)
α/°	90
β/°	91.513(3)
γ/°	90
Volume/Å^3	3943.7(5)
Z	2
ρ_{calc}g/cm^3	1.136
μ/mm^{-1}	0.191
F(000)	1464.0
Crystal size/mm^3	0.4 × 0.2 × 0.2
Radiation	MoKα (λ = 0.71073)
2Θ range for data collection/°	1.824 to 63.218
Index ranges	-24 ≤ h ≤ 24, -15 ≤ k ≤ 15, -32 ≤ l ≤ 16
Reflections collected	43633
Independent reflections	23214 [R_{int} = 0.0440, R_{sigma} = 0.0730]
Data/restraints/parameters	23214/1/853
Goodness-of-fit on F^2	1.028

Final R indexes [I>=2σ (I)]	R_1 = 0.0521, wR_2 = 0.1163
Final R indexes [all data]	R_1 = 0.0743, wR_2 = 0.1300
Largest diff. peak/hole / e Å^{-3}	0.50/-0.30

Table D.2 Fractional Atomic Coordinates (×10^4) and Equivalent Isotropic Displacement Parameters (Å^2×10^3) for mo_MP915_0m_a. U_{eq} is defined as 1/3 of the trace of the orthogonalised U_{IJ} tensor.

Atom	*x*	*y*	*z*	U(eq)
S1B	4406.4(3)	5299.3(8)	2372.0(3)	15.62(13)
Si1	6468.1(5)	3154.9(9)	1727.5(3)	19.73(18)
O1C	8696.4(11)	1364(2)	4729.4(8)	21.3(4)
O3C	6864.3(11)	1591(2)	5028.8(8)	22.3(5)
O2C	7676.7(11)	686(2)	5749.0(8)	20.4(4)
N1C	8134.0(12)	-776(2)	4320.1(9)	15.2(5)
O1A	1918.3(11)	11260(2)	1330.5(8)	20.9(4)
O2A	941.3(11)	10493(2)	2353.9(8)	19.5(4)
O3A	143.0(11)	11438(2)	1632.7(8)	18.5(4)
O1B	4607.2(11)	6382(2)	1974.3(9)	22.5(4)
O8	5741.1(11)	5712(2)	1007.1(8)	20.7(4)
O9	6335.4(11)	6863(2)	1766.4(8)	17.0(4)
C1C	7451.6(14)	-548(3)	4739.3(11)	14.8(5)
C6A	-971.7(15)	10251(3)	362.0(11)	19.8(6)
C5C	6103.1(15)	-584(3)	3466.1(11)	15.9(5)
C1A	725.1(14)	9292(3)	1338.2(11)	14.4(5)
C2C	7377.8(14)	-253(3)	4080.7(10)	15.1(5)
C4C	7463.7(17)	-987(3)	3034.9(11)	23.5(6)
C5B	7183.6(15)	4447(3)	3297.1(11)	16.5(5)
C2B	5910.8(14)	4890(3)	2695.4(10)	15.3(5)
C17A	-51.1(16)	12042(3)	2202.0(12)	19.8(6)
C12A	490.3(19)	7659(4)	2478.6(12)	28.7(7)

Table D.2 Fractional Atomic Coordinates (×10^4) and Equivalent Isotropic Displacement Parameters (Å^2×10^3) for mo_MP915_0m_a. U_{eq} is defined as 1/3 of the trace of the orthogonalised U_{IJ} tensor.

Atom	*x*	*y*	*z*	U(eq)
C5A	-623.9(16)	9225(3)	79.4(11)	19.0(6)
C2A	649.4(15)	9595(3)	679.7(11)	16.4(5)
C9A	-1880.2(17)	8865(3)	-462.7(12)	24.4(7)
C7B	8450.3(17)	3898(3)	3786.6(13)	25.7(7)
C9B	8376.1(16)	5674(3)	3119.8(12)	23.6(6)
C1B	5922.2(14)	4584(3)	2038.2(11)	15.0(5)
N1B	5202.3(12)	4286(2)	2415.6(9)	15.6(5)
C22C	10379.6(15)	103(3)	4812.8(12)	21.7(6)
C11B	6021(2)	2631(4)	995.8(13)	38.5(9)
C13C	7213.1(18)	-2176(4)	5884.3(12)	29.9(8)
C9C	4853.0(17)	-897(3)	2909.2(12)	25.6(7)
C20C	9637.1(15)	-721(3)	4725.1(11)	17.2(5)
C21C	9802.7(16)	-1821(3)	4302.6(13)	23.5(6)
C7A	-1762.5(17)	10597(3)	229.8(13)	24.5(7)
C8A	-2216.3(17)	9909(3)	-184.9(13)	24.8(7)
C10B	7560.7(15)	5447(3)	3016.0(11)	19.6(6)
C6B	7638.7(17)	3669(3)	3684.0(12)	21.3(6)
C13A	-764.9(16)	7620(4)	1483.9(14)	31.0(7)
C21B	2955.7(16)	5062(3)	1861.9(13)	25.2(7)
C8B	8820.0(17)	4906(3)	3505.9(13)	24.8(7)
C6C	5768.8(16)	469(3)	3739.9(12)	21.2(6)
C10C	5633.7(17)	-1263(3)	3051.6(12)	21.7(6)
C22A	3137.0(18)	8176(3)	904.0(13)	28.1(7)
C7C	4985.0(16)	842(3)	3591.4(13)	24.1(6)
C14A	690.1(16)	11705(3)	2604.9(11)	20.6(6)
C15A	1373.6(17)	12635(4)	2544.4(13)	30.6(8)
C23A	2645.8(16)	8823(3)	1922.0(12)	25.3(7)

Table D.2 Fractional Atomic Coordinates (×10^4) and Equivalent Isotropic Displacement Parameters (Å^2×10^3) for mo_MP915_0m_a. U_{eq} is defined as 1/3 of the trace of the orthogonalised U_{IJ} tensor.

Atom	*x*	*y*	*z*	U(eq)
C16C	5919.2(17)	1495(4)	5824.5(13)	30.8(8)
C23C	9326.7(16)	-1162(3)	5323.4(12)	23.4(6)
C17B	6082.2(18)	6820(3)	721.5(12)	23.9(6)
C10A	-1095.5(17)	8529(3)	-334.1(12)	22.5(6)
C21A	3633.1(16)	10185(4)	1390.7(13)	28.3(7)
C16A	515.9(19)	11504(4)	3262.0(12)	32.4(8)
C23B	3543.0(17)	3146(3)	2366.0(13)	27.8(7)
C14B	6288.0(16)	7727(3)	1255.1(11)	20.9(6)
C4B	5839.1(17)	4459(4)	3778.0(12)	27.6(7)
C18A	-183(2)	13450(3)	2095.6(14)	29.0(7)
C8C	4530.4(16)	166(3)	3175.6(12)	25.1(7)
C4A	726.8(18)	8956(4)	-381.7(11)	29.0(7)
C22B	4046.0(17)	3870(3)	1361.6(12)	26.0(7)
C19A	-819.2(16)	11418(4)	2407.7(13)	28.9(7)
C12C	7525.3(18)	-3484(3)	4709.4(13)	26.5(7)
C11C	5928.7(16)	-2093(4)	4917.4(13)	30.7(8)
C18C	8076(2)	2864(4)	5910.5(15)	42.6(10)
S1A	2151.7(4)	10207.4(7)	925.2(3)	16.14(14)
C11A	880.1(18)	6371(3)	1311.6(13)	26.5(7)
C15B	5604.4(18)	8655(3)	1375.0(14)	31.3(7)
C18B	5496(2)	7312(4)	257.1(15)	48.1(11)
S1C	8898.8(3)	287.1(8)	4327.7(3)	14.88(13)
Si1A	332.8(4)	7727.7(9)	1645.4(3)	17.85(17)
C16B	7064.7(18)	8450(4)	1223.6(14)	32.8(8)
B1A	675.3(17)	10477(3)	1766.9(12)	15.8(6)
C3A	241.4(17)	8828(3)	192.2(11)	19.3(6)
N1A	1413.5(13)	9092(2)	917.3(9)	16.0(5)

Table D.2 Fractional Atomic Coordinates (×10^4) and Equivalent Isotropic Displacement Parameters (Å^2×10^3) for mo_MP915_0m_a. U_{eq} is defined as 1/3 of the trace of the orthogonalised U_{IJ} tensor.

Atom	*x*	*y*	*z*	U(eq)
C20A	2929.3(15)	9272(3)	1317.1(12)	19.2(6)
C19C	7261.6(19)	1725(4)	6652.7(12)	38.1(9)
C19B	6841(2)	6346(4)	416.8(16)	45.3(10)
C13B	7520.6(18)	3693(4)	1604.1(17)	43.4(10)
B1B	5928.8(17)	5781(3)	1610.6(13)	16.5(6)
C15C	6466(2)	3563(4)	5484.8(15)	39.7(9)
B1C	7403.8(17)	650(3)	5160.0(13)	17.8(6)
Si3	7033.5(4)	-2083.4(8)	5053.4(3)	18.80(18)
C17C	7410.0(17)	1894(3)	5993.3(12)	25.1(7)
C14C	6663.8(18)	2179(3)	5598.4(12)	24.7(7)
C3C	6956.3(16)	-1025(3)	3598.9(11)	17.4(5)
C12B	6499(3)	1776(4)	2236.3(15)	47.3(10)
C3B	6299.3(16)	4164(3)	3208.1(11)	17.4(6)
C20B	3707.8(15)	4264(3)	1958.0(11)	16.6(5)

Table D.3 Anisotropic Displacement Parameters (Å^2×10^3) for mo_MP915_0m_a. The Anisotropic displacement factor exponent takes the form: -2π^2[h^{2}a*^{2}U$_{11}$+2hka*b*U$_{12}$+…].

Atom	U_{11}	U_{22}	U_{33}	U_{23}	U_{13}	U_{12}
S1B	13.3(3)	16.8(4)	16.7(3)	-2.7(3)	0.1(2)	0.6(3)
Si1	21.1(4)	18.9(5)	19.1(3)	-2.1(3)	-0.6(3)	7.5(3)
O1C	19.9(9)	18.5(12)	25.4(10)	-2.4(8)	1.1(7)	0.1(8)
O3C	27.5(10)	24.1(13)	15.2(9)	-4.8(9)	-0.3(7)	5.6(9)
O2C	18.8(9)	28.1(13)	14.2(8)	-2.1(8)	-1.3(7)	-2.8(8)
N1C	12.3(9)	18.3(13)	14.9(10)	2.6(9)	-0.5(7)	-0.6(9)
O1A	21.7(10)	17.3(12)	23.9(10)	1.6(8)	2.8(7)	3.8(8)
O2A	18.0(8)	26.4(13)	13.8(8)	0.0(8)	-2.3(6)	-1.6(8)

Table D.3 Anisotropic Displacement Parameters (Å^2×10^3) for mo_MP915_0m_a. The Anisotropic displacement factor exponent takes the form: -2π^2[h^{2}a*^{2}U$_{11}$+2hka*b*U$_{12}$+…].

Atom	U_{11}	U_{22}	U_{33}	U_{23}	U_{13}	U_{12}
O3A	19.5(9)	20.1(12)	16.0(9)	-4.5(8)	-1.5(7)	4.5(8)
O1B	19.0(9)	17.2(12)	31.2(11)	4.2(9)	-1.8(8)	-0.3(8)
O8	24.2(10)	24.2(13)	13.6(8)	0.1(8)	-2.5(7)	-0.5(9)
O9	17.3(9)	18.6(12)	15.0(8)	2.2(8)	-1.2(7)	0.2(8)
C1C	11.2(11)	19.3(15)	14.0(11)	1.8(10)	-0.9(8)	-1.6(10)
C6A	18.5(12)	21.4(16)	19.1(12)	-0.9(12)	-4.3(9)	-1.1(12)
C5C	17.3(12)	17.6(15)	12.7(11)	2.9(10)	-2.8(9)	-1.1(11)
C1A	11.3(10)	17.8(15)	14.1(11)	3.1(10)	-0.5(8)	1.7(10)
C2C	14.5(11)	16.4(15)	14.3(11)	0.1(10)	-2.2(9)	-0.3(10)
C4C	25.0(14)	28.9(19)	16.5(12)	-2.1(12)	0.0(10)	6.7(13)
C5B	18.0(12)	15.9(15)	15.3(11)	-3.1(10)	-5.1(9)	2.6(11)
C2B	13.9(11)	15.1(15)	16.6(11)	0.2(10)	-3.4(9)	0.9(10)
C17A	18.4(12)	23.3(17)	17.8(12)	-4.7(11)	2.8(10)	-1.9(11)
C12A	35.8(16)	29(2)	21.7(14)	7.6(14)	2.2(12)	-2.8(15)
C5A	24.0(13)	17.5(16)	15.2(11)	3.0(11)	-6.2(10)	-1.0(11)
C2A	16.5(12)	17.8(15)	14.6(11)	1.3(10)	-1.9(9)	2.3(11)
C9A	29.2(15)	22.9(17)	20.3(13)	5.8(12)	-9.8(11)	-8.3(13)
C7B	25.4(14)	26.4(18)	24.7(14)	-4.8(13)	-10.6(11)	7.6(13)
C9B	20.9(13)	26.6(18)	23.3(13)	0.4(12)	-0.8(10)	0.8(12)
C1B	11.9(11)	17.7(15)	15.4(11)	-1.1(10)	-1.3(9)	1.7(10)
N1B	12.5(9)	17.4(13)	16.8(10)	-1.8(9)	-0.7(8)	2.4(9)
C22C	16.0(12)	22.8(17)	26.0(13)	2.6(12)	-3.7(10)	-1.1(12)
C11B	36.7(18)	45(2)	33.5(17)	-21.6(17)	-10.8(14)	19.3(17)
C13C	33.6(16)	36(2)	19.6(13)	9.7(14)	-0.9(11)	-10.5(15)
C9C	24.2(14)	30(2)	22.7(13)	5.8(13)	-8.5(11)	-7.5(13)
C20C	14.0(11)	19.4(16)	18.2(12)	4.5(11)	-0.7(9)	2.2(11)
C21C	18.8(12)	24.1(18)	27.6(14)	-2.8(13)	-1.4(10)	5.8(12)

Table D.3 Anisotropic Displacement Parameters (Å^2×10^3) for mo_MP915_0m_a. The Anisotropic displacement factor exponent takes the form: -2π^2[h^{2}a*^{2}U$_{11}$+2hka*b*U$_{12}$+…].

Atom	U_{11}	U_{22}	U_{33}	U_{23}	U_{13}	U_{12}
C7A	21.1(13)	26.7(19)	25.6(14)	1.0(12)	-0.7(11)	0.1(12)
C8A	17.7(13)	27.7(19)	28.6(14)	7.4(13)	-5.4(11)	-4.1(12)
C10B	18.1(12)	22.5(18)	18.1(12)	0.2(11)	-2.6(9)	2.3(11)
C6B	24.5(13)	18.6(16)	20.4(13)	-0.7(11)	-7.3(10)	3.3(12)
C13A	18.4(13)	35(2)	39.6(17)	7.8(16)	-0.4(12)	-6.2(14)
C21B	18.0(12)	25.5(19)	31.6(14)	-6.4(13)	-7.1(11)	2.8(12)
C8B	18.0(13)	29(2)	27.0(14)	-8.1(13)	-4.0(10)	3.0(12)
C6C	19.7(12)	25.5(19)	18.2(12)	-2.8(12)	-1.4(9)	1.9(12)
C10C	28.0(14)	17.8(16)	18.9(12)	2.0(11)	-7.2(10)	-1.1(12)
C22A	25.9(14)	26.9(19)	31.5(15)	2.5(14)	2.9(12)	8.9(14)
C7C	19.2(13)	27.2(19)	26.1(14)	1.0(13)	2.2(11)	5.1(12)
C14A	19.6(12)	27.7(18)	14.5(12)	-4.3(11)	2.3(9)	-5.2(12)
C15A	23.7(14)	39(2)	29.7(15)	-10.2(15)	3.0(11)	-12.5(15)
C23A	18.7(13)	36(2)	20.6(13)	10.2(13)	-1.0(10)	5.0(13)
C16C	19.7(14)	42(2)	30.9(15)	-1.6(15)	2.3(11)	1.8(14)
C23C	17.7(12)	31.9(19)	20.6(13)	8.7(12)	0.0(10)	1.6(12)
C17B	34.5(16)	21.3(18)	16.2(12)	3.0(11)	2.9(11)	2.6(13)
C10A	30.7(15)	16.7(16)	19.8(13)	0.5(11)	-7.9(11)	-2.6(12)
C21A	16.2(12)	29.8(19)	38.7(16)	1.4(16)	-0.7(11)	0.5(14)
C16A	34.5(17)	47(2)	16.2(13)	-3.7(14)	3.4(12)	-7.1(16)
C23B	21.2(13)	28.2(19)	33.8(15)	7.4(14)	-3.2(11)	-9.7(13)
C14B	21.7(12)	24.4(17)	16.6(12)	6.2(12)	-0.3(10)	-2.3(12)
C4B	24.0(14)	41(2)	17.9(13)	5.8(13)	-1.5(11)	0.1(14)
C18A	35.2(17)	24.0(19)	28.0(15)	-4.2(13)	3.6(12)	1.8(14)
C8C	17.9(12)	33(2)	23.8(13)	6.3(14)	-4.3(10)	-3.4(14)
C4A	31.6(15)	40(2)	15.1(12)	-4.0(13)	0.0(11)	8.0(15)
C22B	20.6(13)	36(2)	21.5(13)	-9.4(13)	1.4(10)	-2.9(13)

Table D.3 Anisotropic Displacement Parameters ($Å^2 \times 10^3$) for mo_MP915_0m_a. The Anisotropic displacement factor exponent takes the form: -$2\pi^2[h^2a^{*2}U_{11}+2hka^*b^*U_{12}+\ldots]$.

Atom	U_{11}	U_{22}	U_{33}	U_{23}	U_{13}	U_{12}
C19A	18.2(13)	36(2)	32.7(16)	-1.6(14)	5.4(11)	-2.7(13)
C12C	31.9(16)	18.4(17)	28.9(15)	2.8(13)	-1.9(12)	-3.8(13)
C11C	18.6(13)	42(2)	31.3(15)	7.9(15)	-0.5(11)	-13.7(14)
C18C	37.0(18)	50(3)	41.2(19)	-21.1(18)	15.1(15)	-24.4(18)
S1A	15.1(3)	17.6(4)	16.0(3)	4.3(3)	3.6(2)	2.2(3)
C11A	31.7(16)	17.5(17)	30.4(15)	3.5(13)	2.9(12)	1.8(13)
C15B	32.3(16)	30(2)	32.2(16)	6.1(14)	5.5(13)	6.5(14)
C18B	64(3)	46(3)	32.5(18)	7.1(18)	-19.8(17)	1(2)
S1C	12.8(2)	16.4(4)	15.5(3)	3.0(3)	1.4(2)	0.4(3)
Si1A	16.6(3)	18.9(5)	18.0(3)	4.0(3)	-0.1(3)	-1.6(3)
C16B	29.1(16)	39(2)	30.2(16)	7.4(15)	4.1(12)	-6.7(15)
B1A	14.4(12)	19.6(18)	13.5(12)	1.4(11)	0.4(9)	-1.2(11)
C3A	24.3(13)	18.2(16)	15.2(12)	-0.1(11)	-3.1(10)	5.7(12)
N1A	15.3(10)	18.5(14)	14.3(10)	2.8(9)	1.1(8)	2.5(9)
C20A	14.2(12)	21.4(17)	22.1(13)	4.5(11)	1.3(9)	4.7(11)
C19C	31.6(17)	65(3)	17.4(14)	-8.4(16)	2.2(12)	-7.5(18)
C19B	54(2)	37(2)	46(2)	-2.5(18)	28.5(18)	3.5(19)
C13B	23.9(16)	50(3)	57(2)	-12(2)	6.3(15)	14.2(17)
B1B	12.5(12)	21.0(18)	16.0(13)	0.6(12)	0.1(10)	3.9(12)
C15C	66(3)	25(2)	28.9(17)	-4.9(15)	15.9(16)	6.1(18)
B1C	15.6(13)	23.5(19)	14.2(13)	1.8(12)	0.1(10)	-3.6(12)
Si3	17.7(3)	21.7(5)	17.0(3)	3.1(3)	-1.0(3)	-5.7(3)
C17C	24.6(14)	33(2)	17.9(13)	-8.3(13)	4.9(10)	-8.7(13)
C14C	28.8(15)	26.0(19)	19.4(13)	-6.1(12)	3.2(11)	1.0(13)
C3C	20.6(12)	16.0(15)	15.4(12)	1.5(11)	-4.0(9)	1.6(11)
C12B	83(3)	24(2)	35.0(18)	-1.7(16)	3.8(18)	18(2)
C3B	19.7(12)	17.0(16)	15.3(11)	2.0(11)	-3.2(9)	1.9(11)

Table D.3 Anisotropic Displacement Parameters (Å^2×10^3) for mo_MP915_0m_a. The Anisotropic displacement factor exponent takes the form: -2π^2[h^{2}a*^{2}U$_{11}$+2hka*b*U$_{12}$+…].

Atom	U_{11}	U_{22}	U_{33}	U_{23}	U_{13}	U_{12}
C20B	12.8(11)	18.8(16)	18.1(12)	-3.2(11)	-1.3(9)	-0.9(10)

Table D.4 Bond Lengths for mo_MP915_0m_a.

Atom	Atom	Length/Å	Atom	Atom	Length/Å
S1B	O1B	1.491(2)	C5A	C10A	1.406(4)
S1B	N1B	1.709(2)	C5A	C3A	1.523(4)
S1B	C20B	1.832(3)	C2A	C3A	1.506(4)
Si1	C1B	1.903(3)	C2A	N1A	1.472(3)
Si1	C11B	1.864(3)	C9A	C8A	1.390(5)
Si1	C13B	1.879(3)	C9A	C10A	1.384(4)
Si1	C12B	1.845(4)	C7B	C6B	1.394(4)
O1C	S1C	1.492(2)	C7B	C8B	1.388(5)
O3C	B1C	1.369(4)	C9B	C10B	1.400(4)
O3C	C14C	1.463(3)	C9B	C8B	1.385(4)
O2C	B1C	1.382(3)	C1B	N1B	1.522(3)
O2C	C17C	1.460(4)	C1B	B1B	1.583(4)
N1C	C1C	1.516(3)	C22C	C20C	1.525(4)
N1C	C2C	1.469(3)	C13C	Si3	1.876(3)
N1C	S1C	1.702(2)	C9C	C10C	1.392(4)
O1A	S1A	1.491(2)	C9C	C8C	1.385(5)
O2A	C14A	1.461(4)	C20C	C21C	1.525(4)
O2A	B1A	1.374(3)	C20C	C23C	1.520(4)
O3A	C17A	1.467(3)	C20C	S1C	1.841(3)
O3A	B1A	1.377(4)	C7A	C8A	1.388(4)
O8	C17B	1.455(4)	C13A	Si1A	1.868(3)
O8	B1B	1.378(3)	C21B	C20B	1.525(4)
O9	C14B	1.462(3)	C6C	C7C	1.402(4)

Table D.4 Bond Lengths for mo_MP915_0m_a.

Atom	Atom	Length/Å	Atom	Atom	Length/Å
O9	B1B	1.368(4)	C22A	C20A	1.525(4)
C1C	C2C	1.506(3)	C7C	C8C	1.383(4)
C1C	B1C	1.577(4)	C14A	C15A	1.516(4)
C1C	Si3	1.905(3)	C14A	C16A	1.519(4)
C6A	C5A	1.388(4)	C23A	C20A	1.520(4)
C6A	C7A	1.398(4)	C16C	C14C	1.537(4)
C5C	C6C	1.392(4)	C17B	C14B	1.560(4)
C5C	C10C	1.397(4)	C17B	C18B	1.502(4)
C5C	C3C	1.524(4)	C17B	C19B	1.540(4)
C1A	C2A	1.508(3)	C21A	C20A	1.527(4)
C1A	Si1A	1.910(3)	C23B	C20B	1.520(4)
C1A	B1A	1.577(4)	C14B	C15B	1.534(4)
C1A	N1A	1.522(3)	C14B	C16B	1.511(4)
C2C	C3C	1.510(4)	C4B	C3B	1.538(4)
C4C	C3C	1.539(4)	C4A	C3A	1.542(4)
C5B	C10B	1.387(4)	C22B	C20B	1.520(4)
C5B	C6B	1.403(4)	C12C	Si3	1.866(3)
C5B	C3B	1.519(4)	C11C	Si3	1.867(3)
C2B	C1B	1.504(3)	C18C	C17C	1.529(4)
C2B	N1B	1.472(3)	S1A	N1A	1.706(2)
C2B	C3B	1.511(3)	S1A	C20A	1.837(3)
C17A	C14A	1.555(4)	C11A	Si1A	1.865(3)
C17A	C18A	1.519(5)	C19C	C17C	1.511(4)
C17A	C19A	1.526(4)	C15C	C14C	1.516(5)
C12A	Si1A	1.875(3)	C17C	C14C	1.540(4)

Table D.5 Bond Angles for mo_MP915_0m_a.

Atom	Atom	Atom	Angle/˚	Atom	Atom	Atom	Angle/˚
O1B	S1B	N1B	108.96(11)	O2A	C14A	C15A	107.8(2)
O1B	S1B	C20B	107.83(12)	O2A	C14A	C16A	108.3(3)
N1B	S1B	C20B	98.23(12)	C15A	C14A	C17A	113.2(3)
C11B	Si1	C1B	111.64(13)	C15A	C14A	C16A	109.9(2)
C11B	Si1	C13B	108.48(16)	C16A	C14A	C17A	114.9(2)
C13B	Si1	C1B	106.03(15)	O8	C17B	C14B	103.8(2)
C12B	Si1	C1B	113.87(14)	O8	C17B	C18B	108.9(3)
C12B	Si1	C11B	108.13(18)	O8	C17B	C19B	105.8(3)
C12B	Si1	C13B	108.52(19)	C18B	C17B	C14B	116.3(3)
B1C	O3C	C14C	106.6(2)	C18B	C17B	C19B	109.8(3)
B1C	O2C	C17C	106.5(2)	C19B	C17B	C14B	111.6(3)
C1C	N1C	S1C	117.96(18)	C9A	C10A	C5A	121.1(3)
C2C	N1C	C1C	60.57(15)	O9	C14B	C17B	102.8(2)
C2C	N1C	S1C	113.43(19)	O9	C14B	C15B	106.6(2)
B1A	O2A	C14A	106.7(2)	O9	C14B	C16B	108.8(2)
B1A	O3A	C17A	106.7(2)	C15B	C14B	C17B	111.9(2)
B1B	O8	C17B	107.7(2)	C16B	C14B	C17B	116.5(2)
B1B	O9	C14B	107.7(2)	C16B	C14B	C15B	109.5(3)
N1C	C1C	B1C	123.1(2)	C7C	C8C	C9C	119.4(3)
N1C	C1C	Si3	112.5(2)	O1A	S1A	N1A	108.53(11)
C2C	C1C	N1C	58.18(15)	O1A	S1A	C20A	107.67(12)
C2C	C1C	B1C	114.4(2)	N1A	S1A	C20A	98.02(13)
C2C	C1C	Si3	120.95(19)	O1C	S1C	N1C	108.92(11)
B1C	C1C	Si3	115.81(18)	O1C	S1C	C20C	108.03(12)
C5A	C6A	C7A	120.9(3)	N1C	S1C	C20C	96.92(12)
C6C	C5C	C10C	118.3(3)	C12A	Si1A	C1A	110.46(14)
C6C	C5C	C3C	122.9(2)	C13A	Si1A	C1A	109.23(14)
C10C	C5C	C3C	118.7(3)	C13A	Si1A	C12A	107.54(14)

Table D.5 Bond Angles for mo_MP915_0m_a.

Atom	Atom	Atom	Angle/˚	Atom	Atom	Atom	Angle/˚
C2A	C1A	Si1A	120.8(2)	C11A	Si1A	C1A	109.94(13)
C2A	C1A	B1A	114.9(2)	C11A	Si1A	C12A	108.05(15)
C2A	C1A	N1A	58.12(15)	C11A	Si1A	C13A	111.59(16)
B1A	C1A	Si1A	116.19(18)	O2A	B1A	O3A	113.0(2)
N1A	C1A	Si1A	112.05(19)	O2A	B1A	C1A	124.6(3)
N1A	C1A	B1A	122.6(2)	O3A	B1A	C1A	119.6(2)
N1C	C2C	C1C	61.24(15)	C5A	C3A	C4A	111.0(2)
N1C	C2C	C3C	116.1(2)	C2A	C3A	C5A	112.6(2)
C1C	C2C	C3C	127.6(2)	C2A	C3A	C4A	108.4(2)
C10B	C5B	C6B	118.5(2)	C1A	N1A	S1A	117.27(18)
C10B	C5B	C3B	123.0(2)	C2A	N1A	C1A	60.46(15)
C6B	C5B	C3B	118.5(3)	C2A	N1A	S1A	112.22(19)
C1B	C2B	C3B	128.0(2)	C22A	C20A	C21A	110.8(2)
N1B	C2B	C1B	61.52(16)	C22A	C20A	S1A	106.74(18)
N1B	C2B	C3B	115.4(2)	C23A	C20A	C22A	112.6(3)
O3A	C17A	C14A	102.2(2)	C23A	C20A	C21A	111.0(2)
O3A	C17A	C18A	108.8(2)	C23A	C20A	S1A	110.91(18)
O3A	C17A	C19A	106.4(2)	C21A	C20A	S1A	104.4(2)
C18A	C17A	C14A	115.1(3)	O8	B1B	C1B	122.9(3)
C18A	C17A	C19A	110.3(3)	O9	B1B	O8	113.1(3)
C19A	C17A	C14A	113.2(2)	O9	B1B	C1B	121.5(2)
C6A	C5A	C10A	118.0(3)	O3C	B1C	O2C	112.5(3)
C6A	C5A	C3A	123.2(2)	O3C	B1C	C1C	119.8(2)
C10A	C5A	C3A	118.7(3)	O2C	B1C	C1C	124.7(3)
C3A	C2A	C1A	128.1(3)	C13C	Si3	C1C	110.99(14)
N1A	C2A	C1A	61.42(15)	C12C	Si3	C1C	110.47(13)
N1A	C2A	C3A	116.2(2)	C12C	Si3	C13C	107.80(15)
C10A	C9A	C8A	120.2(3)	C12C	Si3	C11C	112.03(16)

Table D.5 Bond Angles for mo_MP915_0m_a.

Atom	Atom	Atom	Angle/˚	Atom	Atom	Atom	Angle/˚
C8B	C7B	C6B	120.2(3)	C11C	Si3	C1C	108.48(14)
C8B	C9B	C10B	120.5(3)	C11C	Si3	C13C	107.04(13)
C2B	C1B	Si1	123.00(19)	O2C	C17C	C18C	107.9(2)
C2B	C1B	N1B	58.18(15)	O2C	C17C	C19C	108.8(3)
C2B	C1B	B1B	114.7(2)	O2C	C17C	C14C	102.0(2)
N1B	C1B	Si1	115.7(2)	C18C	C17C	C14C	112.6(3)
N1B	C1B	B1B	121.1(2)	C19C	C17C	C18C	109.6(3)
B1B	C1B	Si1	113.57(18)	C19C	C17C	C14C	115.4(2)
C2B	N1B	S1B	111.88(19)	O3C	C14C	C16C	107.1(2)
C2B	N1B	C1B	60.30(16)	O3C	C14C	C15C	108.4(2)
C1B	N1B	S1B	117.87(18)	O3C	C14C	C17C	102.4(2)
C8C	C9C	C10C	120.0(3)	C16C	C14C	C17C	111.9(3)
C22C	C20C	C21C	110.6(2)	C15C	C14C	C16C	109.3(3)
C22C	C20C	S1C	105.6(2)	C15C	C14C	C17C	117.0(3)
C21C	C20C	S1C	105.72(17)	C5C	C3C	C4C	111.6(2)
C23C	C20C	C22C	111.0(2)	C2C	C3C	C5C	113.1(2)
C23C	C20C	C21C	112.6(3)	C2C	C3C	C4C	108.2(2)
C23C	C20C	S1C	111.09(18)	C5B	C3B	C4B	111.1(2)
C8A	C7A	C6A	120.3(3)	C2B	C3B	C5B	113.5(2)
C7A	C8A	C9A	119.4(3)	C2B	C3B	C4B	108.1(2)
C5B	C10B	C9B	120.6(3)	C21B	C20B	S1B	104.9(2)
C7B	C6B	C5B	120.8(3)	C23B	C20B	S1B	106.42(18)
C9B	C8B	C7B	119.4(3)	C23B	C20B	C21B	110.5(2)
C5C	C6C	C7C	120.3(3)	C23B	C20B	C22B	113.0(3)
C9C	C10C	C5C	121.2(3)	C22B	C20B	S1B	110.97(18)
C8C	C7C	C6C	120.6(3)	C22B	C20B	C21B	110.7(2)
O2A	C14A	C17A	102.2(2)				

Table D.6 Torsion Angles for mo_MP915_0m_a.

A	B	C	D	Angle/˚	A	B	C	D	Angle/˚
Si1	C1B	N1B	S1B	-145.05(14)	C23C	C20C	S1C	N1C	-56.5(2)
Si1	C1B	N1B	C2B	114.4(2)	C17B	O8	B1B	O9	2.7(3)
Si1	C1B	B1B	O8	47.8(3)	C17B	O8	B1B	C1B	-159.3(2)
Si1	C1B	B1B	O9	-112.8(3)	C10A	C5A	C3A	C2A	-175.5(3)
O2C	C17C	C14C	O3C	-31.0(3)	C10A	C5A	C3A	C4A	62.8(4)
O2C	C17C	C14C	C16C	83.4(3)	C10A	C9A	C8A	C7A	-1.1(5)
O2C	C17C	C14C	C15C	-149.4(3)	C14B	O9	B1B	O8	12.1(3)
N1C	C1C	C2C	C3C	102.1(3)	C14B	O9	B1B	C1B	174.4(2)
N1C	C1C	B1C	O3C	107.2(3)	C18A	C17A	C14A	O2A	-147.6(2)
N1C	C1C	B1C	O2C	-93.9(3)	C18A	C17A	C14A	C15A	-32.0(3)
N1C	C2C	C3C	C5C	167.8(2)	C18A	C17A	C14A	C16A	95.5(3)
N1C	C2C	C3C	C4C	-68.1(3)	C8C	C9C	C10C	C5C	0.6(4)
O1A	S1A	N1A	C1A	-4.9(2)	C19A	C17A	C14A	O2A	84.2(3)
O1A	S1A	N1A	C2A	62.24(18)	C19A	C17A	C14A	C15A	-160.2(3)
O1A	S1A	C20A	C22A	178.02(19)	C19A	C17A	C14A	C16A	-32.8(4)
O1A	S1A	C20A	C23A	55.1(3)	C18C	C17C	C14C	O3C	84.3(3)
O1A	S1A	C20A	C21A	-64.6(2)	C18C	C17C	C14C	C16C	-161.3(3)
O3A	C17A	C14A	O2A	-29.8(3)	C18C	C17C	C14C	C15C	-34.0(4)
O3A	C17A	C14A	C15A	85.8(3)	C18B	C17B	C14B	O9	140.9(3)
O3A	C17A	C14A	C16A	-146.8(3)	C18B	C17B	C14B	C15B	26.8(4)
O1B	S1B	N1B	C2B	63.52(19)	C18B	C17B	C14B	C16B	-100.3(4)
O1B	S1B	N1B	C1B	-3.4(2)	S1C	N1C	C1C	C2C	102.6(2)
O1B	S1B	C20B	C21B	-65.2(2)	S1C	N1C	C1C	B1C	2.5(3)
O1B	S1B	C20B	C23B	177.74(19)	S1C	N1C	C1C	Si3	-144.01(14)
O1B	S1B	C20B	C22B	54.4(2)	S1C	N1C	C2C	C1C	-110.0(2)
O8	C17B	C14B	O9	21.3(3)	S1C	N1C	C2C	C3C	129.6(2)
O8	C17B	C14B	C15B	-92.7(3)	Si1A	C1A	C2A	C3A	3.9(4)
O8	C17B	C14B	C16B	140.2(3)	Si1A	C1A	C2A	N1A	-98.3(2)

Table D.6 Torsion Angles for mo_MP915_0m_a.

A	B	C	D	Angle/˚	A	B	C	D	Angle/˚
C1C	N1C	C2C	C3C	-120.4(3)	Si1A	C1A	B1A	O2A	49.7(3)
C1C	N1C	S1C	O1C	-5.6(2)	Si1A	C1A	B1A	O3A	-110.1(2)
C1C	N1C	S1C	C20C	106.22(19)	Si1A	C1A	N1A	C2A	113.5(2)
C1C	C2C	C3C	C5C	95.2(3)	Si1A	C1A	N1A	S1A	-145.11(13)
C1C	C2C	C3C	C4C	-140.7(3)	B1A	O2A	C14A	C17A	25.6(3)
C6A	C5A	C10A	C9A	0.9(4)	B1A	O2A	C14A	C15A	-93.9(3)
C6A	C5A	C3A	C2A	5.1(4)	B1A	O2A	C14A	C16A	147.3(2)
C6A	C5A	C3A	C4A	-116.7(3)	B1A	O3A	C17A	C14A	23.8(3)
C6A	C7A	C8A	C9A	0.7(5)	B1A	O3A	C17A	C18A	146.0(2)
C5C	C6C	C7C	C8C	0.6(5)	B1A	O3A	C17A	C19A	-95.1(3)
C1A	C2A	C3A	C5A	93.0(3)	B1A	C1A	C2A	C3A	-143.6(3)
C1A	C2A	C3A	C4A	-143.8(3)	B1A	C1A	C2A	N1A	114.2(2)
C1A	C2A	N1A	S1A	-109.7(2)	B1A	C1A	N1A	C2A	-101.0(3)
C2C	N1C	C1C	B1C	-100.1(3)	B1A	C1A	N1A	S1A	0.4(3)
C2C	N1C	C1C	Si3	113.4(2)	C3A	C5A	C10A	C9A	-178.6(3)
C2C	N1C	S1C	O1C	62.34(19)	C3A	C2A	N1A	C1A	-121.0(3)
C2C	N1C	S1C	C20C	174.12(17)	C3A	C2A	N1A	S1A	129.2(2)
C2C	C1C	B1C	O3C	40.5(3)	N1A	C1A	C2A	C3A	102.2(3)
C2C	C1C	B1C	O2C	-160.6(2)	N1A	C1A	B1A	O2A	-94.5(3)
C2B	C1B	N1B	S1B	100.6(2)	N1A	C1A	B1A	O3A	105.7(3)
C2B	C1B	B1B	O8	-163.4(2)	N1A	C2A	C3A	C5A	166.0(2)
C2B	C1B	B1B	O9	36.0(3)	N1A	C2A	C3A	C4A	-70.8(3)
C17A	O3A	B1A	O2A	-8.6(3)	N1A	S1A	C20A	C22A	65.6(2)
C17A	O3A	B1A	C1A	153.4(2)	N1A	S1A	C20A	C23A	-57.4(2)
C5A	C6A	C7A	C8A	0.5(5)	N1A	S1A	C20A	C21A	-177.04(18)
C2A	C1A	B1A	O2A	-161.2(2)	C20A	S1A	N1A	C1A	106.85(19)
C2A	C1A	B1A	O3A	38.9(3)	C20A	S1A	N1A	C2A	173.99(17)
C2A	C1A	N1A	S1A	101.4(2)	C19C	C17C	C14C	O3C	-148.8(3)

Table D.6 Torsion Angles for mo_MP915_0m_a.

A	B	C	D	Angle/˚	A	B	C	D	Angle/˚
C1B	C2B	N1B	S1B	-110.5(2)	C19C	C17C	C14C	C16C	-34.4(4)
C1B	C2B	C3B	C5B	81.0(3)	C19C	C17C	C14C	C15C	92.9(4)
C1B	C2B	C3B	C4B	-155.3(3)	C19B	C17B	C14B	O9	-92.1(3)
N1B	S1B	C20B	C21B	-178.21(18)	C19B	C17B	C14B	C15B	153.8(3)
N1B	S1B	C20B	C23B	64.7(2)	C19B	C17B	C14B	C16B	26.7(4)
N1B	S1B	C20B	C22B	-58.7(2)	B1B	O8	C17B	C14B	-15.2(3)
N1B	C2B	C1B	Si1	-101.9(2)	B1B	O8	C17B	C18B	-139.6(3)
N1B	C2B	C1B	B1B	112.6(2)	B1B	O8	C17B	C19B	102.5(3)
N1B	C2B	C3B	C5B	153.6(2)	B1B	O9	C14B	C17B	-20.3(3)
N1B	C2B	C3B	C4B	-82.6(3)	B1B	O9	C14B	C15B	97.5(3)
N1B	C1B	B1B	O8	-96.9(3)	B1B	O9	C14B	C16B	-144.5(3)
N1B	C1B	B1B	O9	102.4(3)	B1B	C1B	N1B	S1B	-1.0(3)
C22C	C20C	S1C	O1C	-64.4(2)	B1B	C1B	N1B	C2B	-101.6(3)
C22C	C20C	S1C	N1C	-176.89(18)	B1C	O3C	C14C	C16C	-92.6(3)
C21C	C20C	S1C	O1C	178.40(18)	B1C	O3C	C14C	C15C	149.5(3)
C21C	C20C	S1C	N1C	65.9(2)	B1C	O3C	C14C	C17C	25.2(3)
C7A	C6A	C5A	C10A	-1.3(4)	B1C	O2C	C17C	C18C	-92.5(3)
C7A	C6A	C5A	C3A	178.2(3)	B1C	O2C	C17C	C19C	148.6(2)
C8A	C9A	C10A	C5A	0.3(4)	B1C	O2C	C17C	C14C	26.2(3)
C10B	C5B	C6B	C7B	0.3(4)	B1C	C1C	C2C	N1C	115.1(2)
C10B	C5B	C3B	C2B	11.9(4)	B1C	C1C	C2C	C3C	-142.7(3)
C10B	C5B	C3B	C4B	-110.1(3)	Si3	C1C	C2C	N1C	-98.9(2)
C10B	C9B	C8B	C7B	0.5(5)	Si3	C1C	C2C	C3C	3.3(4)
C6B	C5B	C10B	C9B	-0.3(4)	Si3	C1C	B1C	O3C	-107.3(3)
C6B	C5B	C3B	C2B	-168.6(2)	Si3	C1C	B1C	O2C	51.6(3)
C6B	C5B	C3B	C4B	69.4(3)	C17C	O2C	B1C	O3C	-11.5(3)
C6B	C7B	C8B	C9B	-0.5(4)	C17C	O2C	B1C	C1C	-171.7(2)
C8B	C7B	C6B	C5B	0.1(4)	C14C	O3C	B1C	O2C	-9.6(3)

Table D.6 Torsion Angles for mo_MP915_0m_a.

A	B	C	D	Angle/˚	A	B	C	D	Angle/˚
C8B	C9B	C10B	C5B	-0.1(4)	C14C	O3C	B1C	C1C	151.7(3)
C6C	C5C	C10C	C9C	0.6(4)	C3C	C5C	C6C	C7C	178.8(3)
C6C	C5C	C3C	C2C	3.4(4)	C3C	C5C	C10C	C9C	-179.4(3)
C6C	C5C	C3C	C4C	-118.8(3)	C3B	C5B	C10B	C9B	179.2(3)
C6C	C7C	C8C	C9C	0.7(5)	C3B	C5B	C6B	C7B	-179.2(3)
C10C	C5C	C6C	C7C	-1.2(4)	C3B	C2B	C1B	Si1	-0.6(4)
C10C	C5C	C3C	C2C	-176.6(2)	C3B	C2B	C1B	N1B	101.3(3)
C10C	C5C	C3C	C4C	61.2(4)	C3B	C2B	C1B	B1B	-146.1(3)
C10C	C9C	C8C	C7C	-1.2(4)	C3B	C2B	N1B	S1B	128.3(2)
C14A	O2A	B1A	O3A	-11.8(3)	C3B	C2B	N1B	C1B	-121.2(3)
C14A	O2A	B1A	C1A	-172.9(2)	C20B	S1B	N1B	C2B	175.65(17)
C23C	C20C	S1C	O1C	56.0(2)	C20B	S1B	N1B	C1B	108.71(19)

Table D.7 Hydrogen Atom Coordinates (Å×10^4) and Isotropic Displacement Parameters (Å^2×10^3) for mo_MP915_0m_a.

Atom	*x*	*y*	*z*	U(eq)
H3C	6926(18)	-1830(30)	3722(13)	23
H3A	246(18)	7940(40)	291(13)	28
H3B	6226(18)	3230(40)	3142(13)	25
H6A	-667.96	10724.85	649.17	24
H2C	7403.32	674.06	3988.25	18
H4CA	7209.62	-1508.2	2720.68	35
H4CB	7998.6	-1318.76	3130.6	35
H4CC	7506.1	-109.43	2894.38	35
H2B	5837.27	5813.18	2781.05	18
H12G	340.77	6816.78	2623.85	43
H12H	158.56	8303.81	2667.05	43
H12I	1053.9	7820.33	2580.49	43

Table D.7 Hydrogen Atom Coordinates (Å×10^4) and Isotropic Displacement Parameters (Å^2×10^3) for mo_MP915_0m_a.

Atom	*x*	*y*	*z*	U(eq)
H2A	664.99	10524.65	591.66	20
H9A	-2190.16	8380.21	-741.79	29
H7B	8751.55	3362.28	4049.43	31
H9B	8627.06	6361.16	2923.78	28
H22D	10554.9	405.33	4422.94	32
H22E	10807.11	-398.01	5005.49	32
H22F	10253.98	830.73	5066.44	32
H11A	5919.65	3373.44	740.85	58
H11B	5517.56	2187.71	1063.44	58
H11C	6392.14	2057.66	798.33	58
H13D	7034.3	-3002.7	6030.29	45
H13E	6914.04	-1500.64	6079.99	45
H13F	7784.61	-2073.3	5976.92	45
H9C	4541.21	-1376.13	2629.02	31
H21D	9322.2	-2346.32	4255.79	35
H21E	10241.88	-2336.69	4469.11	35
H21F	9950.22	-1487.37	3911.42	35
H7A	-1990.15	11306.49	424.77	29
H8A	-2751.93	10149.97	-278.24	30
H10B	7262.84	5981.66	2750.77	24
H6B	7390.66	2977.32	3878.56	26
H13G	-862.78	7552.38	1050.8	47
H13H	-1028.06	8382.24	1634.3	47
H13I	-979.87	6869.24	1682	47
H21A	3080.35	5808.99	1620.52	38
H21B	2758.65	5335.02	2250.4	38
H21C	2544.72	4554.89	1653.07	38

Table D.7 Hydrogen Atom Coordinates (Å×10^4) and Isotropic Displacement Parameters (Å^2×10^3) for mo_MP915_0m_a.

Atom	*x*	*y*	*z*	U(eq)
H8B	9372.2	5067.98	3577.88	30
H6C	6073.18	936.61	4028.85	25
H10C	5851.23	-1988.51	2863.35	26
H22G	3271.5	8510.48	509.97	42
H22H	2677.71	7604.37	861.9	42
H22I	3595.23	7710.24	1074.54	42
H7C	4763.81	1566.48	3778.19	29
H15G	1468.59	12784.86	2119.39	46
H15H	1857.2	12282.67	2736.99	46
H15I	1236.87	13437.38	2737.69	46
H23G	2216.7	8200.08	1862.53	38
H23H	2445.64	9548.76	2147.6	38
H23I	3092.17	8430.97	2145.9	38
H16D	5497.85	1509.79	5511.77	46
H16E	5730.29	1925.55	6182.77	46
H16F	6054.76	613.79	5922.96	46
H23D	8879.43	-1748.64	5254.87	35
H23E	9145.09	-427.44	5551.39	35
H23F	9755.37	-1595.54	5549.65	35
H10A	-871.6	7816.51	-528.48	27
H21G	3802.69	10464.48	995.53	42
H21H	4077.75	9753.76	1599.31	42
H21I	3470.58	10923.63	1624.61	42
H16G	141.9	10795.45	3301.79	49
H16H	279.33	12275.94	3425.1	49
H16I	1014.27	11309.58	3482.98	49
H23A	3099.11	2645.38	2196.18	42

Table D.7 Hydrogen Atom Coordinates (Å×10[4]) and Isotropic Displacement Parameters (Å[2]×10[3]) for mo_MP915_0m_a.

Atom	*x*	*y*	*z*	U(eq)
H23B	3402.44	3458.73	2762.56	42
H23C	4021.27	2613.02	2402.3	42
H4BA	5895.09	5362.08	3875.4	41
H4BB	6055.99	3948.89	4110.84	41
H4BC	5272.83	4255.63	3711.08	41
H18G	279.16	13807.27	1894.86	44
H18H	-249.44	13879.59	2480.12	44
H18I	-664.25	13573.24	1843.09	44
H8C	4001.53	428.49	3073.66	30
H4AA	482.76	8431.73	-699.02	43
H4AB	1276.46	8673.38	-300.87	43
H4AC	729.52	9845.45	-509.85	43
H22A	3637.28	3408.54	1127.1	39
H22B	4510.73	3319.58	1432.24	39
H22C	4208.08	4626.29	1140.75	39
H19G	-1239.95	11527.5	2098.89	43
H19H	-985.8	11815.54	2780.8	43
H19I	-725.15	10511.42	2475.65	43
H12D	7523.4	-3382.45	4273.44	40
H12E	7233.38	-4256.57	4811.43	40
H12F	8077.5	-3548.04	4862.71	40
H11D	5696.32	-1327.9	5091.21	46
H11E	5699.07	-2845.82	5104.11	46
H11F	5810.82	-2109.71	4485.6	46
H18D	8153.21	3003.17	5482.22	64
H18E	8573.3	2544.83	6096.39	64
H18F	7929.71	3666.77	6099.77	64

Table D.7 Hydrogen Atom Coordinates (Å×10^4) and Isotropic Displacement Parameters (Å^2×10^3) for mo_MP915_0m_a.

Atom	*x*	*y*	*z*	U(eq)
H11G	871.52	6456.94	874.76	40
H11H	621.88	5573.22	1421.29	40
H11I	1434.48	6370.8	1463.25	40
H15A	5560.63	9270.84	1047.35	47
H15B	5713.8	9101.82	1752.78	47
H15C	5102.08	8183.53	1401.28	47
H18A	5715.57	8070.56	68.25	72
H18B	4993.55	7529.14	447.69	72
H18C	5396.1	6658.67	-47.64	72
H16A	7506	7851.55	1174.73	49
H16B	7152.24	8934.78	1593.79	49
H16C	7039.79	9032.67	881.88	49
H19D	6885.35	1025.42	6707.57	57
H19E	7036.28	2508.57	6813.53	57
H19F	7766.81	1532.58	6864.65	57
H19A	6704.7	5629.74	153.76	68
H19B	7233.81	6072.67	722.87	68
H19C	7066.55	7034.36	179.77	68
H13A	7827.95	2993.94	1435.76	65
H13B	7767.67	3955.89	1986.78	65
H13C	7514.97	4411.31	1325.35	65
H15D	6914.86	3974.86	5290.02	59
H15E	6367.75	3986.62	5866.41	59
H15F	5987.06	3623.51	5224.69	59
H12A	5959.97	1601.99	2376.27	71
H12B	6854.93	1960.38	2580.23	71
H12C	6697.29	1033.13	2022.48	71

Crystal Data for $C_{69}H_{120}B_3N_3O_9S_3Si_3$ (M =1348.55 g/mol): monoclinic, space group $P2_1$ (no. 4), a = 16.7481(16) Å, b = 10.5412(7) Å, c = 22.3460(15) Å, β = 91.513(3)°, V = 3943.7(5) Å^3, Z = 2, T = 100(2) K, μ(MoKα) = 0.191 mm^{-1}, $Dcalc$ = 1.136 g/cm^3, 43633 reflections measured (1.824° ≤ 2Θ ≤ 63.218°), 23214 unique (R_{int} = 0.0440, R_{sigma} = 0.0730) which were used in all calculations. The final R_1 was 0.0521 (I > 2σ(I)) and wR_2 was 0.1300 (all data).

References

[1] A. Marotta, H. Fang, C. E. Adams, K. Sun Marcus, C. G. Daniliuc, J. J. Molloy, *Angew. Chem. Int. Ed.* **2023**, *62*, e202307540.

[2] a) T. Fang, L. Xu, Y. Qin, N. Jiang, C. Liu, *Chin. J. Org. Chem.* **2023**, *43*, 777-780; b) F. C. Gao, M. Li, H. Y. Gu, X. Y. Chen, S. Xu, Y. Wei, K. Hong, *J. Org. Chem.* **2023**, *88*, 14246-14254.

[3] R. S. Dawood, I. Georgiou, R. P. Wilkie, W. Lewis, R. A. Stockman, *Chem. Eur. J.* **2017**, *23*, 11153–11158.

[4] H. H. Jung, A. W. Buesking, J. A. Ellman, *Org. Lett.* **2011**, *13*, 3912–3915.

[5] G. Liu, D. A. Cogan, T. D. Owens, T. P. Tang, J. A. Ellman, *J. Org. Chem.***1999**,*64*, 1278-1284.

[6] L. Tarifa, E. Apaloo-Messan, P. Dominguez-Molano, L. Morán-González, O. Salvadó, S. Wilkinson, M. Rodríguez, F. Maseras, E. Fernández *Chem.Eur.*, **2025**, DOI: 10.1002/ceur.202500241
